# Supplementary material for: Clinical efficacy of prebiotics and glycosaminoglycans versus placebo In dogs with food responsive enteropathy receiving a hydrolyzed diet: A pilot study
Source: PLoS One. 2021 Oct 21;16(10):e0250681. doi: 10.1371/journal.pone.0250681 (PMC8530283; doi:10.1371/journal.pone.0250681)

Final Study Report

Study Number: CIC-15-BIO

Study Title: A study investigating the efficacy of BIOEC2015 in reducing the clinical signs associated with canine inflammatory bowel disease (IBD)

Sponsor:
BIOIBERICA S.A.U.
Plaza Francesc Macià, 7
08029 Barcelona
SPAIN

Study Performed By:
Queen Mother Hospital for Animals
Clinical Investigation Centre
Royal Veterinary College
Hawkshead Lane
North Mymms
Hatfield
Hertfordshire, AL9 7TA
United Kingdom

Period of Investigation (Animal Phase):
September 2015 to April 2018

Contents

1. ABBREVIATIONS 6
2. STATEMENTS OF APPROVAL AND COMPLIANCE 8
2.1. Investigator 8
2.2. Sponsor Representative 9
2.3. Monitor 10
3. STUDY PERSONNEL AND SITES 11
3.1. Key Personnel 11
3.2. Study Sites 13
4. INTRODUCTION 14
4.1. Study Identifier 14
4.2. Objective 14
4.3. Background 14
4.4. Key Dates 14
5. MATERIALS AND METHODS 15
5.1. Study Sites 15
5.2. Test Animals 15
5.3. Housing of Animals 15
5.4. Feed and Water 15
5.5. Animal Inclusion and Exclusion Criteria 16
5.5.1. Inclusion Criteria 16
5.5.2. Exclusion Criteria 16
5.6. Post – Inclusion Removals 17
5.7. Animal Fate 17
5.8. Concomitant Treatments 17
5.9. Investigational Veterinary Product (IVP) and placebo 17
5.9.1. Investigational Veterinary Product 17
5.9.2. Placebo 18
5.9.3. Supply and Storage of IVP 19
5.10. IVP Use and Fate 20
5.11. Randomisation and blinding 20
5.12. Sample fate 21

6. EXPERIMENTAL DESIGN 21
6.1. Study Schedule 21
7. OBSERVATIONS AND MEASUREMENTS 21
7.1. Physical Examination 21
7.2. CCECAI and CIBDAI 22
7.3. Diagnostic samples: blood, urine and faeces 22
7.3.1. Blood samples 22
7.3.2. Biochemistry profile 22
7.3.3. Haematology profile 22
7.3.4. Urine sample 23
7.3.5. Faecal sample 23
7.4. Ultrasound 23
7.5. Endoscopy 23
7.5.1. Procedure 23
7.5.2. Histological scoring of biopsies 23
7.6. Adverse Events 23
7.7. Efficacy Assessments 24
7.8. Study End Points 24
8. RECORDS AND REPORTS 24
8.1. Record Storage and Archiving 24
8.2. Protocol Amendments 25
8.3. Protocol Deviations 26
8.4. Preparation of the Investigator Study Report 26
8.5. Data Handling 26
9. DATA ANALYSIS 26
9.1 Statistical Analysis: 26
9.2 Data exclusions: 26
10. RESULTS 26
10.1 Study Population 26
10.2 Evaluation of Efficacy 27
11. DISCUSSION 27
12. CONCLUSIONS 27
13. REFERENCES 28

14. APPENDICES 28
APPENDIX 1 29
TABLE A1. Group 1 Signalment and enrolment data 29
TABLE A2. Group 2 Signalment and enrolment data. 30
TABLE A3. Patient history. 31
TABLE A4. Completion and withdrawal. 35
TABLE A5. Biochemistry results. 37
TABLE A6. Biochemistry - laboratory and investigator comments. 42
TABLE A7. Albumin results for both treatment groups. 47
TABLE A8. Folate results for both treatment groups. 49
TABLE A9. Cobalamin results for both treatment groups. 51
TABLE A10. Screening blood tests 53
TABLE A11. Haematology results for treatment group 1. 55
TABLE A12. Laboratory and investigator comments on haematology results group 1. 58
TABLE A13. Haematology results for treatment group 2. 61
TABLE A14. Laboratory and investigator comments on haematology results group 2. 64
TABLE A15. Urinalysis Group 1. 67
TABLE A16. Urinalysis Group 2 71
TABLE A17. Faecal tests. 74
TABLE A18. IBD Physical exam. 76
TABLE A19. Drinking. 80
TABLE A20. Diet. 81
TABLE A21. General physical exam. 86
TABLE A22. CCECAI Scores. 92
TABLE A23. CIBDAI scores. 99
TABLE A24. WSAVA scores, stomach fundus. 106
TABLE A25. WSAVA scores – Stomach antrum. 108
TABLE A26. WSAVA scores – duodenum. 110
TABLE A27. WSAVA score - ilium. 112
TABLE A28. WSAVA score – colon 114
TABLE A29. UGI Part 1 Equipment used and sampling type. 116
TABLE A30. UGI Part 1 oesophageal lesion code. 119
TABLE A31. UGI part 1 Stomach lesion code. 121

TABLE A32. UGI part 1- Duodenum lesion code. 123
TABLE A33. UGI part 2 stomach lesion code. 126
TABLE A34. UGI part 2 Duodenum lesion code. 129
TABLE A35. LGI part 1 equipment and sampling. 132
TABLE A36. LGI part 1. 138
TABLE A37. LGI Part 2. 140
TABLE A38. LGI part 2 cecum 143
TABLE A39. Deviations. 145
TABLE A40. Concomitant medication - Group 1. 152
TABLE A41. Concomitant medication - Group 2. 157
TABLE A42. Adverse Events 161
TABLE A43. Adverse Events - treatment 164
TABLE A44. Clinical pathology results - Group 1. 166
TABLE A45. Clinical pathology results - Group 1. Diagnosis and comments. 181
TABLE A46. Clinical pathology results -Group 2. 190
TABLE A47. Clinical pathology results - Group 2. Diagnosis and comments. 200
APPENDIX 2 206
Table 1. Descriptive statistics of demographic variables (Group, Age, Gender, and Breed) with data summary after withdrawals within parentheses. 206
Figure 1. Group comparison of average (±sd) CCECAI and CIBDAI profiles 208
Figure 2. Group comparison of average (±sd) profiles for blood samples 209
Figure 3. Group comparison of average (±sd) Endoscopy Score D, I, C profiles 210
Figure 4. Group comparison of average (±sd) profiles for WSAVA scores 211
Figure 5. Pearson correlation coefficients (combining all the visits and groups 1 and 2) using *corrplot* package in R. 212

1. ABBREVIATIONS

ACTH	Adrenocorticotropic hormone
AE	Adverse event
ALP	Alkaline phosphatase
ALT	Alanine aminotransferase
BCS	Body Condition Score
CCECAI	Canine Chronic Enteropathy Clinical Activity Index
CIBDAI	Canine IBD Activity Index
CIC	Clinical Investigations Centre
CK	Creatine kinase
cPLI	Canine pancreatic lipase immunoreactivity
DOB	Date of birth
GCP	Good Clinical Practice
HCT	Hematocrit
HGB	Haemoglobin
IBD	Inflammatory bowel disease
IVP	Investigational veterinary product
LGI	Lower gastrointestinal
MCH	Mean cell haemoglobin
MCHC	Mean corpuscular haemoglobin concentration
MCV	Mean corpuscular volume
NTF	Note to file
PLT	Platelet count
QMHA	Queen Mother Hospital for Animals
NSAID	Non-steroidal anti-inflammatory drug
RDW	Red blood cell distribution width
RTAP	Random treatment allocation plan
RVC	Royal Veterinary College

SID	Once daily
SOP	Standard operating procedure
TLI	Trypsin-like Immunoreactivity test
RBC	Red blood cell count
UGI	Upper gastrointestinal
V1	Visit 1
V2	Visit 2
V3	Visit 3
V4	Visit 4
V5	Visit 5
V6	Visit 6
V7	Visit 7
WBC	White blood cell count
WSAVA	World Small Animal Veterinary Association

2. STATEMENTS OF APPROVAL AND COMPLIANCE

2.1. Investigator

Work conducted at the RVC was performed according to the VICH GL9 (GCP). This included conduct of the in-life phase and associated raw data collection.

All RVC generated study documentation was provided to the report authors and represents an accurate and record of the study.

No claim of compliance is made for work undertaken at Iowa State University this includes statistical analysis, and any results and conclusions drawn from them.

______________________________________ ____________________

Dr. Barbara Glanemann Dipl ECVIM-CA, PGCVetEd FHEA, MRCVS Date

Lead Investigator

______________________________________ ____________________

Prof Karin Allenspach Dr med vet FVH ECVIM-CA PhD Date

Co-Investigator

2.2. Sponsor Representative

As Sponsor for this study I confirm that I approve the content and format of this report as being appropriate for reporting the conduct of this study by the Investigator of this study.

____ ___________________

Sergi Segarra DVM MSc PhD Date

Sponsor Representative

2.3. Monitor

This study has been monitored according to RVC Standard Operating Procedures. The Sponsor was advised on the progress of the study by the Monitor following periodic inspection of various phases. The dates of monitoring visits and the phases inspected are given below:

Date of Inspection	Phase
23Sep15	Pre-study/study set-up
15Mar16	Interim/in-phase
15Nov16-17Nov16	Interim/in-phase
24Jan19	Closeout

Work at the RVC was conducted in compliance with the Protocol, Protocol Amendments and relevant Standard Operating Procedures.

The report describes accurately the methods and procedures used for data collection.

Verification of results and conclusions has not been undertaken and VICH GL9 (GCP) compliance is not claimed for this part of the work.

____ ____________________

Mrs Linda Slater DipAVN(surg) RVN MRQA Date

Monitor

3. STUDY PERSONNEL AND SITES

3.1. Key Personnel

Sponsor	Sergi Segarra<br>R&D Project Leader Animal Health<br>R&D Department<br>BIOIBERICA S.A.U.<br>Plaza Francesc Macià, 7<br>08029 Barcelona<br>SPAIN
Lead Investigator	Barbara Glanemann<br>Queen Mother Hospital for Animals<br>Royal Veterinary College<br>Hawkshead Lane<br>North Mymms<br>Hatfield<br>Hertfordshire, AL9 7TA<br>United Kingdom
Co-Lead Investigator	Karin Allenspach<br>Professor of Internal Medicine<br>1452 Vet Med<br>1800 Christensen Drive<br>Ames, IA 50011-1134<br>USA
Monitor	Linda Slater<br>Clinical Investigations Centre<br>Royal Veterinary College

	Hawkshead Lane<br>North Mymms<br>Hatfield<br>Hertfordshire, AL9 7TA<br>United Kingdom
Co-Study Report Author	Linda Slater<br>Clinical Investigations Centre<br>Royal Veterinary College<br>Hawkshead Lane<br>North Mymms<br>Hatfield<br>Hertfordshire, AL9 7TA<br>United Kingdom
Co-Study Report Author	Karin Allenspach<br>Professor of Internal Medicine<br>1452 Vet Med<br>1800 Christensen Drive<br>Ames, IA 50011-1134<br>USA
Statisticians	Yeon-Jung Seo, BSC MS PhD<br>Post-doctoral research associate<br>Biomedical Sciences<br>Iowa State University<br>Ames<br>IA<br>USA

Jonathan P. Mochel, DVM, MS, PhD, DECVPT

Associate Professor of Pharmacology and mathematical modelling

Biomedical Sciences

Iowa State University

Ames

IA

USA

3.2. Study Sites

The study was conducted at:

Queen Mother Hospital for Animals (QMHA)

Royal Veterinary College

Hawkshead Lane

North Mymms

Hatfield

Hertfordshire

AL9 7TA

UK

Data was collected according to the study protocol and VICH GL9 (GCP).

The study was performed under the Animals (Scientific Procedures) Act 1986, PPL number: 70/7393.

Statistical analysis was performed at:

Biomedical Sciences

Iowa State University
Ames
IA

USA

Compliance with VICH GL9 (GCP) is not claimed for this work or any results or conclusions drawn from it.

4. INTRODUCTION

4.1. Study Identifier

Study Number: CIC-15-BIO

Study Title: A study investigating the efficacy of BIOEC2015 in reducing the clinical signs associated with canine inflammatory bowel disease (IBD)

4.2. Objective

The study aim was to test the hypothesis that BIOEC2015 is effective in reducing the clinical signs associated with canine inflammatory bowel disease (IBD).

The study objective was to evaluate the clinical efficacy of BIOEC2015 in a placebo-controlled, double-blinded clinical trial based on responses given in owner-administered questionnaires and the Canine Chronic Enteropathy Clinical Activity Index (CCECAI).[1]

4.3. Background

It is currently accepted that IBD in dogs – as in humans – is the consequence of a deranged immune response to luminal bacteria in genetically susceptible individuals.[2] The interaction of receptors of the innate immune system – so-called pattern-recognition receptors, PRRs – are expressed by epithelial cells and antigen-presenting cells of the intestinal mucosa. Luminal bacteria seem to play an important role both in the maintenance of tolerance towards commensal bacteria and, in the case of pathogens, in mounting an appropriate inflammatory response.

BIOEC2015 (the compound) is hypothesised to exert its beneficial effects in canine IBD by:

Changing the intestinal microbiome composition by binding to certain TLRs and thereby replacing commensal bacteria that may act as triggers in canine IBD increasing anti-inflammatory cytokine production (IL-10) and decreasing pro-inflammatory cytokine production (IL-1beta, TNF) in the intestinal epithelium.

It was hypothesized that the individual animals receiving the compounds would benefit from the clinical trial and – if *the compounds* are proven to be efficacious – all dogs with IBD in the future. Furthermore, it was hoped that the results of this study would have important clinical implications for the understanding of the pathogenesis of canine IBD. The results may also be relevant for the treatment of human IBD, for which the canine disease may be considered an outbred model.

Ultimately, the aim was to advance the treatment of canine IBD in veterinary medicine.

4.4. Key Dates

The study was conducted according to the authorised protocol and protocol amendments 1-5.

Protocol Signing and Study Initiation	15th September 2015
Experimental Phase Commenced (first “animal task”)	13th October 2015
Experimental Phase Completed (last “animal task”)	24th April 2018
Study Completion	See Investigator signature on page 8

5. MATERIALS AND METHODS

5.1. Study Sites

This study was conducted at one Investigator site in the United Kingdom. This site is a specialist referral hospital. Statistical analysis was performed at Iowa State University.

5.2. Test Animals

Dogs are the intended target species for CIC-15-BIO.

Twenty dogs were enrolled (10 in each treatment group). These consisted of male and female dogs (entire and neutered) of various breeds and crossbreeds aged between approximately 14 and 131 months of age and weighing between 7.85kg and 39.10kg.

Dogs enrolled onto the study were client-owned and met all of the inclusion criteria and none of the exclusion criteria (as detailed in section 5.5). Dogs were identified by name and owner's surname. In addition to this each dog had a unique study identifier assigned at enrolment (consisting of the pre-fix "RVB", followed by a numerical number e.g. RVB01). Dogs were enrolled through the Internal Medicine Service at the QMHA. Animal details can be seen in Appendix 1, Table A1 and A2.

5.3. Housing of Animals

For the majority of the study, dogs were housed in their home environment with their owners. When at the Investigator site for study procedures and if required, dogs were housed in the kennel unit at the QMHA as per standard veterinary practice.

5.4. Feed and Water

The dogs were fed a commercial hydrolysed diet (Purina Veterinary Diets™ HA Hypoallergenic™) by their owners from screening until visit 4. When in their home environment water was provided adlib.

When hospitalised, dogs were fed the standard patient diet (visit 1) or Purina HA (Visit3) and provided with water ad lib (except when withheld for medical reasons e.g. prior to anaesthesia).

Following visit 4 with the exception of RVB29 the owners attempted to reintroduce the pet's usual diet (as chosen by their owner) over the next 7days. Where worsening of clinical signs occurred Pruina HA was reintroduced solely or in part. Five dogs (RVB16, 20, 32, 34 and 35) were successfully returned to their previous diet after gradually reintroducing it following V4. See Appendix 1 Table A20 for further details.

RVB13 reintroduced Chappie between V4 and V5, the dog clinically worsened and the dog returned to Purina but the owner requested that she try Chappie again re-introducing more slowly. The dog was later changed exclusively to Purina HA after worsening again, however, the owner began feeding small amounts of WAGG diet in addition to Purina HA four days prior to V7.

RVB18 was reported to vomit chicken following V3 when it should have exclusively been fed Purina HA; the owner does not know where it scavenged the chicken from. Due to this there was a delay in returning the dog to their usual diet after V4. Prior to re-introducing the dog's normal diet, it scavenged dog food from elsewhere and was sick again. The owner fed Purina HA for the remainder of the study.

5.5. Animal Inclusion and Exclusion Criteria

5.5.1. Inclusion Criteria

Dogs which fulfilled the following inclusion criteria were eligible for recruitment in the study:

- Dogs >/=6 months of age on Day 1
- Dogs >/=5kg on Visit 1
- Chronic history (>3 weeks) of vomiting and/or diarrhoea
- Confirmed diagnosis of IBD based on clinical and histopathological findings, and diet responsive to Purina HA.
- Dogs deemed not to be suffering from any additional disease process as listed below
    - Metabolic disease
    - Neoplastic disease
    - Infectious or autoimmune disease
    - Cardiopulmonary disease that would increase the risks of general anaesthesia will be excluded from the study.
- Patients who have received antibiotic treatment can be included but treatment must have stopped 7 days prior to Day 1
- Homeopathic remedies are acceptable for patients on the study, this should be recorded on the Treatment Form
- Patients receiving NSAID’s can be included but must have stopped treatment 7 days prior to Day 1.
- Patients receiving glucocorticoid medication can be included but must be tapered off the drug and have completely stopped treatment 7 days prior to Day 1.

5.5.2. Exclusion Criteria

Dogs with the following criteria or concurrent diseases were excluded with the exception of RVB11 who had concurrent Giardia; data for this dog was assessed as suitable for inclusion by Karin Allenspach (see NTF):

- Dogs of less than 6 months of age on Day 1
- Dogs weighing <5kg on Visit 1
- Metabolic disease
- Neoplastic disease
- Infectious or autoimmune disease

- Cardiopulmonary disease that would increase the risks of general anaesthesia will be excluded from the study.
- Pregnant and lactating bitches or dogs intended for breeding
- Patients receiving antibiotic therapy within the 7 days prior to Day 1.
- Any dogs that have received glucocorticoid medication and/or non-steroidal anti-inflammatory drugs in the 7 days prior to Day 1 will be excluded from the study.
- Other causes of diarrhoea identified on clinical investigation
- Dogs who were not diet responsive to Purina HA.

5.6. Post – Inclusion Removals

Dogs discontinued the study for the following reasons:

Five dogs were withdrawn (RVB05, 07, 09, 12 and 28) because of worsening of clinical signs. These dogs were removed : RVB05 – prior to Visit 2; RVB07 –prior to Visit 3; RVB09 –at Visit 3, RVB12 –at Visit 3; and RVB28 –prior to Visit 3.

One dog (RVB27) was withdrawn because the dog refused to take the test compound. This dog received five part-doses over a two-week period.

One dog (RVB30) was withdrawn prior to Visit 3 because it required treatment with Apoquel and Cephalexin for a skin condition.

Two further dogs were replaced: RVB04 because the dog was diagnosed with adenocarcinoma after enrolment and RVB23 because the owner decided not to continue after administering only one dose of compound. Their treatments were reallocated following the same process as that described for the non-enrolled dogs.

5.7. Animal Fate

All dogs at the end of the study remained with their owners and were then subject to normal veterinary and husbandry practices.

5.8. Concomitant Treatments

Concomitant medications were approved by the Investigator. All concomitant medications were recorded, these are shown in Appendix 1, Table A40 and A41.

5.9. Investigational Veterinary Product (IVP) and placebo

5.9.1. Investigational Veterinary Product

Name:	BIOEC2015
Identity:	Product A (Treatment 1)
Appearance:	Reddish-brown powder

Odour: Odourless

Manufacturer's name: Bioiberica SAU

Contents: 2500mg resistant starch

300mg prebiotics (β-glucans and mannanoligosacharides (MOS))

200mg chondroitin sulphate

20mg glycosaminoglycans

560mg bentonite

400mg flavourings (hydrolysed; of pork and poultry origin)

20mg iron oxide

Supplied as: Boxes containing 30 sachets.

Batch Details: 2401

Expiry Date: 06-2018

Storage: Stored at room temperature away from moisture and heat.

Continuous monitoring of the room containing the IVP was undertaken using a digital memory monitoring thermometer; maximum and minimum temperatures were obtained and recorded from product receipt and for the study duration. The thermometer used was validated annually.

All supplies were stored in a secure limited access storage area.

Dose: 5.00-14.99kg: 1 sachet SID

15.00-34.99kg: 2 sachets SID

35.00kg or more: 3 sachets SID

The dose was mixed with food or sprinkled over it; dry food was wetted by some owners. For one dog (RVB27), it was necessary to dilute the powder with water and administered using a syringe.

5.9.2. Placebo

Name: Placebo

Identity: Product B (Treatment 2)

Appearance: Reddish-brown powder

Odour: Odourless

Manufacturer's name: Bioiberica SAU

Contents: 660mg bentonite

	400mg flavourings (hydrolysed; of pork and poultry origin)
	20mg iron oxide
Supplied as:	Boxes containing 30 sachets.
Batch Details:	K001
Expiry Date:	05-2018
Storage:	Stored at room temperature away from moisture and heat.
	Continuous monitoring of the room containing the IVP was undertaken using a digital memory monitoring thermometer; maximum and minimum temperatures were obtained and recorded from product receipt and for the study duration. The thermometer used was validated annually.
	All supplies were stored in a secure limited access storage area.
Dose:	5.00-14.99kg: 1 sachet SID
	15.00-34.99kg: 2 sachets SID
	35.00kg or more: 3 sachets SID

5.9.3. Supply and Storage of IVP

The IVP was supplied to the RVC by the Sponsor in one shipment and was received in good condition on 25Sep15.

The boxes containing sachets of IVP were stored at room temperature and dispensed to each owner at visits 1, 2 and 3.

The IVP was labelled as follows:

Product A / B

BIOIBERICA PRJ 00238 - IBD study RVC

Batch No.

Best before date

For Veterinary Clinical Trial Use Only

Store at room temperature away from heat and moisture

Manufactured by: BIOIBERICA S.A. Plaza Francesc Macià, 7. 08029 Barcelona (SPAIN)

For Animal Treatment only

Keep out of reach of children

5.10. IVP Use and Fate

The CIC was responsible for maintaining records including identification, quantities and dates of the test materials received, dispensed and returned. The Dispenser in the CIC completed records at each visit. Raw data associated with drug use and accountability is available in the study file.

Records of receipt, use and unused stock were maintained. Drug accountability was performed and any discrepancies investigated and explained. Drug returns and unused stock were disposed of in pharmaceutical waste by the test site.

5.11. Randomisation and blinding

Dogs who met the inclusion criteria at Visit 1 were assigned one of two treatment groups (product A or B) according to the random treatment allocation plan (RTAP).

The patients were discharged from the hospital once they had recovered sufficiently from anaesthesia and the owners told to feed Purina HA exclusively whilst awaiting biopsy results.

Owners were contacted approximately one-week post discharge to assess response to diet. Dogs who were diet responsive and met all other inclusion criteria were enrolled and owners told to start study medication. Dogs who had not responded to the hypoallergenic diet were fed Purina HA for a further week, reassessed and then enrolled or excluded from the study.

If the dog was not enrolled onto the study, its treatment allocation was added to the end of the RTAP and the treatment reallocated.

Recruitment continued until twenty dogs had been enrolled with 10 dogs in each group. Both groups were fed a hydrolysed (Purina HA) exclusive to any other food from screening until visit 4. Group A (n = 10 dogs) received the test compound BIOEC2015 in addition. Group B (n = 10 dogs) received a placebo designed by Bioiberica Ltd. that was identical appearance to the probiotic formulation.

Two further dogs were replaced: RVB04 because the dog was diagnosed with adenocarcinoma after enrolment and RVB23 because the owner decided not to continue after administering only one dose of compound. Their treatments were reallocated following the same process as that described for the non-enrolled dogs.

Both the dogs' owners and evaluators (including clinicians and those analysing samples and tissues) had no knowledge of the treatment group assignments.

The study nursing staff were not blinded. The study nursing staff acted as dispensers.

Randomisation was done using block randomisation with an allocation ratio of one-to-one, a random number generator in excel and a block size of 4. The RTAP was designed according to this. The CIC nurses had access to this and as dispensers sequentially allocated the study patients to a group.

No patients required un-blinding during the courses of the study.

5.12. Sample fate

No study samples other those sent for diagnostics were stored or analysed. Samples collected for research (sponsor and RVC) are not reported as part of this study.

6. EXPERIMENTAL DESIGN

6.1. Study Schedule

Each dog was evaluated as indicated in the table below.

Table 1 Schedule of Events

Visit Number	Physical examination (general and IBD)	CCECAI	Blood sample	Urine sample	Faecal sample (Diagnostic)	Ultrasound	Endoscopy
Visit 1	√	√	√	√	√	√	√
Visit 2	√	√	√				
Visit 3	√	√	√	√			√
Visit 7	√	√	√	√			

A telephone interview was conducted to confirm enrolment. This occurred approximately one week after endoscopy. If no improvement had been seen on Purina HA, the diet was fed for a further week and a second telephone interview conducted to established whether the dog could be included in the trial. Three dogs (RVB27, 28 and 34) required Purina HA to be fed for a further week before being confirmed as diet responsive and one dog (RVB35) had enrolment delayed by a week avoid appointments clashing with the owner's vacation.

Day 1 was defined as the first day of dosing.

Hospital Visits occurred at: Visit 2 occurred Day 14 (± 2days) except for one dog RVB28 whose Visit 2 occurred 1 day late at Day 17; Visit 3 occurred at Day 70(±2days); and Visit 7 at Day 126 (±5 days).

Telephone visits were conducted for Visit 4 at Day 77 (±5d); Visit 5 at Day 91 (±5 d) and Visit 6 at Day 105 (±5d).

Details of medication given during the courses of the study were documented at each site / telephone visit; these concomitant medications are shown in Appendix 1, Tables A40 and A41.

Patient history including any medication given within three months prior to screening was also documented (details available in study file) and the information used in conjunction with visit 1 assessments to ensure all inclusion/ exclusion criterial including drug withdrawal periods were met.

7. OBSERVATIONS AND MEASUREMENTS

7.1. Physical Examination

A physical examination was performed by the investigator at each site visit (Visits 1, 2, 3 and 7). This included a general examination of the respiratory, cardiac and musculo-skeletal systems. Abnormal findings were documented and classified as clinically / not clinically significant (see Appendix 1, Table A21).

An IBD assessment was also performed by the investigator, which included assessment of abdominal pain/discomfort, pruritus, and ascites/peripheral oedema (see Appendix 1, Table A18).

Information on drinking and diet was also obtained (see Appendix 1, Tables A19 and A20).

7.2. CCECAI and CIBDAI

The canine chronic enteropathy clinical activity index (CCECAI) consisted of the information assessed in the owner questionnaire, clinical findings (oedema formation, ascites and pruritus) and the serum albumin level and was performed at all site visits (Visit 1, 2, 3 and 7).

The Canine IBD Activity Index (CIBDAI) consisted only of information assessed in the owner questionnaire. It formed part of the CCECAI and was also conducted at each telephone visit (Visits 4, 5 and 6).

CCECAI and CIBDAI scores from scheduled visits are shown in Appendix 1, Tables A22 and A23.

7.3. Diagnostic samples: blood, urine and faeces

Diagnostic samples were submitted to the Clinical Pathology Laboratory at the Royal Veterinary College. Samples for RVB12 V2 were sent to external laboratory "Powell Torrance Diagnostic Services" for albumin levels as the RVC analyser was awaiting repair. All results were reviewed by the investigator and any out of range values assessed. The results and outcome of assessment are shown in Appendix 1, Tables A5 – A17).

Additional samples were collected and stored for research purposes (RVC and sponsor); these included blood, urine and faeces / faecal swabs at each site visit. These samples are not to be analysed as part of this study and are therefore not included in this report.

7.3.1. Blood samples

Test	Sample type	Frequency
Biochemistry **	Serum gel and fluoride oxalate	Visits 1 , 3 and 7
Albumin	Serum gel	Visit 2
Haematology **	EDTA	Visits 1, 3 and 7
TLI **	Serum gel	Visit 1 *
Folate and Cobalamin Concentrations **	Serum gel	Visit 1, 3 and 7
cPLI **	Serum gel	Visit 1
ACTH stimulation test **	Serum gel (pre and post synacthen administration)	Visit 1 *

* Where TLI and or ACTH stimulation test had been performed by the referring practice during the 3 months prior to Visit 1 this result was used and the test not repeated. All results are available in the study files.

** Blood results for RVB09 were used from a hospital visit undertaken one week prior to V1.

7.3.2. Biochemistry profile

Serum biochemistry profile included measurement of: Albumin, Globulin, Sodium, Potassium, Chloride, Calcium, Inorganic Phosphorous, Urea, Creatinine, Cholesterol, Total Bilirubin, Amylase, Lipase, ALT, CK and ALP. Where a fluoride oxalate tube was submitted, a glucose measurement was also performed; where this was not done, it was reported as a protocol deviation (see Appendix 1, Table A39).

7.3.3. Haematology profile

Haematology profile included: WBC, Neutrophils, Lymphocytes, Monocytes, Eosinophils, Basophils, RBC, HGB, HCT, MCV, MCH, MCHC, RDW and PLT.

7.3.4. Urine sample

Urinalysis was performed at visits 1 (except RVB27), 3 (except RVB09) and 7 (except RVB01, RVB16, RVB18) and included measurement urine specific gravity, dipsticks and sediment examination.

7.3.5. Faecal sample

Faecal samples were submitted for parasites and culture as part of the screening process undertaken at Visit 1. This was not done for RVB13, RVB20, RVB19, RVB27, and RVB30 where it was not possible to obtain a sample or RVB22 where it was not possible to obtain sufficient sample for faecal culture (see Deviation Table). A faecal sample could not be collected at V1 for RVB32 but a sample was submitted at V2 instead and this was negative for parasites and bacteriological examination.

7.4. Ultrasound

Abdominal ultrasound was performed at Visit 1 as part of the diagnostic evaluation.

7.5. Endoscopy

7.5.1. Procedure

Upper and lower GI Endoscopy was performed under general anaesthesia at Visit 1 and Visit 3. Animals were induced with propofol to effect and maintained on isofluorane / sevofluorane vapourised in oxygen. Study animals were fasted for 48hours prior to the endoscopy and prepared for colonoscopy using commercial and/ or warm water enemas +/- Kleen prep (see Appendix 1 Table A35). Biopsies were taken and placed in 10% formalin for diagnostic evaluation from the duodenum, colon (except RVB02 V1 were due to poor visibility this was not possible), ileum (when possible) and stomach when deemed clinically necessary by investigator.

Additional biopsies were collected and stored for research purposes (RVC and sponsor); these samples are not to be analysed as part of this study and are therefore not included in this report.

A decision was made to withdraw RVB09and RVB12 at V3 due to worsening of clinical signs; only partial data was collected and endoscopy was not performed.

7.5.2. Histological scoring of biopsies

Biopsies were scored by Dr Simon Priestnall, Dept of Pathobiology and Population Sciences (PPS), RVC. This was done in one sitting based on the WSAVA guidelines for the assessment of endoscopic intestinal biopsy samples. Histopathology results are shown in Appendix 1, Tables A24-A28.

Diagnostic evaluation of biopsies was performed by pathologists at the RVC Clinical Pathology Laboratory (see Appendix 1, Tables A44-46).

7.6. Adverse Events

An adverse event was defined as any observation in an animal that is unfavourable and unintended and occurs after the use of the veterinary product or investigational veterinary product (IVP) whether or not considered to be product related.

Details of adverse events and any therapy implemented were documented by the Investigator on an Adverse Event form, and concomitant medication form, where relevant. All adverse events occurring throughout the duration of the study were reported to the study monitor and sponsor.

For the purpose of this study, clinical signs associated with IBD, e.g. vomiting and diarrhoea were not documented as an adverse event unless thought to be IVP related or due to a cause other than inflammatory bowel disease.

No Serious Adverse Event occurred.

During the course of the study 13 adverse events (AEs) occurred, affecting 10 dogs; these are shown in Appendix 1 Tables A42: The causality was classified as: condition/disease diagnosed after enrolment (n=2), unknown/test article (n=3) and other (n=8). Four AEs required treatment (Appendix 1 Table A43).

7.7. Efficacy Assessments

CIBDAI and CCECAI scores were used to assess efficacy based on changes in the CCECAI/CIBDAI value. CIBDAI / CCECAI scores were obtained when site visits (V1, V2, V3 and V7) were performed; CIBDAI scores were also collected when telephone visits (V4-V6) were undertaken. Results are shown in Appendix 1 Tables A22 and A23.

Information about the dogs themselves was collected to assess similarity between groups. This information is shown in Appendix 1 Tables A1 and A2.

Blood tests and urine tests were performed at V1, V3 and V7, and histopathology on intestinal biopsies at V1 and V3 (with the exception of RVB29 and RVB31 at V3). A blood sample was also collected at V2 evaluation of albumin levels. Blood results are shown in Appendix 1 Tables A5-14; albumin results in Appendix 1 Table A7. urine results in Appendix 1 Tables A15 and A16; clinical pathology reports in Appendix 1 Tables A44-A46 and WSAVA scores in Appendix 1 Tables A24-28.

Data was compared between groups and within groups to assess similarity, disease severity and to assess improvement with treatment verse placebo. These results are shown in section 10 of the report.

7.8. Study End Points

Final assessment for the purposes of the study occurred at Visit 7. Thirteen dogs reached this time point with seven others requiring withdrawal prior to this visit (See Appendix 1 Table A4). Data from all completed visits was used for statistical analysis and is shown in the data tables in Appendix 1.

8. RECORDS AND REPORTS

8.1. Record Storage and Archiving

All documents relating to the conduct of the study were kept in a secure location at the RVC for the duration of the study.

All original study documentation will be retained at the RVC CIC archive facilities for a period of two years, at this time the sponsor will be contacted to decide on document retention.

An electronic copy of the study data will be sent to the sponsor following issue of the Final Study Report.

8.2. Protocol Amendments

Five formal amendments were made to the protocol. All were authorised by the sponsor, investigator and study monitor prior to initiation.

Protocol Amendments

Amendment Number	Summary of Amendments
1 (Issued 15Dec15)	- Biopsies were collected into ice cold culture medium (Biopsy sample D) at Visit 1 until 10 full sets (12 biopsies from duodenum, ileum and colon) had been obtained. No further Biopsy sample D were collected after this point.<br><br>- No biopsies needed collecting on ice cold culture medium (Biopsy sample D) at Visit 3.<br><br>- Form 11 changed to reflect changes in Biopsy sample D collection. Additional column added to allow person entering data to sign and date form.<br><br>- PPL number amended to 70/7393<br><br>- Title and qualifications amended for Lead Investigator and Study Monitor<br><br>- Full postal address of sponsor representative added.
2 (Issued 29Mar16)	- Lead investigator role transferred to Oliver Garden. Karin Allenspach transferred to role of co-investigator but remains responsible for compiling Final Study Report. Contact details for co-Karin Allenspach updated
3 (Issued 16Nov16)	- Lead investigator role transferred to Barbara Glanemann following departure of Oliver Garden from RVC on 2nd December 2016. Contact details for co-investigator Karin Allenspach updated.
4 (Issued 14Sep17)	- Form 11 changed to include biopsies for histopathology and list sample tube types required for each test. Investigator signature removed and pages labelled part A and part B; part B completed at Visits 1 and 3 only
5 (Issued16Jul18)	- One additional serum aliquot from Visits 1 and 3 are the property of the sponsor.

8.3. Protocol Deviations

Forty-nine protocol deviations were filed by the Investigator during the study period. A list of deviations including action taken and impact assessment can be found in the Appendix 1 Table A39. This also includes deviations relating to samples collected for non-study purposes (Sponsor and RVC).

The deviations listed do not impact the conclusions of the study.

8.4. Preparation of the Investigator Study Report

This is a final study report, which refers to the conduct of the study specified and appropriately summarises the data generated during the study.

8.5. Data Handling

Data generated was entered into spreadsheets (Microsoft� Excel 2016), which was electronically locked following 100% checking of the data.

9. DATA ANALYSIS

9.1 Statistical Analysis:

Statistical analysis was performed using R software version 3.5.1 (R Foundation for Statistical Computing, Vienna, Austria). Normality was tested for each variable using Shapiro-Wilk for all parametric approaches. When the assumption did not hold, the nonparametric test (Mann-Whitney) was used for the variables measured only at one (visit=1) or two time points (visits=1,3) to compare differences between two groups. With variables involving multiple time points (more than two), each outcome was analyzed using a linear mixed effects model for group comparison. For each analysis, time, group, and their interactions were included as a fixed effect, whereas each dog was treated as a random effect. To test the correlation between variables of interest spearman's rank correlation coefficients and the corresponding p-values were calculated. For all statistical analyses, a p-value < 0.05 was considered significant.

9.2 Data exclusions:

Data from the following unscheduled visits was not included in the analysis:

- Unscheduled visit for RVB07 on 04FEB16 (Withdrawal due to progressive weight loss and diarrhoea)
- Unscheduled visit for RVB09 on 18Feb16 (Incorrect visit time point)
- Unscheduled visit for RVB05 on 21Dec15 (Withdrawn as stopped IVP 01Dec15 and receiving treatment with steroids and cyclosporine since 05Dec15)
- Unscheduled visit for RVB28 on 26Jun17 (Withdrawal, last dose of IVP 05Jun17, treatment with prednisolone and antibiotics started 08Jun17)

The following data was also excluded from the analysis:

- Visit 2 data for RVB27 due to non-compliance with dosing

10. RESULTS

10.1 Study Population

Twenty (20) client-owned dogs were enrolled onto the study. Case allocation to Group 1 and 2 are shown in in Appendix 1 Table A1.

Dogs were randomly assigned to one of the two treatment groups (10 dogs in Group 1, and 10 in Group 2), and they ranged in age from 25 to 131 months for group 1 and 19 to 80 months for group 2, at the initial visit (Visit No =1). Descriptive statistics of demographic variables (Group, Age, Gender, and Breed) are given

in Table 1. All 20 dogs were included in the analysis, and there were no significant mean differences in outcome variables between the missing (13 completed) and nonmissing (20 enrolled) groups. Note that each variable was taken at different time points (among the visits from 1 through 7). For example, TLI and cPLI were measured only at the initial visit, while Albumin, Cobalamin, Folate and Globulin were measured at the visits 1, 3 and 7. An average profile for each group for some of the variables in the dataset are given in Figures 2,3, and 4.

10.2 Evaluation of Efficacy

No statistically significant differences were found in CCECAI (p-value=0.5798) and CIBDAI scores (p-value= 0.9520) between two groups (Figure 1). For blood samples, there were no group effects on average for Albumin (p-value=0.9358), Cobalamin (p-value=0.4094), Folate (p-value=0.445), Globulin (p-value= 0.3580), TLI (p-value=0.8292), and cPLI (p-value=0.7959), (Figure 2). There were also no significant time or group effects on PCV/haematocrit, RBC, erythrocyte indices or haemoglobin concentrations (p-value=0.932) There were also no significant time and group interactions in Endoscopy scores D (p-value=0.3778), I (p-value≈1), and C (p-value=0.8785), (Figure 3). The variable WSAVA (total) is defined as the sum of sub-total scores, that is, WSAVA (total)=SF SubTotal+SA SubTotal+D SubTotal+I SubTotal+C SubTotal. Testing for the interaction of time and group results in p-value= 0.8116 for SF SubTotal, 0.5462 for SA SubTotal, 0.1768 for D SubTotal, ≈1 for I SubTotal, and 0.5034 for C SubTotal, indicating no statistically significant interaction effects, as well as for WSAVA (total) with p-value = 0.4487 (Figure 4).

A graphical display of a correlation matrix is given in Figure 5 for the variables, CCECAI, CIBDAI, SF, SA, D, I and C sub-total scores, WSAVA total, Endoscopy D, I and C, Albumin, and Cobalamin. We see some strong positive correlations between D and I sub-totals and Endoscopy D and I scores. There are some negative correlations between Endoscopy scores (D and I, as well as D and I sub-totals) and Albumin and Cobalamin.

Individual animal data is presented in Appendix 2, Tables A1 and A2.

11. DISCUSSION

Of the 35 dogs enrolled into the clinical trial, 10 in each group reached the point of second endoscopy. A total of 13 dogs (n=8 in the group 1 and n=5 in group 2) reached the trial endpoint of 18 weeks. No statistically significant differences were found in CCECAI (p-value=0.4761) and CIBDAI scores (p=0.8510) between the two groups. Furthermore, there were no group effects on average for Albumin (p=0.9116), cobalamin (p=0.7170), folate (p=0.3530), globulin (p=0.1640), TLI (p0.5503), and cPLI (p=0.8238). There were also no significant time and group interactions in Endoscopy scores for the duodenum (p= 0.8162), ileum (p≈1), and colon (p=0.6289). Similarly, no statistically significant differences between groups were found in total WSAVA scores (p=0.8), and WSAVA scores for duodenum (p=0.2), ileum (p=01.0) and colon (p=0.6). There was a strong positive correlation found between WSAVA duodenal and ileal scores and duodenal and ileal endoscopy scores. Moreover, there was a negative correlation seen between endoscopy scores in the duodenum and ileum and albumin and cobalamin serum concentrations. Power calculation revealed that 60 dogs per group would have been needed to detect statistically significant differences between treatment groups.

12. CONCLUSIONS

Standard dietary treatment induced rapid clinical response in all cases. Because the study was underpowered, it was not possible to determine whether or not supplementation with prebiotic and GAG had an additional effect on clinical outcomes or frequency of relapses.

13. REFERENCES

1. Allenspach K et al. Chronic enteropathies in dogs: evaluation of risk factors for negative outcome. J Vet Intern Med. 2007;21:700-8.

2. Dandrieux et al. Evaluation of lymphocyte apoptosis in dogs with IBD. Am J Vet Res. 2008;69:1279-85.

14. APPENDICES

Appendix 1: Individual Animal Data

Appendix 2: Results tables

APPENDIX 1

TABLE A1. Group 1 Signalment and enrolment data

Group	Treatment	Patient No.	Breed	DOB	Approximate Age (Months)	Gender	Body Condition Score	Inclusion confirmed
1	BIOEC2015	RVB01	Cockapoo	13-Jul-14	14	ME	Ideal (4-5)	28-Oct-15
1	BIOEC2015	RVB07	Staffordshire Bull Terrier	01-Jan-05	131	MN	Too Thin (1-3)	09-Dec-15
1	BIOEC2015	RVB08	Border Collie	03-Jul-13	29	ME	Too Thin (1-3)	17-Dec-15
1	BIOEC2015	RVB13	Labrador Retriever	13-Dec-11	53	MN	Too Heavy (6-9)	17-Jun-16
1	BIOEC2015	RVB16	Cocker Spaniel	16-Jul-06	120	FN	Ideal (4-5)	29-Jul-16
1	BIOEC2015	RVB18	Cross Breed	28-Nov-12	44	FN	Ideal (4-5)	11-Aug-16
1	BIOEC2015	RVB27	Bearded Collie	18-Jun-12	56	FE	Too Thin (1-3)	21-Mar-17
1	BIOEC2015	RVB29	Labrador Retriever	04-Aug-09	91	MN	Ideal (4-5)	18-Apr-17
1	BIOEC2015	RVB32	Lurcher	07-Apr-15	25	FN	Ideal (4-5)	31-May-17
1	BIOEC2015	RVB35	Staffordshire Bull Terrier	17-Feb-11	81	FN	Ideal (4-5)	19-Dec-17

ME = male entire, MN = male neutered, FE = female entire, FN = female neutered

TABLE A2. Group 2 Signalment and enrolment data.

Group	Treatment	Patient No.	Breed	DOB	Approximate Age (Months)	Gender	Body Condition Score	Inclusion confirmed
2	Placebo	RVB02	Cross breed	28-Sep-12	36	ME	Ideal (4-5)	30-Oct-15
2	Placebo	RVB05	Staffordshire Bull Terrier	27-Sep-09	73	MN	Ideal (4-5)	26-Nov-15
2	Placebo	RVB09	German Shepherd	30-Apr-09	80	ME	Too Thin (1-3)	10-Feb-16
2	Placebo	RVB11	Boxer	14-Mar-14	22	ME	Too Thin (1-3)	10-Feb-16
2	Placebo	RVB12	German Shepherd	27-Aug-11	54	FE	Ideal (4-5)	18-Mar-16
2	Placebo	RVB20	Border Collie	09-Sep-14	23	ME	Ideal (4-5)	01-Sep-16
2	Placebo	RVB28	Whippet	20-Aug-15	19	FE	Too Thin (1-3)	18-Apr-17
2	Placebo	RVB30	French Bulldog	01-May-15	23	MN	Ideal (4-5)	25-Apr-17
2	Placebo	RVB31	Lhasa Apso	27-May-12	58	MN	Ideal (4-5)	02-May-17
2	Placebo	RVB34	Labradoodle	13-Sep-14	36	MN	Ideal (4-5)	28-Sep-17

ME = male entire, MN = male neutered, FE = female entire, FN = female neutered

TABLE A3. Patient history.

Group	Treatment	Patient No.	Vaccination	Worming	History of Disease	Duration of Disease	Seasonal Influence
1	BIOEC2015	RVB01	distemper virus, hepatitis contaginosa canis, parvovirus, parinfluenza, leptospirosis	Irregular	Mrs Hartridge has owned Harvey since he was 12 weeks old and obtained him from a breeder. Since she has owned him he has had diarrhoea, the first episode was noted on 3rd November 2014. For this episode Canikur Pro Paste and a bland diet were prescribed. On November 26th 2014 a 3 day pooled sample was analysed and found no evidence of parasites or protozoa. On 4th June 2015 Harvey re-presented for chronic diarrhoea and low BCS (2/5), he also had bilaterally full anal glands due to diarrhoea.	1 year	No
1	BIOEC2015	RVB07	distemper virus, hepatitis contaginosa canis, parvovirus, parinfluenza, leptospirosis	Every 2 months	Max developed (chronic) diarrhoea in August 2015. It is described as primarily small intestine in nature and he has lost weight despite a good appetite. D+ large volume and watery.	3.5months	No
1	BIOEC2015	RVB08	distemper virus, hepatitis contaginosa canis, parvovirus, parinfluenza, leptospirosis	Every 3 months	The owner obtained Finn from a breeder at 8 weeks of age, a few weeks later he started having episodes of yellow diarrhoea. He has continued to have these episodes on and off since then, with no particular pattern. The longest period without diarrhoea was 3 months. Initially the diarrhoea was very mucoid and smelly, however it has now changed. Finn squats for a while and then moves around when trying to pass diarrhoea. Although at the start there was some fresh blood, this is no longer a predominant feature. Finn currently has diarrhoea and has been 6 times in the last 8-9 hours. Over the past week Finn has lost 1.5kg.	Since 8 weeks of age	No
1	BIOEC2015	RVB13	distemper virus, hepatitis contaginosa canis,	Every 6 months	Presented with 3 year chronic history of vomiting and diarrhoea. The owners have tried Dillon on several diets, most recently z/d.	3 years	No

			parvovirus, parinfluenza, leptospirosis		This caused him to have black, watery diarrhoea. He is currently on a mix of Chappie and WAGG. The vomiting is normally just partially digested food brought up 4 hours post eating, however, there was an episode in October 2015 where Dillon had fresh blood in both his vomitus and faeces. Dillon is vaccinated and wormed. He also has a mass on his left eyelid which is being treated with steroid cream.		
1	BIOEC2015	RVB16	distemper virus, hepatitis contaginosa canis, parvovirus, parinfluenza, leptospirosis	Every month	Rosie presented with an intermittent 9 month history of inappetance, vomiting and mixed bowel diarrhoea. Often after I/V fluids her appetite returned, however, recently this has proved difficult. Rosie has also suffered significant weight loss over a short period.	Since October 2015	No
1	BIOEC2015	RVB18	distemper virus, hepatitis contaginosa canis, parvovirus, parinfluenza, leptospirosis	Every 6 months	Bella has been having intermittent chronic vomiting since May 2016, after ingestion of soil containing fertiliser. Since this she has had repeated episodes which have improved after i/v fluids and medication. Her most recent episode was 30/31 July 2016.	Since 28 May 16	No
1	BIOEC2015	RVB27	None	Every 4 months	Long term history of chronic vomiting and diarrhoea since 4 years ago.	Intermitten t for 4 years	No
1	BIOEC2015	RVB29	distemper virus, hepatitis contaginosa canis, parvovirus, parinfluenza, leptospirosis	Irregular	8 month history of vomiting several times a week	8 months	No
1	BIOEC2015	RVB32	distemper virus, hepatitis contaginosa canis, parvovirus, parinfluenza, leptospirosis	Irregular	History of 2 episodes of vomiting. The first episode was associated with diarrhoea with mucus but no blood and inappetance (April). Second episode (end April 2017) mainly vomiting and reduced appetite	Documente d since April 2017 but suspect since prior to rehoming	No

1	BIOEC2015	RVB35	None		Chronic vomiting and diarrhoea for 4 weeks. Watery, yellow vomit three to four times a day which has decreased over the past week. Watery diarrhoea with some blood but less frequent over the past week. Showing fewer signs of lethargy over the last seven days.	4 weeks	No
2	Placebo	RVB02	distemper virus, hepatitis contaginosa canis, parvovirus, leptospirosis, kennel cough	Every 3 months	12 month history of intermittent retching and diarrhoea - watery and sometimes bloody. No food trial attempted but signs worsen when scavenging or when fed chicken. No weight loss. Generally good appetite other than when retching - which is generally self-limiting in <12h.	12 months	No
2	Placebo	RVB05	distemper virus, hepatitis contaginosa canis, parvovirus, parinfluenza, leptospirosis, bordatella	Every 3 months	Acute onset yellow watery diarrhoea started in June 2015. No response to treatment with two courses of antimicrobials, or to change to veterinary prescription diet (hills i/d)	4-5 months	No
2	Placebo	RVB09	distemper virus, hepatitis contaginosa canis, parvovirus, leptospirosis	Every 3 months	5 years of chronic diarrhoea. Varying form very watery to sometimes soft paste. Has received various treatments at referring vets with little success. Been tried on many different diets. Thin (chronically). Cobalamin levels were found to be low here on last visit.	4-5 years	No
2	Placebo	RVB11	distemper virus, hepatitis contaginosa canis, parvovirus, parinfluenza, leptospirosis, rabies	Every 3 months	Onset of loose stools June 2014. Ongoing intermittent diarrhoea with occasional vomiting and weight loss. Faecal analysis on 26 Nov 15 positive for Giardia, treated with metronidazole and fenbendazole. Despite initial improvement, the diarrhoea re-presented, this time with faecal analysis for giardia negative. Diarrhoea still ongoing.	Since 13 Jun 14	No
2	Placebo	RVB12	distemper virus, hepatitis contaginosa canis, parvovirus, parinfluenza, leptospirosis,	Every 3 months	1 year intermittent vomiting and large bowel diarrhoea. Inappetance during these episodes; resolved after several days. In between episodes, is normal. No weight loss. Trialled on amox-clavulanate, metronidazole, maripopitant and ranitidine.	1 year	No

2	Placebo	RVB20	distemper virus, hepatitis contaginosa canis, parvovirus, parinfluenza, leptospirosis	Every 1 month	Woody presented with a history of intermittent, mixed bowel diarrhoea. His episodes of diarrhoea have been increasing in frequency and can be up to 3 times per week. There is often blood present in his faeces. Woody appears uncomfortable during each episode, with an arched back and tense stomach. Between episodes, Woody's stool is normal and he generally has a good appetite.	~1 year	No
2	Placebo	RVB28	distemper virus, hepatitis contaginosa canis, parvovirus, parinfluenza,	Irregular	15 month history of mixed bowel diarrhoea	15 months	No
2	Placebo	RVB30	distemper virus, hepatitis contaginosa canis, parvovirus, parinfluenza, leptospirosis	Every 1 month	4 weeks of diarrhoea. Some improvement on antibiotics but still no formed stools. No vomiting, reduced appetite.	4 weeks	No
2	Placebo	RVB31	distemper virus, hepatitis contaginosa canis, parvovirus, parinfluenza, leptospirosis, Kennel Cough	Irregular	Approximately one year history of intermittent vomiting and chronic large bowel diarrhoea. Previous increased spec cPL. Treat for pancreatitis with low fat gastrointestinal diet but ongoing diarrhoea (vomiting improved). Was very overweight (~14kg) and been on a diet but owner feels excessive weight loss. One collapse ~ 3years ago, suspect anaphylactic shock. Second collapse three weeks ago, cause unknown. Chronic history of pruritis.	~1 year	No
2	Placebo	RVB34	distemper virus, hepatitis contaginosa canis, parvovirus, parinfluenza, leptospirosis, bordatella	Every 1 month	Chronic vomiting for 3 months, no diarrhoea, normal appetite, no weight loss.	3 months	No

TABLE A4. Completion and withdrawal.

Group	Treatment	Patient No.	Last scheduled visit (Visit No)	Date of last scheduled visit	Completed study?	Date of withdrawal	Reason for Withdrawal	Comments
1	BIOEC2015	RVB01	7	02-Mar-16	Yes			
1	BIOEC2015	RVB07	2	22-Dec-15	No	04-Feb-16	Animal withdrawn due to worsening of clinical signs	Progressive weight loss and diarrhoea
1	BIOEC2015	RVB08	7	20-Apr-16	Yes			
1	BIOEC2015	RVB13	7	19-Oct-16	Yes			
1	BIOEC2015	RVB16	7	01-Dec-16	Yes			
1	BIOEC2015	RVB18	7	15-Dec-16	Yes			
1	BIOEC2015	RVB27	1	07-Mar-17	No	06-Jun-17	Other	Owner could not administer IVP
1	BIOEC2015	RVB29	7	23-Aug-17	Yes			
1	BIOEC2015	RVB32	7	04-Oct-17	Yes			
1	BIOEC2015	RVB35	7	24-Apr-18	Yes			
2	Placebo	RVB02	7	02-Mar-16	Yes			
2	Placebo	RVB05	1	16-Nov-15	No	21-Dec-15	Animal withdrawn due to worsening of clinical signs	Clinical decision was made to put dog on steroids and cyclosporine
2	Placebo	RVB09	3	19-Apr-16	No	19-Apr-16	Animal withdrawn due to worsening of clinical signs	Recent 4kg weight loss and chronic diarrhoea. Needs to start on immunomodulatory medication

2	Placebo	RVB11	7	16-Jun-16	Yes			
2	Placebo	RVB12	3	25-May-16	No	25-May-16	Animal withdrawn due to worsening of clinical signs	Withdrawn due to worsening signs and the need for immunomodulatory medication
2	Placebo	RVB20	7	06-Jan-17	Yes			
2	Placebo	RVB28	2	04-May-17	No	26-Jun-17	Animal withdrawn due to worsening of clinical signs	Clinical signs worsened, dog required veterinary attention and received antibiotics and prednisolone (26 Jun 17)
2	Placebo	RVB30	2	09-May-17	No	03-Jul-17	Other	On 19 May 17 this dog was prescribed apoquel and cephalexin for an ongoing skin condition
2	Placebo	RVB31	7	01-Sep-17	Yes			
2	Placebo	RVB34	7	01-Feb-18	Yes			

TABLE A5. Biochemistry results.

Group	Treatment	Patient No.	Visit No.	Total protein	Albumin	Globulin	Sodium	Potassium	Chloride	Calcium	Inorganic Phosphorus	Urea	Creatinine	Cholesterol	Total bilirubin	Amylase	Lipase	ALT	CK	ALP (U/l)	Glucose
Units:				g/l	g/l	g/l	mmol/l	mmol/l	mmol/l	mmol/l	mmol/l	mmol/l	mmol/l	mmol/l	mmol/l	U/l	U/l	U/l	U/l	U/l	mmol/l
Reference range:				49-71	28-39	21-41	142-153	3.9-5.5	105-118	2.13-2.7	0.8-2	3-9.1	59-138	3.3-8.9	0-2.4	176-1245	72-1115	13-88	61-394	19-285	3-6
1	BIOEC2015	RVB01	1	60.8	34.8	26.0	149.0	4.20	114.0	2.64	1.82	5.3	81	10.9	1.5	630	149	44	91	75	
1	BIOEC2015	RVB01	3	63.9	36.7	27.2	148.0	4.10	113.0	2.57	1.41	5.7	87	5.9	1.2	622	158	73	100	44	5.5
1	BIOEC2015	RVB01	7	59.1	35.3	23.8	149.0	3.80	115.0	2.54	1.18	5.7	84	7.0	1.2	629	225	49	128	47	
1	BIOEC2015	RVB07	1	40.8	22.7	18.1	148.0	5.40	115.0	2.09	1.22	6.7	67	3.9	0.7	1102	637	73	151	50	

1	BIOEC20 15	RVB08	1	55.4	31.2	24.2	148. 0	4.50	113. 0	2.33	1.26	4.5	93	4.1	1.5	830	117	33	74	36	4.7
1	BIOEC20 15	RVB08	7	56.0	42.0	14.0	147. 0	4.20	115. 0	2.70	1.30	5.5	106	7.6	2.0	576	24	43	71	107	
1	BIOEC20 15	RVB08	3	55.9	31.5	24.4	146. 0	4.10	115. 0	2.37	1.07	6.0	92	7.4	2.5	647	146	57	60	94	
1	BIOEC20 15	RVB13	1	55.9	32.1	23.8	148. 0	4.80	113. 0	2.43	1.63	3.7	90	4.4	1.4	858	466	29	93	40	4.6
1	BIOEC20 15	RVB13	3	58.6	35.2	23.4	140. 0	4.10	101. 0	2.58	1.56	6.1	96	6.2	2.1	712	443	54	120	60	4.7
1	BIOEC20 15	RVB13	7	55.3	31.9	23.4	149. 0	4.20	109. 0	2.42	1.27	4.0	84	6.3	1.3	737	538	59	109	69	5.0
1	BIOEC20 15	RVB16	1	47.6	27.8	19.8	147. 0	4.60	115. 0	2.26	0.87	3.7	66	2.6	1.0	109 0	126	19	57	9	5.0
1	BIOEC20 15	RVB16	3	46.3	26.4	19.9	145. 0	4.70	112. 0	2.33	0.90	4.8	73	2.8	1.7	966	119	27	106	13	
1	BIOEC20 15	RVB16	7	55.6	30.6	25.0	148. 0	4.60	112. 0	2.52	1.20	5.5	82	3.1	1.8	101 5	198	28	67	16	5.1
1	BIOEC20 15	RVB18	1	55.8	32.5	23.3	151. 0	4.30	114. 0	2.54	1.18	11.2	100	3.9	1.7	759	245	30	127	29	5.2
1	BIOEC20 15	RVB18	3	61.7	35.7	26.0	144. 8	4.35	108. 5	2.62	1.29	6.2	83	7.0	0.3	630	231	46	160	57	5.9
1	BIOEC20 15	RVB18	7	61.7	35.7	26.0	148. 0	4.50	109. 0	2.55	1.33	6.4	73	6.5	1.4	659	217	33	72	60	5.6

1	BIOEC2015	RVB27	1	58.1	32.6	25.5	149.0	4.50	115.0	2.44	0.80	7.1	110	6.2	1.6	600	225	25	184	15	
1	BIOEC2015	RVB29	1	53.3	33.0	20.3	149.0	4.60	115.0	2.33	0.89	3.4	91	4.0	1.9	522	437	35	126	40	
1	BIOEC2015	RVB29	3	57.4	34.7	22.7	150.0	4.80	116.0	2.33	0.94	4.0	98	4.6	1.7	611	499	37	116	43	5.2
1	BIOEC2015	RVB29	7	58.0	31.7	26.3	148.9	4.38	118.2	2.35	0.84	4.8	86	4.58	2.6	587.8	508	34.6	111	46	5.1
1	BIOEC2015	RVB32	1	44.1	28.2	15.9	149.0	4.40	115.0	2.22	1.05	5.4	105	3.8	2.1	1368	427	101	77	37	5.5
1	BIOEC2015	RVB32	3	52.5	34.4	18.1	147.0	4.50	109.0	2.46	1.42	4.3	114	5.9	2.8	380	350	36	54	41	5.1
1	BIOEC2015	RVB32	7	55.9	33.7	22.2	147.2	4.15	113.0	2.42	1.26	7.6	116	5.58	3.5	462.3	382	34.2	118	24	5.2
1	BIOEC2015	RVB35	1	47.5	23.2	24.3	148.1	4.58	121.8	2.27	1.09	4.2	76	3.6	2.1	1151	203	44	152	65	5.7
1	BIOEC2015	RVB35	3	60.1	31.4	28.7	145.9	4.17	111.5	2.35	0.69	4.6	89	5.97	2.7	795.8	223	27.3	99	39	4.8
1	BIOEC2015	RVB35	7	59.6	29.4	30.2	148.8	4.33	111.3	2.51	1.18	5.9	79	6.31	3.1	838	303	23.1	170	25	5.2
2	Placebo	RVB02	1	53.0	33.1	19.9	147.0	4.40	112.0	2.41	1.28	3.5	70	5.2	0.8	528	573	25	268	13	5.2
2	Placebo	RVB02	3	51.3	32.5	18.8	146.0	4.40	109.0	2.38	0.97	4.9	78	5.1	0.0	624	359	27	222	40	

2	Placebo	RVB02	7	55.5	35.2	20.3	147.0	4.30	113.0	2.42	1.23	4.5	65	4.2	1.5	498	482	30	167	65	6.8
2	Placebo	RVB05	1	29.0	15.4	13.6	147.0	4.00	117.0	1.82	0.52	5.7	120	2.5	0.7	1591	275	34	228	25	4.8
2	Placebo	RVB09	1	45.8	23.4	22.4	137.0	3.90	101.0	2.26	1.18	5.7	74	5.7	2.9	1472	147	602	170	547	
2	Placebo	RVB09	3	41.0	16.0	25.0	137.0	3.40	110.0	2.30	1.00	7.1	130	7.7	18.0	1104	29	1251	176	1511	
2	Placebo	RVB11	1	58.3	35.1	23.2	147.0	4.60	113.0	2.50	1.90	8.2	110	4.4	1.1	810	252	40	414	39	
2	Placebo	RVB11	3	56.0	32.0	24.0	143.0	4.20	111.0	2.50	1.60	5.1	99	7.0	2.0	869	129	105	162	566	
2	Placebo	RVB11	7	62.9	34.0	28.9	146.0	4.70	113.0	2.48	1.66	5.9	96	6.8	1.0	776	227	47	271	244	
2	Placebo	RVB12	1	54.6	30.0	24.6	147.0	4.90	114.0	2.37	1.67	3.3	85	4.6	1.9	281	297	33	111	21	
2	Placebo	RVB12	3	54.5	31.3	23.2	149.0	5.20	113.0	1.81	1.03	5.3	104	3.6	1.2	663	209	46	164	25	
2	Placebo	RVB20	1	53.7	34.3	19.4	150.0	4.40	116.0	2.57	1.54	4.1	95	4.8	3.1	397	267	29	62	23	4.8
2	Placebo	RVB20	3	57.2	31.8	25.4	149.0	4.60	113.0	2.52	1.53	7.3	82	4.8	1.5	558	202	34	117	52	5.9
2	Placebo	RVB20	7	55.5	33.6	21.9	146.0	4.30	112.0	2.48	1.24	5.4	88	3.7	1.6	544	112	37	84	25	6.6

2	Placebo	RVB28	1	51.3	31.1	20.2	147.0	5.00	112.0	2.26	1.70	4.9	63	4.5	0.6	366	896	37	68	56	
2	Placebo	RVB30	1	58.0	36.9	21.1	146.0	4.70	108.0	2.69	1.30	3.8	102	4.7	4.7	464	112	40	267	55	4.2
2	Placebo	RVB31	1	54.1	33.3	20.8	146.0	4.50	107.0	2.58	1.31	7.0	85	5.6	1.4	589	241	76	73	70	
2	Placebo	RVB31	3	60.3	35.8	24.5	148.0	4.70	108.0	2.53	1.29	7.0	83	10.5	1.7	648	381	57	107	85	5.5
2	Placebo	RVB31	7	62.8	32.7	30.1	147.1	4.78	110.3	2.65	1.11	6.9	73	10.10	2.9	659.9	354	50.4	114	76	5.5
2	Placebo	RVB34	1	56.7	30.6	26.1	145.7	4.34	114.5	2.46	1.16	4.4	80	8.72	4.1	596.2	557	46.0	117	17	5.0
2	Placebo	RVB34	3	57.3	30.5	26.8	144.6	4.11	114.5	2.45	1.20	4.8	84	7.85	5.3	575.4	436	51.3	67	37	4.4
2	Placebo	RVB34	7	61.7	32.8	28.9	145.8	4.12	112.5	2.49	1.07	5.1	75	9.27	3.6	481.4	545	41.1	153	23	5.2

TABLE A6. Biochemistry - laboratory and investigator comments.

Group	Treatment	Patient No.	Visit No.	Lab Comment	Investigator Comment
1	BIOEC2015	RVB01	1	Serum is very slightly haemolysed. This may falsely increase total protein, albumin, globulins, urea, cholesterol and potassium. Sodium, chloride, calcium, creatinine, ALT, amylase, lipase and CK may be decreased. Levels of phosphorus, bilirubin and ALP may be undetectable if haemolysis is severe. Repeat sampling is recommended.	Elevated Cholesterol NCS
1	BIOEC2015	RVB01	3	Serum is very slightly haemolysed. This may falsely increase total protein, albumin, globulins, urea, cholesterol and potassium. Sodium, chloride, calcium, creatinine, ALT, amylase, lipase and CK may be decreased. Levels of phosphorus, bilirubin and ALP may be undetectable if haemolysis is severe. Repeat sampling is recommended.	
1	BIOEC2015	RVB01	7		Potassium reduction not significant
1	BIOEC2015	RVB07	1		Mild reduction of protein levels, mild clinical significance
1	BIOEC2015	RVB08	1		
1	BIOEC2015	RVB08	7		Elevated Alb, Chol, ALT, ALP. Decreased Globulin = NCS. Protein levels should be monitored - sent to external lab
1	BIOEC2015	RVB08	3		Elevated Tbil and low CK NCS
1	BIOEC2015	RVB13	1		
1	BIOEC2015	RVB13	3		

1	BIOEC2015	RVB13	7		
1	BIOEC2015	RVB16	1		Pan hypoproteinaemia due to protein losing enteropathy (PLE) Hypocholesterolaemia suspect due to PLE. Decreased CK and ALP NCS
1	BIOEC2015	RVB16	3		Albumin and globulin low: significant pan hypoproteinaemia but essentially unchanged significantly from previous. Ongoing hypocholesterolaemia. Low ALP NCS.
1	BIOEC2015	RVB16	7	Serum is slightly lipemic. = This may falsely elevate total protein, albumin, globulins, potassium, phosphorus, calcium, bilirubin, ALT and cholesterol. It may falsely decrease sodium, chloride, urea, creatinine, CK, ALP, amylase and lipase. A repeat fasted sample is recommended.	Hypocholesterolaemia could be clinically significant. Can be associated with protein losing enteropathy. However, both proteins are within normal limits now.
1	BIOEC2015	RVB18	1		increased urea clinically significant, could be associated with gastrointestinal haemorrhage or dehydration
1	BIOEC2015	RVB18	3	Serum is slightly haemolysed. = This may falsely increase total protein, albumin, globulins, urea, cholesterol and potassium. Sodium, chloride, calcium, creatinine, ALT, amylase, lipase and CK may be decreased. Levels of phosphorus, bilirubin and ALP may be undetectable if haemolysis is severe. Repeat sampling is recommended.	
1	BIOEC2015	RVB18	7		
1	BIOEC2015	RVB27	1		Decreased ALP NCS
1	BIOEC2015	RVB29	1		Decreased Globulin NCS
1	BIOEC2015	RVB29	3		
1	BIOEC2015	RVB29	7		High chloride and Low calcium NCS

1	BIOEC2015	RVB32	1		Low globulin concentration and total protein considered likely to be associated with gastrointestinal disease. Increased amylase activity may be associated with borderline increase in cPli supporting possible pancreatitis. Mild increase in ALT activity not clinically significant here.
1	BIOEC2015	RVB32	3		Mild hyperglobulinemia likely reflects underlying IBD. Other changes NCS
1	BIOEC2015	RVB32	7		Low globulin NCS
1	BIOEC2015	RVB35	1		Low total protein and albumin consistent with IBD, high chloride NCS
1	BIOEC2015	RVB35	3	Lipaemia Index: None detected<br>Icterus Index: None detected<br>Haemolysis Index: None detected	Low Inorganic Phosphorus NCS
1	BIOEC2015	RVB35	7	Lipaemia Index: None detected<br>Icterus Index: None detected<br>Haemolysis Index: +	High cholesterol NCS
2	Placebo	RVB02	1	Serum is very slightly haemolysed. = This may falsely increase total protein, albumin, globulins, urea, cholesterol and potassium. Sodium, chloride, calcium, creatinine, ALT, amylase, lipase and CK may be decreased. Levels of phosphorus, bilirubin and ALP may be undetectable if haemolysis is severe. Repeat sampling is recommended	Haemolysis only slight and not significant. Globulin not clinically significant. ALP not clinically significant
2	Placebo	RVB02	3	Serum is very slightly haemolysed. = This may falsely increase total protein, albumin, globulins, urea, cholesterol and potassium. Sodium, chloride, calcium, creatinine, ALT, amylase, lipase and CK may be decreased. Levels of phosphorus, bilirubin and ALP may be undetectable if haemolysis is severe. Repeat sampling is recommended	Decreased Globulin NCS
2	Placebo	RVB02	7		Globulin change is not clinically significant

2	Placebo	RVB05	1		Pan hypoproteinaemia consistent with protein losing enteropathy, total hypocalcaemia related to hypoproteinaemia, hypophosphatemia may be related to vitamin D deficiency, hypocholesterolaemia likely related to intestinal malabsorption, increased amylase non-specific change of no clinical significance
2	Placebo	RVB09	1		Decreased Tpro and Albumin due to IBD. Decreased Sodium, Chloride and elevated Tbil, Amylase, ALT and ALP NCS.
2	Placebo	RVB09	3		Concerned about hypoalbuminemia, total bilirubinaemia and increased ALT and ALP. Withdrawn from trial and recommended further investigations. Clarification: Withdrawn from study due to weight loss and persistent diarrhoea (before receiving these results)
2	Placebo	RVB11	1	Serum is very slightly haemolysed. = This may falsely increase total protein, albumin, globulins, urea, cholesterol and potassium. Sodium, chloride, calcium, creatinine, ALT, amylase, lipase and CK may be decreased. Levels of phosphorus, bilirubin and ALP may be undetectable if haemolysis is severe. Repeat sampling is recommended	Elevated CK NCS
2	Placebo	RVB11	3		Raised ALP and ALT NCS
2	Placebo	RVB11	7		
2	Placebo	RVB12	1		
2	Placebo	RVB12	3		Hypocalcaemia often associated with GI disease
2	Placebo	RVB20	1		Decreased Globulin. Raised Total bilirubin. Not clinically significant

2	Placebo	RVB20	3		
2	Placebo	RVB20	7		Glucose not of clinical significance
2	Placebo	RVB28	1		Decreased Globulin NCS
2	Placebo	RVB30	1		High Total bilirubin NCS
2	Placebo	RVB31	1		Mild hyperglobulinemia could be associated with protein losing enteropathy, but no other supportive changes
2	Placebo	RVB31	3		High cholesterol NCS
2	Placebo	RVB31	7		High cholesterol NCS
2	Placebo	RVB34	1		Increased cholesterol Not clinically significant
2	Placebo	RVB34	3		Increased cholesterol and total bilirubin NCS
2	Placebo	RVB34	7	Lipaemia Index: None detected. Icterus Index: None detected Haemolysis Index: +	High Cholesterol NCS

TABLE A7. Albumin results for both treatment groups.

**Parameter:**			**Albumin**			
**Units:**			g/l			
**Reference range:**			28-39			
**Group**	**Treatment**	**Patient No.**	**Visit 1**	**Visit 2**	**Visit 3**	**Visit 7**
1	BIOEC2015	RVB01	34.8	34.4	36.7	35.3
1	BIOEC2015	RVB07	22.7	18.6		
1	BIOEC2015	RVB08	31.2	30.1	31.5	42.0
1	BIOEC2015	RVB13	32.1	33.6	35.2	31.9
1	BIOEC2015	RVB16	27.8	25.6	26.4	30.6
1	BIOEC2015	RVB18	32.5	36.3	35.7	35.7
1	BIOEC2015	RVB27	32.6			
1	BIOEC2015	RVB29	33.0	32.5	34.7	31.7
1	BIOEC2015	RVB32	28.2	33.4	34.4	33.7
1	BIOEC2015	RVB35	23.2	27.8	31.4	29.4
2	Placebo	RVB02	33.1	33.9	32.5	35.2
2	Placebo	RVB05	15.4			
2	Placebo	RVB09	23.4	18.4	16.0	
2	Placebo	RVB11	35.1	33.2	32.0	34.0
2	Placebo	RVB12	30.0	36.0	31.3	

2	Placebo	RVB20	34.3	30.2	31.8	33.6
2	Placebo	RVB28	31.1	33.4		
2	Placebo	RVB30	36.9	32.5		
2	Placebo	RVB31	33.3	35.1	35.8	32.7
2	Placebo	RVB34	30.6	30.2	30.5	32.8

TABLE A8. Folate results for both treatment groups.

**Parameter:**			**Folate**		
**Units:**			ng/ml		
**Reference range:**			6.5-11.5		
**Group**	**Treatment**	**Patient No.**	**Visit 1**	**Visit 3**	**Visit 7**
1	BIOEC2015	RVB01	15.0	18.3	16.9
1	BIOEC2015	RVB07	11.8		
1	BIOEC2015	RVB08	7.3	19.0	16.4
1	BIOEC2015	RVB13	17.2	17.2	18.1
1	BIOEC2015	RVB16	5.9	>24.00	16.4
1	BIOEC2015	RVB18	>24.00	>24.00	9.2
1	BIOEC2015	RVB27	5.2		
1	BIOEC2015	RVB29	14.3	18.1	16.4
1	BIOEC2015	RVB32	5.4	13.6	14.2
1	BIOEC2015	RVB35	6.1	16.1	9.5
2	Placebo	RVB02	9.7	14.3	13.7
2	Placebo	RVB05	9.2		
2	Placebo	RVB09	9.4	18.5	
2	Placebo	RVB11	8.9	14.2	17.2

2	Placebo	RVB12	13.9		
2	Placebo	RVB20	7.8	>24.00	4.3
2	Placebo	RVB28	6.4		
2	Placebo	RVB30	11.4		
2	Placebo	RVB31	10.8	15.2	12.5
2	Placebo	RVB34	11.0	15.0	8.1

TABLE A9. Cobalamin results for both treatment groups.

**Parameter:**			**Cobalamin**		
**Units:**			ng/ml		
**Reference range:**			>200		
**Group**	**Treatment**	**Patient No.**	**Visit 1**	**Visit 3**	**Visit 7**
1	BIOEC2015	RVB01	709.0	487.0	425.0
1	BIOEC2015	RVB07	153.0		
1	BIOEC2015	RVB08	461.0	429.0	442.0
1	BIOEC2015	RVB13	237.0	265.0	171.0
1	BIOEC2015	RVB16	226.0	636.0	653.0
1	BIOEC2015	RVB18	257.0	1083.0	924.0
1	BIOEC2015	RVB27	177.0		
1	BIOEC2015	RVB29	595.0	583.0	561.0
1	BIOEC2015	RVB32	268.0	530.0	458.0
1	BIOEC2015	RVB35	623.0	290.0	406.0
2	Placebo	RVB02	462.0	529.0	420.0
2	Placebo	RVB05	<150.00		
2	Placebo	RVB09	<150.00	>1200	
2	Placebo	RVB11	361.0	433.0	454.0

2	Placebo	RVB12	203.0	1001.0	
2	Placebo	RVB20	504.0	909.0	675.0
2	Placebo	RVB28	585.0		
2	Placebo	RVB30	1052.0		
2	Placebo	RVB31	43.0	1062.0	803.0
2	Placebo	RVB34	>1200	562.0	418.0

TABLE A10. Screening blood tests.

Parameter:				cPLI	TLI	Cortisol	Cortisol - post ACTH
Units:				ug/l	ug/l	nmol/l	nmol/l
Reference range:				0-200	6-35	<250	<500
Group	Treatment	Patient No.	Visit No.				
1	BIOEC2015	RVB01	1	45.0	11.1	126.0	461.0
1	BIOEC2015	RVB07	1	61.0	42.0	71.2	61.7
1	BIOEC2015	RVB08	1	40.0	23.6	48.9	244.0
1	BIOEC2015	RVB13	1	72.0	10.0	51.5	322.0
1	BIOEC2015	RVB16	1	111.0	10.7	73.8	498.0
1	BIOEC2015	RVB18	1	68.0	14.6	25.5	433.0
1	BIOEC2015	RVB27	1	43.0	29.4	166.0	245.0
1	BIOEC2015	RVB29	1	39.0	25.4	41.1	339.0
1	BIOEC2015	RVB32	1	316.0	>50.0	30.3	345.0
1	BIOEC2015	RVB35	1	93.0	35.3	138.0	345.0
2	Placebo	RVB02	1	48.0	36.4	100.0	440.0
2	Placebo	RVB05	1	296.0	25.7	81.8	403.0
2	Placebo	RVB09	1	74.0	15.6	47.5	350.0
2	Placebo	RVB11	1	76.0	30.1	34.4	239.0
2	Placebo	RVB12	1	41.0	5.8	32.1	279.0

2	Placebo	RVB20	1	62.0	15.7	91.8	282.0
2	Placebo	RVB28	1	54.0	25.4	122.0	400.0
2	Placebo	RVB30	1	30.0	29.9	129.0	317.0
2	Placebo	RVB31	1	89.0	30.4	207.0	400.0
2	Placebo	RVB34	1	142.0	>50.0	37.8	320.0

TABLE A11. Haematology results for treatment group 1.

Parameter:				WBC	Neutrophils	Lymphocytes	Monocytes	Eosinophils	Basophils	RBC	Hgb	HCT	MCV	MCH	MCHC	RDW	Plt	PCV
Units:				10e9/l	10e9/l	10e9/l	10e9/l	10e9/l	10e9/l	10e12/ l	g/dL	%	fL	pg	g/dl	%	10e9/l	%
Reference ranges:				6-17.1	3-11.5	1-4.8	0.15- 1.5	0-1.3	0-0	5.5-8.5	12-18	37-55	60-77	19.5- 24.5	32-36	N/A	150- 900	N/A
Group	Treatment	Patient No.	Visit No.															
1	BIOEC20 15	RVB0 1	1	14.39	11.22	2.30	0.43	0.43	0.00	8.08	19.20	56.30	69.70	23.70	34.00	12.80	186.0 0	54
1	BIOEC20 15	RVB0 1	3	13.21	11.23	1.59	0.40	0.00	0.00	8.03	19.20	55.70	69.30	24.00	34.60	13.20	136.0 0	56
1	BIOEC20 15	RVB0 1	7	10.85	8.14	2.17	0.33	0.22	0.00	7.68	18.40	54.50	71.00	24.00	33.80	13.50	215.0 0	53
1	BIOEC20 15	RVB0 7	1	6.95	5.77	0.70	0.28	0.21	0.00	6.08	13.90	41.70	68.50	22.80	33.30	14.80	923.0 0	40
1	BIOEC20 15	RVB0 8	1	14.10	9.87	2.40	0.42	1.41	0.00	6.98	16.90	49.50	70.90	24.20	34.20	12.30	344.0 0	51

1	BIOEC20 15	RVB0 8	7	10.67	4.16	5.23	0.53	0.75	0.00	6.77	16.10	48.10	71.10	23.80	33.50	13.60	261.0 0	47
1	BIOEC20 15	RVB0 8	3	10.53	4.95	4.21	0.74	0.63	0.00	6.70	16.20	47.20	70.40	24.30	34.50	13.40	283.0 0	46
1	BIOEC20 15	RVB1 3	1	6.71	3.89	2.15	0.40	0.27	0.00	5.85	13.70	39.00	66.60	23.40	35.20	12.80	290.0 0	40
1	BIOEC20 15	RVB1 3	3	7.86	4.32	2.99	0.47	0.08	0.00	6.20	14.40	41.50	66.90	23.20	34.70	12.40	242.0 0	43
1	BIOEC20 15	RVB1 3	7	8.81	6.17	1.59	0.97	0.09	0.00	6.08	14.30	41.00	67.30	23.40	34.80	11.90	251.0 0	42
1	BIOEC20 15	RVB1 6	1	8.04	5.87	1.69	0.16	0.32	0.00	5.41	12.50	36.40	67.30	23.10	34.30	13.00	333.0 0	37
1	BIOEC20 15	RVB1 6	3	8.68	5.73	2.34	0.26	0.35	0.00	6.03	14.40	41.00	67.90	23.80	35.10	13.10	411.0 0	41
1	BIOEC20 15	RVB1 6	7	12.95	7.77	2.98	0.65	1.30	0.26	6.40	15.10	43.40	67.80	23.60	34.90	12.40	404.0 0	44
1	BIOEC20 15	RVB1 8	1	11.44	7.55	1.72	0.69	1.37	0.11	7.47	16.10	46.60	62.40	21.50	34.50	12.30	343.0 0	48
1	BIOEC20 15	RVB1 8	3	7.41	3.78	2.89	0.37	0.37	0.00	7.88	16.90	50.30	63.80	21.40	33.60	12.20	115.0 0	50
1	BIOEC20 15	RVB1 8	7	8.17	4.74	2.37	0.41	0.65	0.00	7.44	16.40	47.60	63.90	22.10	34.50	12.20	302.0 0	48

1	BIOEC2015	RVB27	1	7.72	5.40	0.85	0.46	1.00	0.00	7.94	18.00	52.10	65.70	22.70	34.60	11.80	324.00	53
1	BIOEC2015	RVB29	1	5.08	3.25	1.17	0.30	0.36	0.00	6.23	14.70	42.40	68.00	23.60	34.70	12.00	175.00	43
1	BIOEC2015	RVB29	3	4.52	3.39	0.50	0.23	0.41	0.00	6.27	15.00	42.70	68.20	23.90	35.10	12.10	176.00	47
1	BIOEC2015	RVB29	7	4.91	3.39	0.93	0.29	0.29	0.00	6.72	15.80	47.80	71.10	23.50	33.10	11.70	171.00	47
1	BIOEC2015	RVB32	1	8.41	5.63	1.26	0.50	0.93	0.08	6.61	16.20	45.10	68.20	24.50	35.90	11.70	224.00	46
1	BIOEC2015	RVB32	3	4.34	2.17	1.87	0.22	0.09	0.00	6.62	16.50	48.40	73.00	25.00	34.20	11.70	156.00	46
1	BIOEC2015	RVB32	7	5.72	3.03	1.94	0.34	0.40	0.00	6.54	16.20	48.40	73.90	24.80	33.50	11.20	169.00	48
1	BIOEC2015	RVB35	1	10.96	9.32	0.88	0.66	0.11	0.00	6.63	17.10	50.50	76.20	25.90	33.90	13.90	294.00	52
1	BIOEC2015	RVB35	3	6.00	4.38	1.32	0.18	0.12	0.00	7.65	18.60	56.80	74.20	24.30	32.80	12.70	304.00	55
1	BIOEC2015	RVB35	7	6.02	4.64	0.96	0.30	0.12	0.00	7.41	18.10	55.50	74.80	24.50	32.70	12.70	354.00	55

TABLE A12. Laboratory and investigator comments on haematology results group 1.

Group	Treatment	Patient No.	Visit No.	Lab Comment	Investigator Comment
1	BIOEC2015	RVB01	1	PLT: Few small clumps seen RBC: 1+ polychromasia, 3+ echinocytosis WBC: Unremarkable morphology	Elevated HGB and HCT: NCS.
1	BIOEC2015	RVB01	3	PLT: Abundant small and large clumps seen at feathered edge, thus measured conc. Is INVALID. Manual minimum estimate is >200 x 10e9/L RBC: Unremarkable WBC: Unremarkable	Elevated HGB and HCT: NCS.<br>Low PLT: Invalid
1	BIOEC2015	RVB01	7	PLT: Low number of small clumps seen at feathered edge, thus true conc. is likely higher than measured. RBC: Unremarkable.<br>WBC: Reactive lymphocytes present.	Slight increase in haemoglobin concentration is not clinically significant
1	BIOEC2015	RVB07	1	PLT: Clumping in the feathered edge, thus measured concentration should be considered minimum value only. RBC:2+ eccentrocytosis. WBC: Unremarkable. Interpretation: Eccentrocytosis indicates oxidative injury to the erythrocyte membranes. Mild lymphopenia is likely stress/glucocorticoid associated.	Reduced lymphocytes NCS
1	BIOEC2015	RVB08	1	PLT: No clumps seen. RBC: occ. eccentrocytosis, 3+ echinocytosis. Occasional rouleaux. WBC: Reactive lymphocytes present, often with cleaved nucleus, occasionally nucleus appears convoluted on one side. Occasional hyper segmented neutrophils. The low numbers of eccentrocytes present may suggest a degree of oxidative damage. Neutrophil hyper segmentation may suggest a corticosteroid response. Mild eosinophilia, may reflect a hypersensitivity response, eg to parasite burden or allergen, less commonly noted as a paraneoplastic response	Eosinophils increased - likely due to hypersensitivity e.g. to parasites.
1	BIOEC2015	RVB08	7	PLT: No clumps seen. RBC: Unremarkable morphology. WBC: Unremarkable morphology	Raised Lymphocytes NCS
1	BIOEC2015	RVB08	3	PLT: Occ small clumps seen, thus true concentration is likely higher than measured RBC: 3+ echinocytosis WBC: Unremarkable morphology	

1	BIOEC2015	RVB13	1	PLT: Low number of small clumps seen, thus true concentration is likely higher than measured. RBC: 1+ codocytosis. WBC: Reactive lymphocytes present.	
1	BIOEC2015	RVB13	3	PLT: Low number of small clumps seen at feathered edge, thus true conc. is likely higher than measured. RBC: 1+ codocytosis.<br>WBC: Unremarkable	
1	BIOEC2015	RVB13	7	PLT: Low number of small clumps seen at feathered edge, thus true conc. is likely higher than measured. RBC: Occ. codocytosis, occ. keratocytosis. WBC: Unremarkable	
1	BIOEC2015	RVB16	1	PLT: No clumps seen. RBC: 1+ codocytosis. WBC: Unremarkable morphology	Low RBC and HCT mild non-regenerative anaemia, suspect secondary to chronic inflammatory condition
1	BIOEC2015	RVB16	3	PLT: No clumps seen. Occ. macro platelets noted. RBC: Unremarkable. WBC: Reactive lymphocytes present.	
1	BIOEC2015	RVB16	7	PLT: No clumps seen. RBC: 1+ codocytosis, smudged cells present (lipaemia). WBC: Reactive lymphocytes present.	Basophils NCS
1	BIOEC2015	RVB18	1	PLT: Low number of small clumps seen at feathered edge, thus true conc. is likely higher than measured. RBC: Unremarkable.<br>WBC: Reactive lymphocytes present	Eosinophilia clinically significant, could be associated with parasites or hypersensitivity
1	BIOEC2015	RVB18	3	PLT: Moderate number of large clumps seen, thus measured conc. is INVALID. Manual estimate: > 210 x10e9/L. RBC: Unremarkable. WBC: Reactive lymphocytes present	
1	BIOEC2015	RVB18	7	PLT: No clumps seen. RBC: 1+ polychromasia, WBC: Reactive lymphocytes present.	
1	BIOEC2015	RVB27	1	PLT: No clumps seen. RBC: Unremarkable. WBC: Reactive lymphocytes present	Decreased Lymphocytes NCS
1	BIOEC2015	RVB29	1	PLT: Occ. small clumps seen, thus true conc. is higher than measured. Macro platelets noted. RBC: Unremarkable.<br>WBC: Reactive lymphocytes present	Decreased WBC NCS

1	BIOEC2015	RVB29	3	PLT: No clumps seen. RBC: Unremarkable. WBC: Reactive lymphocytes present	Low WBC's and lymphocytes NCS
1	BIOEC2015	RVB29	7	PLT: Low number of small clumps seen, thus true conc. is higher than measured. RBC: Smudged cells present. WBC: Reactive lymphocytes present	Low WBC and lymphocytes not clinically significant
1	BIOEC2015	RVB32	1	PLT: Low number of small clumps seen, thus true conc. is higher than measured. RBC: Unremarkable. WBC: Reactive lymphocytes present	Raised Basophils - Not Clinically significant
1	BIOEC2015	RVB32	3	PLT: Occ. small clumps seen, thus true conc. is higher than measured. RBC: Unremarkable morphology. WBC: Low numbers of neutrophils display subtle cytoplasmic basophilia.<br>Neutropenia with possible slight toxicity raises concerns for overwhelming inflammation, but drug-induced neutropenia may also be considered, and breed-variation may also contribute	Decreased WBC; decreased neutrophils and raised MCH of no apparent clinical significance as clinically well dog
1	BIOEC2015	RVB32	7	PLT: No clumps seen. RBC: Unremarkable morphology<br>WBC: Reactive lymphocytes present	Low WBC, high MCH NCS
1	BIOEC2015	RVB35	1	PLT: Moderate number of large clumps seen, thus true conc. is higher than measured. RBC: Unremarkable. WBC: Reactive lymphocytes present	Low lymphocytes and high MCH NCS
1	BIOEC2015	RVB35	3	PLT: No clumps seen. RBC: Unremarkable morphology. WBC: Reactive lymphocytes present.	High HGB and HCT NCS
1	BIOEC2015	RVB35	7	PLT: Low number of large clumps seen at feathered edge, thus true conc. is likely higher than measured. RBC: Unremarkable morphology. WBC: Unremarkable.	Low lymphocytes. High HGB and HCT NCS

TABLE A13. Haematology results for treatment group 2.

Parameter:				WBC	Neutrophils	Lymphocytes	Monocytes	Eosinophils	Basophils	RBC	Hgb	HCT	MCV	MCH	MCHC	RDW	Plt	PCV
**Units:**				10e9/l	10e9/l	10e9/l	10e9/l	10e9/l	10e9/l	10e12/l	g/dL	%	fL	pg	g/dl	%	10e9/l	%
**Reference ranges:**				6-17.1	3-11.5	1-4.8	0.15-1.5	0-1.3	0-0	5.5-8.5	12-18	37-55	60-77	19.5-24.5	32-36	N/A	150-900	N/A
**Group**	**Treatment**	**Patient No.**	**Visit No.**															
2	Placebo	RVB02	1	11.42	7.88	2.63	0.69	0.23	0.00	6.65	15.40	46.00	69.20	23.20	33.50	12.00	255.00	44
2	Placebo	RVB02	3	8.14	4.40	2.69	0.49	0.57	0.00	6.97	16.30	50.60	72.60	23.40	32.20	11.50	220.00	49
2	Placebo	RVB02	7	9.25	5.46	2.96	0.09	0.74	0.00	6.72	16.10	47.80	71.10	23.90	33.60	12.80	253.00	46
2	Placebo	RVB05	1	11.68	10.63	0.35	0.47	0.23	0.00	6.13	15.50	45.00	73.30	25.20	34.40	13.20	451.00	45
2	Placebo	RVB09	1	9.37	6.93	1.22	0.56	0.66	0.00	7.64	17.00	51.20	66.90	22.20	33.20	11.80	323.00	52
2	Placebo	RVB09	3	8.37	6.70	0.92	0.67	0.08	0.00	7.76	17.00	51.40	66.30	22.00	33.10	12.80	319.00	52

2	Placebo	RVB11	1	11.25	7.99	2.03	0.68	0.56	0.00	7.41	16.80	49.50	66.80	22.70	34.00	14.40	290.00	49
2	Placebo	RVB11	3	8.84	5.75	2.30	0.53	0.27	0.00	6.15	13.80	42.00	68.40	22.40	32.70	13.90	410.00	41
2	Placebo	RVB11	7	9.60	7.39	1.54	0.19	0.48	0.00	6.95	16.10	46.50	66.90	23.10	34.60	14.00	395.00	48
2	Placebo	RVB12	1	8.64	5.79	1.56	0.52	0.78	0.00	5.57	13.80	39.70	71.20	24.70	34.70	14.40	275.00	39
2	Placebo	RVB12	3	7.85	6.12	1.02	0.39	0.24	0.08	7.78	18.50	55.30	71.20	23.80	33.40	13.80	205.00	55
2	Placebo	RVB20	1	6.15	3.75	1.78	0.31	0.31	0.00	6.03	15.00	42.00	69.70	24.90	35.60	11.90	202.00	42
2	Placebo	RVB20	3	8.95	4.56	2.86	0.72	0.81	0.00	6.17	15.40	44.50	72.00	25.00	34.70	12.10	215.00	45
2	Placebo	RVB20	7	7.63	4.50	1.98	0.38	0.76	0.00	6.70	15.80	46.80	69.70	23.60	33.90	11.90	197.00	45
2	Placebo	RVB28	1	6.37	4.71	1.08	0.25	0.32	0.00	6.19	15.60	44.50	71.80	25.10	35.00	12.10	268.00	47
2	Placebo	RVB30	1	6.98	4.33	1.88	0.21	0.56	0.00	7.16	18.00	50.90	71.10	25.10	35.30	11.90	278.00	53
2	Placebo	RVB31	1	12.09	7.13	3.99	0.24	0.73	0.00	6.48	13.70	39.00	60.20	21.10	35.10	12.60	311.00	41
2	Placebo	RVB31	3	14.57	9.03	4.81	0.44	0.29	0.00	7.27	15.40	43.50	59.90	21.20	35.40	12.90	294.00	46

2	Place bo	RVB31	7	21.91	10.08	9.86	0.88	1.10	0.00	7.19	15.80	47.00	65.30	22.00	33.70	11.90	217.0 0	46
2	Place bo	RVB34	1	10.67	5.12	4.05	0.75	0.75	0.00	6.86	16.20	48.60	70.80	23.60	33.40	11.50	238.0 0	49
2	Place bo	RVB34	3	9.11	5.10	2.10	0.73	1.18	0.00	6.77	15.40	48.30	71.30	22.80	31.90	11.90	260.0 0	46
2	Place bo	RVB34	7	8.56	5.74	1.97	0.09	0.77	0.00	7.63	17.80	53.80	70.50	23.30	33.10	11.60	184.0 0	51

TABLE A14. Laboratory and investigator comments on haematology results group 2.

Group	Treatment	Patient No.	Visit No.	Lab Comment	Investigator Comment
2	Placebo	RVB02	1	PLT: Occ small clumps seen, concentration will be higher RBC: 1+ codocytosis WBC: Reactive lymphocytes seen	
2	Placebo	RVB02	3	PLT: Numerous small clumps. RBC: Unremarkable. WBC: Reactive lymphocytes present.	mild haemolysis NCS
2	Placebo	RVB02	7	PLT: No clumps seen. RBC: 1+ codocytosis WBC: Reactive lymphocytes present.	Monocytopenia not clinically significant
2	Placebo	RVB05	1	PLT: Occ small clumps seen RBC: 3+ echinocytosis WBC: Unremarkable morphology	Low lymphocytes consistent with stress or excitement at time of sampling, high MCH not clinically significant
2	Placebo	RVB09	1	PLT: No clumps seen. Macro platelets present. RBC: Unremarkable. WBC: Unremarkable	
2	Placebo	RVB09	3	PLT: No clumps seen RBC: 3+ echinocytosis WBC: Unremarkable morphology	NCS
2	Placebo	RVB11	1	PLT: Numerous small clumps seen at feathered edge, thus true conc. is likely higher than measured. RBC: 1+ anisocytosis. WBC: Reactive lymphocytes present	
2	Placebo	RVB11	3	PLT: Occ small clumps seen, thus true concentration is likely higher than measured RBC: 2+ acanthocytosis, 3+ echinocytosis WBC: Unremarkable morphology Interpretation: Moderate acanthocytosis reflects shear injury/microangiopathic disease; not previously reported in this patient. Consider checking for possible cardiac disease, splenic haemangiosarcoma or localised vasculitis if the cause is not clinically apparent.	
2	Placebo	RVB11	7	PLT: Low number of small clumps seen, thus true concentration is likely higher than measured. RBC: Unremarkable. WBC: Unremarkable	
2	Placebo	RVB12	1	PLT: Low number of small clumps seen, thus true concentration is likely higher than measured. RBC: 1+ anisocytosis, 1+ macrocytosis Occ. polychromasia, 1+ eccentrocytosis rare keratocytes WBC: Reactive lymphocytes present. Pyknotic cells are noted. Given that blood was obtained and run within a short period (according to submission form) the presence of pyknotic cells is of concern over a more rapid turnover of leukocytes, which may be seen with inflammatory disorders, or occasionally as a response to some drugs. The presence of eccentrocytes indicates oxidative damage. Keratocytes are more likely to reflect microangiopathic damage however	NCS

2	Placebo	RVB12	3	PLT: Low number of small clumps seen, thus true concentration is likely higher than measured. RBC: 1+ anisocytosis, 1+ macrocytosis, 1+ polychromasia, occ. eccentrocytosis. WBC: Reactive lymphocytes present	NCS
2	Placebo	RVB20	1	PLT: No clumps seen. RBC: Occ. acanthocytosis. WBC: Reactive lymphocytes present, consistent with antigenic stimulation	Raised MCH - Not clinically significant
2	Placebo	RVB20	3	PLT: Low number of small clumps seen at feathered edge, thus true conc. is likely higher than measured. RBC: Smudged cells present (Lipaemia). WBC: Reactive lymphocytes present	Increased MCH NCS
2	Placebo	RVB20	7	PLT: No clumps seen. RBC: Unremarkable. WBC: Unremarkable.	
2	Placebo	RVB28	1	PLT: Low number of small clumps seen, thus true conc. is higher than measured. RBC: Unremarkable morphology. WBC: Reactive lymphocytes present.	High MCH NCS
2	Placebo	RVB30	1	PLT: No clumps seen. RBC: Unremarkable. WBC: Reactive lymphocytes present	High MCH NCS
2	Placebo	RVB31	1	PLT: Unremarkable. RBC: 1+ Anisocytosis, 1+ Codocytosis, rare polychromasia. WBC: Reactive lymphocytes present.	rare polychromasia NCS
2	Placebo	RVB31	3	PLT: Occ. small clumps seen, thus true conc. is higher than measured. Macro platelets noted. RBC: 1+ anisocytosis, 1+ macrocytosis, 2+ microcytosis, 1+ polychromasia. WBC: Reactive lymphocytes present. PLT: Occasional small clumps present, thus true cons. is likely higher than measured. Occasional macro platelets seen. RBC: 2+ anisocytosis, 2+ macrocytosis, 1+ microcytosis, occ-1+ polychromasia; nRBCs were mostly metarubricytes. WBC: Moderate numbers of lymphocytes have increased amount of pale blue cytoplasm and rare lymphocytes have dark blue cytoplasm. Lymphocytosis is likely reactive. Slight regenerative picture without anaemia could be due to splenic contraction, recovery from previous anaemia, or possibly compensated blood loss. Low MCV is likely to be individual variability (given previous CBC result of similar value).	High Lymphocytes, low MCV NCS
2	Placebo	RVB31	7	PLT: No clumps seen. RBC: 1+ polychromasia. WBC: Lymphocytes are intermediate size with irregularly round nuclei, stippled to clumped chromatin and moderate pale basophilic cytoplasm. Interpretation: Mild lymphocytosis may be reactive (due to chronic inflammation/antigenic stimulation) however a lymphoproliferative disease process (stage V lymphoma vs CLL) is also possible; further investigation (e.g. PARR on a buffy coat/imaging/aspiration of enlarged lymphoid organs) vs monitoring may be considered, depending on the overall clinical picture.	High WBC and lymphocytes not clinically relevant
2	Placebo	RVB34	1	PLT: Low number of small clumps seen at feathered edge, thus true conc. is likely higher than measured. RBC: Unremarkable. WBC: Reactive lymphocytes present.	
2	Placebo	RVB34	3	PLT: No clumps seen. RBC: Unremarkable morphology. WBC: Reactive lymphocytes present	decreased MCHC NCS
2	Placebo	RVB34	7	PLT: Low number of small clumps seen at feathered edge, thus true conc. is likely higher than measured. RBC: Unremarkable. WBC: Reactive lymphocytes present.	Low monocytes NCS

TABLE A15. Urinalysis Group 1.

Group	Treatment	Patient No.	Visit No.	Urine Collection Method	Urine Specific Gravity	Urine-pH	Urine Dip Stick *	Urine Sediment	Investigator Comment
1	BIOEC2015	RVB01	1	Free Catch	>1.050	6	Bilirubin 2+	Urine colour: dull yellow coloured and cloudy. RBC = 0-10 per HPF (x400) WBC = 0-1 per HPF (x400). Fat droplets, very occasional epithelial cell.	Bilirubin 2+ NCS
1	BIOEC2015	RVB01	3	Free Catch	1.050	6	Protein 1+, Bilirubin 1+	Urine colour: amber coloured and turbid. RBC = 0-10 per HPF (x400) WBC = 0-1 per HPF (x400). Some stained/organic debris. Few fat droplets. Spermatozoa.	
1	BIOEC2015	RVB01	7	Unable to obtain					
1	BIOEC2015	RVB07	1	Free Catch	1.014	7.5	Bilirubin 1+	urine colour : yellow coloured and slightly cloudy = Rbc = 0 - 10 per HPF (x400)<br>Wbc = 0 - 1 per HPF (x400)<br>some stained/organic debris<br>few fat droplets<br>occasional epithelial cell	
1	BIOEC2015	RVB08	1	Free Catch	1.025	6	Bilirubin 1+	urine colour : yellow coloured and clear<br>Rbc = 0 - 10 per HPF (x400)<br>Wbc = 5 - 20 per HPF (x400)<br>occasional bilirubin crystals<br>occasional spermatozoa<br>occasional squamous epithelial cells	Bilirubin 1+ NCS
1	BIOEC2015	RVB08	7	Free Catch	1.045	5.5	Negative	urine colour : amber and clear =<br>Rbc = 0 - 10 per HPF (x400)<br>Wbc = 0 - 1 per HPF (x400)<br>spermatozoa	

1	BIOEC2015	RVB08	3	Free Catch	1.034	6	Bilirubin 1+	urine colour : yellow coloured and cloudy Rbc = 0 - 10 per HPF (x400) Wbc = 1 - 5 per HPF (x400) some pigmented debris spermatozoa	Bilirubin 1+ NCS
1	BIOEC2015	RVB13	1	Free Catch	1.036	6.5	Bilirubin 1+	urine colour : dark yellow coloured and clear = Rbc = 0 - 10 per HPF (x400) Wbc = 0 - 1 per HPF (x400) debris few fat droplets occasional squamous epithelial cell	Bilirubin 1+ NCS
1	BIOEC2015	RVB13	3	Free Catch	1.035	6	Bilirubin 2+	urine colour : yellow coloured and clear Rbc = 0 - 10 per HPF (x400) Wbc = 0 - 1 per HPF (x400) no significant deposit	Not clinically significant
1	BIOEC2015	RVB13	7	Free Catch	1.045	5.5	Protein trace Bilirubin trace	Urine colour: Orange/ yellow coloured and clear. = Rbc = 0 - 10 per HPF (x400) Wbc = 0 - 1 per HPF (x400) Some stained/organic debris Occasional squamous epithelial cell.	NCS
1	BIOEC2015	RVB16	1	Expressed bladder under GA	1.010	7	Negative	Urine colour: Straw coloured and slightly cloudy. = Rbc = 0 - 10 per HPF (x400) Wbc = 0 - 1 per HPF (x400) Stained, granular aggregates, size and shape suggestive of granular cast fragments.	SG result NCS, on intravenous fluids
1	BIOEC2015	RVB16	3	Free Catch	1.032	6.5	Blood 4+	urine colour : yellow coloured and cloudy Rbc = 100 - 200 per HPF (x400) Wbc = 1 - 5 per HPF (x400) fat droplets epithelial cells	Blood - may be significant but from manual expression
1	BIOEC2015	RVB16	7	Not collected					
1	BIOEC2015	RVB18	1	Expressed bladder manually whilst under anaesthetic	1.016	7.5	Bilirubin 1+	urine colour : pale yellow coloured and cloudy = Rbc = 0 - 10 per HPF (x400) Wbc = 0 - 2 per HPF (x400) some pigmented debris amorphous clear crystals (most commonly phosphates or calcium oxalates) epithelial cell few fat droplets	Bilirubin 1+ NCS SG 1.016 - on intravenous fluids

1	BIOEC2015	RVB18	3	Free catch for U/A, Manually expressed bladder under GA for storage	1.031	5	Negative	urine colour : yellow coloured and slightly cloudy = Rbc = 0 - 10 per HPF (x400) Wbc = 0 - 2 per HPF (x400) some stained debris few fat droplets 1 x hyaline casts with fatty inclusions seen in 81 HPF (x400) epithelial cells	Not clinically significant
1	BIOEC2015	RVB18	7	None collected					
1	BIOEC2015	RVB27	1	Unable to collect					
1	BIOEC2015	RVB29	1	Free Catch	1.024	6.5	Bilirubin 2+, blood trace	urine colour : yellow coloured and cloudy Rbc = 0 - 10 per HPF (x400) Wbc = 0 - 1 per HPF (x400) many fat droplets very occasional epithelial cell	Bilirubin 2+ and trace blood NCS
1	BIOEC2015	RVB29	3	Free Catch	1.020	8	Protein 1+	Urine colour: Yellow coloured and cloudy Rbc = 0 - 10 per HPF (x400) Wbc = 0 - 1 per HPF (x400) Organic debris. Unstained, clear granular debris. Occasional epithelial cell.	Protein 1+ NCS
1	BIOEC2015	RVB29	7	Free Catch	1.019	6	Nitrite positive, blood trace protein trace	urine colour : yellow coloured and cloudy Rbc = 0 - 10 per HPF (x400) Wbc = 0 - 2 per HPF (x400) bacteria (rods) amorphous clear crystals (most commonly phosphates or calcium oxalates) occasional squamous epithelial cell	Nitrite positive not clinically significant, likely contamination as free catch sample
1	BIOEC2015	RVB32	1	Free Catch	1.023	8	Protein trace, bilirubin 1+	urine colour : yellow coloured and cloudy Rbc = 0 - 10 per HPF (x400) Wbc = 0 - 1 per HPF (x400) fat droplets	Protein trace and bilirubin 1+ not clinically significant
1	BIOEC2015	RVB32	3	Free Catch	1.037	7	Protein trace, 2+ bilirubin	urine colour : yellow coloured and cloudy Rbc = 0 - 10 per HPF (x400) Wbc = 0 - 1 per HPF (x400) many fat droplets	Protein trace and 2+ bilirubin not clinically significant

1	BIOEC2015	RVB32	7	Free Catch	1.038	9	Protein 3+	urine colour : yellow coloured and turbid = Rbc = 0 - 10 per HPF (x400)<br>Wbc = 0 - 1 per HPF (x400)<br>many fat droplets	Protein 3+ NCS
1	BIOEC2015	RVB35	1	Free Catch	1.018	8.5	Trace protein	Urine colour: straw coloured and slightly cloudy = Rbc = 0 - 10 per HPF (x400)<br>Wbc = 0 - 1 per HPF (x400)<br>Few fat droplets.	
1	BIOEC2015	RVB35	3	Free Catch	1.030	6.5	Negative	Urine colour : yellow coloured and very slightly cloudy = Rbc = 0 - 10 per HPF (x400)<br>Wbc = 0 - 1 per HPF (x400)<br>some debris<br>few fat droplets	
1	BIOEC2015	RVB35	7	Free Catch	1.035	7	Blood trace	Urine colour: pale orange coloured and cloudy = Rbc = 5 - 10 per HPF (x400)<br>Wbc = 1 - 3 per HPF (x400)<br>Triple phosphate crystals.	Blood trace NCS

*Negative results on urine dip stick not listed in table.

NCS = not clinically significant.

TABLE A16. Urinalysis Group 2

Group	Treatment	Patient No.	Visit No.	Urine Collection Method	Urine Specific Gravity	Urine-pH	Urine Dip Stick *	Urine Sediment	Investigator Comment
2	Placebo	RVB02	1	Free Catch	1.043	6	Negative	Urine colour: yellow and turbid. RBC = 0-10 per HPF (x400) WBC = 0-1 per HPF (x400). Many Fat droplets	Trace of blood likely as a result of cystocentesis sampling (i.e. Iatrogenic bleeding)
2	Placebo	RVB02	3	Unable to obtain	>1.050				
2	Placebo	RVB02	7	Free Catch	1.040	9	Negative	Urine colour: straw coloured and cloudy. RBC = 0-10 per HPF (x400) WBC = 0-1 per HPF (x400). Some stained/organic debris. Triple phosphate crystals. Amorphous clear crystals (may be phosphates or calcium oxalates) occasional epithelial cell	
2	Placebo	RVB05	1	Free Catch	1.046	6	Protein 1+, Bilirubin 2+	Urine Colour : amber and cloudy = RBC = 0 - 10 per HPF (x400) WBC = 0 - 1 per HPF (x400) Some stained/organic debris Fat droplets Some epithelial cell	Protein 1+ considered normal for highly concentrated urine, \bilirubin 2+ normal for male dog
2	Placebo	RVB09	1	Free Catch	1.016	5	Negative	urine colour : yellow coloured and clear = Rbc = 1 - 10 per HPF (x400) Wbc = 0 - 1 per HPF (x400) occasional epithelial cell	
2	Placebo	RVB09	3	None collected					
2	Placebo	RVB11	1	Free Catch	>1.050	6.5	Trace protein	urine colour : yellow coloured and slightly cloudy = Rbc = 0 - 10 per HPF (x400) Wbc = 25 - 50 per HPF (x400) few fat droplets squamous epithelial cells spermatozoa	WBC in sediment NCS

2	Placebo	RVB11	3	Free Catch	1.033	6	Protein 1+	urine colour : yellow and cloudy = Rbc = 0 - 10 per HPF (x400) Wbc = 0 - 1 per HPF (x400) spermatozoa Debris - some spherical yellow/green artefacts, possible contaminant if free catch/floor sample	Protein 1+ NCS
2	Placebo	RVB11	7	Free Catch	1.039	5	Trace protein Blood 2+	urine colour : yellow coloured and clear = Rbc = 0 - 10 per HPF (x400) Wbc = 0 - 1 per HPF (x400) very little debris	Blood 2+ NCS
2	Placebo	RVB12	1	cystocentesis	>1.050	6.5	Negative	Urine colour: amber coloured and cloudy. = Rbc = 0 - 10 per HPF (x400) Wbc = 0 - 1 per HPF (x400) Occasional triple phosphate crystal. Many fat droplets	
2	Placebo	RVB12	3	None collected					
2	Placebo	RVB20	1	Cystocentesis	1.013	8.5	Bilirubin 1+	urine colour : straw coloured and clear = Rbc = 0 - 10 per HPF (x400) Wbc = 0 - 1 per HPF (x400) few fat droplets	Bilirubin 1+ Not clinically significant
2	Placebo	RVB20	3	Free Catch	1.042	6.5	Trace protein	Urine colour: Yellow coloured and cloudy. = Rbc = 0 - 10 per HPF (x400) Wbc = 3 - 10 per HPF (x400) Few fat droplets. Few squamous epithelial cells.	
2	Placebo	RVB20	7	Free catch	1.034	6.5	Protein 1+, bilirubin 1+	urine colour : yellow coloured and cloudy = Rbc = 0 - 10 per HPF (x400) Wbc = 50 - 75 per HPF (x400) triple phosphate crystals squamous epithelial cells	sediment and white blood cells, 1+ protein and 1+ bilirubin not of clinical significance
2	Placebo	RVB28	1	Free Catch	1.042	8	Protein trace, Bilirubin 1+	urine colour : yellow coloured and cloudy = Rbc = 0 - 10 per HPF (x400) Wbc = 0 - 1 per HPF (x400) many fat droplets triple phosphate crystals amorphous clear crystals (most commonly phosphates or calcium oxalates)	Bilirubin 1+ NCS

2	Placebo	RVB30	1	Free Catch	1.035	6	Bilirubin 1+	urine colour : yellow coloured and slightly cloudy = Rbc = 0 - 10 per HPF (x400) Wbc = 0 - 1 per HPF (x400) few fat droplets	Bilirubin 1+ NCS
2	Placebo	RVB31	1	Free Catch	>1.050	6.5	Protein 1+ Ketones trace	Urine colour: yellow coloured and cloudy. = Rbc = 0 - 10 per HPF (x400) Wbc = 0 - 1 per HPF (x400) Triple phosphate crystals. Occasional epithelial cell	Protein 1+, ketones trace and triple phosphate crystals NCS
2	Placebo	RVB31	3	Free Catch	1.025	5.5	Negative	Urine colour: Straw coloured and slightly cloudy = Rbc = 0 - 10 per HPF (x400) Wbc = 0 - 1 per HPF (x400) Some stained/ organic debris. Very occasional epithelial cell	
2	Placebo	RVB31	7	Free Catch	1.050	5	protein trace	urine colour : amber coloured and clear = Rbc = 0 - 10 per HPF (x400) Wbc = 0 - 1 per HPF (x400) some stained/organic debris few fat droplets	
2	Placebo	RVB34	1	Free Catch	1.031	8	Protein trace, bilirubin 1+	urine colour : yellow coloured and cloudy = Rbc = 0 - 10 per HPF (x400) Wbc = 0 - 1 per HPF (x400) fat droplets squamous epithelial cells	Bilirubin 1+ not clinically significant
2	Placebo	RVB34	3	Free Catch	1.044	5.5	bilirubin 2+	Urine colour: orange coloured and cloudy = Rbc = 0 - 10 per HPF (x400) Wbc = 0 - 1 per HPF (x400) Squamous epithelial cells. Fat droplets.	Bilirubin 2+ NCS
2	Placebo	RVB34	7	Free Catch	1.050	5.5	Protein trace, Bilirubin 2+	Urine colour: orange coloured and slightly cloudy = Rbc = 0 - 10 per HPF (x400) Wbc = 0 - 1 per HPF (x400) Occasional squamous epithelial cell. Fat droplets.	Bilirubin 2+ NCS

*Negative results on urine dip stick not listed in table.

NCS = not clinically significant.

TABLE A17. Faecal tests.

Group	Treatment	Patient No.	Visit No.	Faecal Culture Result	Faecal Culture - Lab Comment	Faecal Parasites - Endo-egg count	Investigator comment
1	BIOEC2015	RVB01	1	-ve	Normal Faecal flora isolated	no faecal eggs or coccidia oocysts seen	
1	BIOEC2015	RVB07	1	-ve	Normal faecal flora only	no faecal eggs or coccidia oocysts seen	
1	BIOEC2015	RVB08	1	-ve	Normal faecal flora only	no faecal eggs or coccidia oocysts seen	
1	BIOEC2015	RVB13	1				
1	BIOEC2015	RVB16	1	Growth of campylobacter spp. isolated after 48 hours incubation	Growth of Campylobacter spp. isolated after 48 hours incubation. The isolated Campylobacter spp. has been identified as Campylobacter coli.	No faecal eggs or coccidia oocysts seen	Clinical significance unknown as can be present in clinically well patients. In the absence of haemorrhagic diarrhoea, significance of campylobacter spp. tenuous. I would not ascribe any clinical significance to this observation.
1	BIOEC2015	RVB18	1	-ve	Normal faecal flora	no faecal eggs or coccidia oocysts seen	
1	BIOEC2015	RVB27	1				
1	BIOEC2015	RVB29	1	-ve	Normal faecal flora after 48 hours incubation.<br>No Campylobacter isolated.<br>No Salmonella spp. isolated.	No faecal eggs or coccidia oocyts seen	
1	BIOEC2015	RVB32	1				
1	BIOEC2015	RVB32	2	-ve	Normal faecal flora after 48 hours incubation.<br>No Campylobacter isolated.<br>No Salmonella spp. isolated.	No faecal egg or coccidia oocyst seen	
1	BIOEC2015	RVB35	1	Campylobacter spp isolated. No salmonella spp or Yersinia enterocolitica isolated	Campylobacter spp isolated, No salmonella spp or Yersinia enterocolitica isolated	No faecal egg or coccidia oocyt seen	In the absence of haemorrhagic diarrhoea significance of Campylobacter spp. Tenuous. I do not ascribe any clinical significance to this observation for this patient
2	Placebo	RVB02	1	-ve	Normal faecal flora only	<50 Capillaria eggs per gram seen, average size 70x30 microns	

2	Placebo	RVB05	1	-ve	Normal faecal flora only	no faecal eggs or coccidia oocysts seen	
2	Placebo	RVB09	1	-ve	Normal faecal flora only	No faecal eggs or coccidia oocysts seen	
2	Placebo	RVB11	1	Campylobacter lari isolated	Campylobacter lari isolated, mixed faecal flora	Zinc sulphate flotation with centrifugation: Giardia cysts present	
2	Placebo	RVB12	1	-ve	Normal faecal flora only	No faecal eggs or coccidia oocysts seen	NCS
2	Placebo	RVB20	1				
2	Placebo	RVB28	1	-ve	Normal faecal flora after 48 hours incubation. = No Campylobacter isolated. No Salmonella spp. isolated.	2500 Capillaria eggs per gram seen, average size 65 x 30 microns	Not responsible for diarrhoea but most likely respiratory/urinary disease. Should also be cleared up after treatment with fenbendazole
2	Placebo	RVB30	1	-ve	Normal faecal flora isolated = No Salmonella spp, Campylobacter spp or Yersinia enterocolitica isolated	No faecal egg or coccidia oocyst seen	
2	Placebo	RVB31	1	-ve	Normal faecal flora isolated = No Salmonella spp, Campylobacter spp or Yersinia enterocolitica isolated	No faecal egg or coccidia oocyst seen	
2	Placebo	RVB34	1	-ve	Normal faecal flora isolated. No campylobacter spp, Yersinis enterocolitica or Salmonella spp isolated	No faecal egg or coccidia oocyst seen	

NCS = not clinically significant.

A faecal sample not collected at Visit 1 for cases RVB13, 20, 27 and 32. A faecal sample for RVB32 was submitted at visit 2 instead.

TABLE A18. IBD Physical exam.

Group	Treatment	Patient No.	Visit No.	Abdominal Pain/Discomfort	Pain On Palpation	Pruritis	Ascites and Peripheral Odema
1	BIOEC2015	RVB01	1	None	No	None	None
1	BIOEC2015	RVB01	2	Hardly ever	No	None	None
1	BIOEC2015	RVB01	3	None	No	None	None
1	BIOEC2015	RVB01	7	None		None	None
1	BIOEC2015	RVB07	1	Hardly ever	No	None	None
1	BIOEC2015	RVB07	2	Occasionally	No	None	None
1	BIOEC2015	RVB08	1	None	No	None	None
1	BIOEC2015	RVB08	2	Occasionally	No	None	None
1	BIOEC2015	RVB08	7	None	No	None	None
1	BIOEC2015	RVB08	3	None	No	None	None
1	BIOEC2015	RVB13	1	None	No	None	None
1	BIOEC2015	RVB13	3	None	No	None	None
1	BIOEC2015	RVB13	7	None	No	None	None
1	BIOEC2015	RVB13	2	None	No	None	None
1	BIOEC2015	RVB16	1	Occasionally	No	None	None

1	BIOEC2015	RVB16	2	Occasionally	No	Occasional episodes of itching	None
1	BIOEC2015	RVB16	3	Occasionally	No	None	None
1	BIOEC2015	RVB16	7	None	No	None	None
1	BIOEC2015	RVB18	1	Occasionally	No	None	None
1	BIOEC2015	RVB18	2	Hardly ever	No	None	None
1	BIOEC2015	RVB18	3	None	No	None	None
1	BIOEC2015	RVB18	7	None	No	None	None
1	BIOEC2015	RVB27	1	None	No	None	None
1	BIOEC2015	RVB29	1	None	No	None	None
1	BIOEC2015	RVB29	2	None	No	None	None
1	BIOEC2015	RVB29	3	Hardly ever	No	None	None
1	BIOEC2015	RVB29	7	Occasionally	No	Regular episodes of itching but not when sleeping	None
1	BIOEC2015	RVB32	1	None	No	None	None
1	BIOEC2015	RVB32	2	None	No	None	None
1	BIOEC2015	RVB32	3	None	No	None	None
1	BIOEC2015	RVB32	7	None	No	None	None
1	BIOEC2015	RVB35	1	Occasionally	No	None	None
1	BIOEC2015	RVB35	2	None	No	None	None

1	BIOEC2015	RVB35	3	None	No	None	None
1	BIOEC2015	RVB35	7	None	No	None	None
2	Placebo	RVB02	1	Hardly ever	No	None	None
2	Placebo	RVB02	2	None	No	None	None
2	Placebo	RVB02	3	Hardly ever	No	None	None
2	Placebo	RVB02	7	None	No	None	None
2	Placebo	RVB05	1	None	No	None	None
2	Placebo	RVB09	1	Hardly ever	No	None	None
2	Placebo	RVB09	2	None	No	None	None
2	Placebo	RVB09	3	None	No	None	None
2	Placebo	RVB11	1	None	No	None	None
2	Placebo	RVB11	2	None	No	None	None
2	Placebo	RVB11	3	None	No	None	None
2	Placebo	RVB11	7	None	No	None	None
2	Placebo	RVB12	1	Occasionally	No	None	None
2	Placebo	RVB12	2	None	No	None	None
2	Placebo	RVB12	3	Occasionally	No	None	None
2	Placebo	RVB20	1	None	No	None	None
2	Placebo	RVB20	2	None	No	None	None
2	Placebo	RVB20	3	Hardly ever	No	None	None
2	Placebo	RVB20	7	None	No	None	None
2	Placebo	RVB28	1	None	No	None	None
2	Placebo	RVB28	2	Every day	No	None	None
2	Placebo	RVB30	1	Occasionally	No	None	None
2	Placebo	RVB30	2	None	No	None	None
2	Placebo	RVB31	1	Occasionally	No	Occasional episodes of itching	None
2	Placebo	RVB31	2	None	No	Occasional episodes of itching	None
2	Placebo	RVB31	3	None	No	Occasional episodes of itching	None
2	Placebo	RVB31	7	Other	No	None	None

2	Placebo	RVB34	1	None	No	None	None
2	Placebo	RVB34	2	None	No	None	None
2	Placebo	RVB34	3	None	No	None	None
2	Placebo	RVB34	7	None	No	None	None

TABLE A19. Drinking.

Group	Treatment	Patient No.	Visit 1	Visit 2	Visit 3	Visit 7	Withdrawal
1	BIOEC2015	RVB01	Normal	Normal	Normal	Normal	
1	BIOEC2015	RVB07	Normal	Increased			Increased
1	BIOEC2015	RVB08	Normal	Increased	Normal	Normal	
1	BIOEC2015	RVB13	Normal	Normal	Normal	Normal	
1	BIOEC2015	RVB16	Normal	Normal	Normal	Normal	
1	BIOEC2015	RVB18	Decreased	Normal	Normal	Normal	
1	BIOEC2015	RVB27	Normal				
1	BIOEC2015	RVB29	Normal	Normal	Normal	Normal	
1	BIOEC2015	RVB32	Normal	Decreased	Normal	Normal	
1	BIOEC2015	RVB35	Normal	Normal	Normal	Normal	
2	Placebo	RVB02	Normal	Normal	Normal	Normal	
2	Placebo	RVB05	Normal				
2	Placebo	RVB09	Normal	Normal	Increased		Increased
2	Placebo	RVB11	Normal	Normal	Normal	Normal	
2	Placebo	RVB12	Normal	Normal	Normal		Normal
2	Placebo	RVB20	Normal	Normal	Normal	Normal	
2	Placebo	RVB28	Increased	Normal			
2	Placebo	RVB30	Normal	Increased			
2	Placebo	RVB31	Normal	Increased	Normal	Normal	
2	Placebo	RVB34	Increased	Normal	Normal	Normal	

TABLE A20. Diet.

Group	Treatment	Patient No.	Pre Visit 1		Visit 1 - 2	Visit 2 - 3	Visit 3 - 4	Visit 4 - 5		Visit 5 - 6		Visit 6 - 7	
			Diet History	Treats	Purina HA/day	Purina HA/day	Purina HA/day	Feed/day	Treats	Feed/day	Treats	Feed/day	Treats
1	BIOEC2015	RVB01	Purina HA. Fish and potatoes diet. Hills z/d (1tin + 100g dry/day)		220g	180g	200g	160g Hills z/d dry		135g Purina HA + 36g z/d dry		190g Purina HA	Advance Hydrolysed treat
1	BIOEC2015	RVB07	Royal Canin GI Low fat. id canine low fat. Chappie (fish) and butchers (tripe & chicken and tripe & beef) - two meals a day.		As much as he wants								
1	BIOEC2015	RVB08	Royal Canin GI, boiled chicken and rice 125g BID		315g	380g	310g	128g Purina HA and 26g Arden Grange Sensitive White fish and potato		48g Purina HA and 98g Arden Grange		130g Purina HA and 40g Arden Grange	
1	BIOEC2015	RVB13	Chappie fed long term. Tried Hills z/d but had severe diarrhoea so changed back to Chappie. 824g Chappie and 100g		440g	440g	400g	Chappie /Purina mix from V4 but clinical signs worsened		440g Purina HA + 1/2 handful of Chappie dry		440g Purina HA and WAGG	

			WAGG meaty chunks per day.					so exclusively fed 440g Purina HA					
1	BIOEC2015	RVB16	Hills w/d, chicken, low fat Royal Canin		120g	170g	170g	180g Hills w/d dry		180g Hills w/d dry		180g Hills w/d dry	Hills w/d dry
1	BIOEC2015	RVB18	Started RCW Hypoallergenic diet (150g) on 15Jul16, previous to this was on James Well Beloved per day.		155g	155g	155g	155g Purina HA		155g Purina HA		155g Purina HA	Wainwrights hypoallergenic treats x 2
1	BIOEC2015	RVB27	Butchers 400g and sausages	Sausages									
1	BIOEC2015	RVB29	Purina HA (375g) from Feb 17. Dog chews, vegetables	Dog chews, carrots, vegetables	300g	325g	325g	300-350g Purina HA		230g Purina HA and 120g Barking Heads gentle digestion with chicken		280-300g Purina HA	
1	BIOEC2015	RVB32	Chappie at rehoming centre and a few weeks after rehoming. 150g dry plus one tin Milly's Wolfeheart per day since then	Pedigree dentistix SID plus pedigree tasty treats	350g	350g	350g	150g dry plus 1 tin Millies Wolfheart	Bonio x 2 per day	150g dry plus 1 tin Millies Wolfheart	Bonio x 2 per day, Dentisti x x 1 per day	150g dry plus 1 tin Millies Wolfheart	Bonio x 2 per day, Dentistix x 1 per day

1	BIOEC2015	RVB35	Butchers Original dry (250g). Chicken and rice	Dentastix, dog chews-beef	326g	326g	326g	250g Butchers original dry	Dentastix	250g Butchers original dry	Dentastix	326g Bakers dry food	Dentastix, Meaty chews
2	Placebo	RVB02	James Well beloved Lamb and rice. Hills i/d duck and rice. Chicken. White fish. 450g /day of Lily's Kitchen Venison	Tit-bits	160g	160g	160g	160g Wainwrights Rabbit and Vegetable + Purina HA		160g Fish complete diet		160g Purina HA and 2 sardines per day	Rawhide chew and Liver and Venison
2	Placebo	RVB05	James Well beloved wet and dry food. Hills i/d chicken and rice.	chicken									
2	Placebo	RVB09	Cooked chicken with rice/potato and cooked lamb. This is the most recent but was on a GI diet for a short while.		400g	300g - not wanting to eat as much as before							
2	Placebo	RVB11	Previously tried i/d diet, starting on 01 Dec 15 for ~ 3 weeks. 500g/day of raw food mixed with kibble (Origin Tundra)		600g	500-600g	700g	340g Orijen Tundra		700g Purina HA		700g Purina HA	

2	Placebo	RVB12	Feeding chicken and rice or WAGG sensitive. Was on James Well beloved before that.		4 cups	4 cups							
2	Placebo	RVB20	Chicken and rice, Hills z/d. Natures menu raw food diet (300g meat, 100g veg, 100g biscuit)	Bonio. Although stopped recently	300g	300g	300g	310g Chappie Dry		310g Chappie dry, beef flavoured		310g Chappie dry beef wholegrain	Chappie dry beef wholegrain
2	Placebo	RVB28	Puppy dry food, Purina HA, Anallergenic (Royal Canin), chicken/lamb/brown rice		6 handfuls								
2	Placebo	RVB30	Raw food diet initially then at 6 months age changed on to Caninde Duck dry biscuits and cooked chicken. One week ago changed to Purina HA (360g) and Hills z/d (6 spoonful's) per day.		240g								
2	Placebo	RVB31	Chappie, i/d (Hills) with chicken, Burns sensitivity, Hills low fat i/d (1tin/day).		200g	210g	190-200g	200g Purina HA		200g Purina HA		190-200g Purina HA	

2	Placeb o	RVB34	Royal Canin Veterinary Gastro intestinal (350g/day) and fresh chicken. Hills i/d tinned.	Fresh chicken	310g	310g	310g	310g Hills sensitive stomach adult	Training treats - chicken flavour	310g Hills sensitive stomach adult	Training treats (chicken flavour) and small amount s of roast chicken on Sundays	320g Hills science plan sensitive stomach adult	Chicken and chicken based treats

TABLE A21. General physical exam.

Group	Treatment	Patient No.	Visit No.	Temperature (ºC)	Respiratory System	Cardiac system	Musculo-Skeletal system	Comments	Current Weight (kg)	* Baseline Weight (kg)	Baseline Weight Date	Estimated Weight Loss
1	BIOEC2015	RVB01	1	38.5	Normal	Normal	Normal		10.10	10.60	15-Sep-15	Mild (<5%)
1	BIOEC2015	RVB01	2	38.5	Normal	Normal	Normal		10.60	10.10	13-Oct-15	None
1	BIOEC2015	RVB01	3	38.3	Normal	Normal	Normal		10.35	10.10	13-Oct-15	None
1	BIOEC2015	RVB01	7	38.2	Normal	Normal	Normal		10.40	10.10	13-Oct-15	None
1	BIOEC2015	RVB07	1	38.4	Normal	Normal	Abnormal - clinically significant	Muscle mass loss	18.70	19.20	30-Nov-15	Mild (<5%)
1	BIOEC2015	RVB07	2	38.3	Normal	Normal	Abnormal - not clinically significant	mild muscle atrophy: HL and temporal + epaxial	18.45	18.70	30-Nov-15	Mild (<5%)
1	BIOEC2015	RVB08	1	37.4	Normal	Normal	Normal		17.40	19.00	25-Sep-15	Moderate (5-10%)
1	BIOEC2015	RVB08	2	37.9	Normal	Normal	Normal		18.15	17.40	07-Dec-15	None
1	BIOEC2015	RVB08	7	38.8	Normal	Normal	Normal	Within Normal Limits	18.45	17.40	07-Dec-15	None

1	BIOE C201 5	RVB0 8	3	38.0	Normal	Normal	Normal		18.25	17.40	07-Dec-15	None
1	BIOE C201 5	RVB1 3	1	38.2	Normal	Normal	Normal		36.50	38.10	01-Jun-16	Mild (<5%)
1	BIOE C201 5	RVB1 3	3	38.2	Normal	Normal	Normal		38.95	36.50	08-Jun-16	None
1	BIOE C201 5	RVB1 3	7	38.1	Normal	Normal	Normal		39.10	36.50	08-Jun-18	None
1	BIOE C201 5	RVB1 3	2	38.9	Normal	Normal	Normal		37.60	36.50	08-Jun-16	None
1	BIOE C201 5	RVB1 6	1	38.0	Normal	Abnormal - not clinically significant	Normal	II/VI left sided systolic murmur	9.60	9.40	18-Jul-16	None
1	BIOE C201 5	RVB1 6	2	39.1	Normal	Abnormal - not clinically significant	Normal	Grade II/VI L apical murmur	8.90	9.60	20-Jul-16	Moderate (5-10%)
1	BIOE C201 5	RVB1 6	3	39.5	Normal	Normal	Normal		9.50	9.60	20-Jul-16	Mild (<5%)
1	BIOE C201 5	RVB1 6	7	38.7	Normal	Abnormal - not clinically significant	Normal	III/VI left sided systolic murmur	9.85	9.60	20-Jul-16	None
1	BIOE C201 5	RVB1 8	1	38.6	Normal	Normal	Normal		7.85	7.50	09-Apr-15	None
1	BIOE C201 5	RVB1 8	2	39.0	Normal	Normal	Normal		8.30	7.85	02-Aug-16	None

1	BIOE C201 5	RVB1 8	3	38.2	Normal	Abnormal - not clinically significant	Normal	Grade I heart murmur	8.60	7.85	02-Aug-16	None
1	BIOE C201 5	RVB1 8	7	39.1	Normal	Normal	Normal		8.40	7.85	02-Aug-16	None
1	BIOE C201 5	RVB2 7	1	38.5	Normal	Normal	Normal		19.90	20.70	21-Dec-16	Mild (<5%)
1	BIOE C201 5	RVB2 9	1	38.3	Normal	Normal	Normal		25.85	26.60	09-Mar-17	Mild (<5%)
1	BIOE C201 5	RVB2 9	2	38.9	Normal	Normal	Normal		25.65	25.85	03-Apr-17	Mild (<5%)
1	BIOE C201 5	RVB2 9	3	38.5	Normal	Normal	Normal		26.05	25.85	03-Apr-17	None
1	BIOE C201 5	RVB2 9	7	39.0	Normal	Normal	Normal		26.50	25.85	03-Apr-17	None
1	BIOE C201 5	RVB3 2	1	38.6	Normal	Normal	Normal		22.60	24.60	07-May-17	Moderate (5-10%)
1	BIOE C201 5	RVB3 2	2	38.4	Normal	Abnormal - not clinically significant	Normal	Intermittent early systolic grade I/II left systolic heart murmur, suspect physiologic heart murmur	24.00	22.60	16-May-17	None

1	BIOE C201 5	RVB3 2	3	38.5	Normal	Abnormal - not clinically significant	Normal	Grade I/VI left apical systolic murmur	24.30	22.60	16-May-17	None
1	BIOE C201 5	RVB3 2	7	38.7	Normal	Abnormal - not clinically significant	Normal	G I/VI apical systolic murmur	22.95	22.60	16-May-17	None
1	BIOE C201 5	RVB3 5	1	38.4	Normal	Normal	Normal		18.80	18.60	27-Nov-17	None
1	BIOE C201 5	RVB3 5	2	38.7	Normal	Normal	Normal		17.05	18.80	04-Dec-17	Moderate (5-10%)
1	BIOE C201 5	RVB3 5	3	38.4	Normal	Normal	Normal		18.40	18.80	04-Dec-17	Mild (<5%)
1	BIOE C201 5	RVB3 5	7	37.7	Normal	Normal	Normal		20.60	18.80	04-Dec-17	None
2	Place bo	RVB0 2	1	38.2	Normal	Normal	Normal		8.20	8.20	14-Oct-15	None
2	Place bo	RVB0 2	2	37.9	Normal	Normal	Normal		7.95	8.20	14-Oct-15	Mild (<5%)
2	Place bo	RVB0 2	3	38.3	Normal	Normal	Normal		8.05	8.20	14-Oct-15	Mild (<5%)
2	Place bo	RVB0 2	7	38.1	Normal	Normal	Normal		8.40	8.20	14-Oct-15	None
2	Place bo	RVB0 5	1	38.8	Normal	Normal	Normal		20.30	21.80	21-Oct-15	Moderate (5-10%)
2	Place bo	RVB0 9	1	38.6	Normal	Normal	Normal		30.40	30.05	12-Jan-16	None
2	Place bo	RVB0 9	2	38.6	Normal	Normal	Normal		31.50	30.40	26-Jan-16	None
2	Place bo	RVB0 9	3	38.9	Normal	Normal	Normal		27.30	30.40	26-Jan-16	Severe (>10%)

2	Placebo	RVB11	1	38.3	Normal	Normal	Normal	underweight	29.00	29.35	30-Dec-15	Mild (<5%)
2	Placebo	RVB11	2	38.2	Normal	Normal	Normal		29.25	29.00	01-Feb-16	None
2	Placebo	RVB11	3	38.0	Normal	Normal	Normal		30.00	29.00	01-Feb-16	None
2	Placebo	RVB11	7	Not taken	Normal	Normal	Normal		30.35	29.00	01-Feb-16	None
2	Placebo	RVB12	1	38.4	Normal	Normal	Normal		34.00	33.85	07-Mar-16	None
2	Placebo	RVB12	2	39.2	Normal	Normal	Normal		35.00	34.00	09-Mar-16	None
2	Placebo	RVB12	3	39.2	Normal	Normal	Normal		33.75	34.00	09-Mar-16	Mild (<5%)
2	Placebo	RVB20	1	38.8	Normal	Normal	Normal		18.15	18.60	11-Aug-16	Mild (<5%)
2	Placebo	RVB20	2	38.2	Normal	Normal	Normal		19.50	18.15	23-Aug-16	None
2	Placebo	RVB20	3	38.3	Normal	Normal	Normal		20.80	18.15	23-Aug-16	None
2	Placebo	RVB20	7	38.6	Normal	Normal	Normal		20.20	18.15	23-Aug-16	None
2	Placebo	RVB28	1	38.8	Normal	Normal	Normal		9.00	8.95	18-Nov-16	None
2	Placebo	RVB28	2	38.1	Normal	Normal	Normal		8.70	9.00	30-Mar-17	Mild (<5%)
2	Placebo	RVB30	1	37.8	Abnormal - not clinically significant	Normal	Normal		13.00	13.70	27-Mar-17	Moderate (5-10%)
2	Placebo	RVB30	2	38.3	Abnormal - not clinically significant	Normal	Normal	BOAS, not causing problems at present	13.25	13.00	10-Apr-17	None

2	Place bo	RVB3 1	1	37.8	Normal	Abnormal - not clinically significant	Normal	Grade II/VI left apical systolic murmur	10.40	10.30	07-Apr-17	None
2	Place bo	RVB3 1	2	38.3	Normal	Normal	Normal		10.75	10.40	18-Apr-17	None
2	Place bo	RVB3 1	3	38.6	Normal	Normal	Normal		12.30	10.40	18-Apr-17	None
2	Place bo	RVB3 1	7	38.4	Normal	Abnormal - not clinically significant	Normal	Grade I/VI left sided heart murmur noted	12.15	10.40	18-Apr-18	None
2	Place bo	RVB3 4	1	38.4	Normal	Normal	Normal		24.25	24.60	11-Sep-17	Mild (<5%)
2	Place bo	RVB3 4	2	37.6	Normal	Normal	Normal		24.10	24.25	13-Sep-17	Mild (<5%)
2	Place bo	RVB3 4	3	37.7	Normal	Normal	Normal		24.75	24.25	13-Sep-17	None
2	Place bo	RVB3 4	7	38.7	Normal	Normal	Normal		26.15	24.25	13-Sep-17	None

* Baseline weight at Visit 1 is last documented weight by referring veterinary surgeon, at subsequent visits the weight from Visit 1 was used the baseline weight

TABLE A22. CCECAI Scores.

Group	Treatment	Patient No.	Visit No.	Attitude /Activity Score	Appetite Score	Vomiting Score	Stool Consistency Score	Stool Frequency Score	Weight Loss Score	Albumin Levels Score	Ascites and Peripheral Oedema Score	Pruritus Score	CCECAI Total
1	BIOEC2015	RVB01	1	0 = normal	0 = normal	0 = normal	1 = slightly soft faeces or faecal blood, mucus or both	1 = slightly increased (2-3 x per day)	0 = None	0 = >20g/l	0 = none	0 = no puritis	2
1	BIOEC2015	RVB01	2	1 = slightly decreased	0 = normal	1 = mild (up to once a week)	0 = normal	0 = normal	0 = None	0 = >20g/l	0 = none	0 = no puritis	2
1	BIOEC2015	RVB01	3	0 = normal	0 = normal	0 = normal	1 = slightly soft faeces or faecal blood, mucus or both	1 = slightly increased (2-3 x per day)	1 = mild (<5%)	0 = >20g/l	0 = none	0 = no puritis	3
1	BIOEC2015	RVB01	7	0 = normal	0 = normal	0 = normal	0 = normal	1 = slightly increased (2-3 x per day)	0 = None	0 = >20g/l	0 = none	0 = no puritis	1
1	BIOEC2015	RVB07	1	0 = normal	0 = normal	0 = normal	4 = watery diarrhoea	1 = slightly increased (2-3 x per day)	1 = mild (<5%)	0 = >20g/l	0 = none	0 = no puritis	6
1	BIOEC2015	RVB07	2	0 = normal	0 = normal	0 = normal	4 = watery diarrhoea	0 = normal	1 = mild (<5%)	1 = 15-19.9g/l	0 = none	0 = no puritis	6
1	BIOEC2015	RVB08	1	0 = normal	0 = normal	0 = normal	2 = very soft faeces	3 = severely increased (>5 x per day)	2 = moderate (5-10%)	0 = >20g/l	0 = none	0 = no puritis	7
1	BIOEC2015	RVB08	2	0 = normal	2 = moderately decreased	0 = normal	0 = normal	1 = slightly increased (2-3 x per day)	0 = None	0 = >20g/l	0 = none	0 = no puritis	3

1	BIO EC2 015	RVB 08	7	0 = normal	0 = normal	0 = normal	0 = normal	1 = slightly increased (2-3 x per day)	0 = None	0 = >20g/l	0 = none	0 = no puritis	1
1	BIO EC2 015	RVB 08	3	0 = normal	1 = slightly decreased	0 = normal	1 = slightly soft faeces or faecal blood, mucus or both	1 = slightly increased (2-3 x per day)	0 = None	0 = >20g/l	0 = none	0 = no puritis	3
1	BIO EC2 015	RVB 13	1	0 = normal	0 = normal	3 = severe (>3x per week)	0 = normal	0 = normal	1 = mild (<5%)	0 = >20g/l	0 = none	0 = no puritis	4
1	BIO EC2 015	RVB 13	3	0 = normal	0 = normal	1 = mild (up to once a week)	0 = normal	0 = normal	0 = None	0 = >20g/l	0 = none	0 = no puritis	1
1	BIO EC2 015	RVB 13	7	0 = normal	0 = normal	0 = normal	0 = normal	1 = slightly increased (2-3 x per day)	0 = None	0 = >20g/l	0 = none	0 = no puritis	1
1	BIO EC2 015	RVB 13	2	0 = normal	0 = normal	0 = normal	0 = normal	0 = normal	0 = None	0 = >20g/l	0 = none	0 = no puritis	0
1	BIO EC2 015	RVB 16	1	1 = slightly decrease d	0 = normal	2 = moderate (1-3 times per week)	1 = slightly soft faeces or faecal blood, mucus or both	2 = moderately increased (4-5 x per day)	0 = None	0 = >20g/l	0 = none	1 = occasional episodes of itching	7
1	BIO EC2 015	RVB 16	2	1 = slightly decrease d	1 = slightly decreased	2 = moderate (1-3 times per week)	1 = slightly soft faeces or faecal blood, mucus or both	1 = slightly increased (2-3 x per day)	2 = modera te (5-10%)	0 = >20g/l	0 = none	1 = occasional episodes of itching	9
1	BIO EC2 015	RVB 16	3	1 = slightly decrease d	1 = slightly decreased	2 = moderate (1-3 times per week)	1 = slightly soft faeces or faecal blood, mucus or both	1 = slightly increased (2-3 x per day)	1 = mild (<5%)	0 = >20g/l	0 = none	0 = no puritis	7
1	BIO EC2 015	RVB 16	7	0 = normal	0 = normal	0 = normal	0 = normal	2 = moderately increased (4-5 x per day)	0 = None	0 = >20g/l	0 = none	0 = no puritis	2

1	BIO EC2 015	RVB 18	1	1 = slightly decrease d	1 = slightly decreased	3 = severe (>3x per week)	0 = normal	1 = slightly increased (2-3 x per day)	0 = None	0 = >20g/l	0 = none	0 = no puritis	6
1	BIO EC2 015	RVB 18	2	0 = normal	0 = normal	0 = normal	0 = normal	0 = normal	0 = None	0 = >20g/l	0 = none	0 = no puritis	0
1	BIO EC2 015	RVB 18	3	0 = normal	0 = normal	1 = mild (up to once a week)	0 = normal	0 = normal	0 = None	0 = >20g/l	0 = none	0 = no puritis	1
1	BIO EC2 015	RVB 18	7	0 = normal	0 = normal	0 = normal	0 = normal	1 = slightly increased (2-3 x per day)	0 = None	0 = >20g/l	0 = none	0 = no puritis	1
1	BIO EC2 015	RVB 27	1	0 = normal	0 = normal	2 = moderate (1-3 times per week)	1 = slightly soft faeces or faecal blood, mucus or both	1 = slightly increased (2-3 x per day)	1 = mild (<5%)	0 = >20g/l	0 = none	0 = no puritis	5
1	BIO EC2 015	RVB 29	1	0 = normal	0 = normal	2 = moderate (1-3 times per week)	0 = normal	0 = normal	1 = mild (<5%)	0 = >20g/l	0 = none	0 = no puritis	3
1	BIO EC2 015	RVB 29	2	0 = normal	1 = slightly decreased	0 = normal	0 = normal	0 = normal	1 = mild (<5%)	0 = >20g/l	0 = none	0 = no puritis	2
1	BIO EC2 015	RVB 29	3	0 = normal	0 = normal	0 = normal	0 = normal	0 = normal	0 = None	0 = >20g/l	0 = none	0 = no puritis	0
1	BIO EC2 015	RVB 29	7	0 = normal	1 = slightly decreased	0 = normal	0 = normal	0 = normal	0 = None	0 = >20g/l	0 = none	2 = regular episodes of itching , but stops when asleep	3
1	BIO EC2 015	RVB 32	1	0 = normal	0 = normal	1 = mild (up to once a week)	1 = slightly soft faeces or faecal blood, mucus or both	1 = slightly increased (2-3 x per day)	2 = modera te (5-10%)	0 = >20g/l	0 = none	0 = no puritis	5

1	BIO EC2 015	RVB 32	2	0 = normal	0 = normal	0 = normal	0 = normal	1 = slightly increased (2-3 x per day)	0 = None	0 = >20g/l	0 = none	0 = no puritis	1
1	BIO EC2 015	RVB 32	3	0 = normal	0 = normal	0 = normal	0 = normal	1 = slightly increased (2-3 x per day)	0 = None	0 = >20g/l	0 = none	0 = no puritis	1
1	BIO EC2 015	RVB 32	7	0 = normal	0 = normal	0 = normal	0 = normal	1 = slightly increased (2-3 x per day)	0 = None	0 = >20g/l	0 = none	0 = no puritis	1
1	BIO EC2 015	RVB 35	1	1 = slightly decrease d	0 = normal	0 = normal	1 = slightly soft faeces or faecal blood, mucus or both	1 = slightly increased (2-3 x per day)	2 = modera te (5-10%)	0 = >20g/l	0 = none	0 = no puritis	5
1	BIO EC2 015	RVB 35	2	0 = normal	0 = normal	0 = normal	0 = normal	1 = slightly increased (2-3 x per day)	2 = modera te (5-10%)	0 = >20g/l	0 = none	0 = no puritis	3
1	BIO EC2 015	RVB 35	3	0 = normal	0 = normal	0 = normal	0 = normal	1 = slightly increased (2-3 x per day)	1 = mild (<5%)	0 = >20g/l	0 = none	0 = no puritis	2
1	BIO EC2 015	RVB 35	7	0 = normal	0 = normal	0 = normal	0 = normal	1 = slightly increased (2-3 x per day)	0 = None	0 = >20g/l	0 = none	0 = no puritis	1
2	Plac ebo	RVB 02	1	1 = slightly decrease d	1 = slightly decreased	1 = mild (up to once a week)	1 = slightly soft faeces or faecal blood, mucus or both	1 = slightly increased (2-3 x per day)	0 = None	0 = >20g/l	0 = none	0 = no puritis	5
2	Plac ebo	RVB 02	2	0 = normal	0 = normal	0 = normal	0 = normal	0 = normal	1 = mild (<5%)	0 = >20g/l	0 = none	0 = no puritis	1
2	Plac ebo	RVB 02	3	0 = normal	0 = normal	1 = mild (up to once a week)	0 = normal	1 = slightly increased (2-3 x per day)	0 = None	0 = >20g/l	0 = none	0 = no puritis	2

2	Placebo	RVB 02	7	0 = normal	0 = normal	0 = normal	0 = normal	0 = normal	0 = None	0 = >20g/l	0 = none	0 = no puritis	0
2	Placebo	RVB 05	1	0 = normal	0 = normal	0 = normal	4 = watery diarrhoea	2 = moderately increased (4-5 x per day)	2 = moderate (5-10%)	1 = 15-19.9g/l	1 = mild ascites or peripheral edema	0 = no puritis	10
2	Placebo	RVB 09	1	0 = normal	0 = normal	0 = normal	4 = watery diarrhoea	1 = slightly increased (2-3 x per day)	0 = None	0 = >20g/l	0 = none	0 = no puritis	5
2	Placebo	RVB 09	2	0 = normal	0 = normal	0 = normal	4 = watery diarrhoea	0 = normal	0 = None	1 = 15-19.9g/l	0 = none	0 = no puritis	5
2	Placebo	RVB 09	3	0 = normal	1 = slightly decreased	0 = normal	4 = watery diarrhoea	0 = normal	3 = severe (>10%)	1 = 15-19.9g/l	0 = none	0 = no puritis	9
2	Placebo	RVB 11	1	0 = normal	0 = normal	1 = mild (up to once a week)	4 = watery diarrhoea	3 = severely increased (>5 x per day)	1 = mild (<5%)	0 = >20g/l	0 = none	0 = no puritis	9
2	Placebo	RVB 11	2	0 = normal	0 = normal	0 = normal	0 = normal	2 = moderately increased (4-5 x per day)	0 = None	0 = >20g/l	0 = none	0 = no puritis	2
2	Placebo	RVB 11	3	0 = normal	0 = normal	1 = mild (up to once a week)	0 = normal	3 = severely increased (>5 x per day)	0 = None	0 = >20g/l	0 = none	0 = no puritis	4
2	Placebo	RVB 11	7	0 = normal	0 = normal	0 = normal	3 = severe	3 = severely increased (>5 x per day)	0 = None	0 = >20g/l	0 = none	0 = no puritis	6
2	Placebo	RVB 12	1	0 = normal	0 = normal	1 = mild (up to once a week)	0 = normal	0 = normal	0 = None	0 = >20g/l	0 = none	0 = no puritis	1
2	Placebo	RVB 12	2	0 = normal	0 = normal	0 = normal	0 = normal	0 = normal	0 = None	0 = >20g/l	0 = none	0 = no puritis	0

2	Plac ebo	RVB 12	3	0 = normal	1 = slightly decreased	1 = mild (up to once a week)	3 = severe	0 = normal	1 = mild (<5%)	0 = >20g/l	0 = none	0 = no puritis	6
2	Plac ebo	RVB 20	1	0 = normal	0 = normal	0 = normal	3 = severe	1 = slightly increased (2-3 x per day)	1 = mild (<5%)	0 = >20g/l	0 = none	0 = no puritis	5
2	Plac ebo	RVB 20	2	0 = normal	0 = normal	1 = mild (up to once a week)	2 = very soft faeces	0 = normal	0 = None	0 = >20g/l	0 = none	0 = no puritis	3
2	Plac ebo	RVB 20	3	0 = normal	0 = normal	0 = normal	0 = normal	2 = moderately increased (4-5 x per day)	0 = None	0 = >20g/l	0 = none	0 = no puritis	2
2	Plac ebo	RVB 20	7	0 = normal	0 = normal	0 = normal	0 = normal	2 = moderately increased (4-5 x per day)	0 = None	0 = >20g/l	0 = none	0 = no puritis	2
2	Plac ebo	RVB 28	1	1 = slightly decrease d	0 = normal	0 = normal	4 = watery diarrhoea	2 = moderately increased (4-5 x per day)	0 = None	0 = >20g/l	0 = none	0 = no puritis	7
2	Plac ebo	RVB 28	2	0 = normal	0 = normal	0 = normal	2 = very soft faeces	1 = slightly increased (2-3 x per day)	1 = mild (<5%)	0 = >20g/l	0 = none	0 = no puritis	4
2	Plac ebo	RVB 30	1	2 = moderat ely decrease d	3 = severely decreased	0 = normal	3 = severe	0 = normal	2 = modera te (5-10%)	0 = >20g/l	0 = none	0 = no puritis	10
2	Plac ebo	RVB 30	2	0 = normal	0 = normal	0 = normal	0 = normal	1 = slightly increased (2-3 x per day)	0 = None	0 = >20g/l	0 = none	0 = no puritis	1

2	Placebo	RVB 31	1	0 = normal	0 = normal	0 = normal	2 = very soft faeces	1 = slightly increased (2-3 x per day)	0 = None	0 = >20g/l	0 = none	1 = occasional episodes of itching	4
2	Placebo	RVB 31	2	0 = normal	0 = normal	0 = normal	0 = normal	0 = normal	0 = None	0 = >20g/l	0 = none	1 = occasional episodes of itching	1
2	Placebo	RVB 31	3	0 = normal	0 = normal	0 = normal	0 = normal	0 = normal	0 = None	0 = >20g/l	0 = none	1 = occasional episodes of itching	1
2	Placebo	RVB 31	7	0 = normal	0 = normal	0 = normal	0 = normal	0 = normal	0 = None	0 = >20g/l	0 = none	0 = no puritis	0
2	Placebo	RVB 34	1	0 = normal	0 = normal	3 = severe (>3x per week)	0 = normal	0 = normal	1 = mild (<5%)	0 = >20g/l	0 = none	0 = no puritis	4
2	Placebo	RVB 34	2	0 = normal	0 = normal	1 = mild (up to once a week)	0 = normal	1 = slightly increased (2-3 x per day)	1 = mild (<5%)	0 = >20g/l	0 = none	0 = no puritis	3
2	Placebo	RVB 34	3	0 = normal	0 = normal	1 = mild (up to once a week)	0 = normal	1 = slightly increased (2-3 x per day)	0 = None	0 = >20g/l	0 = none	0 = no puritis	2
2	Placebo	RVB 34	7	0 = normal	0 = normal	0 = normal	0 = normal	1 = slightly increased (2-3 x per day)	0 = None	0 = >20g/l	0 = none	0 = no puritis	1

TABLE A23. CIBDAI scores.

Group	Treatment	Patient No.	Visit No.	Attitude/Activity Score	Appetite Score	Vomiting Score	Stool Consistency Score	Stool Frequency Score	Weight Loss Score	CIBDAI Total
1	BIOEC 2015	RVB0 1	1	0 = normal	0 = normal	0 = normal	1 = slightly soft faeces or faecal blood, mucus or both	1 = slightly increased (2-3 x per day)	0 = None	2
1	BIOEC 2015	RVB0 1	2	1 = slightly decreased	0 = normal	1 = mild (up to once a week)	0 = normal	0 = normal	0 = None	2
1	BIOEC 2015	RVB0 1	3	0 = normal	0 = normal	0 = normal	1 = slightly soft faeces or faecal blood, mucus or both	1 = slightly increased (2-3 x per day)	1 = mild (<5%)	3
1	BIOEC 2015	RVB0 1	4	0 = normal	0 = normal	0 = normal	0 = normal	0 = normal	0 = None	0
1	BIOEC 2015	RVB0 1	5	0 = normal	0 = normal	0 = normal	1 = slightly soft faeces or faecal blood, mucus or both	0 = normal	0 = None	1
1	BIOEC 2015	RVB0 1	6	0 = normal	0 = normal	0 = normal	0 = normal	0 = normal	0 = None	0
1	BIOEC 2015	RVB0 1	7	0 = normal	0 = normal	0 = normal	0 = normal	1 = slightly increased (2-3 x per day)	0 = None	1
1	BIOEC 2015	RVB0 7	1	0 = normal	0 = normal	0 = normal	4 = watery diarrhoea	1 = slightly increased (2-3 x per day)	1 = mild (<5%)	6
1	BIOEC 2015	RVB0 7	2	0 = normal	0 = normal	0 = normal	4 = watery diarrhoea	0 = normal	1 = mild (<5%)	5
1	BIOEC 2015	RVB0 8	1	0 = normal	0 = normal	0 = normal	2 = very soft faeces	3 = severely increased (>5 x per day)	2 = moderate (5-10%)	7
1	BIOEC 2015	RVB0 8	2	0 = normal	2 = moderately decreased	0 = normal	0 = normal	1 = slightly increased (2-3 x per day)	0 = None	3
1	BIOEC 2015	RVB0 8	4	0 = normal	0 = normal	0 = normal	1 = slightly soft faeces or faecal blood, mucus or both	0 = normal	0 = None	1
1	BIOEC 2015	RVB0 8	5	0 = normal	0 = normal	0 = normal	0 = normal	0 = normal	0 = None	0
1	BIOEC 2015	RVB0 8	6	0 = normal	0 = normal	0 = normal	0 = normal	0 = normal	1 = mild (<5%)	1

1	BIOEC 2015	RVB0 8	7	0 = normal	0 = normal	0 = normal	0 = normal	1 = slightly increased (2-3 x per day)	0 = None	1
1	BIOEC 2015	RVB0 8	3	0 = normal	1 = slightly decreased	0 = normal	1 = slightly soft faeces or faecal blood, mucus or both	1 = slightly increased (2-3 x per day)	0 = None	3
1	BIOEC 2015	RVB1 3	1	0 = normal	0 = normal	3 = severe (>3x per week)	0 = normal	0 = normal	1 = mild (<5%)	4
1	BIOEC 2015	RVB1 3	3	0 = normal	0 = normal	1 = mild (up to once a week)	0 = normal	0 = normal	0 = None	1
1	BIOEC 2015	RVB1 3	4	0 = normal	0 = normal	0 = normal	0 = normal	0 = normal	1 = mild (<5%)	1
1	BIOEC 2015	RVB1 3	5	0 = normal	0 = normal	1 = mild (up to once a week)	2 = very soft faeces	0 = normal	1 = mild (<5%)	4
1	BIOEC 2015	RVB1 3	6	0 = normal	0 = normal	0 = normal	0 = normal	0 = normal	0 = None	0
1	BIOEC 2015	RVB1 3	7	0 = normal	0 = normal	0 = normal	0 = normal	1 = slightly increased (2-3 x per day)	0 = None	1
1	BIOEC 2015	RVB1 3	2	0 = normal	0 = normal	0 = normal	0 = normal	0 = normal	0 = None	0
1	BIOEC 2015	RVB1 6	1	1 = slightly decreased	0 = normal	2 = moderate (1-3 times per week)	1 = slightly soft faeces or faecal blood, mucus or both	2 = moderately increased (4-5 x per day)	0 = None	6
1	BIOEC 2015	RVB1 6	2	1 = slightly decreased	1 = slightly decreased	2 = moderate (1-3 times per week)	1 = slightly soft faeces or faecal blood, mucus or both	1 = slightly increased (2-3 x per day)	2 = moderate (5-10%)	8
1	BIOEC 2015	RVB1 6	3	1 = slightly decreased	1 = slightly decreased	2 = moderate (1-3 times per week)	1 = slightly soft faeces or faecal blood, mucus or both	1 = slightly increased (2-3 x per day)	1 = mild (<5%)	7
1	BIOEC 2015	RVB1 6	4	1 = slightly decreased	0 = normal	2 = moderate (1-3 times per week)	0 = normal	1 = slightly increased (2-3 x per day)	0 = None	4
1	BIOEC 2015	RVB1 6	5	0 = normal	0 = normal	0 = normal	0 = normal	0 = normal	0 = None	0
1	BIOEC 2015	RVB1 6	6	0 = normal	0 = normal	0 = normal	0 = normal	3 = severely increased (>5 x per day)	0 = None	3
1	BIOEC 2015	RVB1 6	7	0 = normal	0 = normal	0 = normal	0 = normal	2 = moderately increased (4-5 x per day)	0 = None	2

1	BIOEC 2015	RVB1 8	1	1 = slightly decreased	1 = slightly decreased	3 = severe (>3x per week)	0 = normal	1 = slightly increased (2-3 x per day)	0 = None	6
1	BIOEC 2015	RVB1 8	2	0 = normal	0 = normal	0 = normal	0 = normal	0 = normal	0 = None	0
1	BIOEC 2015	RVB1 8	3	0 = normal	0 = normal	1 = mild (up to once a week)	0 = normal	0 = normal	0 = None	1
1	BIOEC 2015	RVB1 8	4	2 = moderately decreased	1 = slightly decreased	2 = moderate (1-3 times per week)	1 = slightly soft faeces or faecal blood, mucus or both	0 = normal	0 = None	6
1	BIOEC 2015	RVB1 8	5	0 = normal	0 = normal	1 = mild (up to once a week)	0 = normal	0 = normal	0 = None	1
1	BIOEC 2015	RVB1 8	6	0 = normal	0 = normal	0 = normal	0 = normal	1 = slightly increased (2-3 x per day)	0 = None	1
1	BIOEC 2015	RVB1 8	7	0 = normal	0 = normal	0 = normal	0 = normal	1 = slightly increased (2-3 x per day)	0 = None	1
1	BIOEC 2015	RVB2 7	1	0 = normal	0 = normal	2 = moderate (1-3 times per week)	1 = slightly soft faeces or faecal blood, mucus or both	1 = slightly increased (2-3 x per day)	1 = mild (<5%)	5
1	BIOEC 2015	RVB2 9	1	0 = normal	0 = normal	2 = moderate (1-3 times per week)	0 = normal	0 = normal	1 = mild (<5%)	3
1	BIOEC 2015	RVB2 9	2	0 = normal	1 = slightly decreased	0 = normal	0 = normal	0 = normal	1 = mild (<5%)	2
1	BIOEC 2015	RVB2 9	3	0 = normal	0 = normal	0 = normal	0 = normal	0 = normal	0 = None	0
1	BIOEC 2015	RVB2 9	4	0 = normal	0 = normal	0 = normal	0 = normal	0 = normal	0 = None	0
1	BIOEC 2015	RVB2 9	5	0 = normal	0 = normal	0 = normal	0 = normal	0 = normal	0 = None	0
1	BIOEC 2015	RVB2 9	6	0 = normal	1 = slightly decreased	0 = normal	1 = slightly soft faeces or faecal blood, mucus or both	1 = slightly increased (2-3 x per day)	0 = None	3
1	BIOEC 2015	RVB2 9	7	0 = normal	1 = slightly decreased	0 = normal	0 = normal	0 = normal	0 = None	1
1	BIOEC 2015	RVB3 2	1	0 = normal	0 = normal	1 = mild (up to once a week)	1 = slightly soft faeces or faecal blood, mucus or both	1 = slightly increased (2-3 x per day)	2 = moderate (5-10%)	5

1	BIOEC 2015	RVB3 2	2	0 = normal	0 = normal	0 = normal	0 = normal	1 = slightly increased (2-3 x per day)	0 = None	1
1	BIOEC 2015	RVB3 2	3	0 = normal	0 = normal	0 = normal	0 = normal	1 = slightly increased (2-3 x per day)	0 = None	1
1	BIOEC 2015	RVB3 2	4	0 = normal	0 = normal	0 = normal	0 = normal	1 = slightly increased (2-3 x per day)	0 = None	1
1	BIOEC 2015	RVB3 2	5	0 = normal	0 = normal	0 = normal	0 = normal	1 = slightly increased (2-3 x per day)	0 = None	1
1	BIOEC 2015	RVB3 2	6	0 = normal	0 = normal	0 = normal	0 = normal	1 = slightly increased (2-3 x per day)	0 = None	1
1	BIOEC 2015	RVB3 2	7	0 = normal	0 = normal	0 = normal	0 = normal	1 = slightly increased (2-3 x per day)	0 = None	1
1	BIOEC 2015	RVB3 5	1	1 = slightly decreased	0 = normal	0 = normal	1 = slightly soft faeces or faecal blood, mucus or both	1 = slightly increased (2-3 x per day)	2 = moderate (5-10%)	5
1	BIOEC 2015	RVB3 5	2	0 = normal	0 = normal	0 = normal	0 = normal	1 = slightly increased (2-3 x per day)	2 = moderate (5-10%)	3
1	BIOEC 2015	RVB3 5	3	0 = normal	0 = normal	0 = normal	0 = normal	1 = slightly increased (2-3 x per day)	1 = mild (<5%)	2
1	BIOEC 2015	RVB3 5	4	0 = normal	0 = normal	0 = normal	0 = normal	0 = normal	0 = None	0
1	BIOEC 2015	RVB3 5	5	0 = normal	0 = normal	0 = normal	0 = normal	0 = normal	0 = None	0
1	BIOEC 2015	RVB3 5	6	0 = normal	0 = normal	0 = normal	0 = normal	0 = normal	0 = None	0
1	BIOEC 2015	RVB3 5	7	0 = normal	0 = normal	0 = normal	0 = normal	1 = slightly increased (2-3 x per day)	0 = None	1
2	Place bo	RVB0 2	1	1 = slightly decreased	1 = slightly decreased	1 = mild (up to once a week)	1 = slightly soft faeces or faecal blood, mucus or both	1 = slightly increased (2-3 x per day)	0 = None	5
2	Place bo	RVB0 2	2	0 = normal	0 = normal	0 = normal	0 = normal	0 = normal	1 = mild (<5%)	1
2	Place bo	RVB0 2	3	0 = normal	0 = normal	1 = mild (up to once a week)	0 = normal	1 = slightly increased (2-3 x per day)	0 = None	2
2	Place bo	RVB0 2	4	0 = normal	0 = normal	0 = normal	0 = normal	0 = normal	1 = mild (<5%)	1

2	Place bo	RVB0 2	5	0 = normal	0 = normal	0 = normal	0 = normal	0 = normal	0 = None	0
2	Place bo	RVB0 2	6	0 = normal	0 = normal	0 = normal	0 = normal	0 = normal	0 = None	0
2	Place bo	RVB0 2	7	0 = normal	0 = normal	0 = normal	0 = normal	0 = normal	0 = None	0
2	Place bo	RVB0 5	1	0 = normal	0 = normal	0 = normal	4 = watery diarrhoea	2 = moderately increased (4-5 x per day)	2 = moderate (5-10%)	8
2	Place bo	RVB0 9	1	0 = normal	0 = normal	0 = normal	4 = watery diarrhoea	1 = slightly increased (2-3 x per day)	0 = None	5
2	Place bo	RVB0 9	2	0 = normal	0 = normal	0 = normal	4 = watery diarrhoea	0 = normal	0 = None	4
2	Place bo	RVB0 9	3	0 = normal	1 = slightly decreased	0 = normal	4 = watery diarrhoea	0 = normal	3 = severe (>10%)	8
2	Place bo	RVB1 1	1	0 = normal	0 = normal	1 = mild (up to once a week)	4 = watery diarrhoea	3 = severely increased (>5 x per day)	1 = mild (<5%)	9
2	Place bo	RVB1 1	2	0 = normal	0 = normal	0 = normal	0 = normal	2 = moderately increased (4-5 x per day)	0 = None	2
2	Place bo	RVB1 1	3	0 = normal	0 = normal	1 = mild (up to once a week)	0 = normal	3 = severely increased (>5 x per day)	0 = None	4
2	Place bo	RVB1 1	4	0 = normal	0 = normal	0 = normal	0 = normal	3 = severely increased (>5 x per day)	0 = None	3
2	Place bo	RVB1 1	5	0 = normal	0 = normal	0 = normal	1 = slightly soft faeces or faecal blood, mucus or both	3 = severely increased (>5 x per day)	1 = mild (<5%)	5
2	Place bo	RVB1 1	6	0 = normal	0 = normal	0 = normal	0 = normal	3 = severely increased (>5 x per day)	0 = None	3
2	Place bo	RVB1 1	7	0 = normal	0 = normal	0 = normal	3 = severe	3 = severely increased (>5 x per day)	0 = None	6
2	Place bo	RVB1 2	1	0 = normal	0 = normal	1 = mild (up to once a week)	0 = normal	0 = normal	0 = None	1
2	Place bo	RVB1 2	2	0 = normal	0 = normal	0 = normal	0 = normal	0 = normal	0 = None	0
2	Place bo	RVB1 2	3	0 = normal	1 = slightly decreased	1 = mild (up to once a week)	3 = severe	0 = normal	1 = mild (<5%)	6

2	Placebo	RVB20	1	0 = normal	0 = normal	0 = normal	3 = severe	1 = slightly increased (2-3 x per day)	1 = mild (<5%)	5
2	Placebo	RVB20	2	0 = normal	0 = normal	1 = mild (up to once a week)	2 = very soft faeces	0 = normal	0 = None	3
2	Placebo	RVB20	3	0 = normal	0 = normal	0 = normal	0 = normal	2 = moderately increased (4-5 x per day)	0 = None	2
2	Placebo	RVB20	4	1 = slightly decreased	0 = normal	0 = normal	1 = slightly soft faeces or faecal blood, mucus or both	1 = slightly increased (2-3 x per day)	0 = None	3
2	Placebo	RVB20	5	0 = normal	0 = normal	0 = normal	0 = normal	1 = slightly increased (2-3 x per day)	0 = None	1
2	Placebo	RVB20	6	0 = normal	0 = normal	1 = mild (up to once a week)	0 = normal	1 = slightly increased (2-3 x per day)	0 = None	2
2	Placebo	RVB20	7	0 = normal	0 = normal	0 = normal	0 = normal	2 = moderately increased (4-5 x per day)	0 = None	2
2	Placebo	RVB28	1	1 = slightly decreased	0 = normal	0 = normal	4 = watery diarrhoea	2 = moderately increased (4-5 x per day)	0 = None	7
2	Placebo	RVB28	2	0 = normal	0 = normal	0 = normal	2 = very soft faeces	1 = slightly increased (2-3 x per day)	1 = mild (<5%)	4
2	Placebo	RVB30	1	2 = moderately decreased	3 = severely decreased	0 = normal	3 = severe	0 = normal	2 = moderate (5-10%)	10
2	Placebo	RVB30	2	0 = normal	0 = normal	0 = normal	0 = normal	1 = slightly increased (2-3 x per day)	0 = None	1
2	Placebo	RVB31	1	0 = normal	0 = normal	0 = normal	2 = very soft faeces	1 = slightly increased (2-3 x per day)	0 = None	3
2	Placebo	RVB31	2	0 = normal	0 = normal	0 = normal	0 = normal	0 = normal	0 = None	0
2	Placebo	RVB31	3	0 = normal	0 = normal	0 = normal	0 = normal	0 = normal	0 = None	0
2	Placebo	RVB31	4	1 = slightly decreased	0 = normal	0 = normal	0 = normal	0 = normal	0 = None	1

2	Placebo	RVB31	5	0 = normal	1 = slightly decreased	0 = normal	2 = very soft faeces	1 = slightly increased (2-3 x per day)	0 = None	4
2	Placebo	RVB31	6	0 = normal	0 = normal	0 = normal	0 = normal	0 = normal	0 = None	0
2	Placebo	RVB31	7	0 = normal	0 = normal	0 = normal	0 = normal	0 = normal	0 = None	0
2	Placebo	RVB34	1	0 = normal	0 = normal	3 = severe (>3x per week)	0 = normal	0 = normal	1 = mild (<5%)	4
2	Placebo	RVB34	2	0 = normal	0 = normal	1 = mild (up to once a week)	0 = normal	1 = slightly increased (2-3 x per day)	1 = mild (<5%)	3
2	Placebo	RVB34	3	0 = normal	0 = normal	1 = mild (up to once a week)	0 = normal	1 = slightly increased (2-3 x per day)	0 = None	2
2	Placebo	RVB34	4	0 = normal	0 = normal	1 = mild (up to once a week)	0 = normal	1 = slightly increased (2-3 x per day)	0 = None	2
2	Placebo	RVB34	5	0 = normal	0 = normal	0 = normal	0 = normal	1 = slightly increased (2-3 x per day)	0 = None	1
2	Placebo	RVB34	6	0 = normal	0 = normal	0 = normal	0 = normal	1 = slightly increased (2-3 x per day)	0 = None	1
2	Placebo	RVB34	7	0 = normal	0 = normal	0 = normal	0 = normal	1 = slightly increased (2-3 x per day)	0 = None	1

TABLE A24. WSAVA scores, stomach fundus.

Group	Treatment	Patient No.	Visit No.	Surface Epithelial Injury	Gastric Pit Epithelial Injury	Fibrosis/Glandular Nesting/Mucosal Atrophy	Intraepithelial Lymphocytes	Lamina Propria Lymphocytes and Plasma Cells	Lamina Propria Eosinophils	Lamina Propria Neutrophils	Gastric Lymphofollicular Hyperplasia	Subtotal
1	BIOEC2015	RVB01	1	0	0	1	0	0	0	0	0	1
1	BIOEC2015	RVB01	3									
1	BIOEC2015	RVB07	1	0	0	1	0	1	0	0	0	2
1	BIOEC2015	RVB08	1	0	1	1	0	0	0	0	0	2
1	BIOEC2015	RVB08	3	0	0	1	0	0	0	0	0	1
1	BIOEC2015	RVB13	1									
1	BIOEC2015	RVB13	3	1	2	2	2	3	0	0	2	12
1	BIOEC2015	RVB16	1	0	2	2	0	2	0	0	0	6
1	BIOEC2015	RVB16	3	0	0	1	0	1	0	0	0	2
1	BIOEC2015	RVB18	1	0	1	2	0	1	0	0	1	5
1	BIOEC2015	RVB18	3	0	2	2	0	2	0	0	1	7
1	BIOEC2015	RVB27	1	0	0	1	0	0	0	0	0	1
1	BIOEC2015	RVB29	1	1	0	1	0	0	0	0	0	2

1	BIOEC2015	RVB32	1	0	0	1	0	0	0	0	0	1
1	BIOEC2015	RVB32	3									
1	BIOEC2015	RVB35	1	0	0	1	0	0	0	0	0	1
1	BIOEC2015	RVB35	3	0	1	1	0	0	0	0	0	2
2	Placebo	RVB02	1	0	0	2	0	0	0	0	0	2
2	Placebo	RVB02	3									
2	Placebo	RVB05	1	0	2	2	0	2	0	0	1	7
2	Placebo	RVB09	1	0	0	1	0	0	0	0	0	1
2	Placebo	RVB11	1	0	1	2	0	0	0	0	0	3
2	Placebo	RVB11	3	0	0	1	0	0	0	0	1	2
2	Placebo	RVB12	1	1	2	2	1	2	0	1	0	9
2	Placebo	RVB20	1	0	0	1	0	0	0	0	0	1
2	Placebo	RVB20	3	0	0	2	0	0	0	0	1	3
2	Placebo	RVB28	1	0	0	0	0	0	0	0	0	0
2	Placebo	RVB30	1	0	0	0	0	0	0	0	1	1
2	Placebo	RVB31	1	0	0	0	0	0	0	0	0	0
2	Placebo	RVB34	1	0	0	3	0	0	0	0	1	4
2	Placebo	RVB34	3									

TABLE A25. WSAVA scores – Stomach antrum.

Group	Treatment	Patient No.	Visit No.	Epithelial Injury	Epithelial Hyperplasia	Mucosal Fibrosis Glandular Atrophy	Intraepithelial Lymphocytes	Lamina Propria Lymphocytes and Plasma Cells	Lamina Propria Eosinophils	Lamina Propria Neutrophils	Gastric Lymphofollicular Hyperplasia	Subtotal
1	BIOEC2015	RVB01	1	0	0	0	0	0	0	0	0	0
1	BIOEC2015	RVB01	3									
1	BIOEC2015	RVB07	1	0	0	1	0	0	0	0	0	1
1	BIOEC2015	RVB08	1	0	0	1	0	0	0	0	0	1
1	BIOEC2015	RVB08	3	0	1	2	0	1	0	0	1	5
1	BIOEC2015	RVB13	1	2	3	3	1	3	0	0	3	15
1	BIOEC2015	RVB13	3	1	1	3	3	2	0	0	2	12
1	BIOEC2015	RVB16	1	0	1	2	0	1	0	0	0	4
1	BIOEC2015	RVB16	3	0	0	0	0	0	0	0	0	0
1	BIOEC2015	RVB18	1	0	1	1	0	0	0	0	0	2
1	BIOEC2015	RVB18	3	0	1	1	0	0	0	0	0	2
1	BIOEC2015	RVB27	1	0	0	0	0	0	0	0	0	0
1	BIOEC2015	RVB29	1	0	0	0	0	0	0	0	0	0
1	BIOEC2015	RVB32	1	0	0	0	0	0	0	0	1	1
1	BIOEC2015	RVB32	3									
1	BIOEC2015	RVB35	1	0	0	0	0	0	0	0	0	0
1	BIOEC2015	RVB35	3	0	0	0	0	0	0	0	1	1
2	Placebo	RVB02	1	0	1	2	0	0	0	0	0	3
2	Placebo	RVB02	3									
2	Placebo	RVB05	1	0	0	1	0	0	0	0	0	1
2	Placebo	RVB09	1	0	0	1	0	0	0	0	0	1
2	Placebo	RVB11	1	0	1	2	0	0	0	0	1	4
2	Placebo	RVB11	3	0	0	0	0	0	0	0	0	0
2	Placebo	RVB12	1	1	0	2	1	2	0	0	0	6
2	Placebo	RVB20	1	0	0	0	0	0	0	0	1	1

2	Placebo	RVB20	3	0	0	0	0	0	0	0	0	0
2	Placebo	RVB28	1	0	0	1	0	0	0	0	0	1
2	Placebo	RVB30	1	0	0	0	0	0	0	0	0	0
2	Placebo	RVB31	1	0	0	1	0	0	0	0	0	1
2	Placebo	RVB34	1	0	0	2	0	0	0	0	2	4
2	Placebo	RVB34	3	0	1	1	0	1	0	1	0	4

TABLE A26. WSAVA scores – duodenum.

Group	Treatment	Patient No.	Visit No.	Villous Stunting	Villous Epithelial Injury	Crypt Distension	Lacteal Dilation	Mucosal Fibrosis	Intraepithelial Lymphocytes	Lamia Propria Lymphocytes and Plasma Cells	Lamia Propria Eosinophils	Lamia Propria Neutrophils	Subtotal
1	BIOEC2015	RVB01	1	1	0	0	0	0	0	1	0	0	2
1	BIOEC2015	RVB01	3	1	0	0	1	1	0	1	0	0	4
1	BIOEC2015	RVB07	1	2	2	2	2	0	1	3	0	0	12
1	BIOEC2015	RVB08	1	1	0	1	1	0	0	1	0	0	4
1	BIOEC2015	RVB08	3	2	1	1	0	1	0	1	0	0	6
1	BIOEC2015	RVB13	1	1	0	0	0	1	0	2	1	0	5
1	BIOEC2015	RVB13	3	1	0	1	0	0	0	1	0	0	3
1	BIOEC2015	RVB16	1	2	0	0	0	0	0	2	0	0	4
1	BIOEC2015	RVB16	3	2	1	1	0	0	0	2	0	0	6
1	BIOEC2015	RVB18	1	1	0	0	0	0	0	2	1	0	4
1	BIOEC2015	RVB18	3	0	1	0	0	0	0	1	1	0	3
1	BIOEC2015	RVB27	1	1	0	0	0	0	0	1	0	0	2
1	BIOEC2015	RVB29	1	0	0	0	0	0	0	2	0	0	2
1	BIOEC2015	RVB32	1	1	0	0	0	0	0	1	1	0	3
1	BIOEC2015	RVB32	3	0	0	0	0	0	0	1	0	0	1
1	BIOEC2015	RVB35	1	2	1	2	1	0	0	3	0	0	9
1	BIOEC2015	RVB35	3	2	0	0	0	0	0	1	0	0	3
2	Placebo	RVB02	1	1	0	0	1	0	0	1	0	0	3
2	Placebo	RVB02	3	1	0	0	0	0	0	1	0	0	2
2	Placebo	RVB05	1	2	2	1	2	1	0	2	0	0	10
2	Placebo	RVB09	1	3	1	2	1	1	0	2	0	0	10
2	Placebo	RVB11	1	3	2	1	0	0	1	2	2	0	11
2	Placebo	RVB11	3	0	0	0	0	0	0	1	0	0	1
2	Placebo	RVB12	1	1	0	0	0	0	0	0	0	0	1
2	Placebo	RVB20	1	1	0	0	1	0	0	1	0	0	3
2	Placebo	RVB20	3	0	0	0	0	0	0	0	0	0	0
2	Placebo	RVB28	1	2	0	0	0	0	1	2	0	0	5

2	Placebo	RVB30	1	0	0	1	0	1	0	1	0	1	4
2	Placebo	RVB31	1	1	0	0	1	0	0	2	0	0	4
2	Placebo	RVB34	1	1	1	1	0	0	0	1	0	0	4
2	Placebo	RVB34	3	1	0	0	0	0	0	1	0	0	2

TABLE A27. WSAVA score - ilium.

Group	Treatment	Patient No.	Visit No.	Villous Stunting	Villous Epithelial Injury	Crypt Distension	Lacteal Dilation	Mucosal Fibrosis	Intraepithelial Lymphocytes	Lamia Propria Lymphocytes and Plasma Cells	Lamia Propria Eosinophils	Lamia Propria Neutrophils	Subtotal
1	BIOEC2015	RVB01	1										
1	BIOEC2015	RVB01	3										
1	BIOEC2015	RVB07	1	0	0	0	2	0	1	2	0	0	5
1	BIOEC2015	RVB08	1	1	0	0	1	0	0	1	1	0	4
1	BIOEC2015	RVB08	3										
1	BIOEC2015	RVB13	1	0	0	0	0	0	0	1	1	0	2
1	BIOEC2015	RVB13	3	0	0	0	1	0	0	2	0	0	3
1	BIOEC2015	RVB16	1	0	0	0	0	0	1	1	0	1	3
1	BIOEC2015	RVB16	3	0	0	0	0	0	0	0	0	0	0
1	BIOEC2015	RVB18	1	0	0	0	1	0	0	1	2	0	4
1	BIOEC2015	RVB18	3	0	0	0	0	0	1	1	0	0	2
1	BIOEC2015	RVB27	1										
1	BIOEC2015	RVB29	1										
1	BIOEC2015	RVB32	1	0	0	0	0	0	0	0	0	0	0
1	BIOEC2015	RVB32	3										
1	BIOEC2015	RVB35	1										
1	BIOEC2015	RVB35	3										
2	Placebo	RVB02	1	0	0	1	1	0	0	1	0	0	3
2	Placebo	RVB02	3	0	0	0	0	0	1	0	0	0	1
2	Placebo	RVB05	1	1	0	0	3	0	0	1	0	0	5
2	Placebo	RVB09	1	2	0	0	0	0	3	3	0	0	8
2	Placebo	RVB11	1	1	0	0	1	0	0	1	1	1	5
2	Placebo	RVB11	3	0	0	0	1	0	1	1	1	0	4
2	Placebo	RVB12	1	0	0	0	0	0	0	1	1	0	2
2	Placebo	RVB20	1	0	0	0	0	0	0	0	1	0	1
2	Placebo	RVB20	3										
2	Placebo	RVB28	1										

2	Placebo	RVB30	1										
2	Placebo	RVB31	1										
2	Placebo	RVB34	1										
2	Placebo	RVB34	3	1	2	0	0	0	0	1	0	1	5

TABLE A28. WSAVA score – colon

Group	Treatment	Patient No.	Visit No.	Surface Epithelial Injury	Crypt Hyperplasia	Crypt Dilation and Distortion	Mucosal Fibrosis and Atrophy	Lamina Propria Lymphocytes and Plasma Cells	Lamina Propria Eosinophils	Lamina Propria Neutrophils	Lamina Propria Macrophages	SubTotal
1	BIOEC2015	RVB01	1	0	0	0	0	1	0	0	0	1
1	BIOEC2015	RVB01	3	1	1	2	1	1	0	0	0	6
1	BIOEC2015	RVB07	1	1	1	1	0	1	0	0	0	4
1	BIOEC2015	RVB08	1	0	1	2	1	1	0	0	0	5
1	BIOEC2015	RVB08	3	1	1	1	1	0	0	0	0	4
1	BIOEC2015	RVB13	1	1	0	1	1	1	0	0	0	4
1	BIOEC2015	RVB13	3	0	0	1	1	1	0	0	0	3
1	BIOEC2015	RVB16	1	0	1	1	1	2	0	0	0	5
1	BIOEC2015	RVB16	3	0	0	1	1	1	0	0	0	3
1	BIOEC2015	RVB18	1	3	0	1	1	2	3	1	1	12
1	BIOEC2015	RVB18	3	3	1	2	2	2	3	1	1	15
1	BIOEC2015	RVB27	1	1	0	1	1	1	0	0	0	4
1	BIOEC2015	RVB29	1	0	0	0	1	0	0	0	0	1
1	BIOEC2015	RVB32	1	1	1	2	1	2	1	0	0	8
1	BIOEC2015	RVB32	3	0	1	0	0	1	0	0	0	2
1	BIOEC2015	RVB35	1	0	0	1	0	2	0	0	0	3
1	BIOEC2015	RVB35	3	0	0	0	0	1	0	0	0	1
2	Placebo	RVB02	1	1	1	1	0	2	1	0	0	6
2	Placebo	RVB02	3	2	1	1	1	2	0	1	0	8
2	Placebo	RVB05	1	1	1	1	0	1	0	0	0	4
2	Placebo	RVB09	1	0	0	0	0	1	0	0	0	1
2	Placebo	RVB11	1	0	0	1	1	1	0	1	0	4
2	Placebo	RVB11	3	0	0	1	0	1	0	0	0	2
2	Placebo	RVB12	1	1	0	1	0	1	0	0	0	3
2	Placebo	RVB20	1	0	0	0	0	1	0	0	0	1
2	Placebo	RVB20	3	1	1	1	0	2	0	0	0	5
2	Placebo	RVB28	1	1	1	1	1	2	0	0	0	6

2	Placebo	RVB30	1	0	0	1	1	1	0	0	0	3
2	Placebo	RVB31	1	1	0	0	0	1	0	0	0	2
2	Placebo	RVB34	1	1	0	0	1	1	0	0	0	3
2	Placebo	RVB34	3	0	0	1	1	1	0	0	0	3

TABLE A29. UGI Part 1 Equipment used and sampling type.

Group	Treatment	Patient No.	Visit No.	Endoscopes Used	Forceps Used	Problems/Complications	Sampling	Documentation
1	BIOEC2015	RVB01	1	GIF-H260	EndoJaw 1550m Model FB-230K	None	biopsy	None
1	BIOEC2015	RVB01	3	GIF-H260	EndoJaw 1550mm Model FB-230K	None	biopsy	Photo
1	BIOEC2015	RVB07	1	GIFH260	Disposable 1550mm 2.8mm	None	biopsy	Photo
1	BIOEC2015	RVB08	1	CF H260 AL	Endojaw FB-2300mm	None	biopsy	Video, photo
1	BIOEC2015	RVB08	3	CF H260 AL	EndoJaw FB-230U 2300mm	None	biopsy	Photo
1	BIOEC2015	RVB13	1	CFH260 AL	EndoJaw 2300mm	None	biopsy	Photo
1	BIOEC2015	RVB13	3	CFH260 AL	FB-230U EndoJaw	None	biopsy	Photo
1	BIOEC2015	RVB16	1	GIF-H260	EndoJaw FB-230K	None	biopsy	Photo
1	BIOEC2015	RVB16	3	CIF-H260 AL	EndoJaw FB-230U	None	biopsy	Photo
1	BIOEC2015	RVB18	1	GIF-H260	FB-230K EndoJaw	None	biopsy	Photo
1	BIOEC2015	RVB18	3	GIF-H260	EndoJaw FB 230K	None	biopsy	Photo
1	BIOEC2015	RVB27	1	CFH260AL	2.8mm / 2300mm	None	biopsy	Photo
1	BIOEC2015	RVB29	1	CF-H260AL	EndoJaw FB-230U Oval Fenestrated	None	biopsy	Photo
1	BIOEC2015	RVB29	3	CF-H260AL	EndoJaw FB-230U Oval Fenestrated	None	biopsy	Photo

1	BIOEC2015	RVB32	1	GIF H260	EndoJaw FB-230K Oval Fenestrated SwingJaw	None	biopsy	Photo
1	BIOEC2015	RVB32	3	CF-H260AL	EndoJaw FB230-U Oval Fenestrated Swing Jaw	None	biopsy	Photo
1	BIOEC2015	RVB35	1	Olympus CF-H260AL	EndoJaw Oval Fenestrated SwingJaw FB-230U	None	biopsy	Photo
1	BIOEC2015	RVB35	3	CF-H260 AL	EndoJaw FB-230U	None	biopsy	Photo
2	Placebo	RVB02	1	GIFXP6N	1550mm 2.0mm Oval Fenestrated	None	biopsy	None
2	Placebo	RVB02	3	GIF-H260	1550mm 2.8mm	None	biopsy	Photo
2	Placebo	RVB05	1	GIF-H260	EndoJaw FB230K	None	biopsy	Photo
2	Placebo	RVB09	1	CFH 260	Biopsy Forceps length 1550mm, channel size 2.8mm and 2300mm	None	biopsy	Photo and video
2	Placebo	RVB11	1	CFH 260 AL	Endojaw FB-230u	Excessive Bleeding, Visualisation obscured	biopsy	Photo
2	Placebo	RVB11	3	CFH260-AL	EndoJaw FB-230u	None	biopsy	Photo
2	Placebo	RVB12	1	CFH260 AL 3.7mm Biopsy Channel	2.8mm/2300mm biopsy forceps	None	biopsy	Photo
2	Placebo	RVB20	1	CFH-260-AL	FB-230U EndoJaw	None	biopsy	None
2	Placebo	RVB20	3	CFH 260AL	EndoJaw FB-230	None	biopsy	Photo
2	Placebo	RVB28	1	2049408	Oval fenestrated swing jaw	None	biopsy	Photo

					1550mm length/2.8mm channel size, model FB-230K			
2	Placebo	RVB30	1	GIF-H260	EndoJaw FB-230U Swing Jaw Oval Fenestrated	None	biopsy	Photo
2	Placebo	RVB31	1	GIF-H260	EndoJaw FB-230U Swing Jaw Oval Fenestrated	None	biopsy	Photo
2	Placebo	RVB31	3	GIF-H260	EndoJaw FB-230K Swing Jaw Oval Fenestrated	None	biopsy	Photo
2	Placebo	RVB34	1	Olympus CF-H260AL	Olympus EndoJaw Oval Fenestrated SwingJaw FB-230U	None	biopsy	Photo
2	Placebo	RVB34	3	CF-H260AL	EndoJaw FB-230U	None	biopsy	Photo

TABLE A30. UGI Part 1 oesophageal lesion code.

Group	Treatment	Patient No.	Visit No.	Hyperaemia/Vascularity	Discolouration	Friability	Haemorrhage	Erosion/Ulcer	Contents (mucus/bile/food)	LES open /other lesions	Comments
1	BIOEC2015	RVB01	1	0	0	0	0	0	0	0	
1	BIOEC2015	RVB01	3	0	0	0	0	0	0	0	
1	BIOEC2015	RVB07	1	0	0	0	0	0	0	0	
1	BIOEC2015	RVB08	1	0	0	0	0	0	0	0	
1	BIOEC2015	RVB08	3	0	0	0	0	0	0	0	
1	BIOEC2015	RVB13	1	0	0	0	0	0	2	0	Liquid in Oesophagus
1	BIOEC2015	RVB13	3	0	0	0	0	0	0	0	
1	BIOEC2015	RVB16	1	0	0	0	0	0	0	0	
1	BIOEC2015	RVB16	3	0	0	0	0	0	0	0	
1	BIOEC2015	RVB18	1	0	0	0	0	0	0	0	
1	BIOEC2015	RVB18	3	0	0	0	0	0	0	0	
1	BIOEC2015	RVB27	1	0	0	0	0	0	0	0	
1	BIOEC2015	RVB29	1	0	0	0	0	0	0	0	
1	BIOEC2015	RVB29	3	0	0	0	0	0	0	0	
1	BIOEC2015	RVB32	1	1	0	0	0	0	0	0	Mild hyperaemia in one focal area of mid oesophagus, not necessarily of clinical significance
1	BIOEC2015	RVB32	3	0	0	0	0	0	0	0	
1	BIOEC2015	RVB35	1	0	0	0	0	0	0	0	
1	BIOEC2015	RVB35	3	0	0	0	0	0	0	0	

2	Placebo	RVB02	1	0	0	0	0	0	1	0	
2	Placebo	RVB02	3	0	0	0	0	0	0	0	
2	Placebo	RVB05	1	0	0	0	0	0	0	0	
2	Placebo	RVB09	1	0	0	0	0	1	0	0	
2	Placebo	RVB11	1	0	0	0	0	0	0	0	
2	Placebo	RVB11	3	0	0	0	0	0	0	0	
2	Placebo	RVB12	1	0	0	0	0	0	0	0	
2	Placebo	RVB20	1	0	0	0	0	0	0	0	
2	Placebo	RVB20	3	0	0	0	0	0	0	0	
2	Placebo	RVB28	1	0	0	0	0	0	0	0	
2	Placebo	RVB30	1	0	0	0	0	0	0	0	
2	Placebo	RVB31	1	0	0	0	0	0	0	0	
2	Placebo	RVB31	3	0	0	0	0	0	0	0	
2	Placebo	RVB34	1	0	0	0	0	0	0	0	
2	Placebo	RVB34	3	0	0	0	0	0	0	0	

TABLE A31. UGI part 1 Stomach lesion code.

Group	Treatment	Patient No.	Visit No.	Can't inflate lumen	Hyperaemia/Vascularity	Oedema	Discolouration	Friability	haemorrhage	Erosion/Ulcer	Contents	Passing scope through pylorus	Comments
1	BIOEC2015	RVB01	1	0	0	0	0	0	0	0	0	0	
1	BIOEC2015	RVB01	3	0	0	0	0	1	0	0	0	0	mild friability
1	BIOEC2015	RVB07	1	0	0	0	0	0	0	0	0	0	
1	BIOEC2015	RVB08	1	0	0	0	0	0	0	0	0	0	
1	BIOEC2015	RVB08	3	0	1	0	0	0	0	0	0	0	mild generalised hyperaemia
1	BIOEC2015	RVB13	1	0	0	0	1	0	0	1	0	0	Two small ulcers seen, one in fundus, one in body
1	BIOEC2015	RVB13	3	0	0	0	0	0	0	0	0	0	
1	BIOEC2015	RVB16	1	0	0	0	1	0	0	0	0	0	Patchy hyperaemia vs pale, diffuse
1	BIOEC2015	RVB16	3	0	2	0	1	0	0	0	0	0	Moderately hyperaemic: patchy distribution (discoloured)
1	BIOEC2015	RVB18	1	0	0	0	0	0	0	0	0	0	Small protruding polyp in fundus only
1	BIOEC2015	RVB18	3	0	1	0	0	0	0	0	0	0	diffusely erythematous
1	BIOEC2015	RVB27	1	0	1	0	1	0	0	0	0	0	
1	BIOEC2015	RVB29	1	0	0	0	2	2	1	1	0	0	Ulcerated area in greater curvature
1	BIOEC2015	RVB29	3	0	0	0	0	0	0	0	0	0	
1	BIOEC2015	RVB32	1	0	0	0	0	0	0	0	0	0	

1	BIOEC2015	RVB32	3	0	0	0	1	0	0	0	2	0	Diffusely inflamed, no specific lesion. Liquid ++ in stomach
1	BIOEC2015	RVB35	1	0	0	0	0	0	1	0	1	2	Grass present
1	BIOEC2015	RVB35	3	0	0	0	0	0	0	0	0	0	
2	Placebo	RVB02	1	0	0	0	0	0	0	0	1	0	
2	Placebo	RVB02	3	0	0	0	0	0	0	0	0	0	
2	Placebo	RVB05	1	0	0	0	0	0	0	0	1	0	small amount of bile and mucus in gastric lumen
2	Placebo	RVB09	1	0	0	0	0	0	0	0	0	0	
2	Placebo	RVB11	1	0	0	0	0	0	0	0	0	0	
2	Placebo	RVB11	3	0	0	0	0	0	0	0	0	0	
2	Placebo	RVB12	1	0	0	0	0	0	0	0	0	0	
2	Placebo	RVB20	1	0	0	0	0	0	0	0	0	0	
2	Placebo	RVB20	3	0	0	0	0	0	0	0	0	0	
2	Placebo	RVB28	1	0	1	0	0	2	0	0	0	0	
2	Placebo	RVB30	1	0	0	0	0	0	0	0	0	2	Difficult to pass scope through pylorus
2	Placebo	RVB31	1	0	0	0	0	0	0	0	0	0	Pyloric sphincter slightly open
2	Placebo	RVB31	3	0	0	0	0	0	0	0	0	0	
2	Placebo	RVB34	1	0	0	0	0	0	0	0	0	0	
2	Placebo	RVB34	3	0	0	0	0	0	0	0	0	0	

TABLE A32. UGI part 1- Duodenum lesion code.

Group	Treatment	Patient No.	Visit No.	Can't inflate lumen	Hyperaemia/Vascularity	Oedema	Discolouration	Friability	Texture	Haemorrhage	Erosion/Ulcer	Lacteal dilation	Contents (mucus/ bile/ food)	Comments
1	BIOEC2015	RVB01	1	0	0	0	0	0	0	1	0	0	1	bile
1	BIOEC2015	RVB01	3	0	1	1	0	1	0	0	0	0	1	mild changes
1	BIOEC2015	RVB07	1	1	2	2	3	3	2	1	0	2	0	
1	BIOEC2015	RVB08	1	0	1	0	1	1	0	1	0	0	0	
1	BIOEC2015	RVB08	3	0	1	0	0	2	0	0	0	0	0	Mild hyperaemia but moderate diffuse friability. Making tunnels within the mucosa with the forceps
1	BIOEC2015	RVB13	1	0	0	2	0	3	2	2	0	0	0	Generalised inflammation
1	BIOEC2015	RVB13	3	0	2	1	2	0	1	0	0	2	0	
1	BIOEC2015	RVB16	1	0	2	1	2	2	2	1	0	0	0	Hyperaemic discolouration. Edematus with cobblestone appearance. Friable, resulting in haemorrhage, no haemorrhage prior to sampling
1	BIOEC2015	RVB16	3	0	2	1	2	3	2	0	0	1	0	Duodenum very friable, discoloured with abnormal texture. Mild lacteal dilation.
1	BIOEC2015	RVB18	1	0	2	1	1	1	1	0	0	0	0	
1	BIOEC2015	RVB18	3	0	0	0	0	0	0	0	0	0	0	
1	BIOEC2015	RVB27	1	0	1	2	1	2	2	0	0	0	1	

1	BIOEC2015	RVB29	1	0	2	1	2	2	1	0	0	1	0	
1	BIOEC2015	RVB29	3	0	0	2	0	0	2	1	0	0	0	Oedematous cobblestoned appearance of duodenum with multiple pinpoint haemorrhages on visible villi
1	BIOEC2015	RVB32	1	0	1	0	0	0	0	0	0	0	0	Generalised hyperaemia of whole region of duodenum examined
1	BIOEC2015	RVB32	3	0	0	1	0	1	1	1	0	0	1	mucus
1	BIOEC2015	RVB35	1	0	2	2	2	2	2	2	0	2	0	Diffusely oedematous and inflamed. Lacteals very visible
1	BIOEC2015	RVB35	3	0	2	3	2	2	2	2	0	2	0	
2	Placebo	RVB02	1	0	0	0	0	0	0	0	0	0	0	
2	Placebo	RVB02	3	0	1	1	0	1	0	0	0	0	0	
2	Placebo	RVB05	1	0	2	2	0	2	0	1	0	2	0	
2	Placebo	RVB09	1	0	3	2	3	3	3	3	0	1	2	a lot of bile
2	Placebo	RVB11	1	1	2	1	2	2	1	3	0	0	0	Cobblestone appearance in duodenum
2	Placebo	RVB11	3	0	1	0	1	1	0	0	0	0	0	Frequent areas of inflammation
2	Placebo	RVB12	1	0	1	1	0	0	1	0	0	0	0	mild hyperaemia, mild oedema, mild cobblestone appearance
2	Placebo	RVB20	1	0	0	0	0	0	0	0	0	0	0	
2	Placebo	RVB20	3	0	0	0	0	0	0	0	0	0	0	
2	Placebo	RVB28	1	0	1	1	1	1	0	0	0	0	0	

2	Placebo	RVB30	1	0	0	0	0	0	0	0	0	1	0	
2	Placebo	RVB31	1	0	0	1	0	0	2	0	0	0	0	Able to see villi very well. Cobblestone appearance.
2	Placebo	RVB31	3	0	0	0	0	0	0	0	0	0	0	
2	Placebo	RVB34	1	0	2	2	1	3	2	0	0	0	1	
2	Placebo	RVB34	3	0	1	1	1	0	1	1	0	0	0	

TABLE A33. UGI part 2 stomach lesion code.

Group	Treatment	Patient No.	Visit No.	Stomach	Site of Lesion	Site of Biopsies	Can't inflate	Hyperemia/Va	Edema	Discolouration	Friability	haemorrhage	Erosion/Ulcer	Contents	Passing scope	Comments
1	BIOEC2015	RVB 01	1	Normal			0	0	0	0	0	0	0	0	0	
1	BIOEC2015	RVB 01	3	Normal			0	0	0	0	1	0	0	0	0	mild friability
1	BIOEC2015	RVB 07	1	Normal		Body, Antrum	0	0	0	0	0	0	0	0	0	
1	BIOEC2015	RVB 08	1	Normal		Fundus, Incisura, Pylorus	0	0	0	0	0	0	0	0	0	
1	BIOEC2015	RVB 08	3	Lesion			0	1	0	0	0	0	0	0	0	mild generalised hyperemia
1	BIOEC2015	RVB 13	1	Lesion	Fundus, body	Fundus, Body, Incisura, Antrum, Pylorus	0	0	0	1	0	0	1	0	0	Two small ulcers seen, one in fundus, one in body
1	BIOEC2015	RVB 13	3	Normal		Fundus, Incisura, Antrum	0	0	0	0	0	0	0	0	0	
1	BIOEC2015	RVB 16	1	Lesion	Fundus, Body, Incisura, Antrum, Pylorus	Fundus, Body, Incisura, Antrum, Pylorus	0	0	0	1	0	0	0	0	0	Patchy hyperaemia vs pale, diffuse
1	BIOEC2015	RVB 16	3	Lesion	Fundus, body	Fundus, Body, Incisura, Pylorus	0	2	0	1	0	0	0	0	0	Moderately hyperaemic:patchy distribution (discoloured)

1	BIOEC2015	RVB 18	1	Lesion	Fundus	Fundus, Body, Incisura, Antrum, Pylorus	0	0	0	0	0	0	0	0	0	Small protruding polyp in fundus only
1	BIOEC2015	RVB 18	3	Lesion	Body	Fundus, Body, Incisura, Pylorus	0	1	0	0	0	0	0	0	0	diffusely erythematous
1	BIOEC2015	RVB 27	1	Lesion		Fundus, Body, Incisura, Antrum, Pylorus	0	1	0	1	0	0	0	0	0	
1	BIOEC2015	RVB 29	1	Lesion	Body	Fundus, Body, Incisura, Antrum	0	0	0	2	2	1	1	0	0	Ulcerated area in greater curvature
1	BIOEC2015	RVB 29	3	Normal			0	0	0	0	0	0	0	0	0	
1	BIOEC2015	RVB 32	1	Normal		Body, Pylorus	0	0	0	0	0	0	0	0	0	
1	BIOEC2015	RVB 32	3	Lesion			0	0	0	1	0	0	0	2	0	Diffusely inflamed, no specific lesion. Liquid ++ in stomach
1	BIOEC2015	RVB 35	1	Lesion		Fundus, Incisura, Antrum, Pylorus	0	0	0	0	0	1	0	1	2	Grass present
1	BIOEC2015	RVB 35	3	Normal		Body, Incisura, Antrum, Pylorus	0	0	0	0	0	0	0	0	0	
2	Placebo	RVB 02	1	Normal		Fundus, Antrum, Pylorus	0	0	0	0	0	0	0	1	0	
2	Placebo	RVB 02	3	Normal			0	0	0	0	0	0	0	0	0	
2	Placebo	RVB 05	1	Lesion		Fundus, Body, Incisura, Pylorus	0	0	0	0	0	0	0	1	0	small amount of bile and mucus in gastric lumen
2	Placebo	RVB 09	1	Normal		Fundus, Antrum, Pylorus	0	0	0	0	0	0	0	0	0	
2	Placebo	RVB 11	1	Normal		Fundus, Body, Pylorus	0	0	0	0	0	0	0	0	0	

2	Placebo	RVB 11	3	Normal		Fundus, Body, Incisura, Antrum	0	0	0	0	0	0	0	0	0	
2	Placebo	RVB 12	1	Normal		Fundus, Body, Incisura, Antrum, Pylorus	0	0	0	0	0	0	0	0	0	
2	Placebo	RVB 20	1	Normal		Fundus, Incisura, Antrum	0	0	0	0	0	0	0	0	0	
2	Placebo	RVB 20	3	Normal		Fundus, Body, Incisura, Antrum	0	0	0	0	0	0	0	0	0	
2	Placebo	RVB 28	1	Lesion	Fundus, Body, Incisura Antrum, Pylorus	Fundus, Pylorus	0	1	0	0	2	0	0	0	0	
2	Placebo	RVB 30	1	Lesion		Body, Incisura, Antrum, Pylorus	0	0	0	0	0	0	0	0	2	Difficult to pass scope through pylorus
2	Placebo	RVB 31	1	Normal		Fundus, Pylorus	0	0	0	0	0	0	0	0	0	Pyloric sphincter slightly open
2	Placebo	RVB 31	3	Normal			0	0	0	0	0	0	0	0	0	
2	Placebo	RVB 34	1	Normal		Fundus, Body, Incisura, Pylorus	0	0	0	0	0	0	0	0	0	
2	Placebo	RVB 34	3	Normal			0	0	0	0	0	0	0	0	0	

TABLE A34. UGI part 2 Duodenum lesion code.

Group	Treatment	Patient No.	Visit No.	Duodenum /Jejunum	Distance tip of scope advanced	Papilla seen?	Can't inflate lumen	Hyperemia/Vascularity	Edema	Discolouration	Friability	Texture	Haemorrhage	Erosion/Ulcer	Lacteal dilation	Contents	Comments
1	BIOEC2015	RVB01	1	Normal	120	Yes	0	0	0	0	0	0	1	0	0	1	bile
1	BIOEC2015	RVB01	3	Normal	100	No	0	1	1	0	1	0	0	0	0	1	mild changes
1	BIOEC2015	RVB07	1	Lesion	40	Yes	1	2	2	3	3	2	1	0	2	0	
1	BIOEC2015	RVB08	1	Lesion	unknown	Yes	0	1	0	1	1	0	1	0	0	0	
1	BIOEC2015	RVB08	3	Lesion	120	Yes	0	1	0	0	2	0	0	0	0	0	Mild hyperemia but moderate diffuse friability. Making tunnels witin the mucosa with the forceps
1	BIOEC2015	RVB13	1	Lesion	120	No	0	0	2	0	3	2	2	0	0	0	Generalised inflammation
1	BIOEC2015	RVB13	3	Lesion	150	Yes	0	2	1	2	0	1	0	0	2	0	
1	BIOEC2015	RVB16	1	Lesion	100	Yes	0	2	1	2	2	2	1	0	0	0	Hyperaemic dicolouration. Edematus with cobblestone appearance. Friable resulting in haemmorhage, no haemorrhage prior to sampling

1	BIOEC2015	RVB16	3	Lesion	100	No	0	2	1	2	3	2	0	0	1	0	Duodenum very friable, discoloured with abnormal texture. Mild lacteal dilation.
1	BIOEC2015	RVB18	1	Lesion	100	Yes	0	2	1	1	1	1	0	0	0	0	
1	BIOEC2015	RVB18	3	Normal	90	Yes	0	0	0	0	0	0	0	0	0	0	
1	BIOEC2015	RVB27	1	Lesion	90	No	0	1	2	1	2	2	0	0	0	1	
1	BIOEC2015	RVB29	1	Lesion	130	No	0	2	1	2	2	1	0	0	1	0	
1	BIOEC2015	RVB29	3	Lesion	130	Yes	0	0	2	0	0	2	1	0	0	0	Oedematous cobblestoned appearance of duodenum with multiple pinpoint haemorrhages on visible villi
1	BIOEC2015	RVB32	1	Lesion	103	No	0	1	0	0	0	0	0	0	0	0	Generalised hyperemia of whole region of duodenum examined
1	BIOEC2015	RVB32	3	Lesion	130	No	0	0	1	0	1	1	1	0	0	1	mucus
1	BIOEC2015	RVB35	1	Lesion	100	No	0	2	2	2	2	2	2	0	2	0	Diffusely oedematous and inflamed. Lacteals very visible
1	BIOEC2015	RVB35	3	Lesion	140	No	0	2	3	2	2	2	2	0	2	0	
2	Placebo	RVB02	1	Normal	80	No	0	0	0	0	0	0	0	0	0	0	
2	Placebo	RVB02	3	Lesion	80	No	0	1	1	0	1	0	0	0	0	0	
2	Placebo	RVB05	1	Lesion	100	No	0	2	2	0	2	0	1	0	2	0	
2	Placebo	RVB09	1	Lesion	60	No	0	3	2	3	3	3	3	0	1	2	a lot of bile
2	Placebo	RVB11	1	Lesion	140	No	1	2	1	2	2	1	3	0	0	0	Cobblestone appearance in duodenum

2	Placebo	RVB11	3	Lesion	110	No	0	1	0	1	1	0	0	0	0	0	Frequent areas of inflammation
2	Placebo	RVB12	1	Lesion	100	No	0	1	1	0	0	1	0	0	0	0	mild hyperaemia, mild oedema, mild cobblestone appearance
2	Placebo	RVB20	1	Normal	130	Yes	0	0	0	0	0	0	0	0	0	0	
2	Placebo	RVB20	3	Normal	130	Yes	0	0	0	0	0	0	0	0	0	0	
2	Placebo	RVB28	1	Lesion		Yes	0	1	1	1	1	0	0	0	0	0	
2	Placebo	RVB30	1	Lesion	103	No	0	0	0	0	0	0	0	0	1	0	
2	Placebo	RVB31	1	Lesion	90	Yes	0	0	1	0	0	2	0	0	0	0	Able to see villi very well. Cobblestone appearance.
2	Placebo	RVB31	3	Normal	100	No	0	0	0	0	0	0	0	0	0	0	
2	Placebo	RVB34	1	Lesion	130	No	0	2	2	1	3	2	0	0	0	1	
2	Placebo	RVB34	3	Lesion	135	No	0	1	1	1	0	1	1	0	0	0	

TABLE A35. LGI part 1 equipment and sampling.

Group	Treatment	Patient No.	Visit No.	Endoscope Used	Forceps Used	Method of colon preparation	Colon Prep Adequate?	Problems/Complications	Reason unable to complete full examination	Reason unable to obtain adequate biopsies	Reason visualisation obscured	Sampling	Documentation
1	BIOEC2015	RVB01	1	GIF-H260	EndoJaw 1550mm Model FB230K	warm water enema and kleen prep	Yes	None				Biopsy	None
1	BIOEC2015	RVB01	3	GIF-H260	EndoJaw 1550mm Model FB230K	warm water enema and kleen prep	Yes	None				Biopsy	Photo
1	BIOEC2015	RVB07	1	GIFH260	1550mm 2.8mm	Enema, Warm water enema 02 Dec 15 and kleen prep 01 dec 15	Yes	None				Biopsy	Video, photo
1	BIOEC2015	RVB08	1	CF H260 AL	Endojaw FB 2300mm	golytely enema	Yes	None				Biopsy	Video, photo
1	BIOEC2015	RVB08	3	CF H260 AL + GIF-H260	Endojaw FB-230U 2300mm + FB-230K 1550mm	Kleen prep, fletchers enema, warm water enema	Yes	None				Biopsy	Photo
1	BIOEC2015	RVB13	1	CFH260AL	EndoJaw 2300mm	Kleen prep/ fletchers enema/warm water enema	Yes	Excessive bleeding, Unable to obtain adequate biopsies		Fell out of ileum		Biopsy	Photo

1	BIOEC2015	RVB13	3	CFH260AL	FB230u EndoJaw	Kleen Prep, Fletchers enema, warm water enema x 2	No	None				Biopsy	Photo
1	BIOEC2015	RVB16	1	GIF-H260	EndoJaw FB - 230K	Warm water enema, fletchers enema, kleen prep	Yes	None				Biopsy	None
1	BIOEC2015	RVB16	3	GIF H260	EndoJaw FB-230U	Kleen Prep, enema	No	Visualisation obscured			Inadequate prep	Biopsy	Photo
1	BIOEC2015	RVB18	1	GIF - H260	FB-230K EndoJaw	Kleen prep, warm water enema, fletchers enema	Yes	None				Biopsy	None
1	BIOEC2015	RVB18	3	GIF XP260N + GIF-H260	FB-230U EndowJaw	Kleen Prep x 2 Warm water enema x 2 Fletcher's enema	No	None				Biopsy	None
1	BIOEC2015	RVB27	1	CFH260AL	2.8mm/2300mm	2 x warm water flush	No	Unable to complete full examination	inability to enter ileum			Biopsy	Photo
1	BIOEC2015	RVB29	1	CF-H260AL	EndoJaw FB-230U Oval Fenestrated	Kleen Prep x 2, warm water enema x1, fletchers enema x 1	Yes	Unable to complete full examination, Unable to obtain adequate biopsies	Not possible to intubate ileum	No ileal biopsies		Biopsy	Photo

1	BIOEC2015	RVB29	3	CF-H260AL	EndoJaw FB-230U Oval Fenestrated	Warm water enema x 3	Yes	Unable to complete full examination, Unable to obtain adequate biopsies	unable to enter ileum	blind biopsies not taken		Biopsy	Photo
1	BIOEC2015	RVB32	1	GIF H260	EndoJaw FB-230k Swing Jaw Oval Fenestrated	Clean prep, warm water enema x 1, fletcher's enema	No	Unable to complete full examination, Unable to obtain adequate biopsies	unable to advance through ileo-colic valve	unable to advance through ileo-colic valve		Biopsy	Photo
1	BIOEC2015	RVB32	3	CF-H260AL	EndoJaw FB-230U Oval Fenestrated Swing Jaw	Warm water enema x 2	Yes	Unable to complete full examination, Unable to obtain adequate biopsies	could not get into ileum	could not get into ileum		Biopsy	Photo
1	BIOEC2015	RVB35	1	Olympus CF-H260AL	EndoJaw Oval Fenestrated SwingJaw FB-230U	Warm water enema x 3	Yes	None				Biopsy	Photo
1	BIOEC2015	RVB35	3	CF-H260AL	EndoJaw FB-230U	Warm water enema under GA	No	Visualisation obscured			Faecal material	Biopsy	Photo
2	Placebo	RVB02	1	GIFXP6N	1550mm 2.8mm Oval Fenestrated	warm water enema and kleen prep	Yes	Visualisation obscured			Faecal matter	Biopsy	None

2	Placebo	RVB02	3	GIF-H260	1550mm 2.8mm	warm water enema and kleen prep orally	No	None				Biopsy	Photo
2	Placebo	RVB05	1	GIF-H260	EndoJaw FB230K	Enemas, warm water and kleen prep	No					Biopsy	Photo
2	Placebo	RVB09	1	CFH 260	1550mm 2.8mm	Kleen prep 26 Jan 16, 2 x enemas 27 Jan 16 (water)	Yes	None				Biopsy	Photo
2	Placebo	RVB11	1	CF-H260AL	EndoJaw FB-230u	Clean prep and warm water enemas	Yes	None				Biopsy	None
2	Placebo	RVB11	3	CFH260-AL	EndoJaw FB-230U	Kleen Prep and warm water enema	Yes	None				Biopsy	None
2	Placebo	RVB12	1	CFH260AL 3.7mm Biopsy Channel	2.8mm/2300mm biopsy forceps	Warm water enema and clean prep 10 Mar 16, Warm water enema 11 Mar 16	Yes	Unable to obtain adequate biopsies		All biopsies required were not collected as the scope came out of the ileum and could no re-enter.		Biopsy	Photo
2	Placebo	RVB20	1	GIF-H260	FB-230U EndoJaw	Kleen prep, fletchers and warm water enemas	No	None				Biopsy	Photo
2	Placebo	RVB20	3	CFH 260 AL	EndoJaw FB-230U	Warm water enemas,	No	Visualisation obscured			Faecal Matter	Biopsy	Photo

						clean prep, fletchers enema							
2	Placebo	RVB28	1	2049408	FB230-K swing jaw	clean prep, warm prep x 2	Yes	None				Biopsy	Photo
2	Placebo	RVB30	1	GIF-H260	EndoJaw FB-230U Oval Fenestrated SwingJaw	1 x fletchers enema, 2 x warm water enema, clean prep, lactulose	Yes	Unable to complete full examination, Unable to obtain adequate biopsies	Could not pass through ileocolic valve	Could not pass through ileocolic valve		Biopsy	Photo
2	Placebo	RVB31	1	GIF-H260	EndoJaw FB-230U Swing Jaw Oval Fenestrated	Clean prep, warm water enema x 2, fletchers enema	No	Unable to complete full examination, Unable to obtain adequate biopsies, Visualisation obscured	unable to enter ileum	unable to biopsy ileum	faecal matter	Biopsy	Photo
2	Placebo	RVB31	3	GIF-H260	EndoJaw FB-230k Swing Jaw Oval Fenestrated	1 X warm water enema	Yes	Unable to complete full examination, Unable to obtain adequate biopsies	could not access ileum	could not biopsy ileum		Biopsy	Photo

2	Placebo	RVB34	1	Olympus CF-H260 AL	Olympus EndoJaw Oval Fenestrated SwingJaw FB-230U	Warm water enema	Yes	None				Biopsy	Photo
2	Placebo	RVB34	3	CF-H260AL	EndoJaw FB-230U	Kleen Prep x 2, Fletchers Enema, Warm water enema	Yes	Excessive bleeding, Visualisation obscured			bleeding	Biopsy	Photo

TABLE A36. LGI part 1.

Group	Treatment	Patient No.	Visit No.	Colon	Visualised ileo-colic valve?	Visualised ceco-colic valve	Colon - Distance scope advanced (cm)	Hyperaemia/Vascularity	Discolouration	Friability/Haemorrhage	Erosion/Ulcer	Intussusception	Mass	Stricture	Artefact	Foreign body	Other	Comments
1	BIOEC2015	RVB01	1	Normal	Yes	Yes		1	0	0	0	0	0	0	0	0	0	
1	BIOEC2015	RVB01	3	Normal	Yes	Yes	90	0	0	0	0	0	0	0	0	0	0	
1	BIOEC2015	RVB07	1	Normal	Yes	Yes		0	0	0	0	0	0	0	0	0		
1	BIOEC2015	RVB08	1	Lesion	Yes	Yes		0	0	0	0	0	0	0	0	0	1	lymphoid follicles seen in descending duodenum
1	BIOEC2015	RVB08	3	Normal	Yes	Yes		0	0	0	0	0	0	0	0	0	0	
1	BIOEC2015	RVB13	1	Lesion	Yes	Yes		0	0	1	0	0	0	0	0	0	0	
1	BIOEC2015	RVB13	3	Normal	Yes	Yes		0	0	0	0	0	0	0	0	0	0	
1	BIOEC2015	RVB16	1	Normal	Yes	Yes		0	0	0	0	0	0	0	0	0	0	
1	BIOEC2015	RVB16	3	Lesion	Yes	Yes		1	1	1	0	0	0	0	0	0	0	Patchy discolouration and friable along entirety
1	BIOEC2015	RVB18	1	Normal	Yes	Yes		0	0	0	0	0	0	0	0	0	0	
1	BIOEC2015	RVB18	3	Normal	Yes	Yes		0	0	0	0	0	0	0	0	0	0	
1	BIOEC2015	RVB27	1	Normal	Yes	Yes	100	0	0	0	0	0	0	0	0	0	0	
1	BIOEC2015	RVB29	1	Normal	Yes	Yes	80	0	0	0	0	0	0	0	0	0	0	
1	BIOEC2015	RVB29	3	Normal	Yes	Yes	70	0	0	0	0	0	0	0	0	0	0	
1	BIOEC2015	RVB32	1	Normal	Yes	Yes		0	0	0	0	0	0	0	0	0	0	

1	BIOEC2015	RVB32	3	Lesion	Yes	Yes	70	0	0	1	0	0	0	0	0	0	0	
1	BIOEC2015	RVB35	1	Lesion	Yes	Yes	30	2	2	2	0	0	0	0	0	0	2	Swelling of mucosa after biopsy
1	BIOEC2015	RVB35	3	Normal	Yes	Yes	160	0	0	0	0	0	0	0	0	0	0	
2	Placebo	RVB02	1	Normal	Yes	Yes		0	0	0	0	0	0	0	0	0		
2	Placebo	RVB02	3	Normal	Yes	Yes		0	0	0	0	0	0	0	0	0	0	
2	Placebo	RVB05	1	Normal	Yes	Yes		0	0	0	0	0	0	0	0	0	0	
2	Placebo	RVB09	1	Lesion	Yes	Yes		0	0	1	0	0	0	0	0	0	0	
2	Placebo	RVB11	1	Lesion	Yes	Yes	120	0	0	1	0	0	0	0	0	0	0	
2	Placebo	RVB11	3	Normal	Yes	No		0	0	0	0	0	0	0	0	0	0	
2	Placebo	RVB12	1	Normal	Yes	Yes		0	0	0	0	0	0	0	0	0	0	
2	Placebo	RVB20	1	Normal	Yes	Yes		0	0	0	0	0	0	0	0	0	0	
2	Placebo	RVB20	3	Normal	Yes	Yes		0	0	0	0	0	0	0	0	0	0	
2	Placebo	RVB28	1	Normal	Yes	Yes	45	0	0	0	0	0	0	0	0	0	0	
2	Placebo	RVB30	1	Lesion	Yes	Yes	50	0	0	1	0	0	0	0	0	0	0	
2	Placebo	RVB31	1	Normal	Yes	Yes	50	0	0	0	0	0	0	0	0	0	0	
2	Placebo	RVB31	3	Normal	Yes	Yes	40	0	0	0	0	0	0	0	0	0	0	
2	Placebo	RVB34	1	Lesion	Yes	Yes	70	3	1	2	1	0	0	0	0	0	0	
2	Placebo	RVB34	3	Lesion	Yes	Yes		0	0	1	0	0	0	0	0	0	0	

TABLE A37. LGI Part 2.

Group	Treatment	Patient No.	Visit No.	Ileum Examined?	Passed scope through ileocolic valve?	Ileum - Biopsy taken?	Ileum - Biopsy taken by?	Ileum	Can't inflate lumen	Hyperaemia/Vascularity	Oedema	Discolouration	Friability/Haemorrhage	Erosion/Ulcer	Lacteal dilation	Texture Of Mucosa	Mass	Other	Comments
1	BIOEC2015	RVB 01	1	Yes	Yes	Yes	Direct Visualisation	Normal	2	1	0	0	0	0	0	0	0	0	
1	BIOEC2015	RVB 01	3	No	No	Yes	Blindly passing forceps through ileocolic valve												
1	BIOEC2015	RVB 07	1	Yes	Yes	Yes	Direct Visualisation	Lesion	0	2	3	3	3	0	3	3	0	0	
1	BIOEC2015	RVB 08	1	Yes	Yes	Yes	Direct Visualisation	Lesion	2	2	1	1	0	0	1	0	0	0	
1	BIOEC2015	RVB 08	3	No	No	No													
1	BIOEC2015	RVB 13	1	Yes	Yes	Yes	Direct Visualisation	Lesion	0	0	0	0	3	0	0	1	0	0	Excessive haemorrhage
1	BIOEC2015	RVB 13	3	Yes	Yes	Yes	Direct Visualisation	Normal	0	0	0	0	0	0	0	0	0	0	
1	BIOEC2015	RVB 16	1	Yes	Yes	Yes	Direct Visualisation	Lesion	0	2	2	2	2	0	0	2	0	0	
1	BIOEC2015	RVB 16	3	Yes	Yes	Yes	Direct Visualisation	Lesion	0	1	0	1	1	0	0	0	0	0	Patchy discolouration and friability
1	BIOEC2015	RVB 18	1	Yes	Yes	Yes	Direct Visualisation	Lesion	0	1	2	1	1	0	0	2	0	0	

1	BIOEC2015	RVB 18	3	No	No	Yes	Blindly passing forceps through ileocolic valve												
1	BIOEC2015	RVB 27	1	No	No	No													
1	BIOEC2015	RVB 29	1	No	No	No													
1	BIOEC2015	RVB 29	3	No	No	No													
1	BIOEC2015	RVB 32	1	No	No	No													
1	BIOEC2015	RVB 32	3	No	No	No													
1	BIOEC2015	RVB 35	1	No	No	No													
1	BIOEC2015	RVB 35	3	No															
2	Placebo	RVB 02	1	Yes	Yes	Yes	Direct Visualisation	Normal	0	0	0	0	1	0	0	0	0		
2	Placebo	RVB 02	3	Yes	Yes	Yes	Direct Visualisation	Normal	0	0	0	0	0	0	0	0	0	0	
2	Placebo	RVB 05	1	Yes	Yes	Yes	Direct Visualisation												
2	Placebo	RVB 09	1	Yes	Yes	Yes	Direct Visualisation	Lesion	0	2	0	3	3	0	0	3	0	0	
2	Placebo	RVB 11	1	Yes	Yes	Yes	Direct Visualisation	Lesion	0	2	1	2	3	0	0	3	0	0	Cobblestone appearance
2	Placebo	RVB 11	3	Yes	Yes	Yes	Direct Visualisation	Lesion	0	0	1	0	2	0	0	1	0	0	
2	Placebo	RVB 12	1	Yes	Yes	Yes	Direct Visualisation	Lesion	0	0	1	0	1	0	0	0	0	0	Mild oedema. Mild haemorrhage on obtaining biopsies consistent with friable mucosa
2	Placebo	RVB 20	1	Yes	Yes	Yes	Direct Visualisation	Normal	0	0	0	0	0	0	0	0	0	0	

2	Placebo	RVB 20	3	No															
2	Placebo	RVB 28	1	Yes	Yes	Yes	Direct Visualisation	Normal	0	0	0	0	0	0	0	0	0		
2	Placebo	RVB 30	1	No	No	No													
2	Placebo	RVB 31	1	No	No	No													
2	Placebo	RVB 31	3	No	No	No													
2	Placebo	RVB 34	1	No	No	No													
2	Placebo	RVB 34	3	Yes	Yes	Yes	Direct Visualisation	Lesion	1	1	1	1	3	0	0	2	0	0	

TABLE A38. LGI part 2 cecum

Group	Treatment	Patient No.	Visit No.	Cecum - Examined?	Cecum - intubated?	Cecum - Normal?
1	BIOEC2015	RVB01	1	Yes	yes	yes
1	BIOEC2015	RVB01	3	Yes	yes	yes
1	BIOEC2015	RVB07	1	Yes	yes	yes
1	BIOEC2015	RVB08	1	Yes	yes	yes
1	BIOEC2015	RVB08	3	Yes	yes	yes
1	BIOEC2015	RVB13	1	Yes	yes	yes
1	BIOEC2015	RVB13	3	Yes	yes	yes
1	BIOEC2015	RVB16	1	No		
1	BIOEC2015	RVB16	3	Yes	yes	yes
1	BIOEC2015	RVB18	1	No		
1	BIOEC2015	RVB18	3	No		
1	BIOEC2015	RVB27	1	Yes	yes	yes
1	BIOEC2015	RVB29	1	No		
1	BIOEC2015	RVB29	3	No		
1	BIOEC2015	RVB32	1	Yes	yes	yes
1	BIOEC2015	RVB32	3	Yes	yes	yes
1	BIOEC2015	RVB35	1	No		
1	BIOEC2015	RVB35	3	Yes	yes	yes
2	Placebo	RVB02	1	No		
2	Placebo	RVB02	3	Yes	yes	yes
2	Placebo	RVB05	1			
2	Placebo	RVB09	1	Yes	yes	yes
2	Placebo	RVB11	1	Yes	yes	yes
2	Placebo	RVB11	3	No		
2	Placebo	RVB12	1	Yes	yes	yes
2	Placebo	RVB20	1	Yes	yes	yes
2	Placebo	RVB20	3	Yes	yes	yes
2	Placebo	RVB28	1	Yes	yes	yes
2	Placebo	RVB30	1	Yes	yes	yes

2	Placebo	RVB31	1	No		
2	Placebo	RVB31	3	No		
2	Placebo	RVB34	1	No		
2	Placebo	RVB34	3	No		

TABLE A39. Deviations.

Patient No.	Visit No.	Deviation date	Deviation	Action/Impact
RVB02	1	16-Oct-15	Poor visibility in colon due to faecal matter and bleeding. Not all biopsies required were collected.	Will only be able to process duodenal samples for this dog.
RVB03	1	03-Nov-15	Faeces not stored in RVC Canine IBD archive in error.	Action taken: N/A. Dog was not included in trial as he didn't eat the prescription diet.
RVB02	3	07-Jan-16	Only a small amount of urine could be collected, so there was not enough to be able to store any. Max was walked out many times before endoscopy but no urine was passed. Bladder was too small for cystocentesis.	No impact on study.
RVB05	1	19-Nov-15	Form 10 lower GI page 2 - details were not completed for ileum and Cecum as Oliver Garden took samples for the study (ASPA licenced procedure) and did not complete the data at the time of the scope. James Swann (who performed the rest of the procedure) was unable to complete this section of the form.	Patient has been withdrawn from the study so no action taken. No impact on study.
RVB09	1	27-Jan-16	Dog seen 1 week ago and assessed as suitable for trial. Client had to think about decision to sign up for trial. Clinically exactly the same as 1 week ago, therefore test results from 1 week ago taken as results for 1st visit.	Tests not repeated. Taken results from one week ago.
RVB09	N/A	18-Feb-16	Visit 2 conducted on the wrong date 18Feb16. Patient had only been on the study drug for 1 week, not two. Blood and faeces collected and paperwork completed as unscheduled visit.	Visit 2 appointment made for 24Feb16. Action: Incorrect visit 2 recorded as unscheduled visit. No impact on study.
RVB11	1	04-Feb-16	Only one "plain" biopsy from the duodenum and one "snap frozen" biopsy sample from the duodenum were collected due to difficulty obtaining the samples.	No impact on study.
RVB02	7	02-Mar-16	Only enough urine could be collected for analysis. Multiple attempts were made to collect urine to store. Unfortunately they were unsuccessful.	No impact on study.
RVB01	7	02-Mar-16	No urine collected for analysis or storage. Multiple attempts were made but unfortunately they were unsuccessful.	No impact on study.

RVB05	withdrawal	02-Dec-15	Dog was removed from study because of deterioration. Dog did not come to see us at that time. Dog received 5 doses of compound / placebo with last dose given on 01Dec15. Dog started receiving steroids and cyclosporine on 05Dec15, which was ongoing at the time of visiting us on 21Dec15.	1) Withdrawal form has now been completed. 2) Data collected at visit on 21Dec15 cannot be used for the study.
N/A	N/A	23-Mar-16	An old version of form 11 was used in error for hospital visits conducted between 15Dec15 and 22Mar16.	All forms have now been replaced. No impact on study as only formatting changes.
RVB12	2	30-Mar-16	Owners did not bring in a faecal sample with them at visit 2 and a faecal swab was not taken in error. Owner brought in a faecal sample on 03Apr16 which has been processed and stored.	Owners reminded again that a faecal sample will need to be collected for each hospital visit. CIC nurses understand that a faecal swab should be taken if a faecal sample is not collected. No substantial impact on study.
RVB12	2	30-Mar-16	Royal veterinary College Pathology laboratory sent the blood sample taken for albumin at Visit 2 away to an external laboratory for analysis. This is because their lab machine was broken. The sample was sent to "Powell Torrance Diagnostic Services".	None. Albumin results received. No substantive impact on study.
RVB09	3	19-Apr-16	As patient was withdrawn from study due to worsening of clinical signs, weight loss and needs to be started on immunomodulatory medication. All of visit 3 was not completed as the patient did not have an endoscopy, therefore no biopsy samples could be collected. Forms 11 page 2, 9, 10 could not be completed. No urine could be collected.	Dog withdrawn from study - no further sampling to occur. No impact on remainder of study, other than absence of data from this animal.
RVB11	1	04-Feb-16	Giardia sp detected in faeces on initial screening tests	Giardiasis is a known cause of diarrhoea in certain dogs and would confound the interpretation of any response to the diet and active product / placebo. The data from this case may have to be excluded from the final analysis.
RVB14	1	10-Jun-16	Snap frozen tissue biopsies were not collected from the ileum due to difficulty obtaining samples. Biopsies were also not collected for cell culture from all three areas (duodenum, ileum and colon) for this patient due to time constraints on the day of the endoscopy. Oliver Garden was informed about this on the morning of the endoscopy by email.	None. No impact on overall study, though clearly no data from these areas of GIT.
RVB11	7	16-Jun-16	Visit 7 - Only 2ml of serum gel collected instead of 3ml due to difficulty obtaining sample	None. No impact, sufficient volume for assays.

RVB13	1	08-Jun-16	Unable to collect a voided faecal sample from RVB13, a faecal swab was collected instead.	This study sample will not be available for inclusion, with minimal or no impact on the whole study.
RVB12	3	25-May-16	Patient withdrawn from study due to worsening of clinical signs of IBD, part way through visit 3. Therefore, endoscopy was not performed and therefore no samples collected or stored for the study and for the IBD archive. Urine and faeces not collected and so urinalysis not submitted. No urine or faeces samples were stored for the study or IBD archive. Also, there was insufficient blood for the lab to perform a folate test, so there are no results for this test.	None - results missing for samples not submitted at this visit. Unable to take any further action. Impact statement: A complete dataset will not be available for this dog - i.e. only partial dataset.
RVB12	2 and 3	visit 2 - 30Mar16 and Visit 3 - 25May16	Incorrect version of form 11 "sample Record Form" used for visits 2 and 3 in error. It is believed that the study booklet was in use during the form switch over, so the forms were not changed.	All study booklets checked again to ensure correct version of form 11 filed. Impact statement: No substantive impact on study.
various	various	21-Jul-16	Blood glucose not performed in error for the following patients: RVB01 V1 and V7, RVB02 V3, RVB03 V1, RVB04 V1, RVB07 V1, RVB08 V3 and V7, RVB09 V1 and V3, RVB10 V1, RVB11 V1, V3 and V7, RVB12 V1 and V3, and RVB14 V1.	Lack of blood glucose concentrations will have minimal or no impact on the study.
RVB19 and 20	1	22-Aug-16	Both patients (RVB19 and 20) did not have an abdominal ultrasound scan during visit 1. Both patients have recently had an ultrasound scan, therefore the Investigator deemed it unnecessary to repeat.	Recent copies of the ultrasonography reports have been printed and authenticated by Investigator signature. Impact statement: This deviation is unlikely to have any impact on the study.
RVB13	5	19-Sep-16	Between V4 and V5 Mrs Robinson changed the diet from Purina HA over to Chappie. Within a few days Dillon showed signs of V+/D+. His CIBDAI score increased by more than 50%. According to the protocol, Dillon should now be fed Purina HA, however Mrs Robinson would like to try the Chappie again. She is currently changing the diet very slowly. If Dillon shows any signs of D+/V+, she will stop and only feed Purina HA for the remainder of the study.	Minimal impact on the study - for practical purposes, an insubstantive deviation.
RVB20	1	23-Aug-16	We were unable to obtain a faecal sample and therefore unable to perform faecal culture and floatation tests. There is no faeces for storage, however, a faecal swab was obtained and stored.	Only partial data for faecal analysis - in reality, unlikely to impact key data derived from the study adversely.
RVB22	1	13-Oct-16	Faecal culture results not obtained for visit 1. This is because we were unable to collect a faecal sample from the patient.	None - Oliver Garden confirmed that faecal culture is unnecessary at this point as the dog has not been

			A faecal swab was taken instead, for storage. However, QMHA staff managed to collect a faecal sample in the end but only sent it to the lab for parasitology. We decided not to request a faecal culture test on this sample as the dog was not enrolled on the study (as she did not eat the hypoallergenic diet).	enrolled on the study. Impact statement: No substantive impact on study.
RVB19	1	15-Aug-16	We were unable to collect a faecal sample and therefore unable to perform a faecal culture and floatation tests. There is no faeces for storage, however, a faecal swab was obtained and stored.	No substantive impact on study.
RVB13	7	07-Nov-16	On checking the owner diary I noticed that the owner started to introduce Wagg diet on the 15Oct16. She continued to give small amounts of the Wagg diet everyday up till the V7 appointment on 19Oct16. As discussed with Mrs Richardson, Dillon should have only been fed Purina HA exclusively. I am unsure why Mrs Richardson started to introduce the Wagg diet.	Impact on study: If Dillon did not experience a deterioration of clinical signs, the impact on the study is likely to be minimal at this visit. If deterioration of clinical signs was experienced the data for V7 may regrettably be challenging to interpret.
RVB18	3	N/A	There are two deviations to records for RVB18: 1) following the endoscopy at V3 Bella's owner reported that Bella was very sick and vomited chicken. This is a deviation as Bella should only be fed Purina HA for a week past endoscopy. WE are unsure where the dog received the chicken. 2) Due to the dog having sickness and diarrhoea post endoscopy we had to prolong the switch over from Purina HA to her normal diet for another week.	1) Impact on study: single dietary transgression unlikely to influence study in any substantive way - minimal impact. 2) Impact on study: Delayed transition to normal diet unlikely to impact study in any substantive way.
RVB18	5	10-Nov-16	On 29Oct16 the owner accidentally left the other dogs food down. Bella ate this food and due to this all day on the 29Oct16 she was vomiting. On the 30Oct16 she was lethargic but did not vomit anymore. The owner would now like to keep the dog on Purina HA. She continued to eat Purina HA and the test substance during the bout of sickness.	Impact on study: This dietary indiscretion and the subsequent flare-up of clinical signs may confound interpretation of the data gathered at visit 6. Long-term, this mishap is unlikely to impact the study's overall conclusions.
RVB23	N/A	09-Nov-16	On speaking to Mrs How she informed me that Dolly has been receiving Non-steroidal, since the 03NOv16. She also gave only 1 sachet of product on the 31Oct16. She now	The case will be withdrawn and replaced with a new case in due course.

			asked to be removed from the study due to difficulties with keeping treats away from Dolly due to her grandchildren.	
RVB20	4	17-Nov-16	Mrs Fry reported at V4 that on the 16Nov16 Woody has 3 bouts of watery diarrhoea in the morning. He did not eat his breakfast, however he ate his dinner with the IVP on it. He is back to normal today. The CCECAI score reflects the whole week not just this episode.	Consider it reasonable for patient to have been on normal diet for 4, rather than 7 days on this occasion. Minimal impact on study.
RVB20	N/A	02-Oct-16	On the 2nd October 2016 Woody may have eaten 3 mouthfuls of his litter mates food. Mrs Fry is sure he didn't eat the food but cannot be 100%. Therefore she has written this in the owner diary. Woody showed no signs of sickness in the days post the possible ingestion.	Impact on study: Minimal impact on study in absence of clinical signs.
RVB23	N/A	15-Nov-16	We would normally request the owners to bring back the patients for a withdrawal visit. However as the dog only received one dose of IVP we would have no data and therefore decided to not ask the patient to come back.	Impact on study: No impact on study since dog withdrawn from study.
RVB16	7	01-Dec-16	Mrs Glenn was unable to collect a urine sample from Rosie this morning. I tried again to obtain a free catch sample, however Rosie was very nervous and would not pass urine.	Impact on study: minimal impact on study.
RVB18	7	15-Dec-16	Mrs Daniel was unable to collect a urine sample, at the appointment we took Bella outside again and she still did not pass any urine. Therefore there is no urine sample for V7.	Minimal Impact on study
RVB27	1	07-Mar-17	Indigo would not defecate during her time in the hospital and would only urinate on her bed. Therefore a urine and faecal sample were not collected prior to her scope.	Impact statement: Presenting sign at current visit was vomiting - faeces could not be obtained at the first visit - but this is not considered to have a major impact on the study. Lack of urine sample is not considered to have an impact on the study.
RVB27	N/A	06-Jun-17	Owner was not able to administer IVP and it was decided that this dog needed to be withdrawn from the study. The owner attempted to but failed to administer the IVP after V2. This continued for several days but the owner did not record it in the owner diary as they felt it was unnecessary as the dog was not taking the test substance. A CIC nurse contacted the owner on 27Apr17 and later emailed them to	Information from Visit 2 will be used as a withdrawal visits. A Urine sample was not collected at this visit and it is required for the normal withdrawal visit. Lack of collection of urine at withdrawal visit has minimal impact on study.

			arrange withdrawal from the study. Unfortunately an immediate withdrawal visit was not arranged and the owner was not able to return sooner than the 06Jun17.	NTF 18/07/17: Owner only part dosed dog for 5 days between inclusion and V2. Investigator assessed that V2 data needed to be excluded due to non-compliance of dosing.
RVB31	1	18-Apr-17	Glucose was not run as part of the standard Biochemistry profile. The nurses who collected and sent the sample to the laboratory was not aware of this. Therefore blood glucose results are not available for RVB31 at visit 1.	Nurse is now aware that a sample for glucose must be collected and sent with the biochemistry sample. Minimal impact on study.
RVB30	1	10-Apr-17	Could not obtain a faecal sample as the dog did not pass faeces during its hospital stay. Could not obtain a sufficient sample during the rectal exam.	Swab taken for storage. Sample sent in by owner 5 days post discharge for microbiology and parasitology. All Faecal results were found to be acceptable for enrolment. No significant impact on study.
RVB28	2	04-May-17	Owner was asked to start IVP on Wednesday the 19May17. They did not understand the significance of this and started the IVP on Tuesday the 18May17. As a result the prearranged Visit 2 appointment was on day 17 and this was not realised until the time of the appointment.	Minimal impact
RVB32	1	16-May-17	Dizzy did not pass faeces during her stay in the hospital, therefore a sample could not be obtained for storage or lab testing. The owner was said to have sent a sample in the post following Dizzy's discharge, however this was not received and thought to be lost in the post.	The owner will be bringing in a fresh faecal sample on V2, as by the time this was realised Dizzy had already started the diet. Parasite and bacteriological examination were negative. No impact.
RVB32	1	16-May-17	Urine was collected by the medicine nursing team on the 18th of May 2017 and sent to the laboratory for urinalysis. It was not recorded on its kennel sheet and the clinical investigations team was not informed. Therefore a sample was sent to the laboratory but none was stored.	The medicine nurses were asked if in future all samples collected could be clearly marked on the animals kennel sheet and to inform the CIC team. Minimal Impact.
RVB30	withdrawal	03-Jul-17	This dog had an ongoing skin condition. On 19May17 the referring vet prescribed Apoquel and cephalexin. Both of these drugs are considered to interfere with parameters on this study. Therefore a decision was made to withdraw this dog from the study. The dog has received medication for several days before CIC was informed.	Data from Visit 2 to be used as withdrawal visit.
RVB29	5	20-Jul-17	This dog had started on Purina HA prior to enrolment on the study. The owner was very reluctant to change the diet	Minimal impact on study

			since the number of episode of vomiting and diarrhoea had been greatly reduced since the dog started on the diet. At visit 5 the owner agreed to switch the diet to the dog was being fed prior to Purina HA.	
RVB29	7	23-Aug-17	We were unable to collect a urine sample from the patient on the day of the visit. The owners were given a pot to collect a urine sample and post this as soon as possible. The sample was received in the post 48hrs after the visit and processed according to the protocol.	Minimal Impact on study
RVB29	3	26-Jun-17	Histopathology samples were not collected and sent to the laboratory for analysis during the second endoscopy in error. Impact statement: this has an impact on study as no adequate histology samples are available to include this dog in the comparison between the endoscopies, frozen karnovski fixative samples were evaluated but considered inadequate. All other parameters collected from this dog (e.g. clinical score, blood results etc.) can be included and used.	Staff were retrained on the study protocol. Form 11 of the study booklet was redesigned to include the collection of samples for histopathology and fluoride oxalate for biochemistry, to reduce the risk of future errors. Frozen Karnovsky samples from the colon and duodenum were sent to histopathology laboratory to assess suitability for histological evaluation. A copy of the results have been placed in the study Booklet. Histology findings re to be assessed prior to inclusion of data.
RVB31	3	11-Jul-17	Histology samples were not collected and sent to the laboratory for analysis during the second endoscopy in error. Impact statement: this has an impact on study as no adequate samples are available for comparison between 1st and 2nd endoscopy. Frozen Karnovski fixative samples were evaluated but considered inadequate. All other parameters collected for this dog can be included.	Staff will be retrained on the study protocol. Form 11 of the study booklet was redesigned to include the collection of samples for histopathology and fluoride oxalate for biochemistry, to reduce the risk of future errors. Frozen samples from the colon and duodenum were sent to histopathology laboratory to assess suitability for histological evaluation. A copy of the results have been placed in the study Booklet. Histology findings re to be assessed prior to inclusion of data.
RVB35	2	25-Dec-17	Roxy ate some turkey and potatoes on Christmas day (25Dec17), when she should only be fed Purina HA.	Owners confirmed that no other treats have been fed and that they understand Roxy should be fed Purina HA only. Impact statement: Minimal Impact.
N/A	N/A	N/A	Statistical Analysis of the data was performed on R instead of SPPS.	Appropriate Statistics were applied in order to analyse the data. No impact on the study results

TABLE A40. Concomitant medication - Group 1.

Group	Treatment	Patient No.	Start Date	Generic Drug Name	Trade Name	Dose	Units	Route	Frequency	End Date
1	BIOEC2015	RVB01	15-Oct-15	Fenbendazole	Panacur	2.25	g	PO	Once daily	20-Oct-15
1	BIOEC2015	RVB01	11-Nov-15	Maropitant	Cerenia	8	mg	PO	Once	11-Nov-15
1	BIOEC2015	RVB01	17-Nov-15	Milbemycin/Praziquantel	Milbemax	2.5/25	mg	PO	Once	17-Nov-15
1	BIOEC2015	RVB01	16-Dec-15	Maropitant	Cerenia	8	mg	PO	once	16-Dec-15
1	BIOEC2015	RVB01	28-Dec-15	Fluralaner	Biavecto	250	mg	PO	Once	28-Dec-15
1	BIOEC2015	RVB01	04-Jan-16	Maropitant	Cerenia	8	mg	PO	Once	04-Jan-16
1	BIOEC2015	RVB01	06-Jan-16	Methadone	Methadone	2	mg	IV	Once	06-Jan-16
1	BIOEC2015	RVB01	06-Jan-16	Acepromazine	ACP	0.1	mg	IV	Once	06-Jan-16
1	BIOEC2015	RVB01	06-Jan-16	Propafol	Propafol+	60	mg	IV	Once	06-Jan-16
1	BIOEC2015	RVB01	06-Jan-16	Atropine	Atropine	0.2	mg	IV	Once	06-Jan-16
1	BIOEC2015	RVB01	29-Feb-16	Milbemycin/Praziquantel	Milbemax	2.5/25	mg	PO	Once	29-Feb-16
1	BIOEC2015	RVB01	02-Mar-16	Maropitant	Cerenia	8	mg	PO	Once	02-Mar-16
1	BIOEC2015	RVB07	10-Dec-15	B12 Injection	Vitbee	0.25	mg	SC	Once	10-Dec-15
1	BIOEC2015	RVB07	17-Dec-15	B12 Injection	Vitbee	0.25	mg	SC	Once	17-Dec-15
1	BIOEC2015	RVB07	23-Dec-15	B12 Injection	Vitbee	0.25	mg	SC	Once	23-Dec-15
1	BIOEC2015	RVB08	10-Dec-15	Fenbendazole	Panacur	4.5	g	PO	SID	15-Dec-15
1	BIOEC2015	RVB08	24-Feb-16	Methadone	Methadone	3.65	mg	IV	once	24-Feb-16
1	BIOEC2015	RVB08	24-Feb-16	Propofol	Propafol+	130	mg	IV	once	24-Feb-16

1	BIOEC2015	RVB08	24-Feb-16	Isoflurane	Isoflo	n/a	n/a	Inhalation	Once	24-Feb-16
1	BIOEC2015	RVB08	24-Feb-16	Sevoflurane	Sevoflo	n/a	n/a	Inhalation	Once	24-Feb-16
1	BIOEC2015	RVB08	09-Apr-16	Febanantel/Pyrantel Embonate/Praziquantel	Drontal Tasty Bone	300/288/100	mg	PO	Once	09-Apr-16
1	BIOEC2015	RVB13	10-Jun-16	Omeprazole	Omeprazole	40	mg	PO	SID	17-Jun-16
1	BIOEC2015	RVB13	25-Aug-16	Buprenorphine	Buprecare	0.777	mg	IV	Once	25-Aug-16
1	BIOEC2015	RVB13	25-Aug-16	Acepromazine	ACP	0.3885	mg	IV	Once	25-Aug-16
1	BIOEC2015	RVB13	25-Aug-16	Propafol	Propoflo	120	mg	IV	Once	25-Aug-16
1	BIOEC2015	RVB13	25-Aug-16	Propafol	Propoflo	19	mg	IV	Once	25-Aug-16
1	BIOEC2015	RVB13	25-Aug-16	Sevoflurane	Sevoflo	n/a	n/a	Inhalation	Once	25-Aug-16
1	BIOEC2015	RVB16	10-Sep-15	Phenylpropanolamine	Urilin	10	mg	PO	BID	Ongoing
1	BIOEC2015	RVB16	04-Aug-16	Maropitant	Cerenia	24	mg	PO	Once	04-Aug-16
1	BIOEC2015	RVB16	09-Aug-16	Maropitant	Cerenia	24	mg	PO	Once	09-Aug-16
1	BIOEC2015	RVB16	02-Oct-16	Maropitant	Cerenia	24	mg	PO	Once	02-Oct-16
1	BIOEC2015	RVB16	19-Sep-16	Chlorphenamine	Piriton	4	mg	PO	Once	19-Sep-16
1	BIOEC2015	RVB16	07-Oct-16	Butorphanol	Alvegesic	1.9	mg	IV	Once	07-Oct-16
1	BIOEC2015	RVB16	07-Oct-16	Medetomidine	Sedastart	0.019	mg	IV	Once	07-Oct-16
1	BIOEC2015	RVB16	07-Oct-16	Propafol	Propoflo	40	mg	IV	Once	07-Oct-16
1	BIOEC2015	RVB16	07-Oct-16	Buprenorphine	Buprecare	0.19	mg	IV	Once	07-Oct-16
1	BIOEC2015	RVB16	07-Oct-16	Sevoflurane	Sevoflo	n/a	n/a	Inhalation	Once	07-Oct-16
1	BIOEC2015	RVB16	07-Oct-16	Isoflurane	Isoflo	n/a	n/a	Inhalation	Once	07-Oct-16
1	BIOEC2015	RVB16	07-Oct-16	Propafol	Propoflo	5	mg	IV	Once	07-Oct-16
1	BIOEC2015	RVB16	07-Oct-16	Medetomidine	Sedastart	0.029	mg	IV	Once	07-Oct-16
1	BIOEC2015	RVB16	07-Oct-16	Butorphanol	Alvegesic	1.9	mg	IV	Once	07-Oct-16

1	BIOEC2015	RVB16	13-Oct-16	Maropitant	Cerenia	24	mg	PO	Once	13-Oct-16
1	BIOEC2015	RVB16	16-Nov-16	Leptospirosis	Nobivac L4	n/a	n/a	SC	Once	16-Nov-16
1	BIOEC2015	RVB18	17-Aug-16	B12 Injection	Vitbee	0.05	mg	SC	Once	17-Aug-16
1	BIOEC2015	RVB18	06-Aug-16	Fenbendazole	Panacur	1.8	g	PO	SID	08-Aug-16
1	BIOEC2015	RVB18	24-Aug-16	B12 Injection	Vitbee	0.05	mg	SC	Once	24-Aug-16
1	BIOEC2015	RVB18	02-Sep-16	B12 Injection	Vitbee	0.05	mg	SC	Once	02-Sep-16
1	BIOEC2015	RVB18	09-Sep-16	B12 Injection	Vitbee	0.05	mg	SC	Once	09-Sep-16
1	BIOEC2015	RVB18	16-Sep-16	B12 Injection	Vitbee	0.05	mg	SC	Once	16-Sep-16
1	BIOEC2015	RVB18	24-Sep-16	B12 Injection	Vitbee	0.05	mg	SC	Once	24-Sep-16
1	BIOEC2015	RVB18	08-Oct-16	B12 Injection	Vitbee	0.05	mg	SC	Once	08-Oct-16
1	BIOEC2015	RVB18	08-Sep-16	Fenbendazole	Panacur	1.8	g	PO	Once	08-Sep-16
1	BIOEC2015	RVB18	20-Oct-16	Medetomidine	Sedastart	0.085	mg	IV	Once	20-Oct-16
1	BIOEC2015	RVB18	20-Oct-16	Butorphanol	Alvegesic	1.7	mg	IV	Once	20-Oct-16
1	BIOEC2015	RVB18	20-Oct-16	Propafol	Propoflo	15	mg	IV	Once	20-Oct-16
1	BIOEC2015	RVB18	20-Oct-16	Sevoflurane	Sevoflo	n/a	n/a	Inhalation	Once	20-Oct-16
1	BIOEC2015	RVB18	20-Oct-16	Omeprazole	Omeprazole	8.5	mg	IV	Once	20-Oct-16
1	BIOEC2015	RVB18	20-Oct-16	Propafol	Propoflo	25	mg	IV	Once	20-Oct-16
1	BIOEC2015	RVB18	20-Oct-16	Medetomidine	Sedastart	0.085	mg	IV	Once	20-Oct-16
1	BIOEC2015	RVB18	24-Oct-16	B12 Injection	Vitbee	0.05	mg	SC	Once	24-Oct-16
1	BIOEC2015	RVB18	03-Nov-16	Imidacloprid	Advantage	100	mg	Topical	Once	03-Nov-16
1	BIOEC2015	RVB18	21-Nov-16	B12 Injection	Vitbee	0.05	mg	SC	Once	21-Nov-16
1	BIOEC2015	RVB18	12-Dec-16	Imidacloprid	Advantage	100	mg	Topical	Once	12-Dec-16
1	BIOEC2015	RVB27	14-Mar-17	Vitamin B12	VitBee250	800	µg	SC	Once	14-Mar-17
1	BIOEC2015	RVB27	21-Mar-17	B12 Cobalamin	Anivet	800	µg	SC	Once	21-Mar-17
1	BIOEC2015	RVB27	06-Apr-17	B12 Injection	Anivit B12	800	mcg	SC	Once	06-Apr-17
1	BIOEC2015	RVB27	28-Mar-17	B12 Injection	Anivit B12	800	mcg	SC	Once	28-Mar-17
1	BIOEC2015	RVB29	07-Apr-17	Tramadol	Tramadol	50	mg	PO	SID	04-May-17
1	BIOEC2015	RVB29	07-May-17	Selamectin	Stronghold	240	mg	Topical	Once	07-May-17
1	BIOEC2015	RVB29	28-Jun-17	Methadone	Synthadon	5.21	mg	IV	Once	28-Jun-17

1	BIOEC2015	RVB29	28-Jun-17	Acepromazine	ACP	0.13	mg	IV	Once	28-Jun-17
1	BIOEC2015	RVB29	28-Jun-17	Sevoflurane	Sevoflo	N/A	N/A	Inhalation	Once	28-Jun-17
1	BIOEC2015	RVB29	28-Jun-17	Propofol	Propoflo Plus	190	mg	IV	Once	28-Jun-17
1	BIOEC2015	RVB32	20-May-17	Vitamin B12	VitBee	1	mg	SC	Once	20-May-17
1	BIOEC2015	RVB32	27-May-17	Vitamin B12	VitBee	1	mg	SC	Once	27-May-17
1	BIOEC2015	RVB32	03-Jun-17	Vitamin B12	VitBee	1	mg	SC	Once	03-Jun-17
1	BIOEC2015	RVB32	10-Jun-17	Vitamin B12	VitBee	1	mg	SC	Once	10-Jun-17
1	BIOEC2015	RVB32	04-Jun-17	Milbemycin Oxime / Praziquantel	Milquantel	12.5 / 125	mg	PO	Once	04-Jun-17
1	BIOEC2015	RVB32	04-Jul-17	Milbemycin Oxime / Praziquantel	Milquantel	12.5 / 125	mg	PO	Once	04-Jul-17
1	BIOEC2015	RVB32	04-Aug-17	Milbemycin Oxime / Praziquantel	Milquantel	12.5 / 125	mg	PO	Once	04-Aug-17
1	BIOEC2015	RVB32	09-Aug-17	Methadone	Synthadon	4.8	mg	IV	Once	09-Aug-17
1	BIOEC2015	RVB32	09-Aug-17	Acepromazine	ACP	0.12	mg	IM	Once	09-Aug-17
1	BIOEC2015	RVB32	09-Aug-17	Propofol	Propoflo Plus	120	mg	IV	Once	09-Aug-17
1	BIOEC2015	RVB32	09-Aug-17	Sevoflurane	Sevoflo	N/A	N/A	Inhalation	Once	09-Aug-17
1	BIOEC2015	RVB32	17-Jun-17	Cobalamine	VitBee250	1	mg	SC	Once	17-Jun-17
1	BIOEC2015	RVB32	24-Jun-17	Cobalamine	VitBee250	1	mg	SC	Once	24-Jun-17
1	BIOEC2015	RVB32	16-Aug-17	Fluralaner	Bravecto	1000	mg	PO	Once	16-Aug-17
1	BIOEC2015	RVB32	04-Sep-17	Milbemycin / Praziquantel	Milquantel	12.5 / 125	mg	PO	Once	04-Sep-17
1	BIOEC2015	RVB32	22-Sep-17	Amoxicillin / Clavulanic Acid	Synulox	200 / 50	mg	PO	BID	27-Sep-17
1	BIOEC2015	RVB32	22-Sep-17	Robenacoxib	Onsior	40	mg	PO	SID	27-Sep-17
1	BIOEC2015	RVB32	02-Oct-17	Leptospira Interrogans	Nobivac lepto 4	1	ml	SC	Once	02-Oct-17

1	BIOEC2015	RVB32	02-Oct-17	Distemper, Adenovirus 2 + parvovirus	Nobivac DHP	1	ml	SC	Once	02-Oct-17
1	BIOEC2015	RVB32	02-Oct-17	Bordatella Bronchiseptica	Nobivac KC	0.4	ml	Nasal	Once	02-Oct-17
1	BIOEC2015	RVB35	28-Feb-18	Methadone	Synthadon	3.608	mg	IV	Once	28-Feb-18
1	BIOEC2015	RVB35	28-Feb-18	Acepromazine	ACP	0.0902	mg	IV	Once	28-Feb-18
1	BIOEC2015	RVB35	28-Feb-18	Propofol	Propoflo Plus	110	mg	IV	Once	28-Feb-18
1	BIOEC2015	RVB35	28-Feb-18	Isoflurane	Isocare	n/a	n/a	Inhalation	Once	28-Feb-18
1	BIOEC2015	RVB35	28-Feb-18	Sevoflurane	Sevoflo	n/a	n/a	Inhalation	Once	28-Feb-18
1	BIOEC2015	RVB35	28-Feb-18	Propofol	Propoflo Plus	9.02	mg	IV	Once	28-Feb-18

TABLE A41. Concomitant medication - Group 2.

Group	Treatment	Patient No.	Start Date	Generic Drug Name	Trade Name	Dose	Units	Route	Frequency	End Date
2	Placebo	RVB02	28-Nov-15	Imidacloprid/Moxidectin	Advocate	100/25	mg	Topical	Once a month	28-Dec-15
2	Placebo	RVB02	07-Jan-16	Butorphanol	Alvegesic	2.4	mg	IV	Once	07-Jan-16
2	Placebo	RVB02	07-Jan-16	Acepromazine	ACP	0.03	mg	IV	Once	07-Jan-16
2	Placebo	RVB02	07-Jan-16	Propofol	Propoflo Plus	20	mg	IV	Once	07-Jan-16
2	Placebo	RVB02	07-Jan-16	Sevoflurane	Sevoflo	n/a	n/a	Inhalation	Two doses	07-Jan-16
2	Placebo	RVB02	07-Jan-16	Medetomidine	Sedastart	0.008	mg	IV	Once	07-Jan-16
2	Placebo	RVB05	23-Oct-15	B12 Injection	Anivit B12	0.25	mg	SC	Once	23-Oct-16
2	Placebo	RVB05	05-Dec-15	Cyclosporine	Atopica	1000	mg	PO	SID	Ongoing
2	Placebo	RVB05	05-Dec-15	Prednisolone	Prednisolone	40	mg	PO	SID	Ongoing
2	Placebo	RVB05	04-Dec-15	B12 Injection	Vitbee	0.25	mg	SC	Once	04-Dec-15
2	Placebo	RVB05	10-Dec-15	B12 Injection	Vitbee	0.25	mg	SC	Once	10-Dec-15
2	Placebo	RVB05	17-Dec-15	B12 Injection	Vitbee	0.25	mg	SC	Once	17-Dec-15
2	Placebo	RVB09	03-Feb-16	B12 Injection	Vitbee	0.75	mg	SC	Once	03-Feb-16
2	Placebo	RVB09	12-Feb-16	B12 Injection	Vitbee	0.75	mg	SC	Once	12-Feb-16
2	Placebo	RVB09	17-Feb-16	B12 Injection	Vitbee	0.75	mg	SC	Once	17-Feb-16
2	Placebo	RVB09	24-Feb-16	B12 Injection	Vitbee	0.75	mg	SC	Once	24-Feb-16
2	Placebo	RVB09	02-Mar-16	B12 Injection	Vitbee	0.75	mg	SC	Once	02-Mar-16
2	Placebo	RVB09	05-Apr-16	B12 Injection	Vitbee	0.75	mg	SC	Once	05-Apr-16
2	Placebo	RVB09	12-Apr-16	B12 Injection	Vitbee	0.75	mg	SC	Once	12-Apr-16
2	Placebo	RVB09	18-Apr-16	B12 Injection	Vitbee	0.75	mg	SC	Once	18-Apr-16
2	Placebo	RVB11	05-Feb-16	Fenbendazole	Panacur	6.75	g	PO	SID	09-Feb-16
2	Placebo	RVB11	07-Apr-16	Imidacloprid/Moxidectin	Advocate	400/100	mg	Topical	Once	07-Apr-16
2	Placebo	RVB11	22-Apr-16	Buprenorphine	Buprecare	0.594	mg	IM	Twice	22-Apr-16

2	Placebo	RVB11	22-Apr-16	Medetomidine	Sedastart	0.297	mg	IM	Once	22-Apr-16
2	Placebo	RVB11	22-Apr-16	Propafol	Vetafol	50	mg	IV	Once	22-Apr-16
2	Placebo	RVB11	22-Apr-16	Isoflurane	Isocare	n/a	n/a	Inhalation	Once	22-Apr-16
2	Placebo	RVB11	25-May-16	Nobivac Lepto 4	Nobivac lepto 4	n/a	Vaccine	SC	Once	25-May-16
2	Placebo	RVB11	25-May-16	Nobivac KC	Nobivac KC	n/a	Vaccine	Intra-nasal	Once	25-May-16
2	Placebo	RVB12	12-Mar-16	Omeprazole	Omeprazole	40	mg	PO	SID	18-Mar-16
2	Placebo	RVB12	11-Mar-16	Fenbendazole	Panacur	1700	mg	PO	SID	13-Mar-16
2	Placebo	RVB12	07-Apr-16	B12 Injection	Vitbee	1	mg	SC	Once	07-Apr-16
2	Placebo	RVB12	14-Apr-16	B12 Injection	Vitbee	1	mg	SC	Once	14-Apr-16
2	Placebo	RVB12	21-Apr-16	B12 Injection	Vitbee	1	mg	SC	Once	21-Apr-16
2	Placebo	RVB12	27-Apr-16	Ranitide	Zantac	75	mg	SC	Once	27-Apr-16
2	Placebo	RVB12	27-Apr-16	Buprenorphine	Buprendale	0.67	mg	IM	Once	27-Apr-16
2	Placebo	RVB12	27-Apr-16	Maropitant	Cerenia	30	mg	SC	Once	27-Apr-16
2	Placebo	RVB12	05-May-16	B12 Injection	Vitbee	1	mg	SC	Once	05-May-16
2	Placebo	RVB12	17-May-16	B12 Injection	Vitbee	1	mg	SC	Once	17-May-16
2	Placebo	RVB20	24-Aug-16	Fenbendazole	Panacur	4.5	g	PO	SID	03-Sep-16
2	Placebo	RVB20	09-Sep-16	Imidacloprid/Moxidectin	Advocate	250/62.5	mg	Topical	Once	09-Sep-16
2	Placebo	RVB20	29-Sep-06	Praziquantel/Pyrantel/ Febantel	Cazitel	50/49.8/250	mg	PO	Once	29-Sep-16
2	Placebo	RVB20	03-Nov-16	Fenbendazole	Panacur	4.5	g	PO	Once	03-Nov-16
2	Placebo	RVB20	03-Nov-16	DA2Pi/CPV-L	Vanguard	n/a	n/a	SC	Once	03-Nov-16
2	Placebo	RVB20	10-Nov-16	Medetomidine	Domitor	0.06	mg	IV	Once	10-Nov-16
2	Placebo	RVB20	10-Nov-16	Butorphanol	Torbugesic	5.99	mg	IV	Once	10-Nov-16
2	Placebo	RVB20	10-Nov-16	Propofol	Propoflo	20	mg	IV	Once	10-Nov-16
2	Placebo	RVB20	10-Nov-16	Propofol	Propoflo	6	mg	IV	Once	10-Nov-16
2	Placebo	RVB20	10-Nov-16	Propofol	Propoflo	5	mg	IV	Once	10-Nov-16
2	Placebo	RVB20	10-Nov-16	Propofol	Propoflo	4	mg	IV	Once	10-Nov-16
2	Placebo	RVB20	12-Nov-16	Imidacloprid/Moxidectin	Advocate	250/62.5	mg	Topical	Once	12-Nov-16

2	Placebo	RVB20	16-Dec-16	Imidacloprid/Moxidectin	Advocate	250/62.5	mg	Topical	Once	16-Dec-16
2	Placebo	RVB28	01-Apr-17	Fenbendazole	Panacur 22 %	2	g	PO	SID	10-Apr-17
2	Placebo	RVB30	18-Apr-17	Artuvetrin		0.6	ml	SC	Once	18-Apr-17
2	Placebo	RVB30	30-Apr-17	Fusidic Acid	Isathal Eye drops	1	Drop	Topical	BID	07-May-17
2	Placebo	RVB30	01-May-17	Milbemycin Oxime/Praziquantel	Milbemax Tablets	12.5/125	mg	Oral	Once	01-May-17
2	Placebo	RVB31	27-Apr-17	B12	VitBee250	0.5	mg	SC	Once	27-Apr-17
2	Placebo	RVB31	03-May-17	Cobalamine	VitBee250	0.5	mg	SC	Once	03-May-17
2	Placebo	RVB31	11-May-17	Cobalamine	VitBee250	0.5	mg	SC	Once	11-May-17
2	Placebo	RVB31	18-May-17	Cobalamine	VitBee250	0.5	mg	SC	Once	18-May-17
2	Placebo	RVB31	15-Jun-17	Cobalamine	VitBee250	0.5	mg	SC	Once	15-Jun-17
2	Placebo	RVB31	13-Jul-17	Medetomidine	Sedastart	0.06	mg	IM	Once	13-Jul-17
2	Placebo	RVB31	13-Jul-17	Butorphanol	Alvegesic	2.5	mg	IM	Once	13-Jul-17
2	Placebo	RVB31	13-Jul-17	Medetomidine	Sedastart	0.06	mg	IM	Once	13-Jul-17
2	Placebo	RVB31	13-Jul-17	Sevoflurane	Sevoflo	N/A	N/A	Inhalation	Once	13-Jul-17
2	Placebo	RVB31	13-Jul-17	Propofol	Propoflo Plus	11.9	mg	IV	Once	13-Jul-17
2	Placebo	RVB31	26-Jul-17	Cobalamine	VitBee250	0.5	mg	SC	Once	26-Jul-17
2	Placebo	RVB34	15-Nov-17	Leptspirosis	Nobivac L4	1	ml	SC	Once	15-Nov-17
2	Placebo	RVB34	15-Nov-17	Bordetella Bronchiseptica	Nobivac KC	0.4	ml	Nasal	Once	15-Nov-17
2	Placebo	RVB34	03-Dec-17	Praziquantel	Droncit	150	mg	po	Once	03-Dec-17
2	Placebo	RVB34	12-Nov-17	Imadacloprid/Moxidectin	Advocate XL	400/100	mg	topical	Once	12-Nov-17
2	Placebo	RVB34	11-Oct-17	Imadacloprid/Moxidectin	Advocate XL	400/100	mg	topical	Once	11-Oct-17
2	Placebo	RVB34	07-Dec-17	Methadone	Synthadon	4.95	mg	IV	Once	07-Dec-17
2	Placebo	RVB34	07-Dec-17	Acepromazine	ACP	0.124	mg	IV	Once	07-Dec-17
2	Placebo	RVB34	07-Dec-17	Midazolam	Hypnovel	4.95	mg	IV	Once	07-Dec-17
2	Placebo	RVB34	07-Dec-17	Propofol	Propoflo Plus	unknown	mg	IV	Once	07-Dec-17
2	Placebo	RVB34	07-Dec-17	Sevofluorane	Sevoflo	n/a	n/a	inhalation	Once	07-Dec-17

2	Placebo	RVB34	14-Dec-17	Imadacloprid/Moxidectin	Advocate XL	400/100	mg	topical	Once	14-Dec-17
2	Placebo	RVB34	16-Jan-18	Imadacloprid/Moxidectin	Advocate XL	400/100	mg	topical	Once	16-Jan-18

TABLE A42. Adverse Events

Group	Treatment	Patient No.	Abnormal clinical signs observed / recorded by	Date first observed	Clinical signs still present?	End date	Severity of Clinical Signs	Clinical signs	Diagnosis	Tentative / Final	Treatment needed?
1	BIOEC2015	RVB07	Owner	01-Feb-16	yes	ongoing	Moderate	Hair loss on the tail, presumed weight loss, polydipsia, increase in frequency of diarrhoea.	IBD severe	Final	yes
1	BIOEC2015	RVB32	Veterinarian	approximately 18Sep17	No	24-Sep-17	Mild	Lameness on right foreleg	penetrating wound	tentative	yes
1	BIOEC2015	RVB35	Owner	18-Jan-18	No	18-Jan-18	Mild	Patient scavenged some food while out walking and was unwell afterwards with one episode of vomiting. No further vomiting since.	Dietary indiscretion.	Final	No
1	BIOEC2015	RVB35	Owner	10-Mar-18	No	14-Mar-18	Mild	Diarrhoea - The other dog in the home also had diarrhoea. Both dogs improved without treatment.	Unknown	Final	No

2	Placebo	RVB02	Owner	23-Nov-15	No	24-Nov-15	Mild	Retching twice; very quiet not himself.	IBD	Final	No
2	Placebo	RVB09	Investigator	Reported 19/04/2016	yes	ongoing	severe	Worsening signs of inflammatory bowel disease. Persistent watery diarrhoea, slightly decreased appetite, severe weight loss, marked hypoalbuminemia , marked elevation in liver enzymes.	worsening of IBD +/- liver disease	tentative	yes
2	Placebo	RVB11	owner	24-May-16	no	by 02/07/2016	mild	ear shaking	otitis externa	tentative	yes
2	Placebo	RVB12	Owner	22-Apr-16	No	29-Apr-16	severe	vomiting, lethargy, inappetance after eating margarine	Food indiscretion	tentative	yes
2	Placebo	RVB30	owner	28-Apr-17	no	03-May-17	mild	simmer tear test performed - 18mm right eye, 20mm left eye. Left eye slightly inflamed sclera, fluorescein test was negative.	Keratoconjunctivitis sicca	Tentative	yes
2	Placebo	RVB30	Owner	11-May-17	no	11-May-17	Mild	Vomited bile - drank from muddy puddle the previous day	dietary indiscretion; resoled without intervention	final	No

2	Placebo	RVB30	Owner	09-Jun-17	no	10-Jun-17	mild	Vomited on morning of 09Jun17 and decreased appetite on 10Jun17.	unknown - clinical signs resolved without intervention	tentative	no
2	Placebo	RVB31	Owner	13-Aug-17	no	13-Aug-17	Mild	Dog found and ate faeces whilst out on a walk. The dog then regurgitated the faeces approximately 30 seconds later.	dietary indiscretion	final	no
2	Placebo	RVB34	Owner	30-Oct-17	No	30-Oct-17	Mild	Spud was observed by owners to eat a "squeaker" from a squeaky toy. He then vomited up part of the squeaker and passed the rest in his faeces later without incident.	Ingestion of foreign body	final	No

TABLE A43. Adverse Events - treatment

Group	Treatment	Patient No.	Date first observed	Treatment needed?	Treatment given	Causality	Date AE recorded
1	BIOEC2015	RVB07	01-Feb-16	Yes	Appointment made at hospital 04Feb16. Moderate weight loss. Biochemistry, haematology, and folate and cobalamine run. Prednisolone prescribed.	Other. Worsening clinical signs of IBD.	02-Feb-16
1	BIOEC2015	RVB32	approximately 18Sep17	Yes	Yes - please see concomitant treatment form	Disease/condition diagnosed after enrolment	27-Sep-17
1	BIOEC2015	RVB35	18-Jan-18	No		Unknown	23-Jan-18
1	BIOEC2015	RVB35	10-Mar-18	No		Unknown	19-Mar-18
2	Placebo	RVB02	23-Nov-15	No		Other	25-Nov-15
2	Placebo	RVB09	Reported 19/04/2016	Yes	Further investigations needed to assess liver function but needs immunosuppressant therapy for IBD. Withdrawn from trial due to worsening signs of IBD.	Other	29-Apr-16

2	Placebo	RVB11	24-May-16	Yes	Triz aural crystals with 4ml dexamethasone (colvasone) dispensed but not yet administered by owner. Update 16Jun16: owner confirmed at visit 7 on 16Jun16 that ear drops were not administered / used at any point.	Other	recorded 26May16, signed 27/05/2016
2	Placebo	RVB12	22-Apr-16	Yes	Omeprazole, IVFT and maropitant	Other	Recorded 28Apr16, Signed by investigator 29/04/2016
2	Placebo	RVB30	28-Apr-17	Yes	Fusidic acid (Isathal eye drops), one drop applied to left eye twice daily for 7 days.	Disease/condition diagnosed after enrolment	06-Jun-17
2	Placebo	RVB30	11-May-17	No		Other	01-Aug-17
2	Placebo	RVB30	09-Jun-17	No		Unknown	01-Aug-17
2	Placebo	RVB31	13-Aug-17	No		Other	25-Aug-17
2	Placebo	RVB34	30-Oct-17	No		Other	01-Nov-17

TABLE A44. Clinical pathology results - Group 1.

Group	Treatment	Patient No.	Visit No.	Tissue - Microscopic description - Stomach	Tissue - Microscopic description - Duodenum	Tissue - Microscopic description - Ileum	Tissue - Microscopic description - Colon
1	BIOEC2015	RVB01	1	F2971 Stomach (10 sections and smaller fragments, one level) the sections extend from the surface mucosa to the deeper aspects lamina propria and do not include muscularis mucosa. The epithelial surface exhibits multifocal moderate to marked attenuation. Prominent bands of fibrocytes extend through these sections, from the superficial lamina propria, to the deeper aspects of the sections, were they surround and isolate individual crypts. They are present in bands of up to 10 cells thick. Low numbers of lymphocytes and plasma cells are present within the lamina propria in these number up to 20 per 40 X (high powered) field. Low numbers of neutrophils and eosinophils are present and these number up to 5 per 40 X field. In some of the sections, more prominent lymphocyte numbers are present with up to 100 noted per 40 X field. In these areas, low numbers of intraepithelial lymphocytes are	F2972 Duodenum (10 pieces and smaller fragments, one level). These sections extend from the surface mucosa to the mid and deep lamina propria. They do not include muscularis mucosa. Villi are reduced to approximately 75% of their expected length. Villi are approximately 1 to 1 and 1/2 times increased in with. Villi are lined by tall columnar well differentiated epithelial cells. Low numbers of intraepithelial lymphocytes are present. The lamina propria contains low numbers of lymphocytes and plasma cells which occupying up to 20% of each 40 X (high powered) field. Lymphocytes and plasma cells extend to the deeper aspects of the mucosa where they surround crypts within the deep aspect of the lamina propria. Clusters of lymphocytes and plasma cells are present in up to 10 cells in small clusters in these areas. Low numbers of neutrophils and eosinophils are present within the lamina propria, with up to 5	F2973 Ileum (10 pieces and smaller fragments, one level) the sections extend from the mucosal surface to the deep lamina propria and do not include muscularis mucosa. There is moderate to marked crush artefact throughout the sections, affecting up to 50% tissue examined. Sections are of reasonable diagnostic quality. The mucosa of these sections exhibits moderate atrophy, there is extensive villous atrophy. Epithelial cells are extensively replaced by goblet cells (mucus metaplasia). In other areas, there is multifocal mucosal erosion and attenuation and in these areas there are low numbers of attendant neutrophils, lymphocytes and plasma cells. The lamina propria is expanded by bands of well differentiated fibrocytes which extend from the surface into the	F2974 Colon (12 pieces and smaller fragments, one level) these fragments extend from the surface to the deep lamina propria and occasionally include fragments of muscularis mucosa. There is multifocal mild crush artefact affecting less than 10% of the sections. The sections are of good diagnostic quality. The surface mucosa is lined by well differentiated columnar epithelial cells. The superficial lamina propria contains low numbers of lymphocytes and plasma cells. The mucosal crypts are mildly distorted and are arranged perpendicular to the mucosal surface influenced cases. In some of the sections, there is mild increase in superficial lamina propria fibrous sites. Fibrocytes extend around crypts in bands of up to 5 cells thick. Low numbers of eosinophils and neutrophils are scattered within the

				present, with up to 10 noted per 40 X field. Occasional clusters of lymphocytes are present in nodular aggregates and these compose less than 5% of the sections examined. In one of the more fragmented sections, increased numbers of neutrophils are present within the lamina propria, with up to 20 noted per 40 X field.	of each cell type noted per 40 X field. Lacteals are within normal limit and occasionally contain low numbers of inflammatory cells (draining).	deeper aspects of the lamina propria, extending around crypts in bands of up to 5 cells thick. Multifocal clusters of neutrophils are present within the lamina propria, associated with lesser numbers of lymphocytes and plasma cells. Crypts are often mildly distended with amphophilic fibrillar material, mixed with necrotic cellular debris and occasional mononuclear inflammatory cells. Within some of the sections, crypts have a contorted and convoluted appearance (hyperplasia).	collagenous stroma in these areas, with up to 10 cells noted per 40 X field.
1	BIOEC2015	RVB01	3		Duodenum; G39 (x1): ten sections are examined which are variably composed of mucosa and submucosa. Multifocally there is an infiltrate of low numbers of lymphocytes and plasma cells within the lamina propria. Multifocally, crypts are separated by a mild increase in proprial fibrous tissue. Rare villus lacteals are mildly dilated and there is a mild increase in epithelial goblet cells. There is no evidence of epithelial erosion or ulceration.	Ileum; G40 (x1): one section is examined which is composed of superficial villi tips. The section is too superficial to be graded. There is no evidence of epithelial erosion or ulceration in the mucosa which is present.	Colon; G41 (x1): fifteen sections are examined which are variably composed of mucosa and submucosa. Diffusely there is a mild increase in fibrous tissue within the lamina propria. Multifocally the lamina propria is expanded by extravasated erythrocytes (acute haemorrhage, interpretation, procedural). There is no evidence of epithelial erosion or ulceration.

1	BIOEC2015	RVB07	1	Stomach (seven pieces): The gastric mucosal biopsies have either fundic or pyloric morphology and are generally intact in the plane of section with a good density of glands. There are scattered lymphocytes and plasma cells in the superficial lamina propria and there is a little mucus and cellular debris on the mucosal surface. Neither neoplastic cells nor infective agents are recognised.	Duodenum (thirteen pieces): The duodenal mucosal biopsies demonstrate moderate villous atrophy and spatulation in association with heavy infiltration of the villous cores by plasma cells accompanied by smaller numbers of lymphocytes. Intravillous lymph lacteals are often dilated and a small proportion (less than 10%) of crypts are distended by exudate. There is a mild increase in goblet cell numbers in the villous epithelium and an associated mild increase in lymphocyte trafficking across that epithelium. Neither neoplastic cells nor infective agents are recognised.	Ileum (twelve pieces): The ileal mucosal biopsies demonstrate mild villous atrophy and moderate lymphoplasmacytic villous core infiltration in associated with marked mucosal oedema and lymph lacteal dilation. Villous goblet cell numbers are increased and there is excessive mucus on the mucosal surface. Neither neoplastic cells nor infective agents are recognised.	Colon (twelve pieces): The colonic mucosal biopsies are generally intact in the plane of section and have regularly spaced crypts. There is a marginal increase in number of plasma cells and lymphocytes in the lamina propria. Neither neoplastic cells nor infective agents are recognised.
1	BIOEC2015	RVB08	1	F3673 (x1) Stomach; Six sections are examined, comprising gastric mucosa and scant underlying mucosa (three fundic, three pyloric). Multifocally the superficial propria is congested. Occasional pyloric glands are separated by increased fibrous connective tissue.	F3674 (x1) Duodenum; Twelve sections are examined, comprising superficial fragments of duodenal mucosa and scant to absent underlying submucosa. Diffusely villous propria contains mildly increased numbers of lymphocytes and plasma cells.	F3675 (x1) Ileum; Ten sections are examined, comprising ileal mucosa and scant underlying submucosa. Multifocally villi are mildly reduced in length and lined by moderately increased numbers of goblet cells (mucoid metaplasia). Moderately increased numbers of lymphocytes are observed trafficking between enterocytes. Occasional villi contain mildly dilated (ectatic)	F3676 (x1) Colon; Ten sections are examined, comprising superficial fragments of colonic mucosa and scant to absent submucosa. Multifocally surface epithelium is attenuated and the subjacent propria is moderately oedematous and contains mildly increased numbers of lymphocytes and plasma cells.

1	BIOEC2 015	RVB08				lacteals. Multifocally the propria is markedly expanded by enlarged lymphoid follicles and villi contain high numbers of lymphocytes and plasma cells admixed with moderately increased numbers of eosinophils.	
1	BIOEC2 015	RVB08	3	G539(x1) Stomach; Eight fragments of stomach are examined, comprising six with pyloric and two with fundic mucosa and scant underlying propria. Multifocally, occasional glands are separated by increased connective tissue. Multifocally there are occasional clusters of lymphocytes and plasma cells within the superficial propria that do not infiltrate glandular structures. There are mildly increased lymphoid follicles occupying up to 10% of pyloric biopsy areas.	G540(x1) Duodenum; Three fragments are examined, comprising poorly orientated duodenal mucosa and scant underlying propria. There are mildly increased numbers of lymphocytes and plasma cells trafficking between villous epithelium. Multifocally the subjacent propria contains mild to moderately increased numbers of lymphocytes and plasma cells admixed with fewer eosinophils. Occasional crypts are dilated by eosinophilic cellular and karyorrhectic debris (crypt abscesses).		G541(x1) Colon; Eight fragments of colonic mucosa and scant underlying propria are examined. Multifocally surface enterocytes are mildly attenuated, while crypt enterocytes are mildly hyperplastic, with accompanying mild distortion of crypt architecture. Occasionally the superficial propria is mildly expanded (oedematous) or contains extravasated erythrocytes (haemorrhage, possibly procedural related). There are mildly increased numbers of lymphocytes and plasma cells with occasional clusters of up to ten neutrophils within the propria.
1	BIOEC2 015	RVB13	1	Stomach, G1670 x1. 8 sections are examined which comprise	Duodenum, G1671 x1. 12 sections are examined which	Ileum, G1672 x 1: 10 sections are examined which	Colon, G1673 x 1: 12 sections are examined which

				surface epithelium and lamina propria extending to the muscularis mucosa, there is mild crush artefact and they are of good diagnostic quality. There is multifocal mild attenuation of surface epithelium and one section is overlain by a dense mat of necrotic debris and fibrin. The lamina propria is moderately infiltrated with lymphocytes and plasma cells and moderately irregular glands are moderately separated with fibrous connective tissue. There is marked lympho-follicular hyperplasia.	comprise surface epithelium and lamina propria extending to the muscularis mucosa, there is minimal crush artefact and 4 are well oriented for examination, they are of good diagnostic quality. There are mildly increased numbers of intraepithelial lymphocytes and there is mild shortening and blunting of villous profiles. The lamina propria is moderately infiltrated with lymphocytes and plasma cells which separate crypts.	comprise surface epithelium and lamina propria extending to the muscularis mucosa, there is minimal crush artefact and 5 are well oriented for examination, they are of good diagnostic quality. There is mild villous stunting and a moderate increase in intraepithelial lymphocytes and a mild increase in goblet cells. The surface is covered with copious mucus admixed with inflammatory debris. The lamina propria is moderately infiltrated with lymphocytes and plasma cells.	comprise surface epithelium and lamina propria extending to the muscularis mucosa, there is minimal crush artefact and they are of good diagnostic quality. There is a mild increase in lamina propria lymphocytes and plasma cells, otherwise the sections are within normal limits.
1	BIOEC2015	RVB13	3	G2615: stomach (10 sections) these sections extend from the surface epithelium to the deep lamina propria and often include muscularis mucosa. There is multifocal moderate crush artefact affecting up to 30% of the sections. The sections are of good diagnostic quality. The surface is lined by a well differentiated tall columnar epithelial layer which shows multifocal marked attenuation. Multifocally, the surface	G2616: duodenum (12 sections and smaller fragments) these sections of small intestinal mucosa extend from the surface epithelium, often including deep lamina propria and occasionally muscularis mucosa. There is multifocal mild crush artefact affecting up to 50% of the sections. Sections of good diagnostic quality. Multiple villi profiles are evident and these show marked stunting, blunting and fusion. Villi are lined by tall columnar well	G2617: ileum (12 sections and smaller fragments) these are sections of small intestine and extend from the surface epithelium to the deep lamina propria, often including muscularis mucosa. There is multifocal mild crush artefact affecting less than 10% of the sections. Sections are of good diagnostic quality. Villi show multifocal= marked stunting, blunting and fusion. They are	G2618: Colon (10 sections and smaller fragments) the sections consist of colonic mucosa, extending from the surface epithelium to the deep lamina propria, and often including muscularis mucosa. There is multifocal mild crush artefact. The sections are of good diagnostic quality. There is multifocal mild attenuation of the surface epithelium. Multifocally, the epithelium is absent (ulceration).

				epithelium is absent (ulceration). Mild to moderately increased numbers of intraepithelial lymphocytes are present, with up to 30 cells noted per 40 X field in some areas. The underlying lamina propria contains high numbers of lymphocytes and plasma cells. These occupying up to 60% of the lamina propria per 40 X field. There are accompanied by low numbers of neutrophils and eosinophils and lesser histiocytes, which number up to 10 cells each per 40 X field. Mild diffuse fibrosis is present, with slender bands of fibrous connective tissue extending around gastric glands, in bands of up to 5 cells thick. Multifocal lymphoid aggregates are present and occupying up to 40% of the biopsies.	differentiated epithelial cells. Occasional segmental mild increases in intraepithelial lymphocytes is noted. There is diffuse mild to moderate increase in lamina propria lymphocytes and plasma cells, which occupying up to 60% of the lamina propria in some areas. These are accompanied by clusters of neutrophils and eosinophils, numbering up to 5 cells each per 40 X field. Low numbers of large granular lymphocytes are present in intraepithelial areas. Lacteal is are mildly ectatic, measure approximately one third of the diameter of the villous.	lined by tall columnar well differentiated epithelial cells. There is diffuse mild increase in intraepithelial lymphocytes. Lamina propria lymphocytes and plasma cells are a diffusely moderately increased in number, occupying up to 60% of the lamina propria in some fields. There are accompanied by high numbers of eosinophils, which number up to 20 cells per 40 X field, and lesser numbers of neutrophils, numbering up to 10 cells per 40 X field. There is multifocal marked lymphoid hyperplasia. Lymphoid follicles appear well differentiated, with prominent germinal centres containing large numbers of polygonal cells.	Subjacent to areas of attenuation, there are clusters of neutrophils, numbering up to 10 cells per 40 X field. There is diffuse mild increase in lamina propria macrophages, which number up to 20 cells per 40 X field. Frequent large granular lymphocytes are evident within intraepithelial areas. There is diffuse mild to moderate increase in lamina propria lymphocytes and plasma cells, often noted in clusters between the crypts in the deeper lamina propria. Crypts are generally orientated perpendicular to the mucosal surface. Occasionally, there are moderately to markedly convoluted and lined by several layers of epithelial cells. The occasionally mildly distended with small amounts of mucoid material.
1	BIOEC2 015	RVB16	1	G2168 Stomach (12 sections and smaller fragments) the sections consist of gastric mucosa and extend from the surface epithelium and often include the muscularis mucosa. There is multifocal mild crush	G2169 Duodenum (12 sections and some smaller fragments) these sections extend from the surface epithelium and occasionally include the muscularis mucosa. The majority of the sections extend to mid lamina propria.	G2170 Ileum (10 sections and smaller fragments) the sections extend from the surface mucosa and include lamina propria. There is multifocal mild crush artefact. There is	G2171 Colon (sections and some smaller fragments) the sections consist of colonic mucosa and extend from the surface epithelium to the deep lamina propria, or occasionally including

				artefact affecting up to 20% of the sections. Sections are of good diagnostic quality. The mucosa is lined by tall columnar epithelial cells. There is multifocal mild epithelial attenuation. There is multifocal moderate accumulation of surface mucus. Dense clusters of spiral 20 µm long bacteria are present within the surface mucus. There are low numbers of intraepithelial lymphocytes with 2 to 3 noted per 40 X field. Lymphoid follicles are present in the deep mucosa and account for up to 10% of the biopsy. The lamina propria is diffusely mildly expanded by clusters of lymphocytes and plasma cells. These occupying up to 60% of the lamina propria in some fields. The superficial lamina propria contains high numbers of parietal and chief cells. There is multifocal mild atrophy of these glands. In general, they are well differentiated. Multifocally, there is moderate increase in lamina propria fibrosis. In affected areas, bands of fibrocytes up to 5 cells thick extending between the glands.	Crypt architecture is evident in one of the sections. Villi villous morphology is often difficult to appreciate, however, when present in longitudinal sections, villi often moderately blunted to markedly pigmented and stunted. There is extensive villous fusion. Villi are generally lined by tall columnar epithelium. There is multifocal moderate epithelial attenuation. Expected numbers of intraepithelial lymphocytes are present. There is a mild diffuse increase in lamina propria lymphocytes, occupying up to 55% of each 40 X field. Clusters of lymphocytes and plasma cells extend between crypts and groups of up to 12 to 20 cells. Clusters of eosinophils are noted in groups of up to 20 per 40 X field. These are accompanied by approximately equal numbers of neutrophils, noted both within the superficial and deep lamina propria. There is multifocal moderate hyperplasia of the crypt epithelium with distortion of the epithelial cells and loss of perpendicular arrangement to the surface. Lacteals are present and appear within	multifocal mild crush artefact affecting up to 10% of the sections. Sections are of good diagnostic quality. There is multifocal moderate stunting and fusion. Moderately increased numbers of intraepithelial lymphocytes are present. There is diffuse moderate increase in lamina propria lymphocytes and plasma cells, which occupy up to 60% of each 40 X field in some areas. Low numbers of lymphocytes of neutrophils and eosinophils are present in the lamina propria, clusters of up to 20 per 40 X field in some areas. Is occasional clusters of macrophages are also present lamina propria.	muscularis mucosa. There is multifocal mild surface epithelial attenuation. Occasional areas of erosion and ulceration are noted. The lamina propria is expanded by moderately increased numbers of lymphocytes and plasma cells, which occupying up to 50% to occasionally 60% of the each 40 X field. occasional dense aggregates of lymphocytes and plasma cells are arranged in follicles within the sections. Epithelial cells lining crypts are generally well differentiated. There is multifocal mild crypt ectasia. Occasionally, markedly tortuous crypts are noted loss of perpendicular arrangement to the surface mucosa. Occasional neutrophils are noted within the lamina propria with 1 to 2 noted per 40 X field. Mildly accumulations of surface mucus are present in some areas

				Occasional clusters of neutrophils are scattered within the superficial lamina propria. In occasional fields, dense clusters of intraepithelial lymphocytes are present. A number up to 10 per 40 X field in these areas. Minimal disruption of the underlying basement membrane is noted.	normal limits. Occasional mild lymphatic lacteal ectasia is noted with occasional lacteal measuring = up to 50% of the diameter of the associated villous. occasional well formed lymphoid follicles are present within the superficial mucosa. Occasional areas of mucosal fibrosis noted with slender bands of fibrous connective tissue extending between the crypts. there is focal crush artefact in this area, with mild anisokaryosis and anisocytosis in the crypt epithelial cell population. There is multifocal mild crush artefact present in this area.		
1	BIOEC2 015	RVB16	3	G3084 (x1) Stomach (10 sections): The sections consist of pyloric and fundic gastric mucosa and extend from the surface epithelium to the lamina propria and does not include muscularis mucosa. There is multifocal mild crush artefact. The sections are variably orientated and are of good diagnostic quality. The mucosa is lined by tall columnar epithelial cells with no evidence of attenuation or degeneration. There is minimal surface mucus and spiral bacteria are not evident at the	G3085 (x1) Duodenum (8 sections and smaller fragments): The sections extend from the surface epithelium to the mid to deep lamina propria and do not include muscularis mucosa. Sections are variably orientated and there is mild to moderate crushing artefact. The samples are of adequate diagnostic quality. Where longitudinally sectioned villi are mildly stunted with wide bases. The villous epithelium is present in a single layer of tall columnar cells and erosion or ulceration is not observed. There is an	G3086 (x1) Ileum (9 sections and smaller fragments): The sections extend from the surface epithelium and include lamina propria, with one section including muscularis mucosa. There is multifocal mild to moderate crush artefact. Sections are of good diagnostic quality. Where longitudinally sectioned there is mild stunting of villi. On the mucosal surface are bacterial colonies admixed with increased volumes of mucus. There is a diffuse	G3087 (x1) Colon (8 sections): The sections extend from the surface epithelium to the deep lamina propria, frequently including muscularis mucosa. A single layer of columnar epithelium covers the surface and crypts and attenuation, erosion or ulceration is not noted. There is mild goblet cell hyperplasia with an excess of surface mucus present with mild thickening and distortion of crypts. There is

				mucosal surface.<br>Low numbers of intraepithelial lymphocytes are present, with 1-2 observed per high power field (400x).There is diffuse expansion of lamina propria by blood vessels (congestion) with a mild, diffuse increase in the number of lymphocytes and plasma cells. Cellular debris is noted within the lumen of a single gastric gland (gland abscess). Lymphoid follicles are present within the deep mucosa of multiple sections, accounting for <10% of the biopsy sections. There is a small to moderate amount of fibrous connective tissue (fibrosis) separating the gastric glands of the fundus in the superficial mucosa.	increased proportion of goblet cells within the surface epithelium. Intraepithelial cells are present at 2-3 per high power field (400x).The superficial lamina propria blood vessels are expanded by red blood cells (congestion) and crypts are occasionally distended with mucus, with rare crypts containing = fragments of deeply basophilic material (mineral). There is a mild increase in lymphocytes and plasma cells within the lamina propria and neutrophils are observed within vessels. Areas of lymphofollicular hyperplasia are present within multiple sections.	mild increase in lamina propria lymphocytes and plasma cells. Neutrophils, eosinophils or macrophages are not observed.	a mild increase in lamina propria lymphocytes and plasma cells with multifocally mildly increased separation of crypts by fibrous tissue.
1	BIOEC2015	RVB18	1	G2372 (×1): 13 fundic and pyloric gastric biopsies are examined. The epithelium lining the sections of fundus is diffusely intact and is multifocally infiltrated by scattered lymphocytes at a frequency of up to 2 cells per high power field stretch of epithelium. Gastric pits are lined by well-differentiated chief and parietal cells and towards the luminal surface	G2373 (×1): 12 biopsies of duodenal mucosa are examined. Mucosal villi lining these sections are tall and slender, and are lined by tall columnar epithelial cells with expected numbers of goblet cells present. Intraepithelial lymphocytes are present at a frequency of up to 10 cells per high-power field stretch. Lymphocytes and plasma cells are present within the	G2374 (x1): 11 biopsies of ileal mucosa are examined. Within these sections, = mucosal villi are tall and slender and are lined by tall columnar epithelial cells with expected numbers of goblet cells present. Increased numbers of intraepithelial lymphocytes are identified at a frequency of between 20 to 30 cells per HPF stretch	G2375 (x1): 10 colonic biopsies are examined. The epithelium overlying these sections is diffusely intact and is formed tall enterocytes and lesser numbers of goblet cells. Occasional intraepithelial lymphocytes are present, at a frequency of <1 cell per high power field stretch. Infiltrating the lamina propria are increased

				are separated by small amounts of oedema and small proliferations of fibrous connective tissue. Within the deeper aspects of the mucosa, fundic glands are separated by 1-2 layers of fibroblasts. Low numbers of lymphocytes and plasma cells multifocally infiltrate the lamina propria, at a frequency of up to 20 cells per high power field. Lymphoid aggregates occupy less than 10% of the biopsy area. The epithelium lining the sections of pylorus is diffusely intact and is multifocally infiltrated by scattered lymphocytes at a frequency of up to 2 cells per high power field stretch of epithelium. Pyloric glands are individualised by expected amounts of collagenous connective tissue. Infiltrating this fibrous stroma are expected numbers of lymphocytes and plasma cells at a frequency of up to 20 cells per high power field. Neutrophils and eosinophils are not identified. Lymphoid aggregates occupy less than 10% of the biopsy area.	lamina propria in expected numbers, representing up to 25% of the total tissue area with 1-2 lymphocytes and plasma cells present per intercrypt space. Eosinophils are present at a frequency of less than 2 per high-power field. Lacteals represent less than 25% of the total villous width.	of epithelium. There is prominent dilation of central lacteals and these lacteals occupy up to 75% of the villous width. Intestinal crypts are well organised and separated by expected amounts of fibrovascular connective tissue. Infiltrating the lamina propria are increased numbers of eosinophils at a frequency of between 10 to 20 cells per HPF and increased numbers of lymphocytes and plasma cells also infiltrate the lamina propria at a frequency of between 20 to 30 cells per HPF. Scattered neutrophils are also identified at a frequency of between 5 to 10 cells per HPF.	numbers of eosinophils at a frequency of between 10 to 20 cells per HPF and these cells are accompanied by mildly increased numbers of lymphocytes and plasma cells which filled the inter-crypt space but do not disrupt normal perpendicular crypt architecture. Scattered neutrophils are also identified at a frequency of between 5 to 10 cells per HPF. The epithelium lining the colonic crypts is mildly hyperplastic.

1	BIOEC2 015	RVB18	3	Stomach (one section): 11 sections of fundic and pyloric mucosa are examined. Infiltrating the lamina propria are moderately increased numbers of lymphocytes and plasma cells which are supported by increased proliferations of fibrovascular connective tissue. Together these changes cause separation of the gastric glands	Duodenum (one section): 12 sections of duodenal mucosa are examined. The villi overlying these sections are tall and slender and are lined by intact epithelium. Infiltrating the lamina propria are mildly increased numbers of lymphocytes and plasma cells.	Ileum (one section): 4 sections of ileal mucosa are examined. Increased numbers of lymphocytes and plasma cells multifocally infiltrate the lamina propria and are variably separated by moderate amounts of oedema. Overlying the epithelial surface are moderate amounts of mucin	Colon (one section): 13 sections of colonic mucosa are examined. The epithelium overlying these sections is variably eroded and infiltrating the underlying lamina propria are numerous eosinophils admixed with fewer lymphocytes, plasma cells and scattered neutrophils. These inflammatory infiltrates are separated by moderate amounts of oedema. Goblet cells lining the mucosal glands are almost entirely devoid of mucus.
1	BIOEC2 015	RVB27	1	H686, stomach x1. Eleven sections are examined, they extend from the surface epithelium to the muscularis mucosa, there is minimal crush artefact and they are of good diagnostic quality. There is a mild increase in mucosal fibrous connective tissue with nesting of adjacent glands. There is a mild increase in superficial lymphocytes and plasma cells.	H687, duodenum x1. Thirteen sections are examined, they extend from the surface epithelium to the muscularis mucosa, their orientation is adequate and there is minimal crush artefact, they are of good diagnostic quality. Sample orientation hinders examination of villous morphology but where this is appreciable, they are within normal limits. The villous lamina propria is mildly infiltrated by low numbers of plasma cells and lymphocytes. Otherwise the		H688, colon x1. Thirteen sections are examined, they extend from the surface epithelium to the muscularis mucosa, the orientation is good and there is minimal crush artefact, they are of excellent diagnostic quality. There is rare attenuation of the surface epithelium. Multifocally, the lamina propria is infiltrated by low numbers of lymphocytes and plasma cells.

					sections are morphologically unremarkable.		
1	BIOEC2 015	RVB29	1	H956 (x1) Stomach (9 sections): The samples consist of 3 sections of pyloric and 6 sections of fundic gastric mucosa and extend from the surface epithelium to the lamina propria. There is multifocal mild crush artefact and acute haemorrhage presumed to be procedure related. The sections are variably orientated and are of good diagnostic quality. Within fundic sections the surface epithelium is multifocally and extensively flattened (attenuated) and there is increased separation of gastric glands by loosely arranged fibrous tissue (oedema, fibrosis and atrophy). Affecting both fundic and pyloric sections there is a diffuse increase in lamina propria lymphocytes and plasma cells and low numbers of neutrophils are observed. Intraepithelial lymphocyte numbers are considered within normal limits	H957 (x1) Duodenum (12 sections and fragments): The sections extend from the surface epithelium to the mid to deep lamina propria and rarely contain fragments of muscularis mucosae. Prominent gut associated lymphoid tissue is present within the sections. Sections are variably orientated and there is mild to moderate crushing artefact. The samples are of good diagnostic quality. Villi appear of adequate length where longitudinally sectioned and the villous epithelium is present in a single layer of tall columnar cells. No erosions or ulcerations are observed. Intraepithelial lymphocytes are present within normal limits. There is a moderate increase in lymphocytes and plasma cells within the lamina propria and occasional neutrophils are present. Central lymphatics are within normal limits.		H958 (x1) Colon (13 sections): The sections extend from the surface epithelium to the deep lamina propria, occasionally including the muscularis mucosa. The sections are variably orientated and of good diagnostic quality. The surface epithelium is intact and the number of lymphocytes and plasma cells within the lamina propria is considered within normal limits.
1	BIOEC2 015	RVB29	3				
1	BIOEC2 015	RVB32	1	H1415, stomach x1. Fourteen sections of pyloric and gastric	H1416, duodenum x1. Eight sections are examined, they	H1417, ileum x1. One section is examined, it	H1418, colon x1. Eleven sections are examined which

				morphology are examined, they extend from the surface epithelium to the muscularis mucosa, there is minimal crush artefact and they are of good diagnostic quality. There is no significant inflammation, erosion, ulceration, neoplasia and there are no microorganisms detected in the examined sections.	extend from the surface epithelium to the muscularis mucosa, they are well orientated and are of good diagnostic quality. The villous propria is infiltrated by low numbers of lymphocytes and plasma cells and fewer eosinophils which also surround and separate crypts. There is no evidence of neoplasia in the examined sections.	comprises transverse sections of villi, it is not adequate for examination, within these limits there is no significant inflammation, erosion, ulceration, neoplasia and there are no microorganisms detected.	extend from the surface epithelium to the muscularis mucosa, the lamina propria is infiltrated by moderate numbers of lymphocytes, plasma cells and eosinophils. There is no evidence of neoplasia in the examined sections.
1	BIOEC2015	RVB32	3		Duodenum, H2302 (x1); seven pieces of tissue representing duodenal mucosa are examined, one of which includes muscularis mucosa. The sections are variably orientated with mild, multifocal crush artefact, and are of good diagnostic quality. Where longitudinally sectioned villi appear tall and slender and the surface of the tissues is covered by a single layer of tall columnar epithelial cells with few goblet cells, without evidence of ulceration or attenuation. The number of intraepithelial lymphocytes is considered within acceptable limits and the number of lamina propria lymphocytes and plasma cells is mildly to moderately increased.		Colon, H2303 (x1); eight pieces of colonic mucosa which frequently extend to and include muscularis mucosa are examined. The sections are variably orientated and of good diagnostic quality with multifocal crush artefact. The surface of the tissues is covered by a single layer of epithelium overlying which is a moderate amount of mucus admixed with sloughed epithelial cells. Ulceration or attenuation is not apparent. The number of lymphocytes and plasma cells within the lamina propria is considered within acceptable limits and there

					Occasional eosinophils are observed within the lamina propria. Villous lacteals, where visible, are not considered dilated beyond normal limits and there is no apparent crypt distension or increased fibrous tissue within the lamina propria.		are multifocal, large lymphoid aggregates throughout the tissue sections. Occasional individual scattered eosinophils are observed. Lamina propria blood vessels are multifocally congested (possible sampling artefact).
1	BIOEC2 015	RVB35	1	H3552 Stomach x1<br>11 sections of well orientated, partial thickness fundic and pyloric gastric mucosa, with minimal crush artefact, are examined. Helical bacteria (Helicobacter spp.) are multifocally present in the mucus layer of the pylorus	H3553 Duodenum x1<br>11 sections of well orientated, partial thickness duodenal mucosa, with minimal crush artefact, are examined. The lamina propria contains a moderately increased number of lymphocytes, plasma cells, macrophages and neutrophils and crypts are multifocally expanded by necrotic debris in the lumen (crypt abscesses). Villi are multifocally blunted and fused and lacteals are multifocally, minimally dilated.		H3554 Colon x1<br>11 sections of poorly orientated, partial thickness colonic mucosa, with minimal crush artefact, are examined. The sections show a mild multifocal increase in lamina proprial lymphocytes and plasma cells.
1	BIOEC2 015	RVB35	3	Stomach, J663 x 1: Eleven tissue sections and fragments from fundic and antral mucosa are examined. The majority are of good diagnostic quality and variably extend from the mucosa to the deep lamina propria and occasionally to the muscularis mucosa. The mucosa is lined by well-differentiated tall-	Duodenum, J664 x 1: Ten tissue sections and fragments are examined. They consist of longitudinal and transverse sections that extend from the mucosa to the deep lamina propria and occasionally to the muscularis mucosa. There is diffuse mild shortening and widening of villous profiles with occasional mild dilation of		Colon, J665 x 1: Twelve tissue sections and fragments are examined. They are of good diagnostic quality and include from the mucosa to the muscularis mucosa. The lining epithelium does not exhibit evidence of erosion. The lamina propia contains minimally increased

				columnar epithelium, covered by a small amount of pale basophilic material (mucous secretion) in which are embedded moderate numbers of Helicobacter spp. bacteria. Multifocally the mucosa exhibits small haemorrhages (sampling related). Numbers of proprial lymphocytes and plasma cells are within normal limits. There is no evidence of erosion, ulceration, significant inflammation or neoplasia within the examined tissues.	lacteals. The lamina propria contains mildly increased numbers of lymphocytes and plasma cells. Very rarely crypts are dilated and contain eosinophilic fluid with embedded degenerate neutrophils (crypt abscesses). There is no evidence of infections agents or neoplasia within the examined sections.		numbers of lymphocytes and plasma cells which produce minimal distortion of the crypts.

TABLE A45. Clinical pathology results - Group 1. Diagnosis and comments.

Group	Treatment	Patient No.	Visit No.	Tissue - Microscopic Diagnosis	Tissue - Pathologist Comment	Tissue - Investigator Comment
1	BIOEC2015	RVB01	1	Gastritis, lymphocytic, plasmacytic, neutrophilic, eosinophilic, multifocal, mild with mucosal fibrosis, multifocal, moderate; stomach. Enteritis, neutrophilic, eosinophilic, multifocal, mild with mucosal atrophy, multifocal, mild; duodenum. Enteritis, neutrophilic, lymphoplasmacytic ulcerative and erosive, multifocal to coalescing, moderate with mucosal fibrosis and atrophy, multifocal, moderate; ileum. Colitis, eosinophilic, neutrophilic, multifocal, mild with mucosal fibrosis, multifocal, moderate; colon	There are extensive chronic active changes present within these sections of stomach and intestines. Mixed cell types are present and would indicate a chronic hypersensitivity is most likely occurring. The most prominent inflammation is occurring in the ileum, where there are dense clusters of neutrophils present within the lamina propria. This likely indicates a bacterial infection in this site. At this site.<br>I did not detect the presence of neoplasia, or microbes in these sections.	Consistent with Inflammatory Bowel disease
1	BIOEC2015	RVB01	3	Duodenum; mild lymphoplasmacytic infiltration with mild mucosal fibrosis, chronic. Ileum; sample non-diagnostic Colon; mild mucosal fibrosis, chronic	Histopathological examination of the received tissues reveals the mild chronic changes within the duodenum and the colon. There was no evidence of neoplasia in the examined sections.	Still signs of inflammatory bowel disease with chronic changes

1	BIOEC2015	RVB07	1	Small intestine, duodenum and ileum: Enteritis, lymphoplasmacytic,<br>moderate to marked, with villous atrophy and lymph lacteal dilation.<br>Stomach: No significant abnormality recognised. Colon: No significant abnormality recognised.	Endoscopic biopsy of the proximal and distal small intestine reveals significant inflammatory and villous atrophic changes that are likely to be of functional significance at both sites. The prominent lymph lacteal dilation may also be a proxy marker for associated lymphangiectasia involving the deeper layers of the small intestinal wall. Marginal inflammatory changes that are barely out with the normal range for an adult dog, are present in the stomach and colon and are not likely to be of functional significance.	Consistent with IBD
1	BIOEC2015	RVB08	1	MICROSCOPIC DIAGNOSIS:<br>1) Stomach; Multifocal, mild, pyloric, mucosal fibrosis<br>2) Duodenum; Diffuse, mild, lymphoplasmacytic proprial infiltrate<br>3) Ileum; Diffuse, moderate, lymphoplasmacytic and eosinophilic ileitis, with moderate mucoid metaplasia and mild villous stunting<br>4) Colon; Multifocal, mild, lymphoplasmacytic colitis, with moderate oedema<br>5) Caecum; Multifocal, mild, lymphoplasmacytic typhlitis, with moderate oedema	Microscopic examination reveals significant inflammation within the submitted sections of ileum. There is moderate lymphoplasmacytic and eosinophilic ileitis accompanied by mild villous stunting and moderate mucoid metaplasia. This is likely to be functionally significant. A mild lymphoplasmacytic colitis and typhlitis are also present, accompanied by moderate oedema. The oedema may be inflammatory in origin or related to hypoproteinaemia. The duodenal proprial infiltrate is not accompanied by architectural changes and therefore its significance is unclear. The gastric mucosal fibrosis is mild and unlikely to be of significance. A cause for the ileal, caecal and colonic inflammation is not observed in these sections:<br>micro-organisms, parasites and foreign bodies are not identified. Neoplasia is also not identified but its presence out with the submitted samples cannot be excluded.	Results consistent with IBD

1	BIOEC2015	RVB08	3	1) Stomach; multifocal mild, proprial fibrosis, mild proprial lymphoplasmacytic infiltration and mild pyloric lymphoplasmacytic follicular hyperplasia<br>2) Duodenum; multifocal, mild to moderate, lymphoplasmacytic and eosinophilic duodenitis, with mild cryptal distension<br>3) Colon; multifocal, mild, lymphoplasmacytic and neutrophilic colitis, with mild epithelial attenuation, proprial oedema and fibrosis	Histopathological examination reveals mild lymphoplasmacytic follicular hyperplasia within the pyloric sections examined, accompanied by a mild lymphoplasmacytic proprial infiltration and mild proprial fibrosis. There is no evidence of direct surface epithelial damage indicative of gastritis however; therefore this response is non-specific and unlikely to be functionally significant. There are more significant changes present within the duodenum consisting of a mild to moderate lymphoplasmacytic and eosinophilic duodenitis. There is an absence of significant epithelial injury or villous changes but cryptal distension is present. A cause for these duodenal changes is not apparent in the examined sections but the presence of eosinophils is consistent with hypersensitivity, food allergy or endoparasitism (parasites are not observed). There is a mild lymphoplasmacytic and neutrophilic colitis accompanied by mild epithelial attenuation and superficial proprial oedema and fibrosis. The cause for this is uncertain; micro-organisms were not observed. Neoplasia was not observed in any off the submitted tissues however its presence out with the examined sections cannot be excluded.	Consistent with IBD
1	BIOEC2015	RVB13	1	Stomach, marked diffuse chronic lymphoplasmacytic gastritis<br>Ileum and duodenum, moderate diffuse chronic lymphoplasmacytic enteritis<br>Colon, minimal lymphoplasmacytic colitis	Histopathological examination of the submitted sections reveals significant changes in the stomach and small intestine. The changes in the stomach are the most severe. The inciting cause of these changes, especially given the duration of the clinical signs is not clear from these sections, but we have no evidence of neoplasia or infectious organisms in the examined sections. The gastric lymphofollicular hyperplasia is striking,	Consistent with IBD

					but may just indicate the chronicity of the disease process.	
1	BIOEC2015	RVB13	3	G2615: Gastritis, ulcerative and erosive, lymphoplasmacytic, neutrophilic, eosinophilic, histiocytic, multifocal, marked, chronic with lymphoid hyperplasia, multifocal, marked; stomach G2616:Enteritis, lymphoplasmacytic, eosinophilic, neutrophilic, multifocal to coalescing, moderate, chronic with villous atrophy, multifocal, marked; duodenum G2617: Ileitis, lymphoplasmacytic, neutrophilic, eosinophilic, multifocal to coalescing, marked, chronic with mucosal atrophy, multifocal, moderate; ileum G2618: Colitis, lymphoplasmacytic, neutrophilic, histiocytic, multifocal, marked, chronic ulcerative; Colon	The appearance of these sections is consistent with chronic gastroenteritis. The most severely affected areas are the stomach, and to a lesser extent the duodenum and ileum and colon. The inflammatory cell types present in the stomach, duodenum and ileum are mixed. Prominent numbers of eosinophils are present, potentially indicating a hypersensitivity type disorder, which could be consistent with IBD, as noted clinically. Low to moderate numbers of neutrophils are present at all sites, likely indicating a secondary bacterial infection is occurring. Increased numbers of large granular lymphocytes are noted in the duodenum and ileum, likely indicating an innate immune response to luminal antigens. There are low numbers of macrophages in the lamina propria in the stomach and colon. This likely indicates a "clean-up" process is taking place. Perhaps FISH could be ordered on the colonic sections, although the appearance is not typical of histiocytic ulcerative colitis. There was no evidence of microbial pathogens in these sections and no evidence of neoplasia was noted.	Consistent with IBD

1	BIOEC2015	RVB16	1	G2168: gastritis, lymphoplasmacytic, neutrophilic, multifocal, moderate, chronic, with mucosal fibrosis, multifocal, moderate; stomach<br>G2169: enteritis, lymphoplasmacytic, eosinophilic, neutrophilic, multifocal to coalescing, moderate, chronic; duodenum<br>G2170: enteritis, lymphoplasmacytic, neutrophilic, eosinophilic, multifocal to coalescing, chronic; ileum<br>G2171: colitis, lymphoplasmacytic,, multifocal to coalescing, moderate, chronic with mucosal fibrosis, multifocal, moderate neutrophilic; colon	There is evidence of chronic gastroenteritis in these sections. The more severe changes are within the duodenum, ileum and colon, which would correlate with the clinical signs. The severity of the inflammation present would fit with a protein losing enteropathy. The inflammatory cells present at these sites are mixed. There are moderately increased numbers of eosinophils present within the stomach, duodenum and the ileum, which may indicate a hypersensitive=ty type disorder. There are also moderately increased numbers of neutrophils present at both sites, indicative of a secondary bacterial infection. I did not detect evidence of microbial pathogens<br>in these sections. Neither was there evidence of neoplasia present.	Consistent with IBD
1	BIOEC2015	RVB16	3	G3084, stomach; mild, diffuse, lymphoplasmacytic gastritis with mucosal fibrosis G3085, duodenum; chronic, mild, diffuse, lymphoplasmacytic duodenitis with mild crypt distension and mineralisation G3086, ileum; mild, diffuse, lymphoplasmacytic ileitis<br>G3087, colon; chronic, mild, diffuse, lymphoplasmacytic colitis with mild mucosal fibrosis and crypt distortion	There is evidence of chronic, mild, lymphoplasmacytic gastroenteritis throughout the examined sections, consistent with an ongoing inflammatory bowel disease. Neutrophils or eosinophils within the lamina propria are not identified and there is no evidence of microbial organisms or neoplasia identified. The slides from endoscopic biopsies submitted in 07/16 have been reviewed and compared with the current submission. Progression of the inflammatory and/or fibrosing disease process is not apparent and there is overall decrease in the amount and type of the inflammatory cell component	

1	BIOEC2015	RVB18	1	1. G2372 (×1): Within normal limits, fundus and pylorus, stomach<br>2. G2373 (×1): Within normal limits, duodenum<br>3. G2374 (x1): Enteritis, eosinophilic, chronic, multifocal, moderate with lymphangiectasia, multifocal, moderate, ileum<br>4. G2375 (x1): Colitis, eosinophilic, chronic, multifocal, moderate, colon	The ileal and colonic mucosa of this dog is chronically inflamed and the mixed, eosinophil-rich character of the inflammatory infiltrate is most consistent with a chronic hypersensitivity reaction. Such reaction is not specific to a particular cause and may be reactive to either ingested antigens, enteric parasites, changes within the microbiome or reflect a more generalised inflammatory reaction as encompassed by the term 'inflammatory bowel disease'. There is variable oedema of the lamina propria which may potentially reflect increased gastrointestinal permeability secondary to the inflammation within these tissues or enteric albumin loss. The<br>sections of stomach and duodenum are within normal limits. There is no evidence of neoplasia within the examined sections and microorganisms are not identified.	Clinically consistent with IBD
1	BIOEC2015	RVB18	3	Gastritis, lymphocytic to plasmacytic, chronic, multifocal, moderate, stomach. Enteritis, lymphocytic to plasmacytic, chronic, multifocal, mild, duodenum and ileum. Colitis, eosinophilic, chronic, multifocal, marked, colon.	These samples of gastrointestinal mucosa are chronically inflamed with the most severe inflammation affecting the colon. The eosinophil rich character of the inflammatory infiltrate within the colon is most suggestive of a reaction against either ingested antigens or enteric parasites. There is no evidence of neoplasia within the examined sections.	Consistent with IBD
1	BIOEC2015	RVB27	1	Stomach; minimal lymphoplasmacytic gastritis with mild mucosal fibrosis<br>Duodenum; mild lymphoplasmacytic duodenitis<br>Colon; minimal lymphoplasmacytic colitis	Histopathological examination reveals a range of mild inflammatory changes in the gastrointestinal tract of this dog. There is no evidence of neoplasia or the presence<br>of microorganisms in the examined sections. If other causes have been ruled out, the histological changes are potentially consistent with IBD as a cause of the clinical<br>signs in this dog	

1	BIOEC2015	RVB29	1	Stomach; diffuse, mild lymphoplasmacytic and neutrophilic gastritis with mild surface epithelial injury and glandular atrophy affecting the fundus Duodenum; moderate lymphoplasmacytic and neutrophilic duodenitis Colon; histologically normal	Microscopic examination of the submitted gastrointestinal endoscopic biopsies identifies a mild to moderate lymphoplasmacytic and neutrophilic inflammatory infiltrate within the stomach and duodenum. In addition, there is evidence of surface epithelial injury and glandular atrophy within the fundic region of the stomach. These changes are nonspecific and may be indicative of an inflammatory bowel disease type process. Some of the changes may also be partly attributable to the repeated and chronic vomiting episodes. The sections of colon appear histologically normal and there is no evidence of neoplasia within any of the examined tissue sections.	Consistent with IBD
1	BIOEC2015	RVB29	3	Duodenum; mild lymphoplasmacytic duodenitis. Colon; histologically unremarkable	There is moderate presumptive freeze-thaw artefact affecting the tissues with retraction of crypt epithelium from the basement membrane. There is also some loss of cellular detail. Within these limits it is possible to appreciate the cellularity and usually the cell types present within the tissues. Assessment of lacteals given the artefactual changes becomes more challenging to accurately assess. Sections available here are also orientated in such a way that accurate assessment of villous morphology is precluded. In this case there appears to be a mild lymphoplasmacytic duodenitis, the colon is considered histologically unremarkable. I have noticed that endoscopic biopsies were assessed histologically from this dog (P303949) in April, it would be interesting to know if these samples were obtained at the same time. Please do not hesitate to contact me should you wish to discuss this further (6265, pittaway@rvc.ac.uk). These samples, within the limits of freeze-thaw artefact,	

					identify a mild lymphoplasmacytic duodenitis, compared to a moderate lymphoplasmacytic and neutrophilic duodenitis that was diagnosed from samples submitted in April. This may suggest an improvement in the degree of inflammation present in the duodenum. These results, however, may not be directly comparable and should be interpreted in light of the fact that only two small fragments are available to= be assessed in these most recent samples, compared to 12 sections in April, which is therefore less reliably representative. In addition, the prior freezing of the most recent sample potentially makes the identification of specific subtle features, such as the presence of individual neutrophils, challenging. The colon in both sets of samples is considered histologically normal.	
1	BIOEC2015	RVB32	1	Stomach; no significant changes<br>Duodenum; mild lymphoplasmacytic and eosinophilic duodenitis<br>Ileum; no significant changes<br>Colon; mild to moderate lymphoplasmacytic and eosinophilic colitis	Histopathological examination of the submitted sections reveals potentially significant inflammatory changes in the duodenum and colon of this dog. The character of the inflammation is potentially consistent with eg a dietary hypersensitivity if this is compatible with the clinical picture in this case.	Clinical and histopathological evidence of inflammatory change in duodenum and colon
1	BIOEC2015	RVB32	3	Duodenum; mild to moderate lymphoplasmacytic duodenitis<br>Colon; histologically unremarkable	Microscopic examination of the submitted duodenal and colonic endoscopic biopsies is carried out. This identifies a mild to moderate lymphoplasmacytic duodenitis. The colon is considered histologically unremarkable. The changes within the duodenum would be consistent with an inflammatory bowel disease providing other causes have been excluded. There is no evidence of neoplasia or of infectious organisms within the examined tissue sections.	

1	BIOEC2015	RVB35	1	Stomach, pylorus: Occasional Helicobacter spp.<br>Duodenum: Moderate, diffuse lymphoplasmacytic and suppurative duodenitis<br>Colon: Minimally increased proprial lymphocytes and plasma cells	The only significant changes in the sections examined are in those from the duodenum. The duodenal lamina propria is expanded by a mixed population of inflammatory cells, making inflammatory bowel disease the most likely diagnosis. Lacteal dilation is relatively minimal in the sections examined, but the villi are somewhat distended by the proprial infiltrate which may have resembled lacteal dilation on endoscopy; alternatively, the dilation may be deeper in the submucosa, which is not sampled. Despite the endoscopic description of severe inflammation in the colon on endoscopy, there is only a minimal increase in inflammatory cells in the sections we have examined, though these may not be representative of the whole colon.	
1	BIOEC2015	RVB35	3	MICROSCOPIC DIAGNOSES: =<br>1. Stomach; moderate numbers of luminal Helicobacter spp.<br>2. Duodenum; multifocal, mild, chronic lymphoplasmacytic duodenitis<br>3. Colon; diffuse, chronic, minimal lymphoplasmacytic inflammation	Microscopic examination reveals mild inflammatory changes affecting the duodenal mucosa and colon. These changes, in-keeping with the reported history of good response to dietary modification, possibly suggest dietary hypersensitivity as the primary problem rather than true IBD. However, as IBD is usually a diagnosis of exclusion, a mild form cannot be ruled out. There is no evidence of neoplasia or infectious agents in the examined tissues.	

TABLE A46. Clinical pathology results -Group 2.

Group	Treatment	Patient No.	Visit No.	Tissue - Microscopic description - Stomach	Tissue - Microscopic description - Duodenum	Tissue - Microscopic description - Ileum	Tissue - Microscopic description - Colon
2	Placebo	RVB02	1	Gastric fundus and antrum; F2997 (x1): 10 good quality endoscopic biopsies representing superficial and deep fundic and pyloric mucosa are examined. Occasional Helicobacter-type organisms are observed on the epithelial surface. Multifocally, gastric glands are diffusely separated by clear space (oedema) within the lamina propria (sampling artefact).	Duodenum; F2998 (x1): Eleven well orientated endoscopic biopsies of duodenal mucosa including partial and full length villi fragments are examined. Where longitudinally sectioned, villus morphology is within normal limits. The lamina propria is infiltrated by low to moderately increased numbers of lymphocytes and plasma cells.	Ileum; F2999 (x1): Ten well orientated endoscopic biopsies of superficial and full thickness mucosa are examined. Where sectioned longitudinally villi appear mildly shortened with broad bases (atrophy). There is mild cryptal enterocyte hyperplasia. Within the lamina propria, there is a mild, multifocal increase in eosinophils.	Colon; F3000 (x1): Ten, well orientated endoscopic biopsies which extend to the lamina muscularis are examined. Multifocally, epithelium is mildly attenuated and the lamina propria is mildly expanded by clear spaces (oedema). There is a mild increase in eosinophils within the superficial lamina propria.
2	Placebo	RVB02	3		Duodenum; G49 (x1): ten sections are examined which are composed of poorly orientated superficial mucosa. There is no evidence of mucosal erosion or ulceration. The lamina propria is infiltrated by low numbers of lymphocytes and plasma cells.	Ileum; G50 (x1): two sections are examined which are composed of superficial samples which are subject to significant squash artefact. On the limited mucosa available for examination there is no evidence of erosion or ulceration. Occasional intraepithelial lymphocytes are present. The lamina propria and crypt and villi architecture are unable to be assessed in these sections.	Colon; G51 (x1): twelve sections are examined which are composed of mucosa and lamina propria. There is no evidence of mucosal erosion or ulceration. Diffusely there are scattered intraepithelial lymphocytes. The lamina propria is diffusely infiltrated by low numbers of neutrophils, lymphocytes and plasma cells and crypts are separated by a mildly increased amount of fibrous tissue. Multifocally crypts are mildly hyperplastic.

2	Placeb o	RVB05	1	F3398 (x1) Stomach: 8 sections of fundic and pyloric mucosa are examined. The epithelium overlying these sections is diffusely intact. Intraepithelial lymphocytes are present in increased numbers at a frequency of up to 5 cells per high-power field stretch of epithelium. Mucosal glands are separated by mild proliferations of fibrous connective tissue and this fibrous stroma is multifocally infiltrated by moderately increased numbers of lymphocytes and plasma cells at a frequency of up to 50 cells per HPF. Expanding the superficial submucosa are moderate amounts of oedema. Eosinophils are present at a frequency of up to 2 cells per HPF and neutrophils are not identified. Lymphoid aggregates occupy up to 25% of the biopsy area (marked hyperplasia).	F3399 (× 1) Duodenum: Villi within these sections are reduced to approximately 75% of normal height and overlaid by diffusely intact tall columnar epithelial cells. Intraepithelial lymphocytes are present at a frequency of up to 20 cells per HPF stretch of epithelium. Central lacteals are dilated and occupy up 50% of the villous width. Up to 25% of the intestinal crypts are dilated and contains abundant accumulations of amorphous eosinophilic material admixed with degenerate cellular debris (crypt abscesses). Expanding the lamina propria are moderately increased numbers of lymphocytes and plasma cells which occupy up to 75% of the area per HPF. Up to 10 cells are present between the crypts. Approximately 5 eosinophils are identified per HPF and neutrophils are not identified.	F3400 (x1) Villi within these sections are reduced to approximately 50% of normal height and overlaid by diffusely intact tall columnar epithelial cells. Intraepithelial lymphocytes are present at a frequency of up to 20 cells per HPF stretch of epithelium. Central lacteals are markedly dilated and occupy up 75% of the villous width. Up to 25% of the intestinal crypts are dilated and contains abundant accumulations of amorphous eosinophilic material admixed with degenerate cellular debris (crypt abscesses). Expanding the lamina propria are moderately increased numbers of lymphocytes and plasma cells which occupy up to 50% of the area per = HPF. Up to 5 cells are present between the crypts. Approximately 5 eosinophils are identified per HPF and neutrophils are not identified.	F3401 (×1) Colon: The epithelium overlying these sections is diffusely intact and is formed of tall columnar epithelial cells. Colonic crypts are well organised and are separated by expected amounts of fibrous connective tissue. Infiltrating the lamina propria are mildly increased numbers of lymphocytes and plasma cells. Eosinophils and neutrophils are not identified.
2	Placeb o	RVB09	1	Gastric fundus and antrum; G268 (x1): Nine fragments of tissue representing superficial and deep fundic and pyloric mucosa are examined. Occasionally gastric glands are diffusely separated by clear	Duodenum; G269 (x1): Eleven variably orientated endoscopic biopsies of duodenal mucosa including partial and full length villi fragments are examined. Where longitudinally sectioned, villi are markedly	Ileum; G270 (x1): Thirteen variably orientated endoscopic biopsies of superficial mucosa are examined. Where sectioned longitudinally villi appear markedly shortened and fused	Colon; G271 (x1): Ten, variably orientated endoscopic biopsies representing superficial and deep colonic mucosa are examined. The lamina propria is mildly expanded

				space (oedema) within the lamina propria (sampling artefact).	reduced in length and frequently fused. The lamina propria is infiltrated by moderately increased numbers of lymphocytes, plasma cells and occasional neutrophils and crypts are multifocally distended and contain clusters of viable and degenerate neutrophils.	with broad bases (atrophy). Within the lamina propria, there is a moderate, diffuse increase in lymphocytes, plasma cells and histiocytes.	by clear spaces (oedema) and there is a diffuse increase in lymphocytes and plasma cells.
2	Placeb o	RVB11	1	Stomach, G338 (x1): Nine sections of stomach of good diagnostic quality are examined, the largest measuring 2mm x 2mm. There is mild expansion of the lamina propria by clear spaces (oedema) and separation of glands by a very mild increase in fibrous connective tissue. There is no overt evidence of epithelial injury, inflammation or neoplasia within the examined sections.	Duodenum, G339 (x1): Eleven sections of duodenum of good diagnostic quality are examined, the largest measuring 3mm x 2mm. There is multifocal necrosis of the epithelium and lamina propria, associated with infiltration by moderate numbers of neutrophils. There is villus blunting and fusion and the lamina propria is infiltrated by moderate to large numbers of lymphocytes and plasma cells and lesser numbers of neutrophils and eosinophils. There are multifocal crypt abscesses. Intra-epithelial large lymphocytes are observed within crypt epithelium. Overlying the epithelial surface is a moderate increase in basophilic material (mucus) admixed with mixed bacterial colonies.	Ileum, G340 (x1): Ten sections of ileum of good diagnostic quality are examined, the largest measuring 4mm x 2mm. There is a moderate increase in the number of lymphocytes and plasma cells, with low numbers of neutrophils, within the lamina propria. There is multifocally extensive, mild to moderate, lacteal ectasia. Moderate numbers of crypt abscesses are observed multifocally. Overlying the epithelial surface is a moderate increase in basophilic material (mucus) admixed with mixed bacterial colonies.	Colon, G341 (x1): Fourteen sections of colon are examined. There is mild crypt hyperplasia. Two lymphoid follicles are observed. The overlying mucus contains low numbers of mixed bacterial colonies.

2	Placebo	RVB11	3	Stomach; G1127 (x1): ten sections are examined which are variably composed of fundic and pyloric mucosa, lamina propria and muscularis mucosa. The majority of sections are of diagnostic quality. Most sections examined are within normal limits, with no evidence of mucosal erosion or ulceration, inflammation or neoplasia. Occasionally there is very mild superficial proprial oedema. In one section there is a mildly hyperplastic lymphoid follicle.	Duodenum; G1128 (x1): fourteen sections are examined which are variably composed of mucosa and lamina propria. Approximately half of the sections are of diagnostic quality with the other half being either too small or superficial. There is diffuse mild blunting of villi and multifocal mild lacteal ectasia. There is no evidence of mucosal erosion or ulceration, infection or neoplasia. The lamina propria is diffusely infiltrated by a minimally increased lymphocytes and plasma cells.	Ileum; G1129 (x1): nine sections are examined which are variably composed of mucosa and lamina propria. The majority of sections are of diagnostic quality. There is no evidence of mucosal erosion or ulceration, infection or neoplasia. Multifocally there is mild lacteal ectasia and the lamina propria is diffusely infiltrated by minimally increased lymphocytes and plasma cells	Colon; G1130 (x1): eleven sections are examined which are variably composed of mucosa, lamina propria and muscularis mucosa. The majority of sections are of diagnostic quality. There is no evidence of mucosal erosion or ulceration, infection or neoplasia. Focally there is mild dysplasia of crypts and diffusely the lamina propria is infiltrated by minimally increased lymphocytes and plasma cells.
2	Placebo	RVB12	1	Stomach: G702 (x1): Eleven pieces of stomach are examined which are variably composed of fundic and pyloric mucosa and submucosa. The biopsies are of excellent diagnostic quality. There is no ulceration or erosion of the gastric epithelium and the prevalence of lymphoid follicles within the lamina propria are within normal limits.	Duodenum: G703 (x1): Ten pieces of duodenum are examined which are composed of mucosa and submucosa. The biopsies are of excellent diagnostic quality. There is no ulceration or erosion of the epithelium and villi are an appropriate length.	Ileum 6704: Nine pieces of ileum are examined which are variably composed of mucosa, submucosa and muscularis. The biopsies are of excellent diagnostic quality. There is no ulceration or erosion of the epithelium, however it is multifocally overlain by a large amount of mucus and cellular debris. The villi are mildly stunted and there is a very mild increase in eosinophils, lymphocytes and plasma cells within the lamina propria. Occasionally crypts	Colon: G705 (x1): Ten pieces of colon are examined which are composed of mucosa and submucosa. The biopsies are of excellent diagnostic quality. There is no ulceration or erosion of the epithelium, however diffusely the epithelium is overlain by a large amount of mucus. The collagen fibres of the superficial lamina propria are expanded by clear space (oedema) and diffusely the lamina propria is infiltrated by

						are surrounded by mildly increased fibrous tissue	low numbers of lymphocytes and plasma cells. The colonic crypts are mildly hyperplastic
2	Placeb o	RVB20	1	G2582 stomach (6 sections and smaller fragments) these sections of gastric mucosa extend from the surface epithelium to the deep lamina propria and often include small fragments of muscularis mucosa. There is multifocal mild crush artefact. This affects less than 10% of the sections. The sections are of good diagnostic quality. The mucosa is lined by well differentiated tall columnar surface epithelial layer. There is multifocal mild epithelial attenuation. Small amounts of amphiphilic mucoid material are present in the surface mucosa. Occasional clusters of spiral eosinophilic to basophilic bacteria are present on the mucosal surface. These measure up to 30 µm in length. Cellular infiltrate is not noted around these organisms. Multifocally, the surface epithelial cells are absent (ulceration). The underlying lamina propria contains mildly increased numbers of lymphocytes and	G2583: duodenum (14 sections) the sections extend from the surface epithelium to the deep lamina propria, often including muscularis mucosa. The sections are of good diagnostic quality. There is little crush artefact present, which is mild, affecting up to 10% of the sections. Villi are present and are lined by tall columnar well differentiated epithelial cells. There is moderate villous stunting blunting and fusion. Villi are approximately a 3rd to half of there are expected length. There are often double there are expected with. Lamina propria lymphocytes and plasma cells are diffusely mildly increased. The occupying up to 50% of the lamina propria in some high powered fields. There are accompanied by clusters of eosinophils and neutrophils, numbering up to 10 cells of each type per 40 X field. = Crypts are often mildly dilated and contain small amounts of eosinophilic material and	G2584: ileum (14 sections and smaller fragments) these sections of ileal mucosa extend from the surface epithelium to the deep lamina propria, occasionally including muscularis mucosa. The mucosa is lined by tall columnar well differentiated epithelial cells. There is minimal crush artefact present sections are of good diagnostic quality. There are low numbers of intraepithelial lymphocytes present. The lamina propria contains diffusely mildly increased numbers of lymphocytes and plasma cells. Scattered clusters of eosinophils and neutrophils are present within the lamina propria. Multiple submucosal lymphoid follicles are present and appear well differentiated. They have prominent germinal centres and high numbers of dendritic cells mixed with lymphocytes.	G2585: colon (214 sections and smaller fragments) the sections extend from the surface epithelium to the deep lamina propria. They include muscularis mucosa in some areas. There is multifocal mild crush artefact. The sections are of good diagnostic quality. Sections are lined by tall columnar well differentiated epithelial cells. There is multifocal marked epithelial attenuation. Multifocal clusters of neutrophils are noted in the superficial lamina propria in these areas. Expected numbers of lymphocytes and plasma cells are present in the lamina propria. Occasional clusters of eosinophils are present in the lamina propria numbering up to 5 cells per 40 X field. Crypts are well oriented to the mucosal surface. There is minimal crypt contortion. There is multifocal mild crypt dilation.

				plasma cells, which occupying up to 50% of the lamina propria. This mainly affects pyloric sections. In sections from the body of the stomach, there is diffuse mild atrophy of parietal and chief cells. Small amounts of fibrous connective tissue are present throughout the lamina propria, extending in bands of approximately 2 to 3 cells between individual glandular nests. Occasional clusters of eosinophils and neutrophils are present in the superficial lamina propria. These number up to 5 cells of each type per 40 X field. Fibrous tissue is more prominent in pyloric areas, with bands of fibrous tissue extending between gastric glands in bands of up to 10 cells thick. More prominent neutrophilic and lesser eosinophilic aggregates are present at this site. Dense aggregates of lymphoid follicles are present mainly affecting pyloric samples and occupying up to 50% of the samples in these areas.	necrotic material. Intraepithelial lymphocytes are mildly increased, with up to 20 cells noted per 40 X field. Intraepithelial large granular lymphocytes are multifocally mildly increased.		
2	Placeb o	RVB20	3	G3486 (x1) Stomach (12 sections): The sections consist of pyloric and fundic gastric mucosa and extend from the	G3487 (x1) Duodenum (11 sections): The sections extend from the surface epithelium to the mid to deep		G3488 (x1) Colon (12 sections): The sections extend from the surface epithelium to the deep

				surface epithelium to the lamina propria and in one section includes muscularis mucosa. There is minimal crush artefact. The sections are variably orientated and are of good diagnostic quality. The mucosa is lined by tall columnar epithelial cells with no evidence of attenuation or degeneration. There is moderate surface mucus and spiral bacteria are not evident. Low numbers of intraepithelial lymphocytes are present. There is mild, diffuse expansion of lamina propria by mildly increased numbers of lymphocytes and plasma cells. Lymphoid follicles are present within the deep mucosa of multiple sections, accounting for <10% of the biopsy sections. There is a small to moderate amount of fibrous connective tissue (fibrosis) separating the gastric glands of the fundus in the superficial mucosa.	lamina propria and do not include muscularis mucosa. Sections are variably orientated and there is minimal crushing artefact. The samples are of good diagnostic quality. Where longitudinally sectioned villi appear mildly stunted. The villous epithelium is present in a single layer of tall columnar cells and erosion or ulceration is not observed. There is an increased proportion of goblet cells within the surface epithelium. Intraepithelial cells are present in acceptable numbers. There is a mild increase in lymphocytes and plasma cells within the lamina propria.		lamina propria, often including muscularis mucosa. A single layer of columnar epithelium covers the surface and crypts and attenuation, erosion or ulceration is not noted. There is mild goblet cell hyperplasia with mild thickening and distortion of crypts which occasionally contain cellular debris (crypt
2	Placebo	RVB28	1	H896 (x1) Stomach (12 sections): The sections consist of pyloric and fundic gastric mucosa and extend from the surface epithelium to the lamina propria with one section including muscularis mucosa. There is multifocal mild crush	H897 (x1) Duodenum (12 sections): The sections extend from the surface epithelium to the mid to deep lamina propria. Sections are variably orientated and there is mild to moderate crushing artefact. The samples are of adequate	H898 (x1) 'Ileum'/colon (3 sections): The sections extend from the surface epithelium and include lamina propria and muscularis mucosa. Sections are variably orientated and villi are not apparent with large numbers	H899 (x1) Colon (9 sections): The sections extend from the surface epithelium to the deep lamina propria, frequently including the muscularis mucosa. The surface mucosa is multifocally lost (eroded)

				artefact. The sections are variably orientated and are of good diagnostic quality. The mucosa is lined by tall columnar epithelial cells with no evidence of attenuation or degeneration. Spiral bacteria are observed at the mucosal surface. Low numbers of intraepithelial lymphocytes are present and the number of lymphocytes and plasma cells within the lamina propria are considered within normal limits. There is multifocal expansion of superficial blood vessels by erythrocytes (congestion).	diagnostic quality. Villi appear mildly shortened and the villous epithelium is present in a single layer of tall columnar cells. No erosions or ulcerations are observed. Intraepithelial lymphocytes are present within normal limits. There is a mild increase in lymphocytes and plasma cells within the lamina propria and occasional neutrophils are present. Central lymphatics are within normal limits.	of goblet cells present, consistent with colonic mucosa. The surface mucosa is multifocally lost (eroded) with adjacent epithelial cells being attenuated. Underlying superficial lamina propria is minimally expanded by brightly eosinophilic material (presumptive fibrin) and clusters of neutrophils. There is a diffuse, mild increase in lamina propria lymphocytes and plasma cells and intraepithelial lymphocytes appear within normal limits.	with adjacent epithelial cells being attenuated. Underlying superficial lamina propria is minimally expanded by brightly eosinophilic material (presumptive fibrin) and clusters of neutrophils. The lamina propria is loosely arranged (oedematous) and there is a diffuse, mild increase in lamina propria lymphocytes and plasma cells. Crypts are occasionally hyper cellular and distorted (crypt hyperplasia).
2	Placebo	RVB30	1	H1050, stomach x1. Nine sections comprising fundic and pyloric mucosa are examined. The sections extend from the surface epithelium to the muscularis mucosa, they are well oriented, there is minimal crush artefact and they are of good diagnostic quality. They are histologically unremarkable. No microorganisms are seen and there is no evidence of neoplasia in the examined sections.	H1051, duodenum x1. Fourteen sections are examined, the sections predominantly comprise transverse profiles of villi, there is mild crush artefact, and they are of moderate diagnostic quality. Villous architecture is difficult to assess but where present they are within normal limits. The villous propria is infiltrated with low numbers of plasma cells and fewer lymphocytes, there is a single crypt abscess in one section. No microorganisms are seen and there is no evidence of		H1052, colon x1. Twelve sections are examined, they extend from the surface epithelium to the muscularis mucosa, they are well oriented, there is minimal crush artefact and they are of good diagnostic quality. There is minimal superficial lamina propria infiltration with lymphocytes and plasma cells and a mild increase in tortuosity of crypts. No microorganisms are seen and there is no evidence of

					neoplasia in the examined sections.		neoplasia in the examined sections.
2	Placebo	RVB31	1	Stomach; H1124 (x1): Ten sections are examined that are composed of fundic and pyloric mucosa and lamina propria and most sections are of good diagnostic quality. Diffusely fundic glands are separated by a mild increase in proprial fibrous tissue and the lamina propria is infiltrated by mildly increased numbers of lymphocytes, plasma cells, fewer eosinophils and occasional neutrophils. Diffusely pyloric glands are separated by a marked increase in proprial fibrous tissue and the lamina propria is infiltrated by mildly increased numbers of lymphocytes, plasma cells and occasional neutrophils. There is abundant mucus on the mucosal surface. There is no evidence of mucosal erosion or ulceration in either location.	Duodenum; H1125 (x1): Fifteen sections are examined that are composed of mucosa and superficial lamina propria, frequently sections are superficial/tangential. Four sections are of reasonable diagnostic quality. The lamina propria is infiltrated by mildly increased numbers of plasma cells with fewer lymphocytes and occasional eosinophils and neutrophils. There is mild villous atrophy, occasional mild dilation of villous lacteals and mucosal glands are mildly hyperplastic. There is no evidence of mucosal erosion or ulceration.		Colon; H1126 (x1): Ten sections are examined that are composed of mucosa, lamina propria and muscularis mucosa. The majority of sections are of good diagnostic quality. The lamina propria is moderately expanded by increased amounts of fibrous tissue that is infiltrated by occasional neutrophils. Diffusely there is mild goblet cell hyperplasia and abundant surface mucus. There is no evidence of mucosal erosion or ulceration.
2	Placebo	RVB31	3				
2	Placebo	RVB34	1	H2698, stomach x1. 12 sections of pyloric and fundic mucosa are examined, the majority comprise well orientated sections extending from the surface epithelium to	H2699, duodenum x 1. 13 sections of duodenal mucosa are examined, four of these extend from the surface epithelium to the muscularis mucosa, there is minimal crush		H2700, colon x 1. 11 sections of colonic mucosa are examined, they extend from the surface epithelium to the muscularis mucosa, there is mild crush artefact,

				the muscularis mucosa, there is moderate crush artefact, they are adequate for examination. There is a mild increase in lamina propria connective tissue and corresponding glandular atrophy. There is no evidence of erosion, ulceration, significant inflammation or neoplasia in the examined sections.	artefact, they are adequate for examination. The remainder comprise cross sections of villi. Villous profiles are diffusely mildly broadened and shortened. The villous propria is multifocally mildly infiltrated with aggregates of plasma cells, fewer lymphocytes and rare neutrophils.		they are adequate for examination. The lamina propria is mildly expanded by oedema and mildly infiltrated with low numbers of lymphocytes and plasma cells.
2	Placebo	RVB34	3	Stomach, H3559 x 1: One tissue sample is examined and consists of pyloric mucosa that extents to the deep lamina propia. There is mild proprial fibrosis and a mild increase of neutrophils and eosinophils. There is no evidence of Helicobacter spp. or neoplasia within the examined tissues.	Duodenum, H3559 x 1: Ten tissue segments and fragments are examined. They are for good diagnostic quality. There is moderate crush artefact that affect 15% of the examined tissue. They extend from the mucosa to the deep lamina Propria and consist of longitudinal and very superficial transverse sections of the villous tips. In longitudinal sections villous tips are of expected length and thickness. Numbers of proprial lymphocytes and plasm a cells are within normal limits. There is no evidence of neoplasia within the examined tissues.	Ileum, H3560 x 1: Twelve poorly oriented and partially folded tissue sections and fragments are examined. There is moderate crush artefact that affects 15% of the examined tissue. They extend from the mucosa to the deep lamina propria and occasionally contain muscularis mucosae. Villous profiles are of expected length and thickness. Proprial neutrophils are minimally increased. There is no evidence of neoplasia within the examined tissues.	Colon, H3561 x 1: Twelve tissue sections are examined that extend from the mucosa to the deep lamina propria and occasionally contain muscularis mucosae. They are of good diagnostic quality and contain minimal crush artefact. The tunica mucosa is line by a columnar well-differentiated epithelium. Numbers if proprial lymphocytes and plasma cells are within normal limits. There is no evidence of neoplasia within the examined tissues.

TABLE A47. Clinical pathology results - Group 2. Diagnosis and comments.

Group	Treatment	Patient No.	Visit No.	Tissue - Microscopic Diagnosis	Tissue - Pathologist Comment	Tissue - Investigator Comment
2	Placebo	RVB02	1	1. Duodenum; diffuse, mild, lymphoplasmacytic infiltrate<br>2. Ileum; mild eosinophilic ileitis with mild villus atrophy<br>3. Colon; diffuse, mild eosinophilic colitis with proprial oedema	Histopathological examination of the received tissues reveals the presence of mild inflammatory changes within the duodenum, ileum and colon. These changes are mild and non-specific, but could be compatible with the clinical diagnosis of inflammatory bowel disease. Many of these cases are idiopathic however potential contributory factors to consider include aberrant immune responses to luminal microflora or dietary antigens or underlying systemic disease. Mucosal ulceration, glandular atrophy and/or fibrosis or neoplastic disease is not observed in these sections.	Mild IBD
2	Placebo	RVB02	3	Duodenum; mild lymphoplasmacytic infiltrate<br>Ileum; sample non-diagnostic<br>Colon; mild neutrophilic and lymphoplasmacytic colitis with mucosal fibrosis and crypt hyperplasia	Histopathological examination of the received tissues reveals a mild neutrophilic and lymphoplasmacytic colitis. The changes in the duodenum are mild. The ileal sample could not be assessed in detail due to the extent of squash artefact within the submitted samples.	Still showing signs of inflammation with fibrosis, therefore tis may be potentially chronically present

2	Placebo	RVB05	1	F3398 (x1): Gastritis, lymphocytic to plasmacytic, chronic, multifocal, moderate, stomach with marked lymphoid follicular hyperplasia<br>F3399 (× 1): Enteritis, lymphocytic to plasmacytic, chronic, multifocal, moderate, duodenum<br>F3400 (× 1): Enteritis, lymphocytic to plasmacytic, chronic, multifocal, mild with lymphangiectasia, multifocal, marked, ileum F3401 (×1): Colitis, lymphocytic to plasmacytic, chronic, multifocal, mild, colon	These sections of gastrointestinal mucosa are chronically inflamed with the predominance of inflammation present within the small intestine. The character of the inflammatory infiltrate and concurrence of crypt abscess formation is consistent<br>with a diagnosis of inflammatory bowel disease (IBD). Indeed, the formation of crypt abscesses and marked dilation of lymphatics within the small intestinal mucosa is often, as in this case, associated with a protein loosing enteropathy. There is no evidence of neoplasia within the examined sections.	Consistent with PLE/chronic enteropathy
2	Placebo	RVB09	1	1. Duodenum; diffuse, moderate lymphoplasmacytic and neutrophilic duodenitis with crypt abscessation and marked villous atrophy<br>2. Ileum; moderate lymphoplasmacytic and histiocytic enteritis with marked villous atrophy<br>3. Colon; diffuse, moderate lymphoplasmacytic colitis with proprial oedema	Histopathological examination of the received tissues reveals the presence of moderate inflammatory changes within the duodenum, ileum and colon alongside marked villous atrophy of the duodenum and ileum. These changes are non-specific<br>and generally consistent with the chronicity of the clinical signs described in this dog however no overt fibrosis is apparent. Many of these cases are idiopathic however potential contributory factors to consider include aberrant immune responses to luminal microflora or dietary antigens or underlying systemic disease. Mucosal ulceration or neoplastic disease is not observed in these sections.	Consistent with IBD

2	Placebo	RVB11	1	1. Duodenum; diffuse, chronic-active, marked, mixed (lymphoplasmacytic, eosinophilic and neutrophilic) duodenitis with multifocal necrosis<br>2. Ileum; diffuse, moderate, lymphoplasmacytic, ileitis	Examination of the received tissues reveals marked duodenitis and moderate ileitis. The changes which include a mixed inflammatory infiltrate (lymphoplasmacytic, neutrophilic and eosinophilic) are suggestive of proximal small intestinal inflammatory bowel disease which may be worsened by dysbiosis in this case. A Giemsa stain to characterise the morphology of the microorganisms present is pending and will be reported as an addendum when complete. There are minimal changes, unlikely to be clinically significant, within the sections of stomach and colon examined. There is no evidence of neoplasia within the examined sections	Results consistent with IBD
2	Placebo	RVB11	3	1. Stomach; no significant histopathological abnormality detected<br>2. Duodenum; minimal chronic lymphoplasmacytic proprial inflammation<br>3. Ileum; minimal chronic lymphoplasmacytic proprial inflammation<br>4. Colon; minimal chronic lymphoplasmacytic proprial inflammation	Histopathological examination of the received tissues reveals only the presence of minimal proprial inflammation within the duodenum, ileum and colon, consistent with the improvement of clinical signs. There is no evidence of neoplasia in the examined sections	Marked improvement in results
2	Placebo	RVB12	1	Stomach: within normal limits<br>Duodenum: within normal limits<br>Ileum: mild catarrhal eosinophilic lymphoplasmacytic enteritis<br>Colon: mild catarrhal lymphoplasmacytic colitis	Histopathological examination of the received tissues reveals the presence of a mild eosinophilic lymphoplasmacytic enteritis and lymphoplasmacytic colitis. The cause of the pathology was not evident, however there was no evidence of neoplasia or pathogens on the examined sections.	Stomach and duodenum diagnosis: NCS.<br>Ileum and Colon Diagnosis: Consistent with IBD

2	Placebo	RVB20	1	G2582: gastritis, erosive and ulcerative, neutrophilic, eosinophilic, lymphoplasmacytic, multifocal, moderate, chronic with lymphoid hyperplasia, and fibrosis, multifocal, moderate; stomach<br>G2583: enteritis, lymphoplasmacytic, neutrophilic, eosinophilic, multifocal to coalescing, moderate, chronic with villous atrophy, multifocal, moderate; duodenum<br>G2584: ileitis, lymphoplasmacytic, neutrophilic, eosinophilic, multifocal to coalescing, moderate, chronic with villous atrophy and lymphoid hyperplasia, multifocal, moderate to marked; ileum<br>G2585: colitis, erosive, neutrophilic, eosinophilic, multifocal, moderate, chronic;<br>colon	The most severe areas of inflammation in these sections are present within the small<br>intestine and gastric pylorus region. There is evidence of a chronic mixed<br>gastroenteritis. Mildly to moderately increased numbers of eosinophils are present, which may reflect a hypersensitivity response. Mildly to moderately increased numbers of neutrophils are also noted, which would likely indicating a reaction to luminal bacteria. There is similar inflammation present in the other areas of the stomach and the colon, with areas of ulceration present in<br>both sites. I did not detect the presence of neoplasia in any of these sections. There are some Helicobacter species noted in the stomach mucosa, however, no detectable cellular response is evident. Prominent numbers of large granular lymphocytes are present within the surface mucosa of the duodenum, which may indicate an innate immune response to surface antigens.	Consistent with IBD

2	Placebo	RVB20	3	Stomach; diffuse, mild, lymphoplasmacytic gastritis with mild fibrosis and glandular atrophy<br>Duodenum; diffuse, mild, lymphoplasmacytic duodenitis with mild villous stunting<br>Colon; diffuse, mild, lymphoplasmacytic and eosinophilic colitis with mild fibrosis and crypt abscessation	The previous endoscopic biopsies and report have been reviewed alongside the current submission. There is evidence of ongoing mild, lymphoplasmacytic gastroenteritis and colitis throughout the examined sections, consistent with an ongoing inflammatory bowel disease. Neutrophils or eosinophils within the lamina propria are not identified within the stomach or duodenum with only few eosinophils observed within the colon. There is no evidence of microbial organisms or neoplasia. Progression of the inflammatory disease process is not apparent and there is overall decrease in the amount and type of the inflammatory cell component.	Improved from last biopsies, suggestive of improved IBD
2	Placebo	RVB28	1	Stomach; no pathologic abnormalities identified<br>Duodenum; mild lymphoplasmacytic and neutrophilic duodenitis with mild villous blunting<br>Colon; moderate, multifocal, erosive, lymphoplasmacytic and neutrophilic colitis with mild multifocal crypt hyperplasia	Microscopic examination of the submitted endoscopic gastrointestinal biopsies identifies mild changes within the duodenum and moderate changes within the colon. The stomach is considered histologically normal with the potential for<br>congestion resulting in a macroscopically visible hyperaemia. The significance of spiral bacteria within the stomach of dogs currently remains unknown however there is no associated inflammation in this case. Villi are not appreciable on the submitted ileal sections and as such are more consistent with coming from a proximal colonic location. The changes observed within the duodenum and colon are considered non-specific with no infectious causes or evidence of neoplasia identified within the examined sections.	Findings consistent with IBD
2	Placebo	RVB30	1	Stomach; no significant changes<br>Duodenum; mild mucosal plasmacytic inflammation<br>Colon; minimal mucosal lymphoplasmacytic inflammation	Histopathological examination of the submitted tissue reveals mild changes in the intestine of this dog, which, if other causes are ruled out, could be compatible with a diagnosis of IBD. There is no evidence of neoplasia in the examined sections.	

2	Placebo	RVB31	1	Stomach; moderate proprial fibrosis with mild mixed proprial inflammation<br>Duodenum; mild villous atrophy with mild mixed proprial inflammation<br>Colon; moderate proprial fibrosis with occasional neutrophils	Examination of the received tissues reveals mild, chronic inflammatory changes within the stomach, duodenum and colon. There was no evidence of neoplasia in the examined sections and the inflammation could be consistent with inflammatory bowel disease if other differentials have been ruled out.	Biopsies consistent with IBD
2	Placebo	RVB31	3	Duodenum; histologically unremarkable<br>Colon; histologically unremarkable	The tissues are affected by what is presumed to be freeze thaw artefact, but within these limits there are no significant changes in the examined sections	
2	Placebo	RVB34	1	Stomach; minimal to mild glandular atrophy and fibrosis<br>Duodenum; mild duodenitis, plasmacytic, lymphocytic and neutrophilic<br>Colon; minimal to mild lymphoplasmacytic infiltration.	Histological examination reveals a range of changes in the tissues of this dog. The changes in the stomach are mild and non-specific and unlikely to have been functionally significant. The changes in the duodenum may have been functionally significant, they may be compatible with IBD if other causes have been ruled out.<br>The changes in the colon may also be consistent with IBD. There is no evidence of neoplasia and no infectious organisms have been detected in the examined sections.	
2	Placebo	RVB34	3	1. Stomach, pylorus; multifocal, minimal, proprial fibrosis with minimal, multifocal, neutrophilic, eosinophilic, infiltrates<br>2. Duodenum; without significant histopathological changes<br>3. Ileum; multifocal, minimal, neutrophilic infiltrates<br>4. Colon; without significant histopathological changes	Microscopic examination reveals that one of the samples submitted as duodenum is in fact pyloric mucosa with minimal fibrosis and very few proprial neutrophils and eosinophils. The samples from the ileum are poorly oriented but within these limits and similarly to the duodenum and colon, there are no significant histopathological changes in the examined tissues.	

APPENDIX 2

Table 1. Descriptive statistics of demographic variables (Group, Age, Gender, and Breed) with data summary after withdrawals within parentheses.

(A) Dog demographics (20 experimental units including 7 withdrawals)

Group	Age in months (at Visit 1)	Gender	Breed
**1**	25	FN	Lurcher
**1**	44	FN	Cross Breed
**1**	56	FE	Bearded Collie (withdraw)
**1**	81	FN	Staffordshire Bull Terrier
**1**	120	FN	Cocker Spaniel
**1**	14	ME	Cockapoo
**1**	29	ME	Border Collie
**1**	53	MN	Labrador Retriever
**1**	91	MN	Labrador Retriever
**1**	131	MN	Staffordshire Bull Terrier (withdraw)
**2**	19	FE	Whippet (withdraw)
**2**	54	FE	German Shepherd (withdraw)
**2**	22	ME	Boxer
**2**	23	ME	Border Collie

**2**	23	MN	French Bulldog (withdraw)
**2**	36	ME	Collie X
**2**	36	MN	Labradoodle
**2**	58	MN	Lhasa Apso
**2**	73	MN	Staffordshire Bull Terrier (withdraw)
**2**	80	ME	German Shepherd (withdraw)

(B) Contingency table by group vs. gender (frequency after withdrawals within parentheses)

**Group / Gender**	**1**	**2**	**Row Total**
**F (FE or FN)**	5 (4)	2 (0)	7 (4)
**M (ME or MN)**	5 (4)	8 (5)	13 (9)
**Column Total**	10 (8)	10 (5)	20 (13)

(C) Descriptive statistics of age (in months at Visit 1) by group and gender

**Group / Gender**	**1**	**2**	**Gender average ± sd**

**F**<br><br>**(FE or FN)**	65.20 ± 36.75<br><br>(67.50 ± 42.02)	36.50 ± 24.75<br>(0)	57.00 ± 34.62<br><br>(67.50 ± 42.02)
**M**<br><br>**(ME or MN)**	63.60 ± 47.60<br><br>(46.75 ± 33.59)	43.88 ± 23.38<br><br>(35.00 ± 14.53)	51.46 ± 34.26<br><br>(40.22 ± 23.81)
**Group average ± sd**	64.40 ± 40.10<br><br>(57.13 ± 36.92)	42.40 ± 22.43<br><br>(35.00 ± 14.53)	53.40 ± 33.58<br><br>(48.62 ± 31.48)

Figure 1. Group comparison of average (±sd) CCECAI and CIBDAI profiles

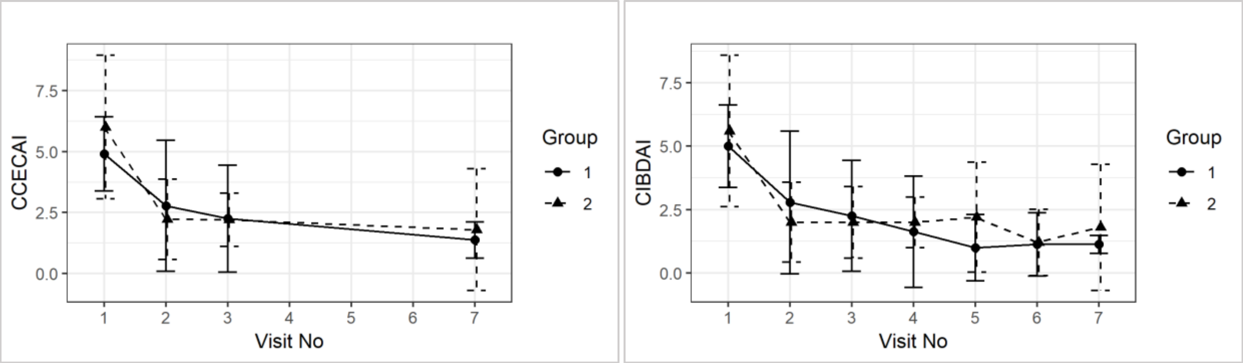

Figure 2. Group comparison of average (±sd) profiles for blood samples

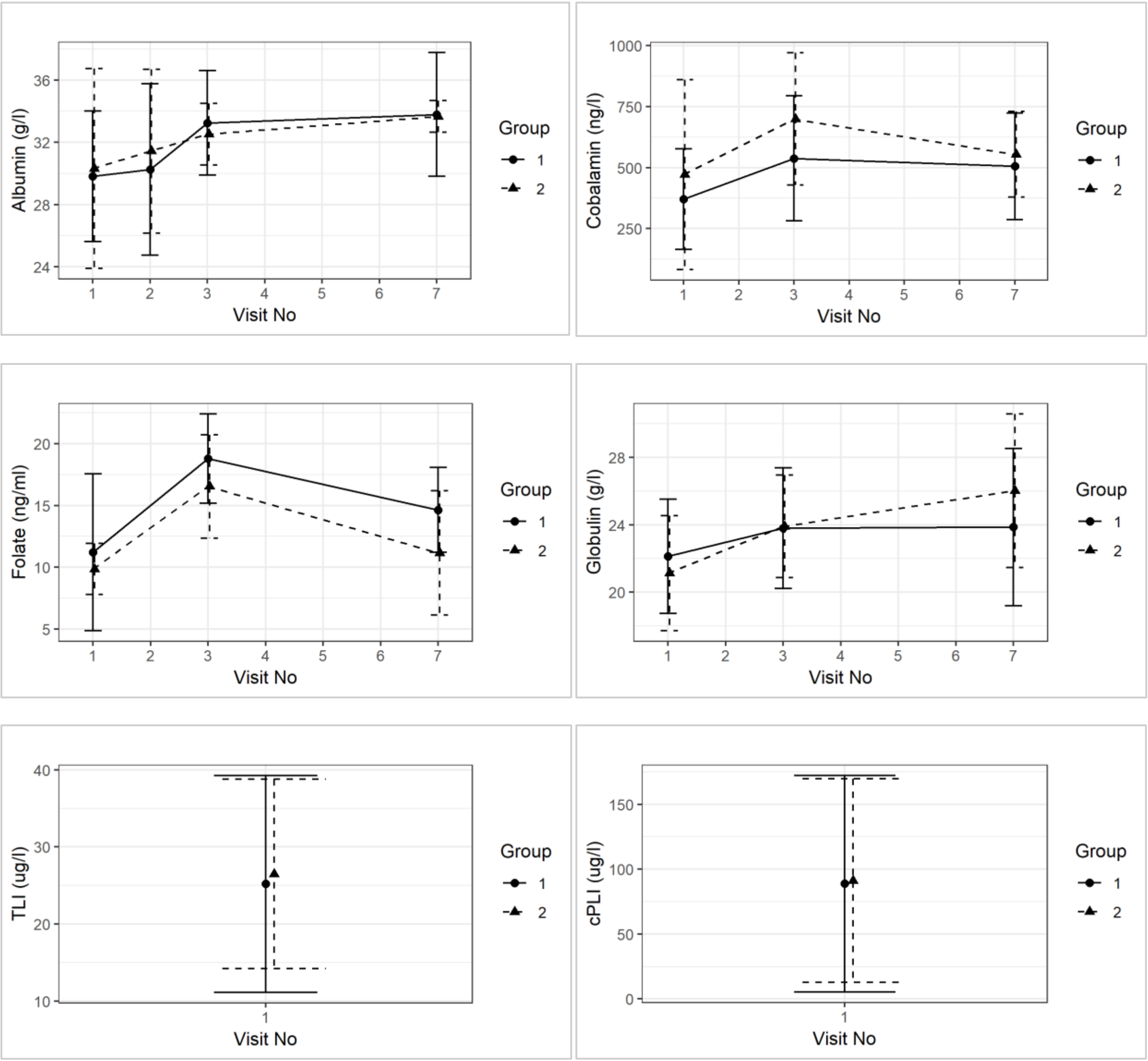

Figure 3. Group comparison of average (±sd) Endoscopy Score D, I, C profiles

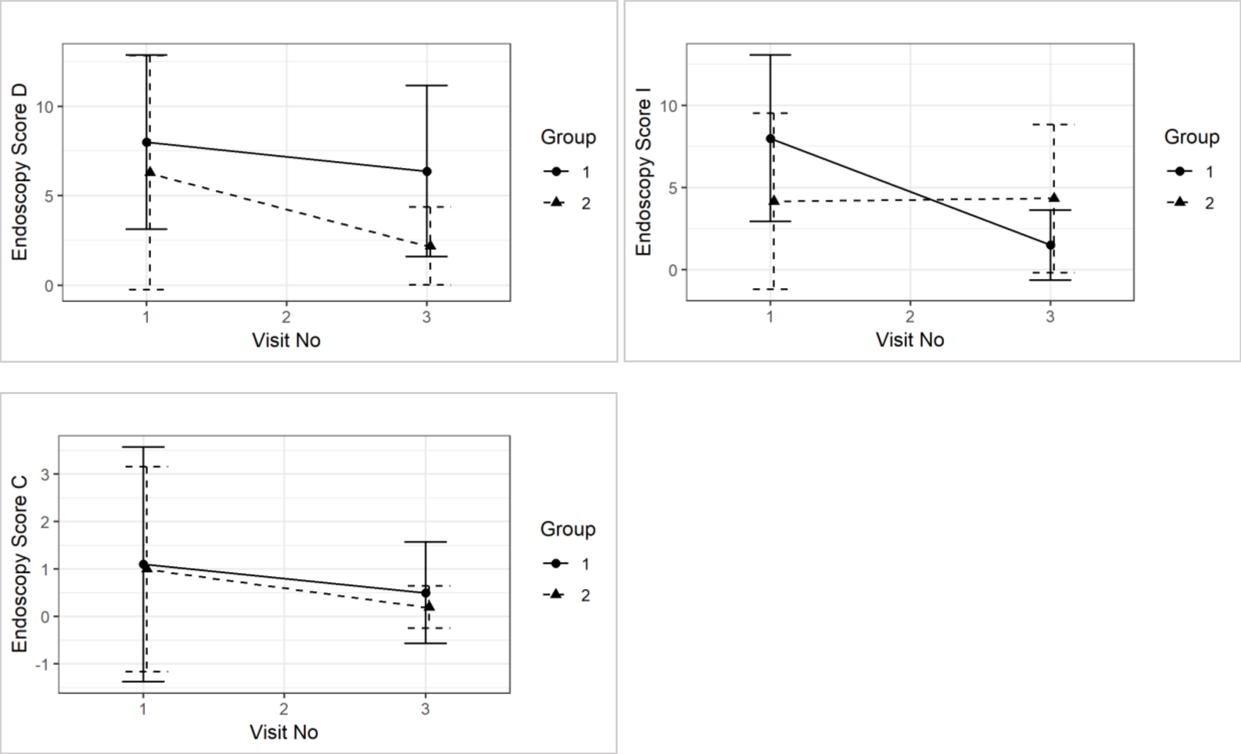

Figure 4. Group comparison of average (±sd) profiles for WSAVA scores

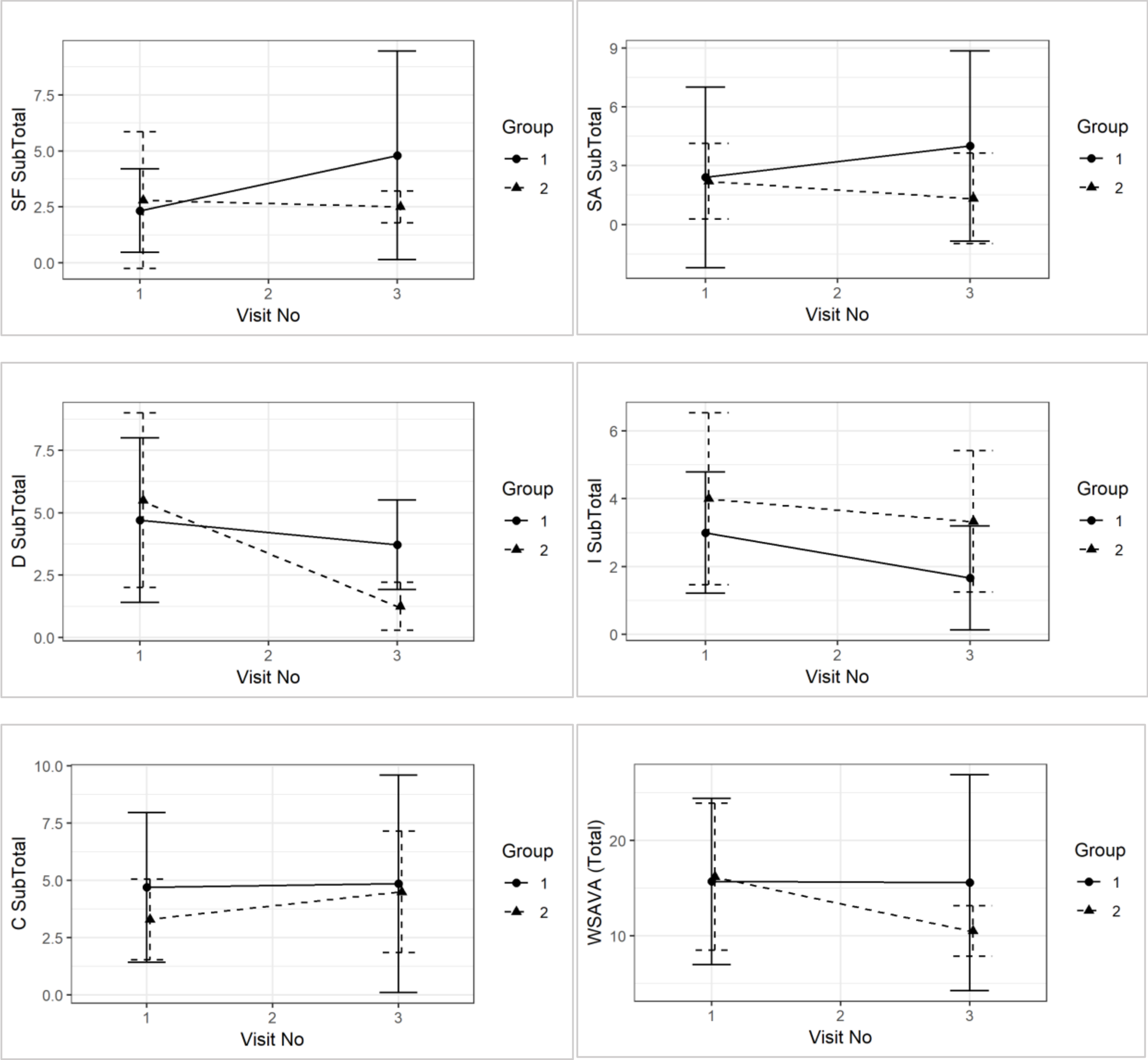

Figure 5. Pearson correlation coefficients (combining all the visits and groups 1 and 2) using *corrplot* package in R.

Positive correlations are displayed in blue color and negative correlations are in red. Additional colors (corresponding to their correlation values) are added in the plot when the test for linear association between paired variables results in p-value < 0.05 (p-values are not shown in the plot).

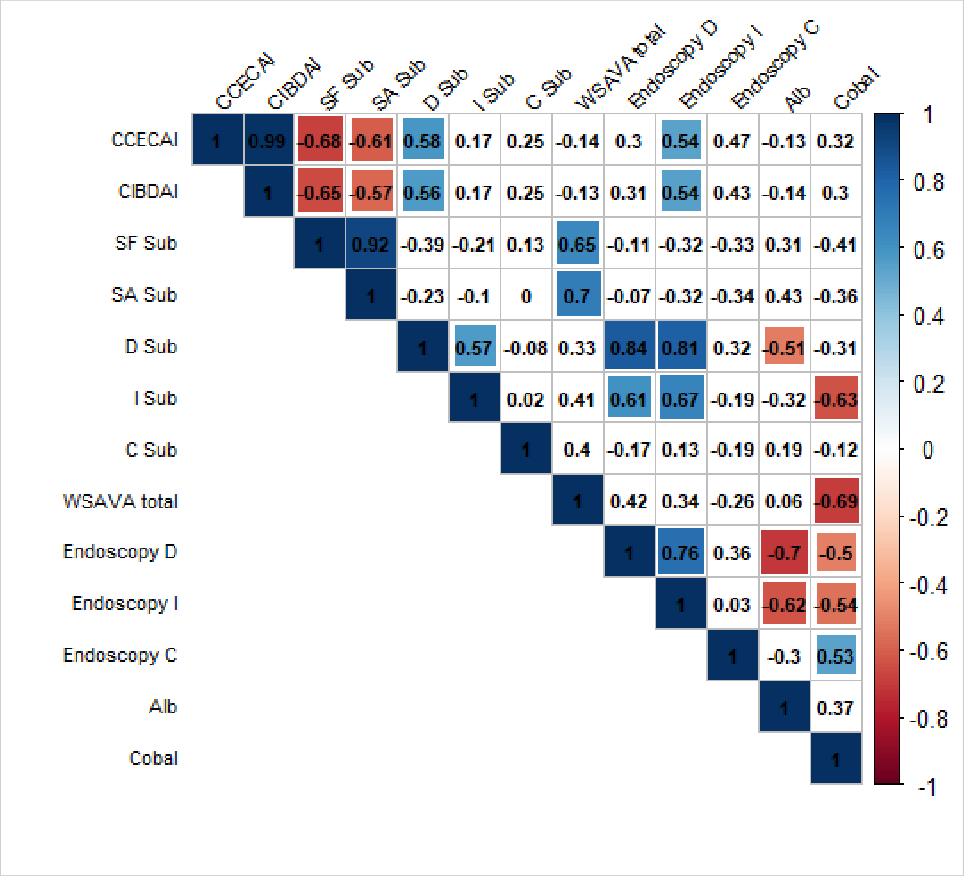

Supplement: S1 File — (PDF) [file pone.0250681.s001.pdf]
