# Supplementary material for: Clinical efficacy of prebiotics and glycosaminoglycans versus placebo In dogs with food responsive enteropathy receiving a hydrolyzed diet: A pilot study
Source: PLoS One. 2021 Oct 21;16(10):e0250681. doi: 10.1371/journal.pone.0250681 (PMC8530283; doi:10.1371/journal.pone.0250681)

Summary: demographics **updated on Sep. 2018**

Total number of experimental units = 20 (including 3 withdraws, RVB07 (Group=1), RVB09 (Group=2), RVB12 (Group=2))

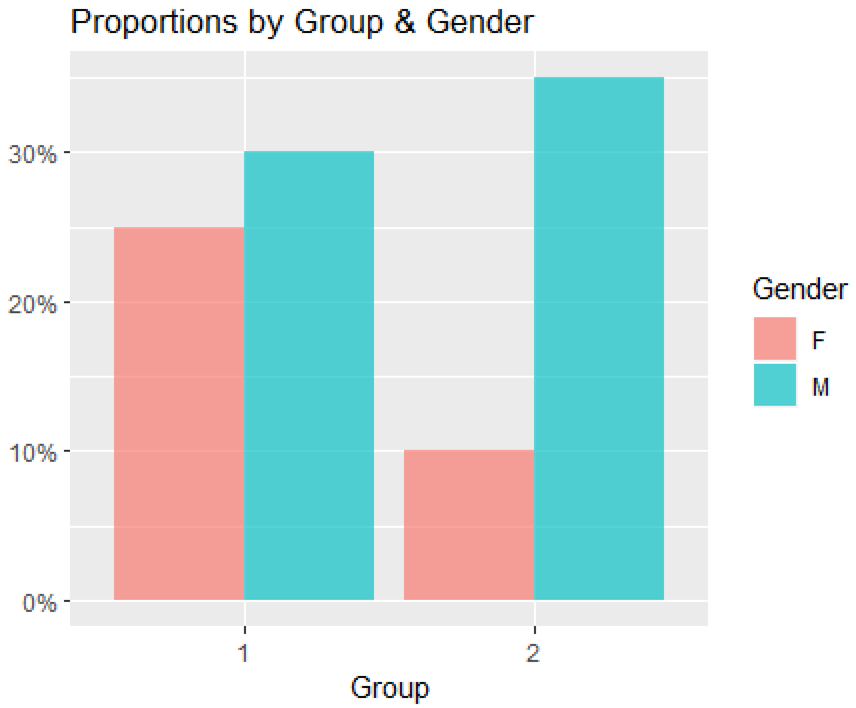

Total Observations in Table: 20

Gender	Group 1	Group 2	Row Total
F	5<br>0.250	2<br>0.100	7<br>0.350
M	6<br>0.300	7<br>0.350	13<br>0.650
Column Total	11<br>0.550	9<br>0.450	20

Age in Months (at initial visit, “Visit=1” ONLY)

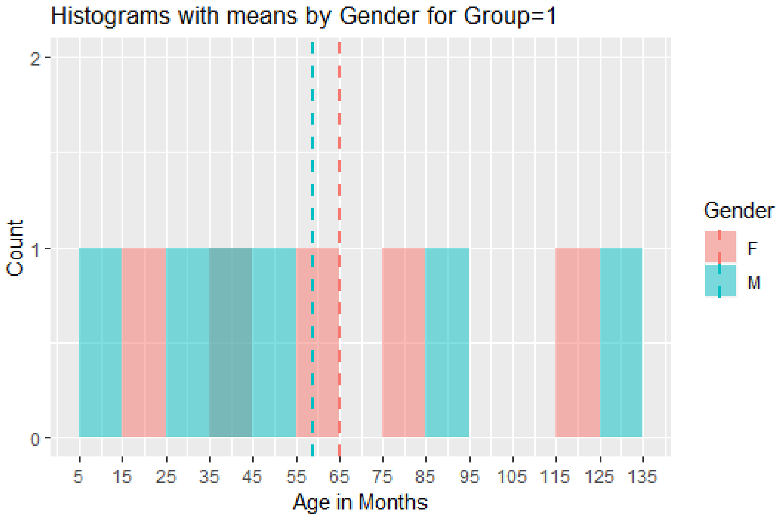

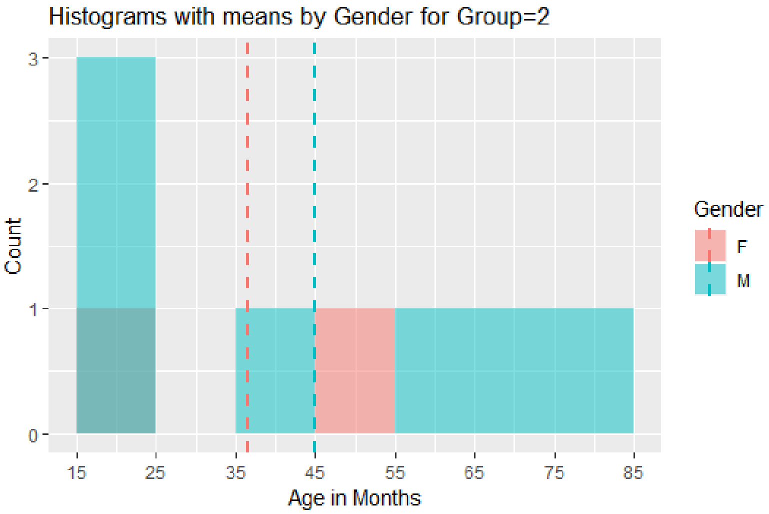

Group	Gender	Age.mean	Age.sd
1	F	65.20	36.75
1	M	59.00	44.04
2	F	36.50	24.75
2	M	45.00	25.02

Proportions by Gender, Group and Breed

	Gender	Group	Breed	n	proportion
1	F	1	Bearded Collie	1	0.05
2	F	1	Cocker Spaniel	1	0.05
3	F	1	Cross Breed	1	0.05
4	F	1	Lurcher	1	0.05
5	F	1	Staffordshire Bull Terrier	1	0.05
6	F	2	German Shepherd	1	0.05
7	F	2	Whippet	1	0.05
8	M	1	Border Collie	1	0.05
9	M	1	Cockapoo	1	0.05
10	M	1	Labradoodle	1	0.05
11	M	1	Labrador Retriever	2	0.10
12	M	1	Staffordshire Bull Terrier	1	0.05
13	M	2	Border Collie	1	0.05
14	M	2	Boxer	1	0.05
15	M	2	Collie X	1	0.05
16	M	2	French Bulldog	1	0.05
17	M	2	German Shepherd	1	0.05
18	M	2	Lhasa Apso	1	0.05
19	M	2	Staffordshire Bull Terrier	1	0.05

CCECAI & CIBDAI scores by group

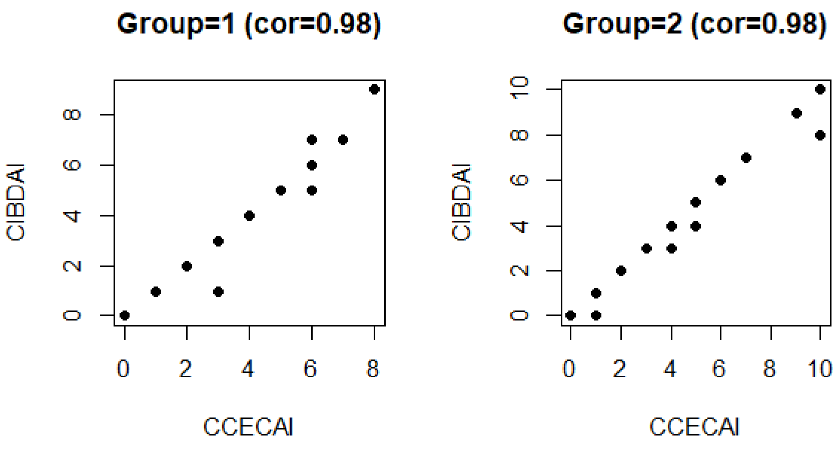

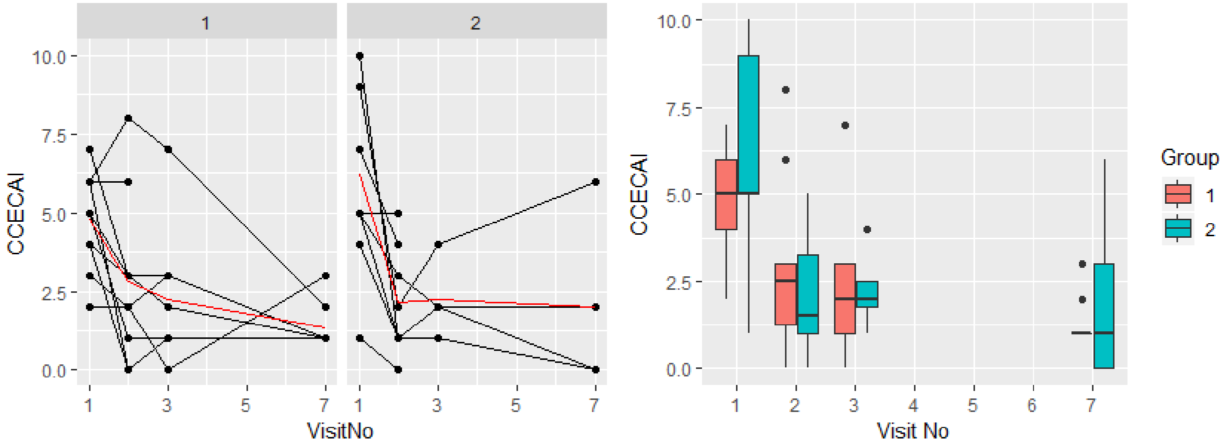

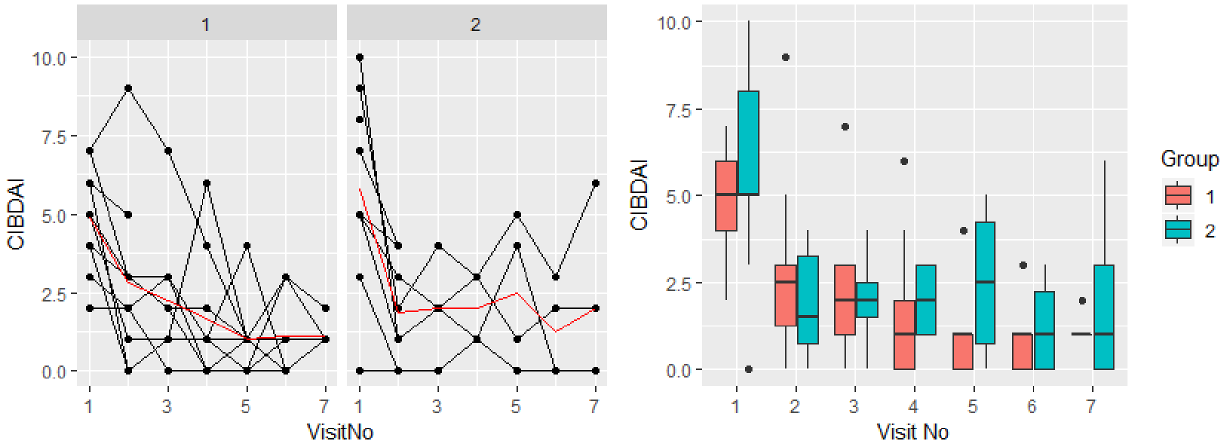

***Two variables, CCECAI and CIBDAI, are highly correlated.**

***P-value for CIBDAI = 0.4731 (the AUCs of the two groups are not significantly different at alpha=0.05)**

Endoscopy Scores by group (Visit No = 1,3)

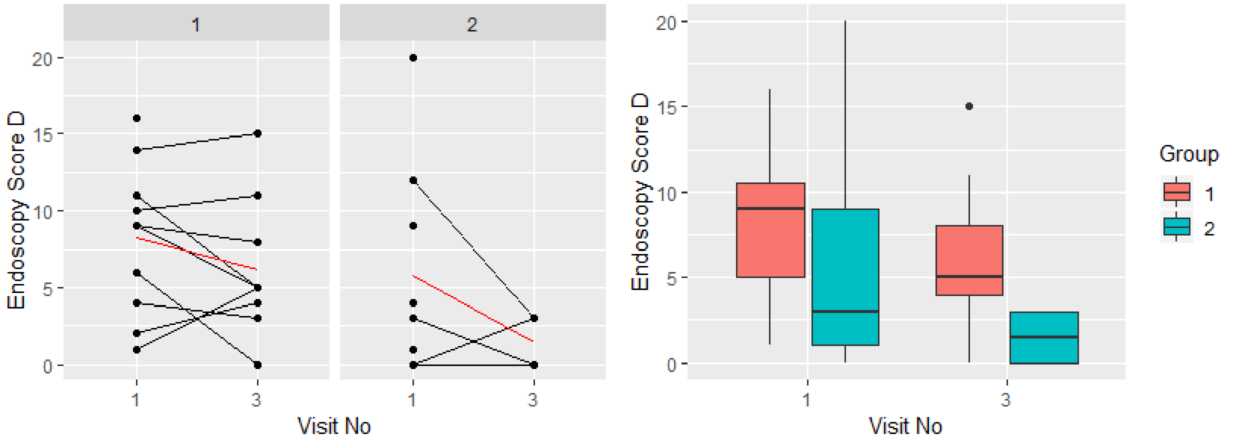

P-value=0.5641 (the interaction between Visit No and Group type is not significant at $\alpha = 0.05$)

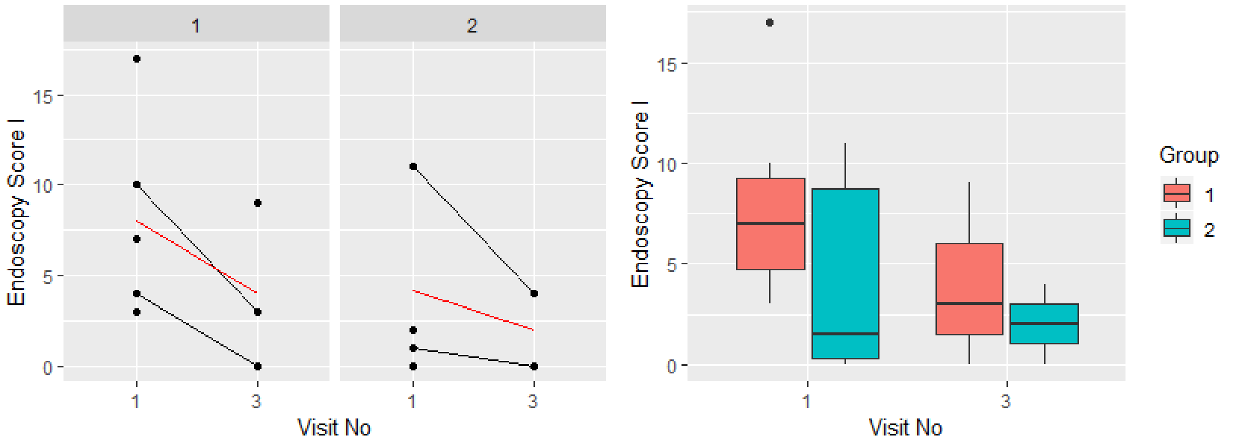

P-value=0.7381 (the interaction between Visit No and Group type is not significant at $\alpha = 0.05$)

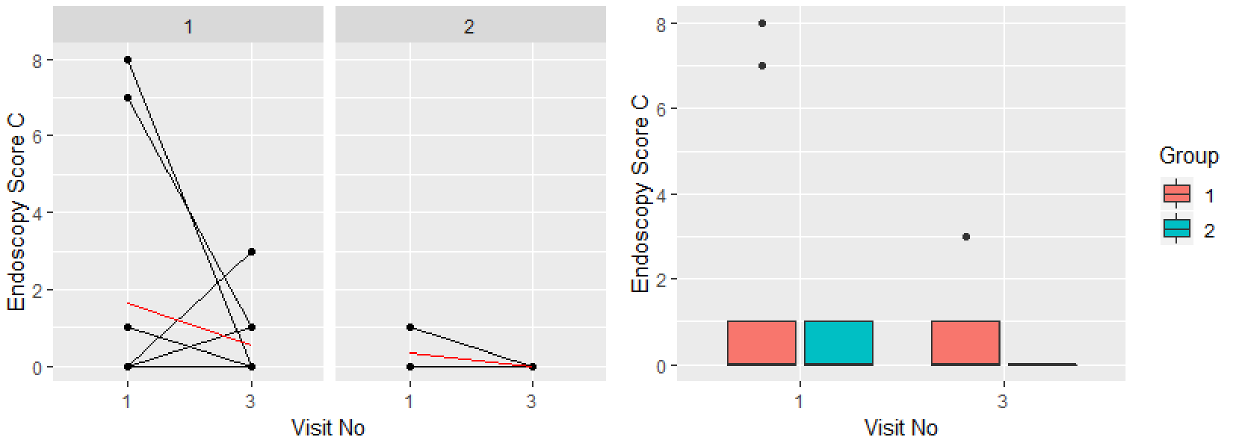

P-value=0.5897 (the interaction between Visit No and Group type is not significant at $\alpha = 0.05$)

WSAVA scores by group (Visit No = 1,3)
TOTAL = SFSubTotal + SASubTotal + DSubTotal + ISubTotal + CSubTotal

SF SubTotal

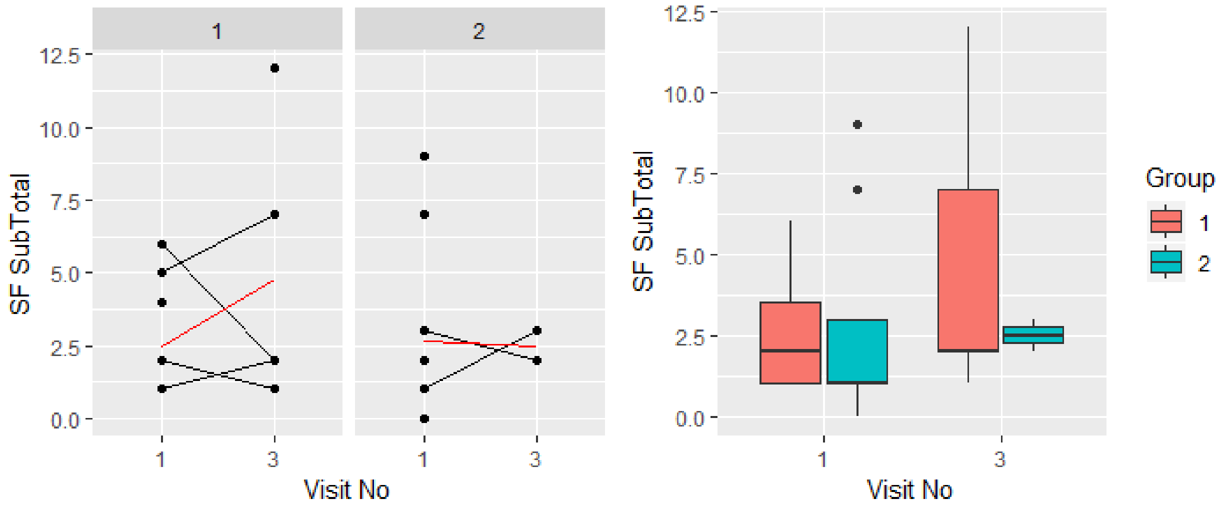

P-value = 0.4004
(the interaction between Visit No and Group type is not statistically significant at $\alpha = 0.05$)

SA SubTotal

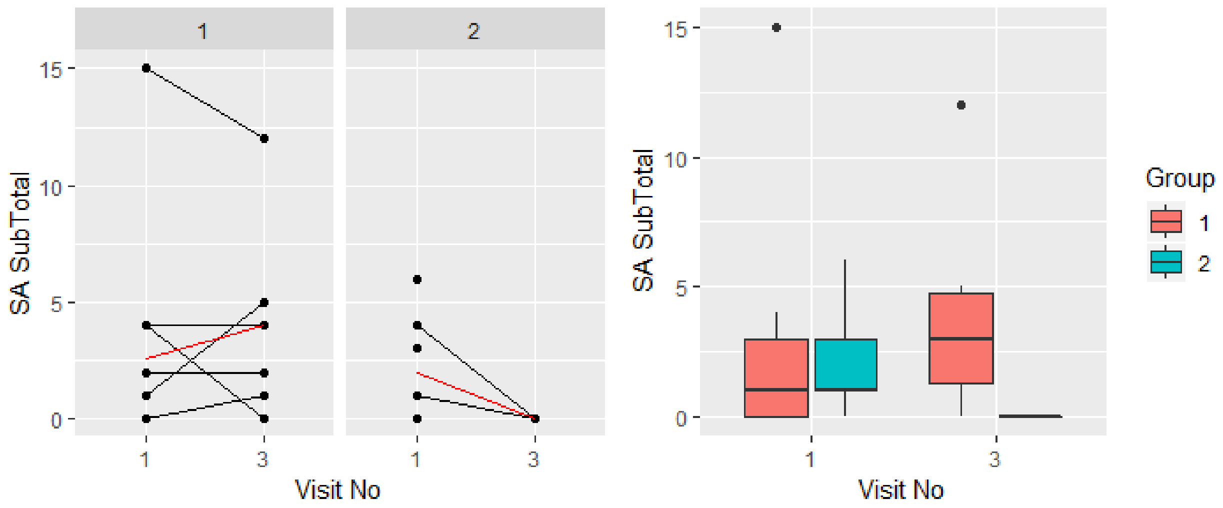

P-value = 0.3178
(the interaction between Visit No and Group type is not statistically significant at $\alpha = 0.05$)

D SubTotal

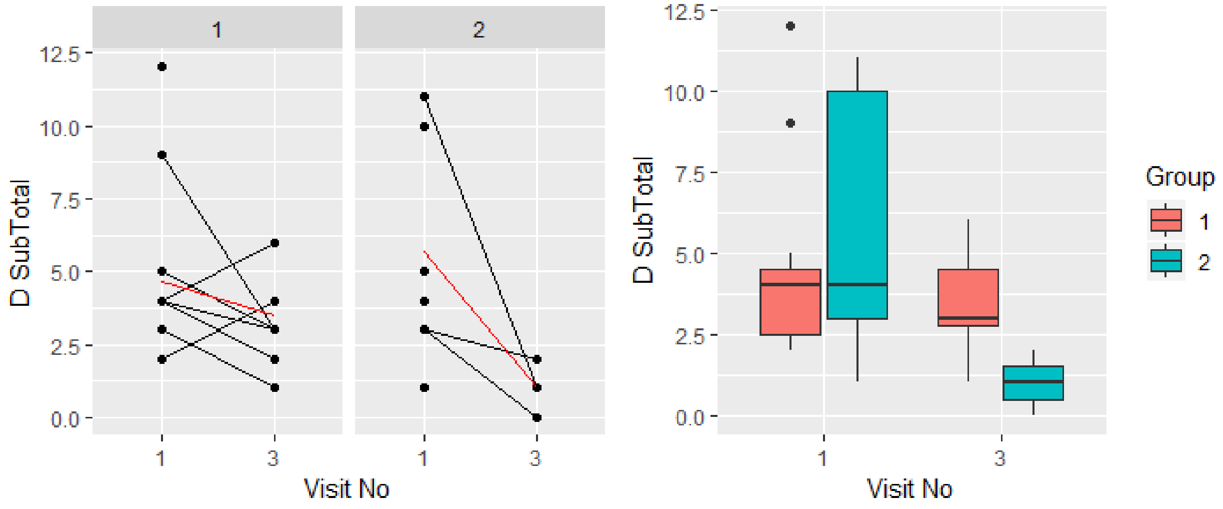

P-value = 0.1487
(the interaction between “Visit No” and “Group” type is not statistically significant at $\alpha = 0.05$)

I SubTotal

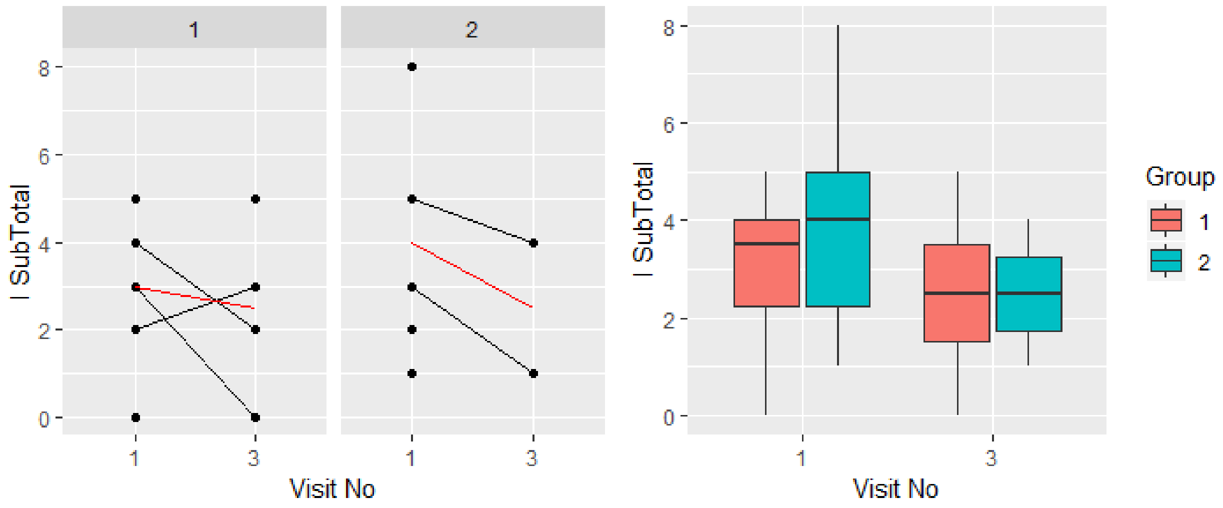

P-value = 0.6637
(the interaction between “Visit No” and “Group” type is not statistically significant at $\alpha = 0.05$)

C SubTotal

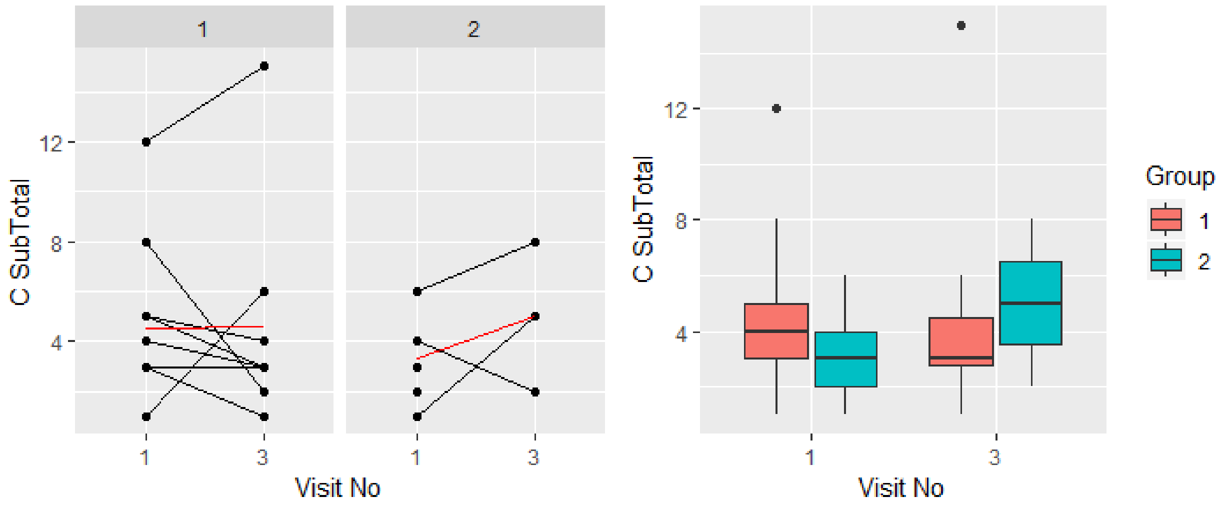

P-value = 0.5511
(the interaction between "Visit No" and "Group" type is not statistically significant at $\alpha = 0.05$)

TOTAL=SF SubTotal + SA SubTotal + D SubTotal + I SubTotal + C SubTotal

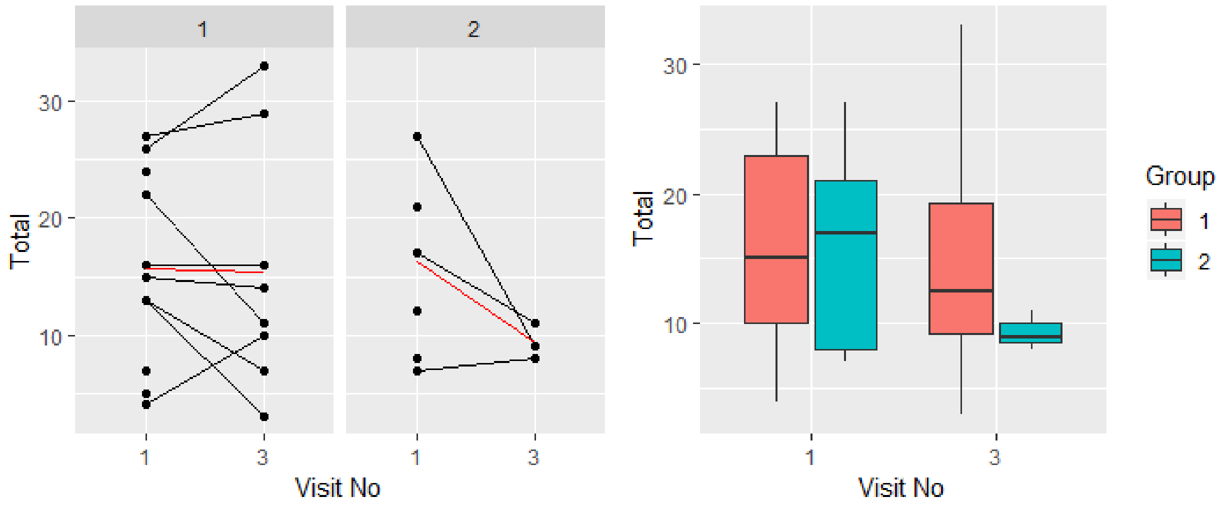

P-value = 0.3430
(the interaction between "Visit No" and "Group" type is not statistically significant at $\alpha = 0.05$)

cPLI ug/l (cPLI serum concentration) by group (Visit No = 1 ONLY)

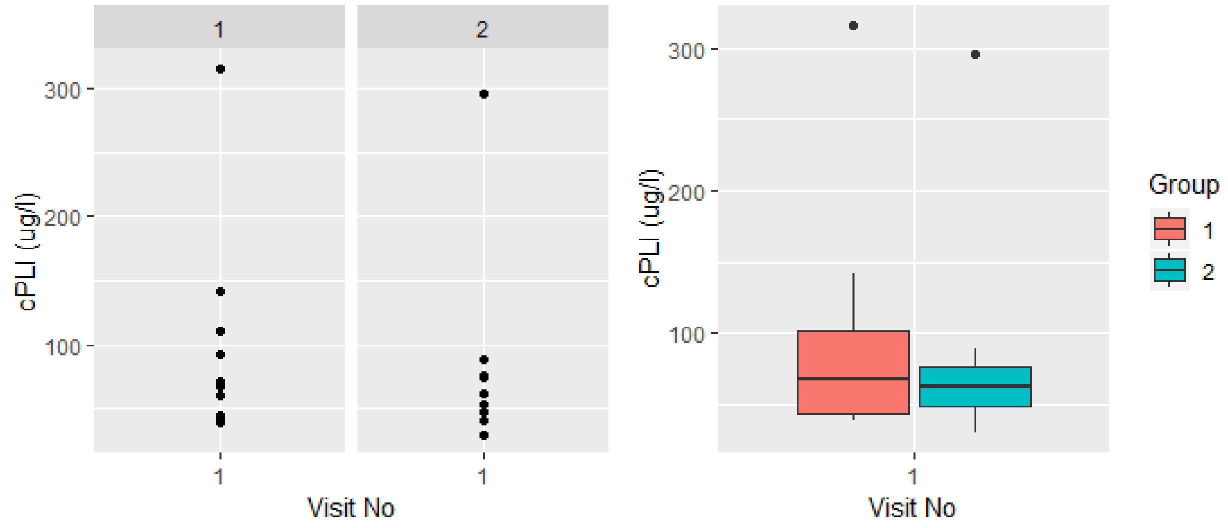

P-value = 0.8265 (no significant difference between two groups at $\alpha = 0.05$)

# cPLI, Outliers removed (RVB05, RVB32)

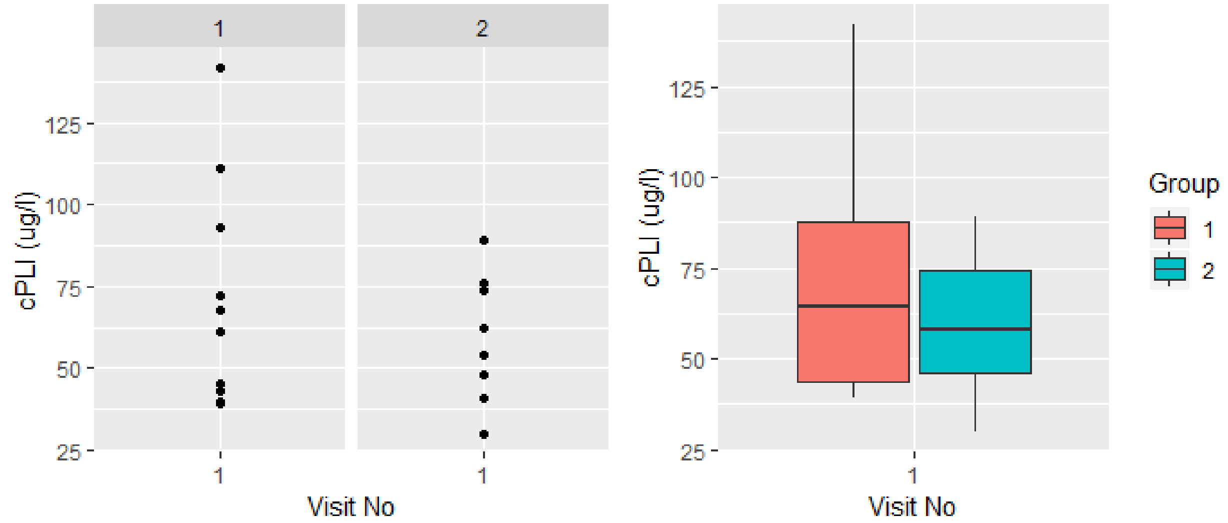

P-value = 0.3894 (no significant difference between two groups at $\alpha = 0.05$)

TLI ug/l (TLI serum concentration) by group (Visit No = 1 ONLY)

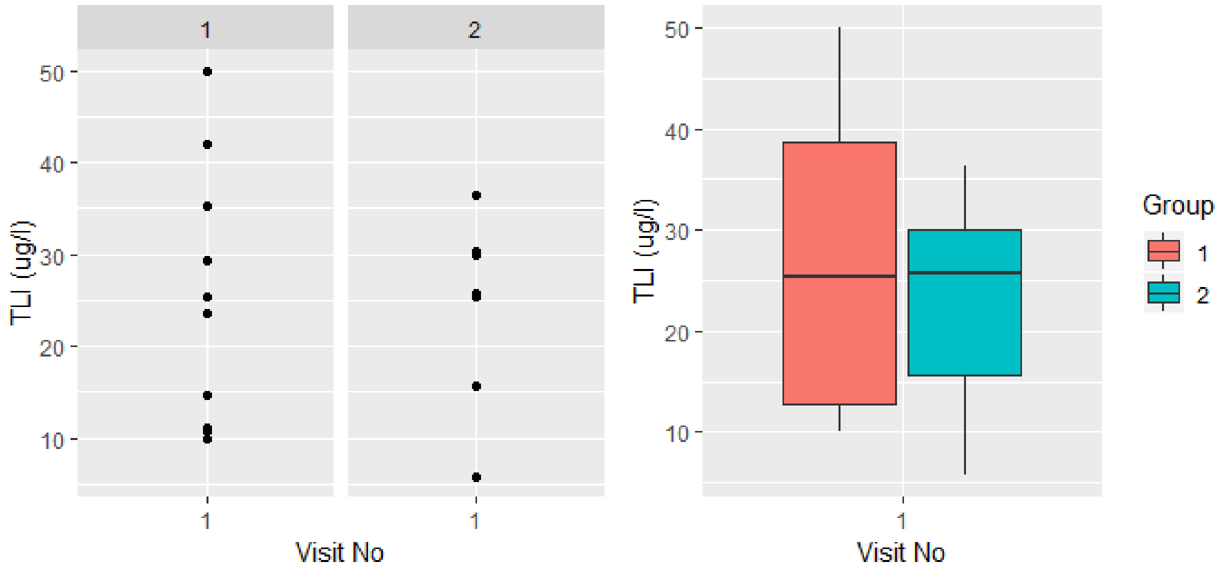

P-value = 0.5503 (no significant difference between two groups at $\alpha = 0.05$)

Alb g/l (serum albumin concentration) by group

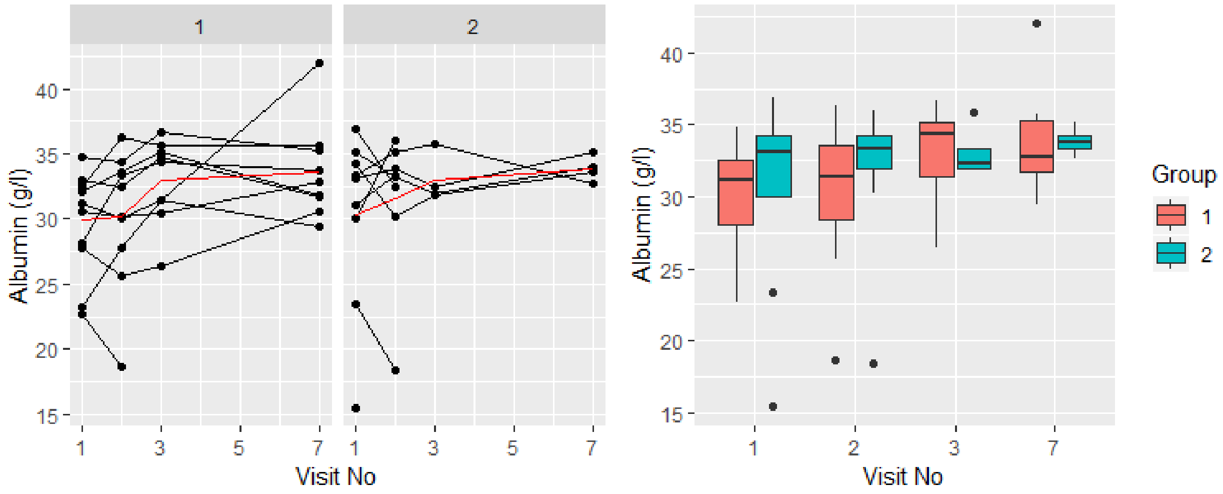

P-value = 0.1132 (no significant difference between two groups at $\alpha = 0.05$)

Glob g/l (serum globulin concentration) by group

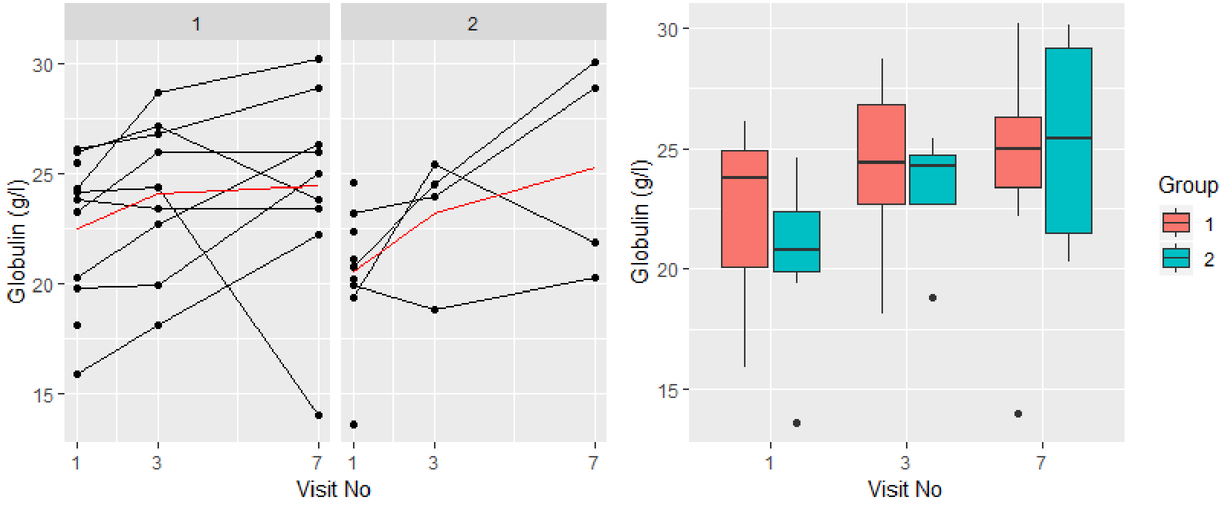

P-value = 0.7952 (no significant difference between two groups at $\alpha = 0.05$)

Folate ng/ml (serum folate concentration) by group

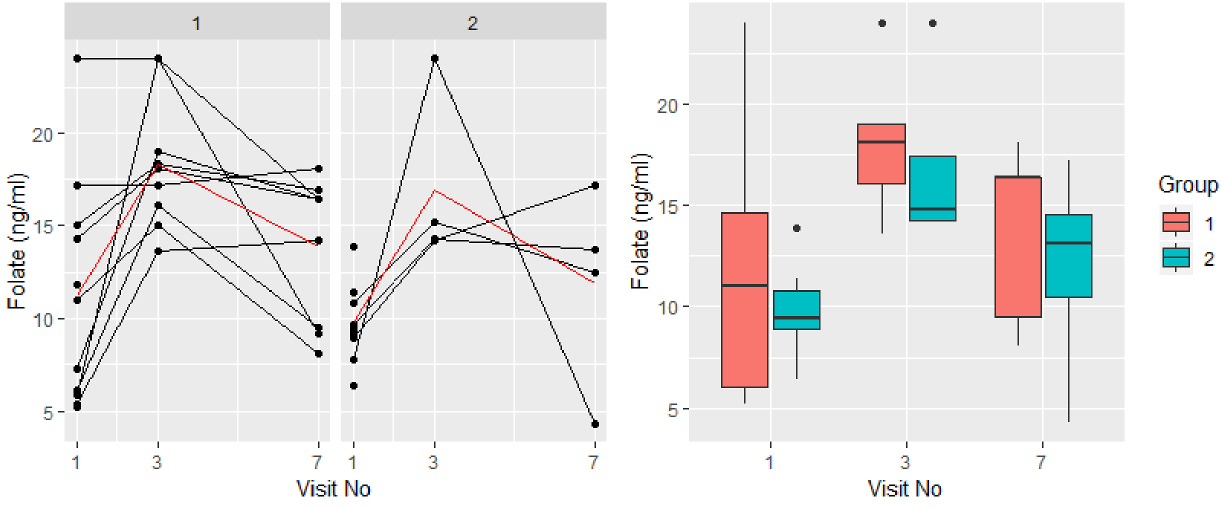

P-value = 0.2390 (no significant difference between two groups at $\alpha = 0.05$)

Cobal ng/l (serum cobalamin concentration) by group

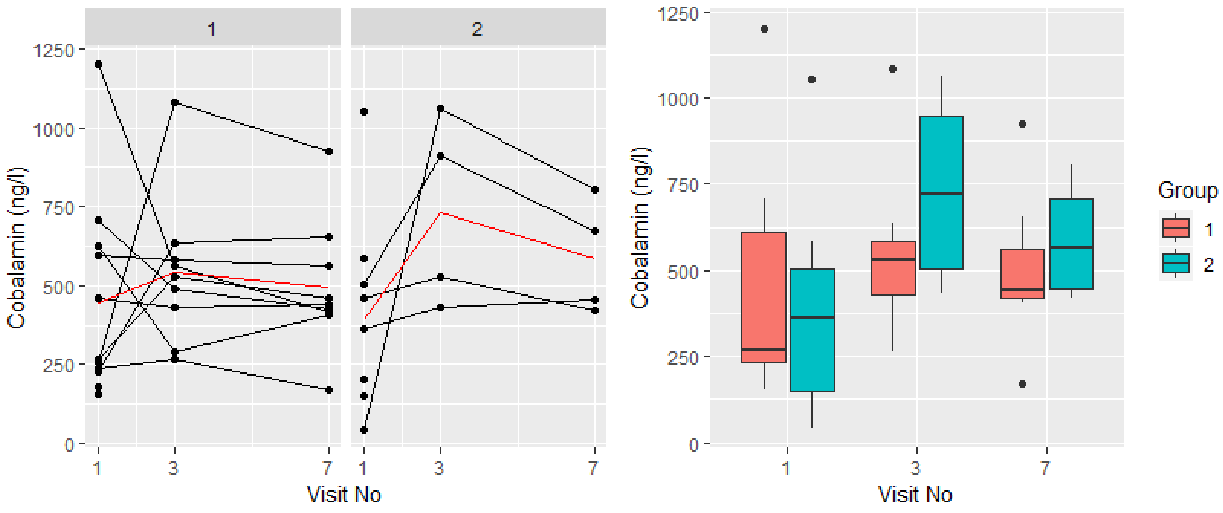

P-value = 0.3913 (no significant difference between two groups at $\alpha = 0.05$)

Correlations of all endoscopy score with CCECAI, CIBDAI and WSAVA scores "by Group"

Group=1

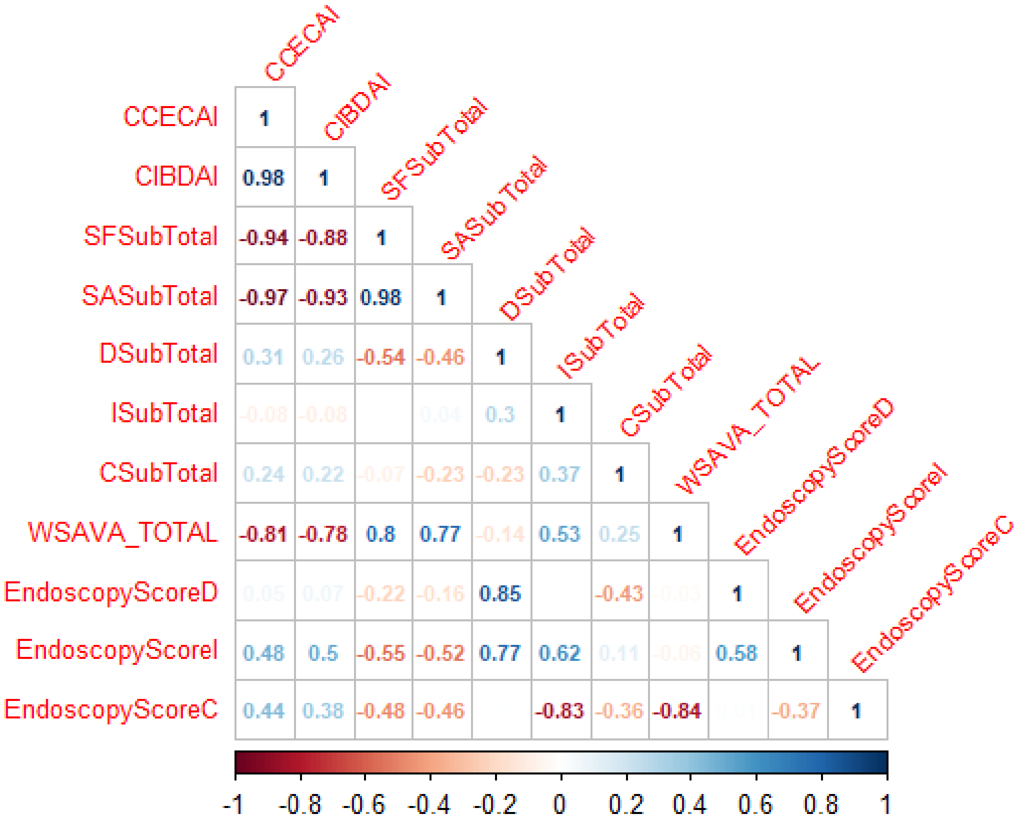

Group=2

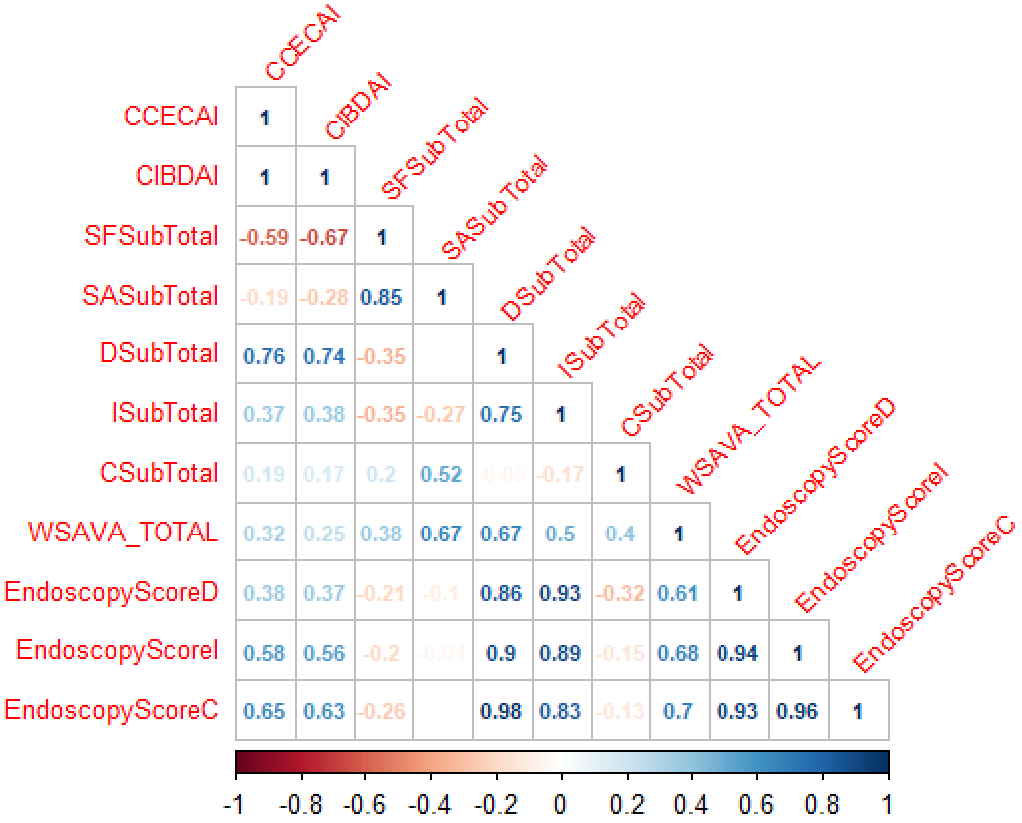

GROUP=1 & 2 Combined

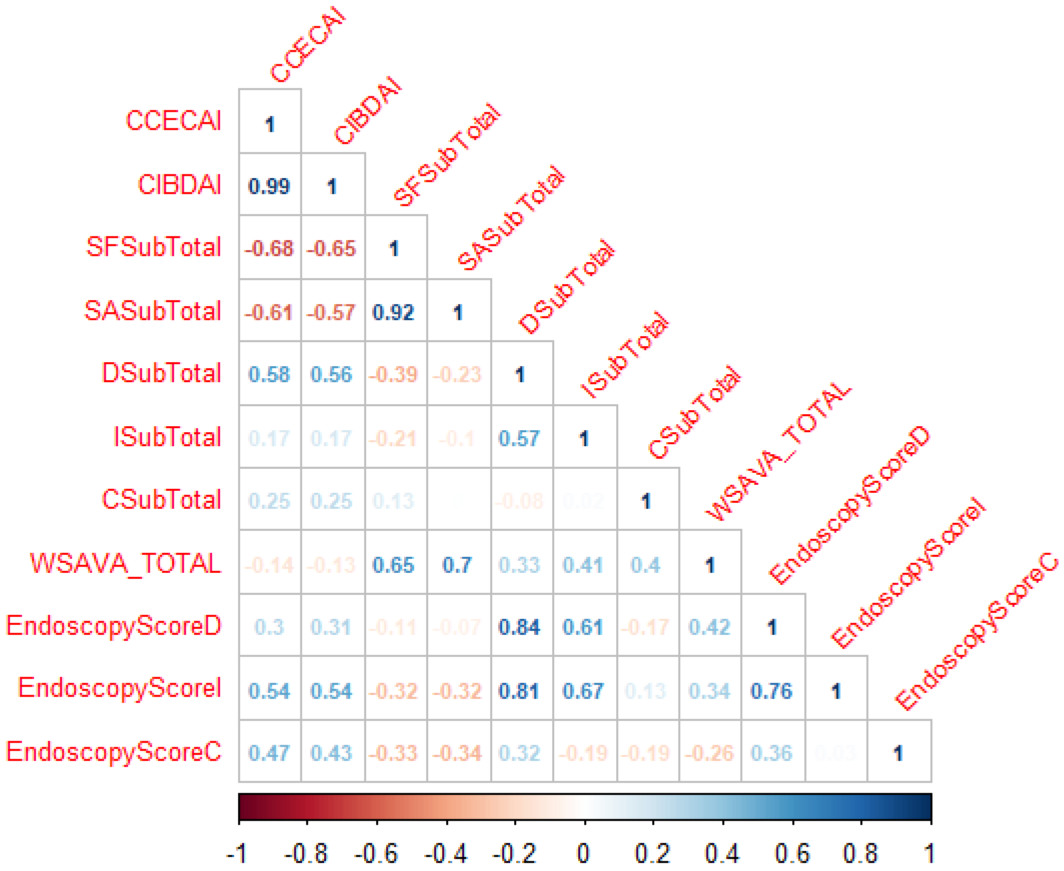

Serum albumin & cobalamin concentration with CCECAI, CIBDAI and WSAVA scores "by Group"

Group=1

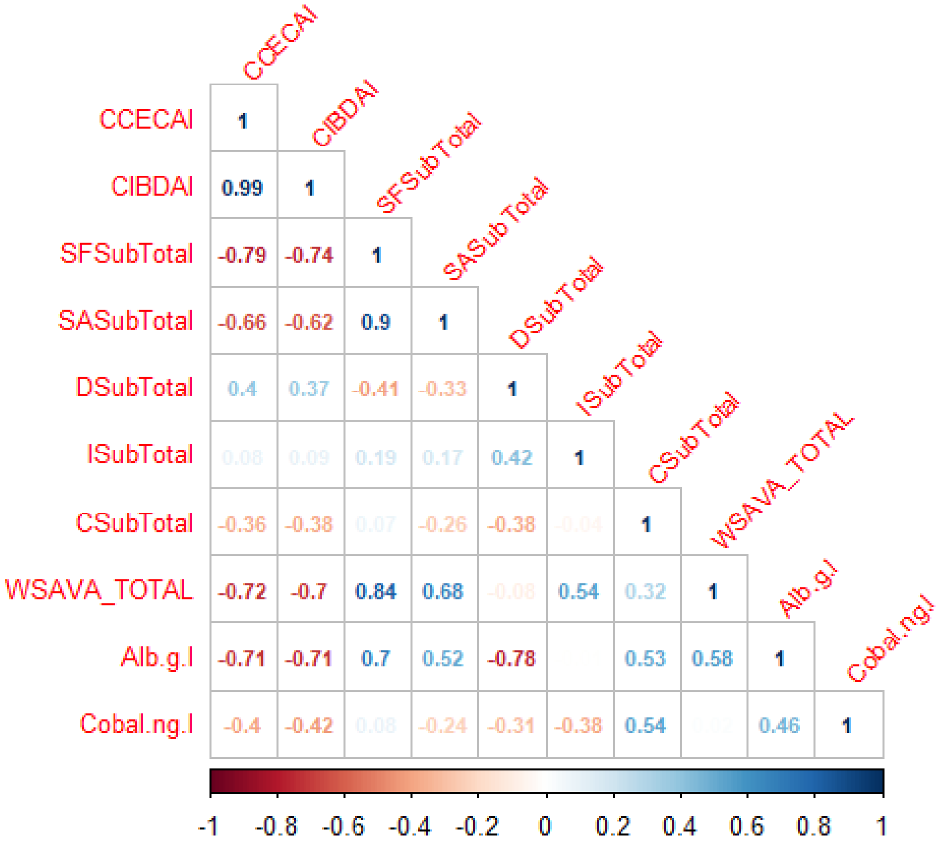

Group=2

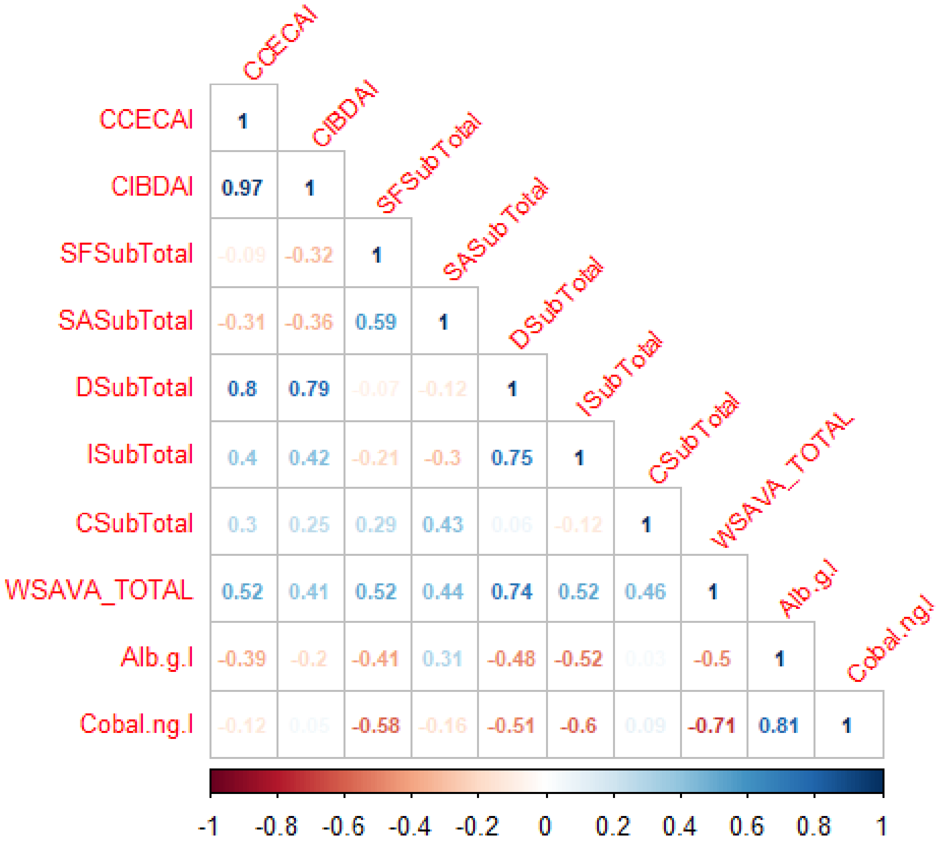

GROUP=1 & 2 Combined

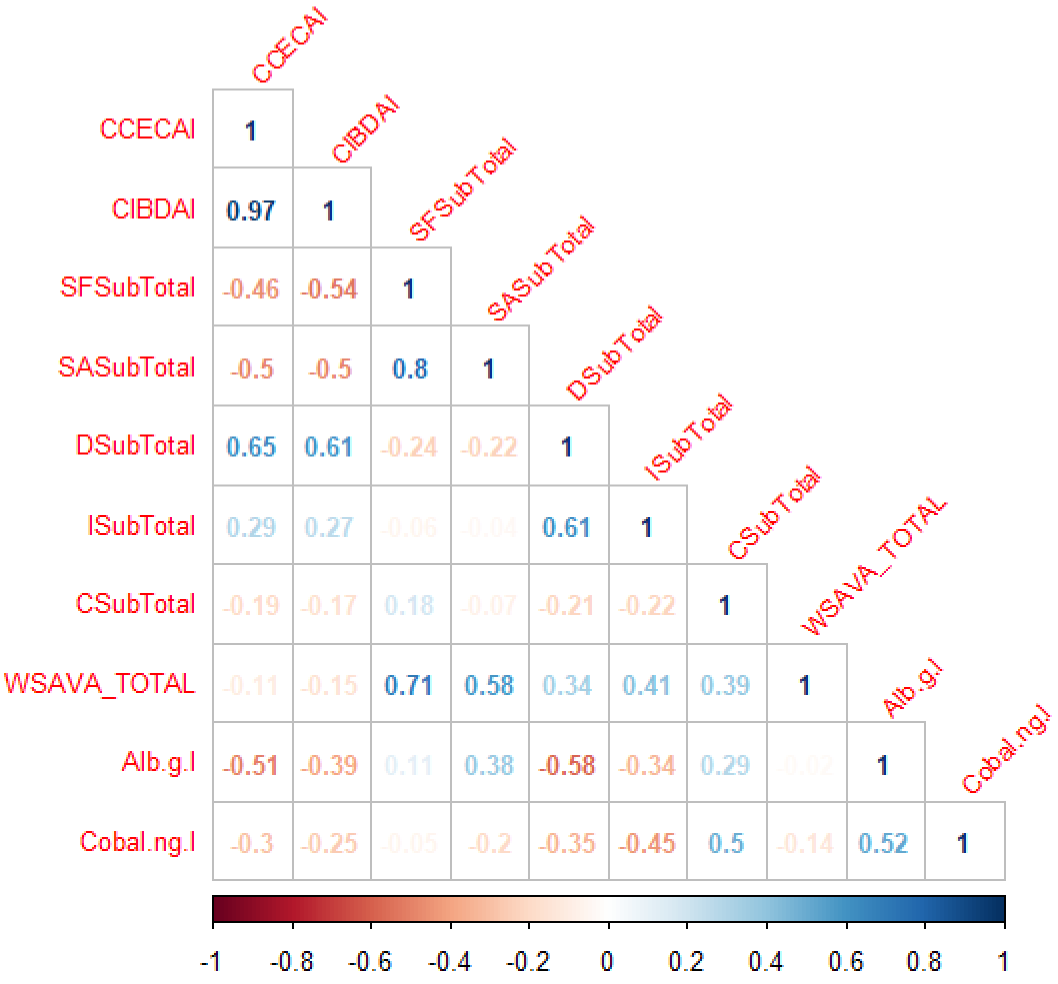

Supplement: S2 File — (PDF) [file pone.0250681.s002.pdf]
