# Supplementary material for: Clinical efficacy of prebiotics and glycosaminoglycans versus placebo In dogs with food responsive enteropathy receiving a hydrolyzed diet: A pilot study
Source: PLoS One. 2021 Oct 21;16(10):e0250681. doi: 10.1371/journal.pone.0250681 (PMC8530283; doi:10.1371/journal.pone.0250681)
Supplement: S3 File — (PDF) [file pone.0250681.s003.pdf]

Jan.2019

SCFA Bioiberica dogs (with 14 dogs total, 9 dogs in Trt 1 and 5 dogs in Trt 2)

Raw data | **Normalized with Internal standard**

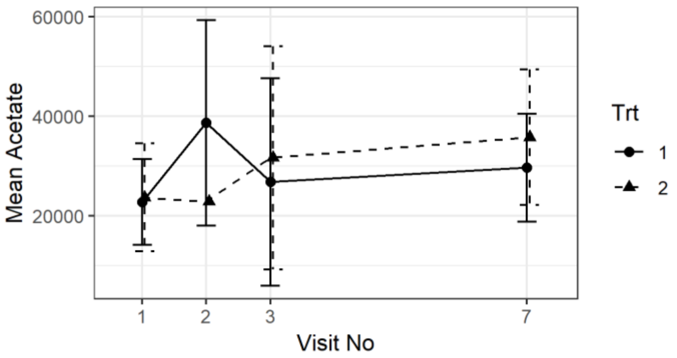

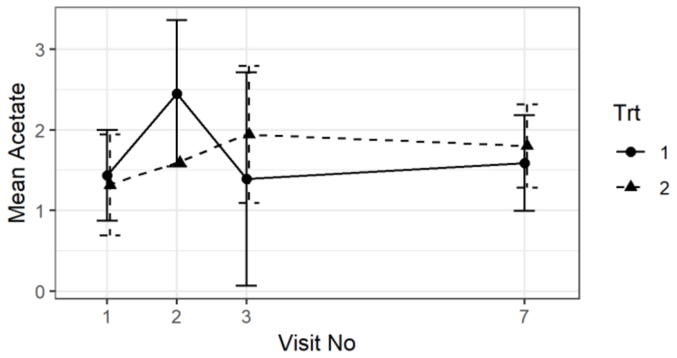

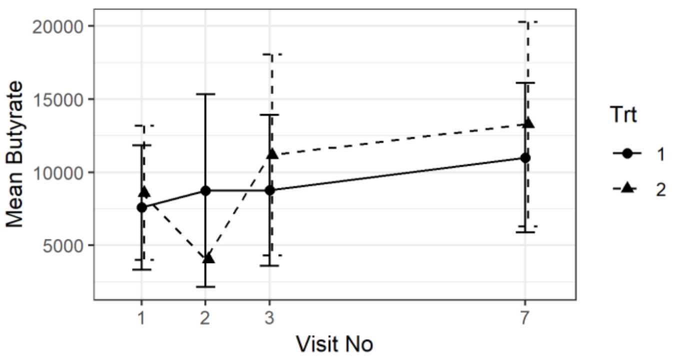

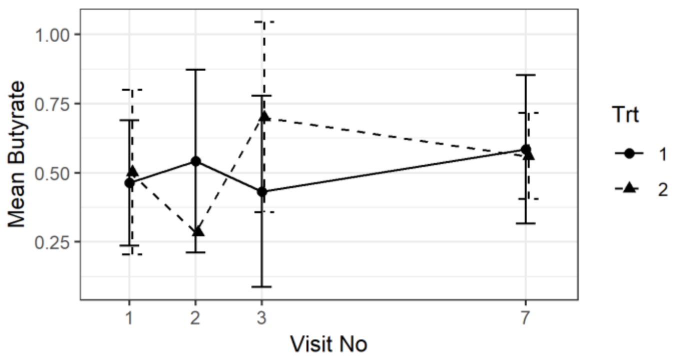

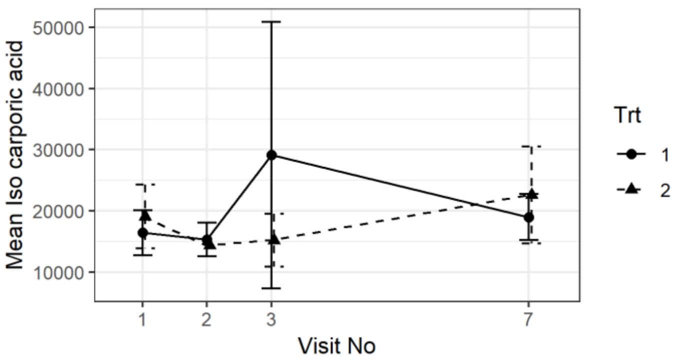

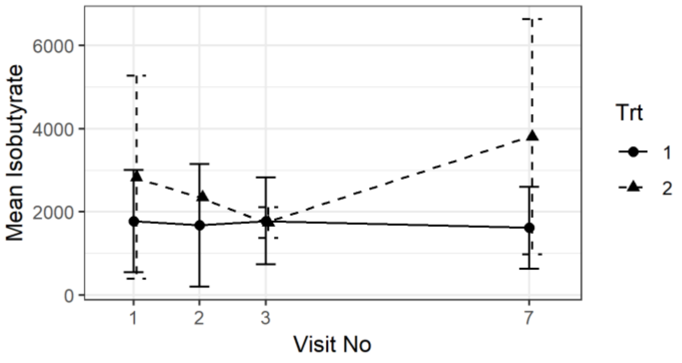

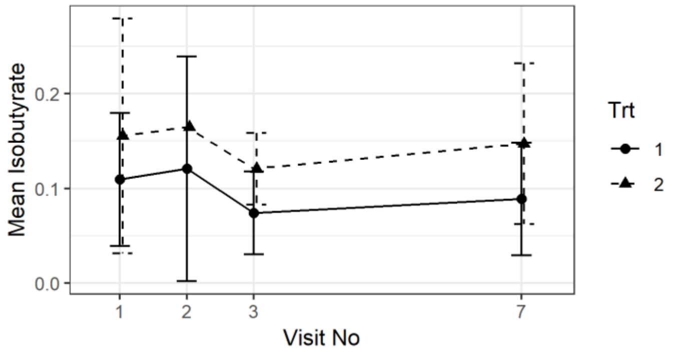

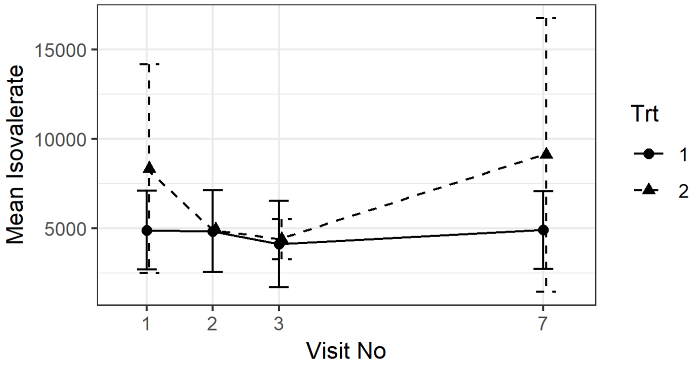
Mean Isovalerate
15000
10000
5000
1
2
3
7
Visit No
Trt
1
2

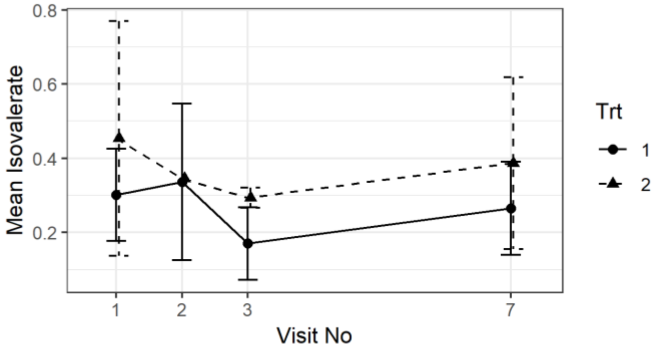
Mean Isovalerate
0.8
0.6
0.4
0.2
1
2
3
7
Visit No
Trt
1
2

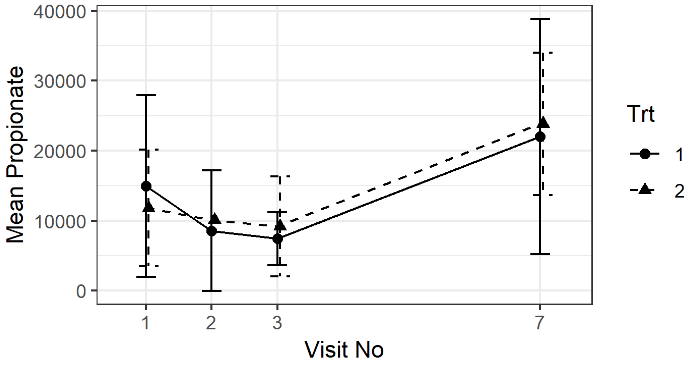
Mean Propionate
40000
30000
20000
10000
0
1
2
3
7
Visit No
Trt
1
2

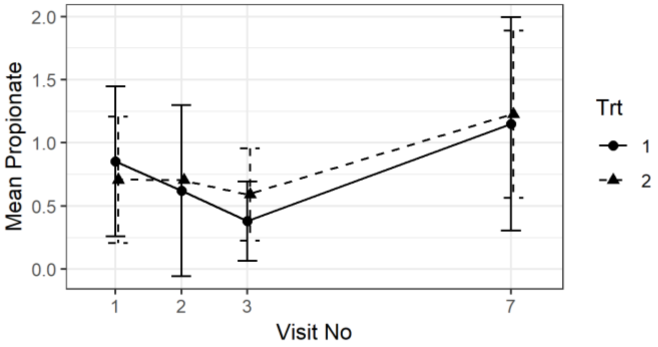
Mean Propionate
2.0
1.5
1.0
0.5
0.0
1
2
3
7
Visit No
Trt
1
2

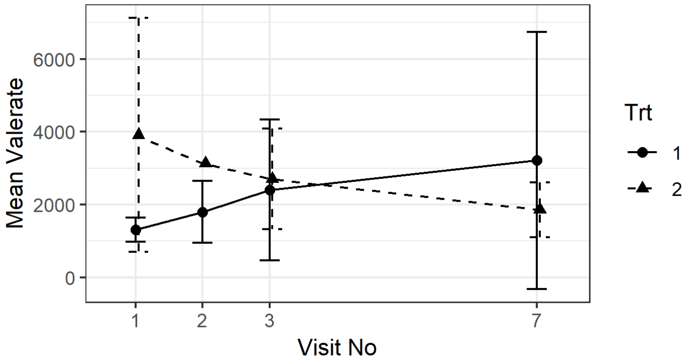
Mean Valerate
6000
4000
2000
0
1
2
3
7
Visit No
Trt
1
2

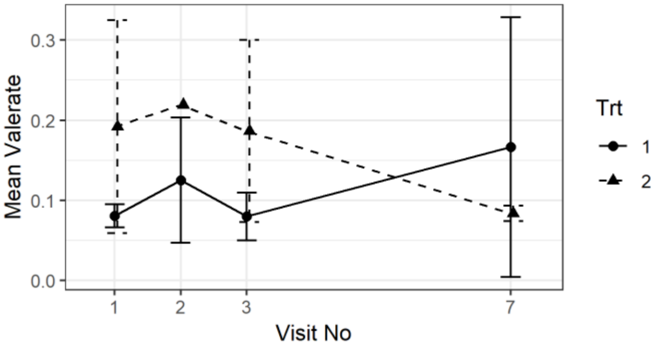
Mean Valerate
0.3
0.2
0.1
0.0
1
2
3
7
Visit No
Trt
1
2

1. Visits 1 and 2 are not used for comparisons since there is only one dog (Woody) in Trt=2, measured at Visit=2.

2. Visits=1, 3: Trt 1 vs Trt 2 (Raw data)

Testing for significant interaction using ANCOVA (one-way)

Raw data

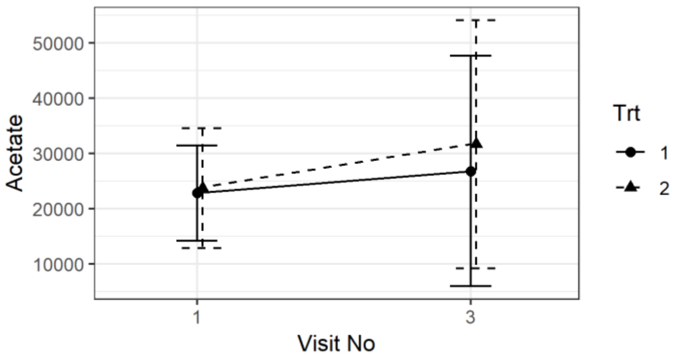

p-value = 0.7955

Normalized with Internal standard

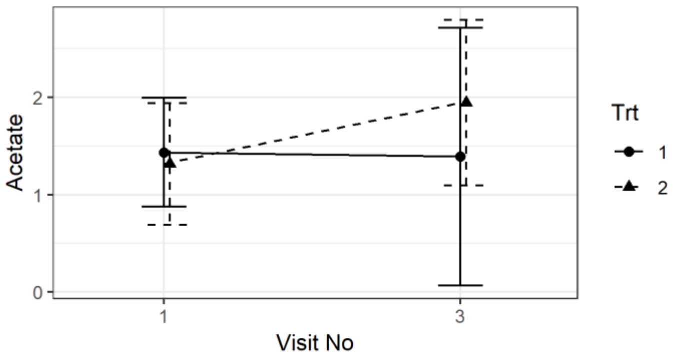

p-value = 0.4215

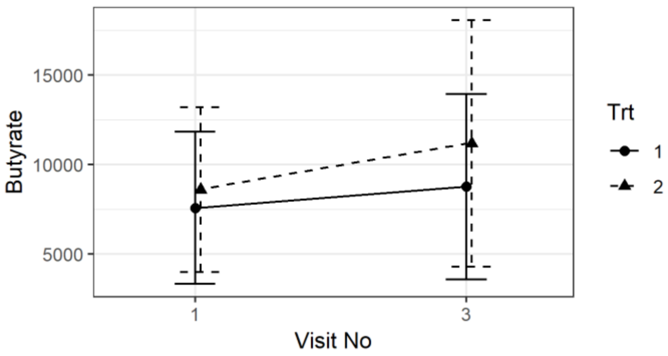

p-value = 0.7670

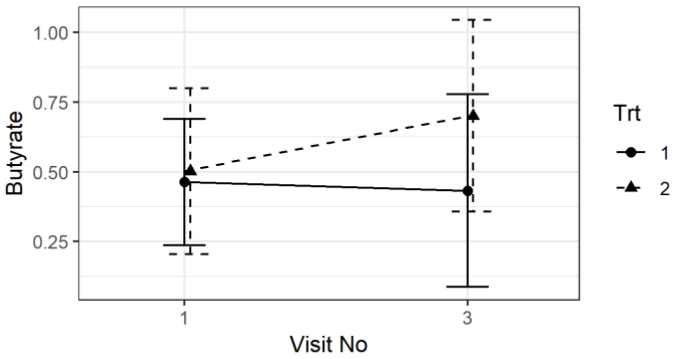

p-value = 0.4104

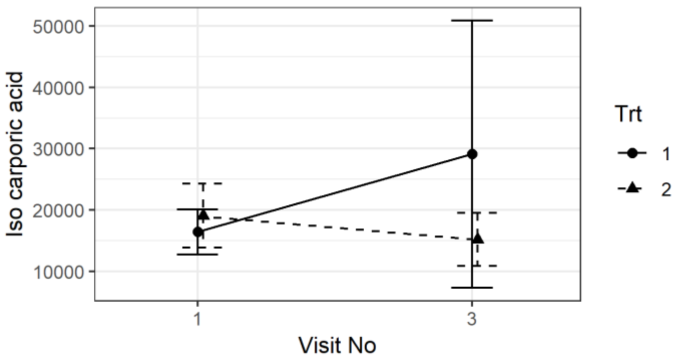

p-value = 0.1483

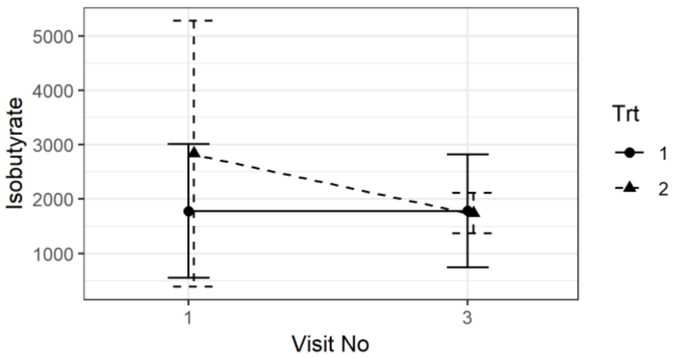

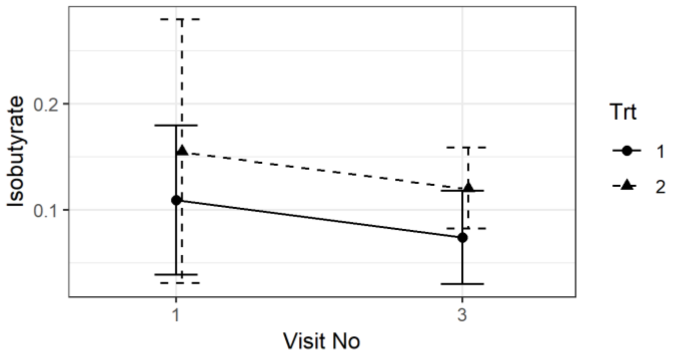

p-value = 0.3751

p-value = 0.9925

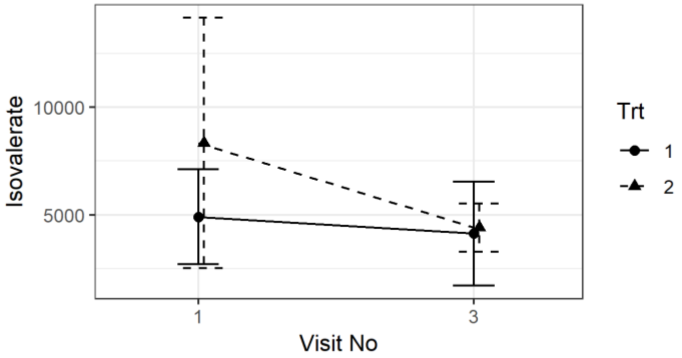

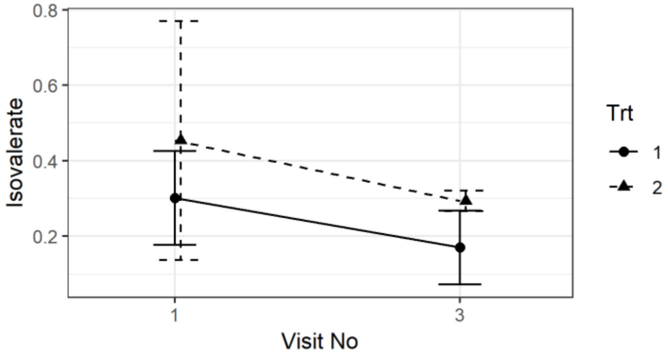

p-value = 0.2611

p-value = 0.8414

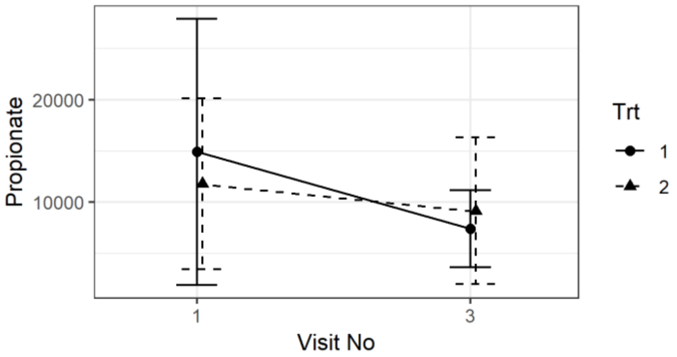

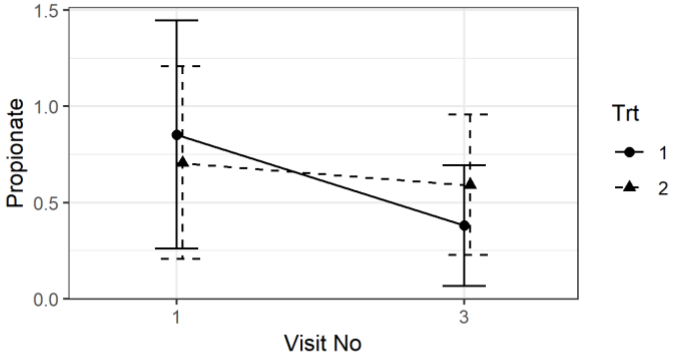

p-value = 0.5422

p-value = 0.3868

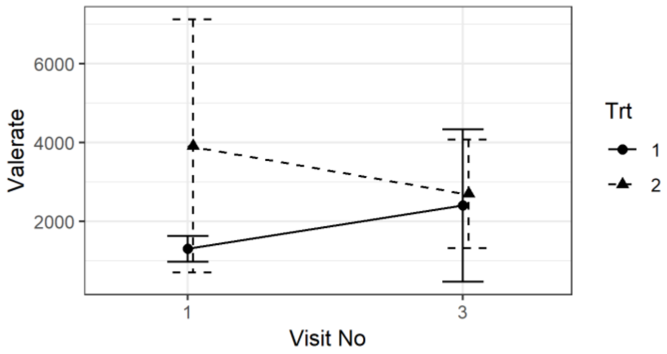

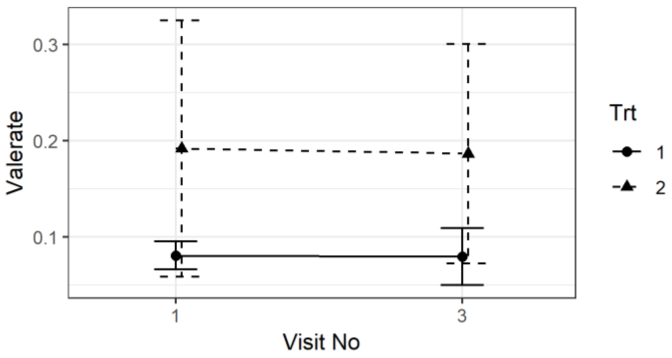

p-value = 0.1753

p-value = 0.9520

No significant interaction between treatment and visit No for all variables

3. Visits=3, 7: Trt 1 vs Trt 2 (Raw data)

Testing for significant interaction using ANCOVA (one-way)

Raw data

Normalized with Internal standard

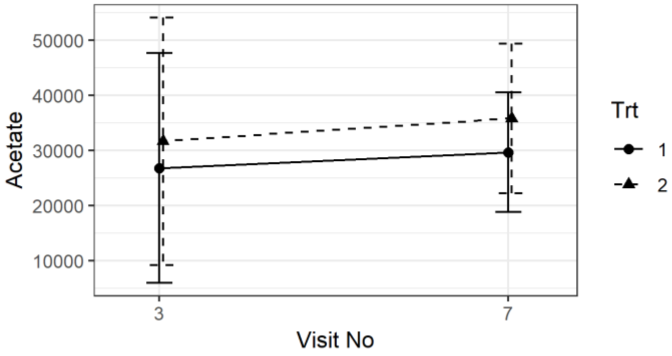

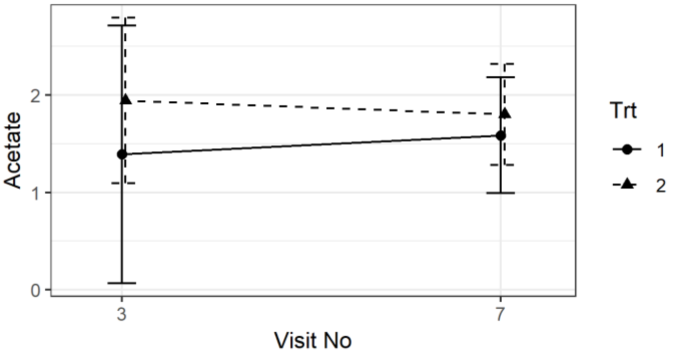

p-value = 0.9298

p-value = 0.6545

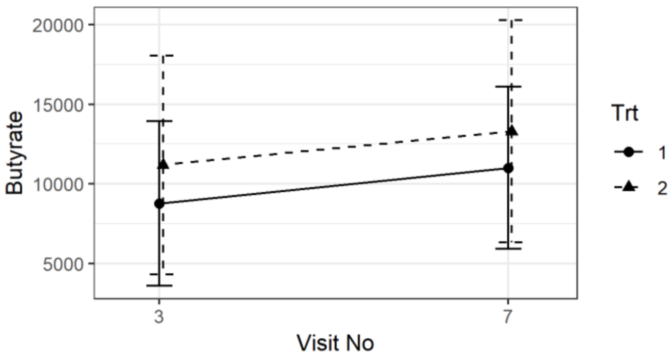

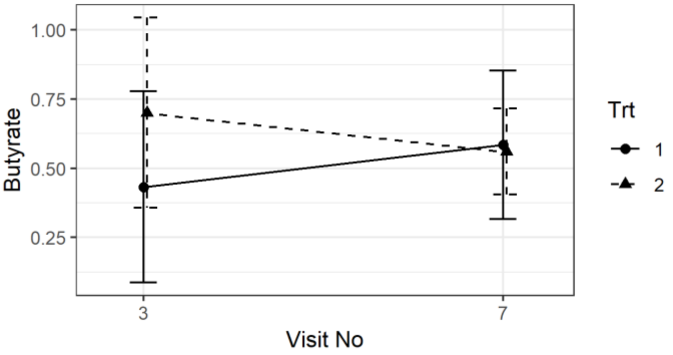

p-value = 0.9791

p-value = 0.2568

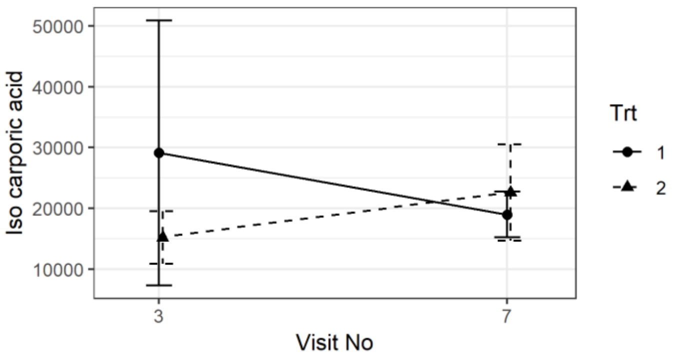

p-value = 0.0955

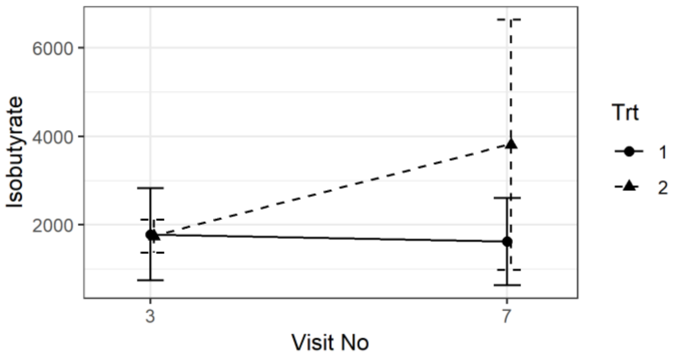

p-value = 0.0713

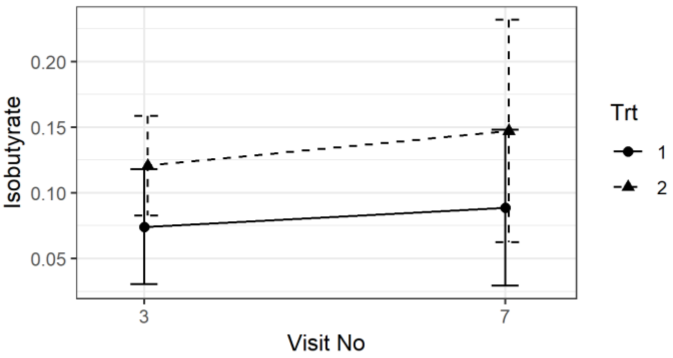

p-value = 0.8094

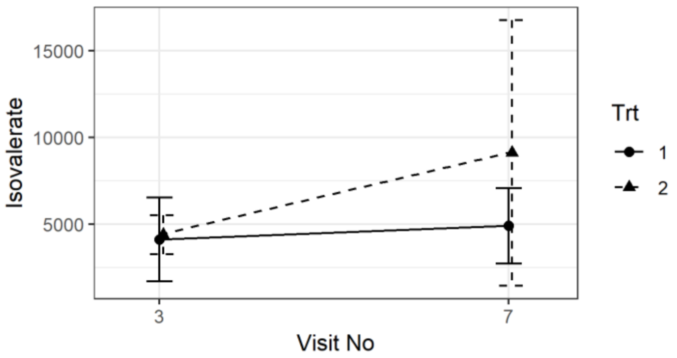

p-value = 0.2058

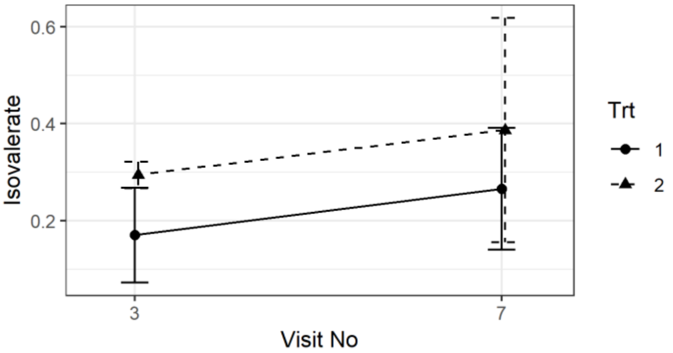

p-value = 0.9793

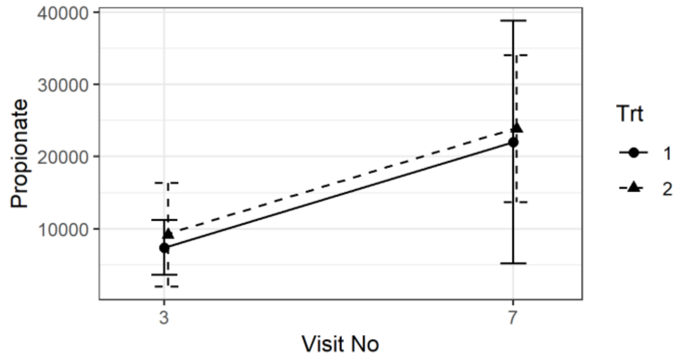

p-value = 0.9941

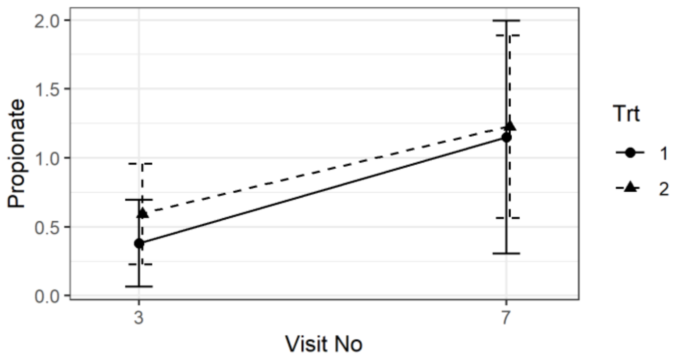

p-value = 0.8036

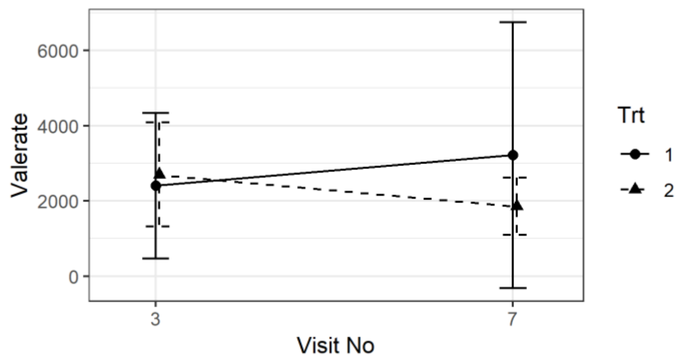

p-value = 0.4524

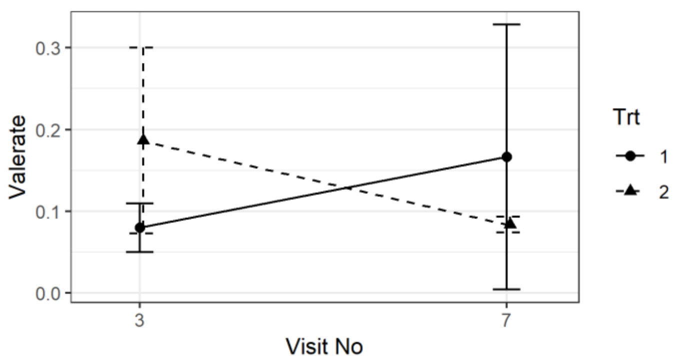

p-value = 0.0689

No significant interaction between treatment and visit No for all variables
